# Changes in breeding periodicity of a long-lived vertebrate over 30 years

**DOI:** 10.1098/rsos.260642

**Published:** 2026-09-09

**Authors:** Jared J. Tromp, Mohd Uzair Rusli, David T. Booth, Graeme C. Hays

**Affiliations:** ^1^ Deakin Centre for Marine Science, School of Life and Environmental Sciences, Deakin University, Geelong, Victoria, Australia; ^2^ Institute of Oceanography and Environment, Universiti Malaysia Terengganu, Kuala Nerus, Terengganu, Malaysia; ^3^ School of Environment, The University of Queensland, Brisbane, Queensland, Australia

**Keywords:** shifting baselines, demographics, remigration interval, sea turtles, marine megafauna, population recovery

## Abstract

Changes in breeding periodicity in vertebrates can highlight important trends in population demographics that have conservation implications. Following widespread conservation measures, some previously endangered species have shown population recoveries across their range, with green turtles (*Chelonia mydas*) being an exemplar of global species recovery. At sites in the Indo-Pacific, green turtle nesting numbers have increased fourfold in 30 years. As sea turtles are generally non-annual breeders, we used a total of 1399 measurements of the breeding interval for marked individuals to reveal that the relative proportion of individuals breeding with a 4-year versus a 3-year interval has increased over time: 28% in 1997–2012 versus 37% in 2013–2024. Furthermore, this percentage of 4- versus 3-year nesting intervals was linked to annual nest numbers (*R*^2^ = 0.23, *F*_1,23_ = 6.92, *p* = 0.01). This lengthening of the breeding (remigration) interval may reflect density dependence, restricting foraging success so that individuals need longer to build body condition for migration and breeding. These results provide the first evidence for long-term lengthening of breeding intervals in this taxon and might have important global conservation implications in terms of reduced per capita reproductive output, a finding that echoes recent observations with cetaceans.

## 1. Introduction

Set against the background of international efforts to increase global ocean conservation such as the Kunming–Montreal Global Biodiversity Framework (which sets out to protect and restore 30% of the world's land and seas globally by 2030: [1]), there are notable exemplars showing how conservation efforts can be effective and drive species recoveries. For example, following a moratorium on commercial whaling, humpback whale (*Megaptera novaeangliae*) numbers have increased from a few hundred to over 40 000 around eastern Australia [2]. As well as species recoveries in specific localities, there are also notable exemplars of global species recoveries, with one of the most notable being the green turtle (*Chelonia mydas*) that were listed by the IUCN as ‘endangered’ in 2004 but in 2025 this listing was changed to ‘least concern’ [3,4] owing to recoveries in nesting numbers at many sites around the world. These conservation successes show what is possible if direct and bycatch mortality are reduced through, for example, the creation of conservation zones and international treaties to reduce harvest and trade [5,6]. Although population recoveries are to be applauded, they might have unexpected demographic consequences. For example, for terrestrial mammals it has been known for many decades that density-dependent processes may operate, with reduced foraging success and reproductive output at high animal densities (e.g. [7]). High population densities have also been shown to degrade habitat through overgrazing [8,9]. Density-dependent processes might also act synergistically with climate change-induced changes in food availability [10–12]. For marine mega-fauna, as time series lengthen, it will start to be possible to assess whether long-term changes in key demographic features might be linked to density dependence or climate change. It is important to identify these long-term demographic changes to fully understand obstacles to species recoveries. For example, the southern right whale (*Eubalaena australis*) has shown declining reproductive output over time in southern Australia, with more and more individuals skipping breeding years [13]. The reasons for this decline and the consequences are both uncertain [13].

Recently, conservation efforts with some marine megafauna have led to expanding populations and resultant density-dependent food limitations. For example, high densities of green turtles have been observed over-grazing seagrass meadows [14]. Similarly, with juvenile hawksbill turtles (*Eretmochelys imbricata*) at a fully protected coastal foraging site, an exceptionally high density of individuals has been reported [15] that is linked to an exceptionally slow growth rate, probably owing to density-dependent food limitation [16]. However, another possibility for sea turtles at some sites is that even with recent population increases, numbers might still be orders of magnitude below historic levels that existed centuries ago before intense human exploitation, a concept termed shifting baselines [17]. In this scenario, recovering populations might still be a long way from experiencing density-dependent food limitation.

Sea turtles are generally non-annual breeders, owing to the invariant costs of migration and breeding [18]. For example, in captive sea turtles, the interval between breeding seasons (termed the remigration interval) is shorter than for wild turtles because of higher food availability combined with no migration costs [19]. As a corollary, it might be expected that if food availability decreases for wild turtles, for example, because of density-dependent processes, then their remigration intervals might lengthen. In support of this hypothesis, in several sea turtle species, inter-annual variability in remigration intervals has been linked to weather conditions which might serve as a proxy for foraging conditions [20,21]. However, sufficiently long time series to assess systematic long-term changes in remigration intervals have yet to be investigated. Here, we examine long-term changes in remigration intervals for a population where nesting numbers have shown a marked increase over 30 years and where marking of individuals allows their breeding periodicity to be assessed.

## 2. Material and methods

### 2.1. Study site

**Figure 1.**
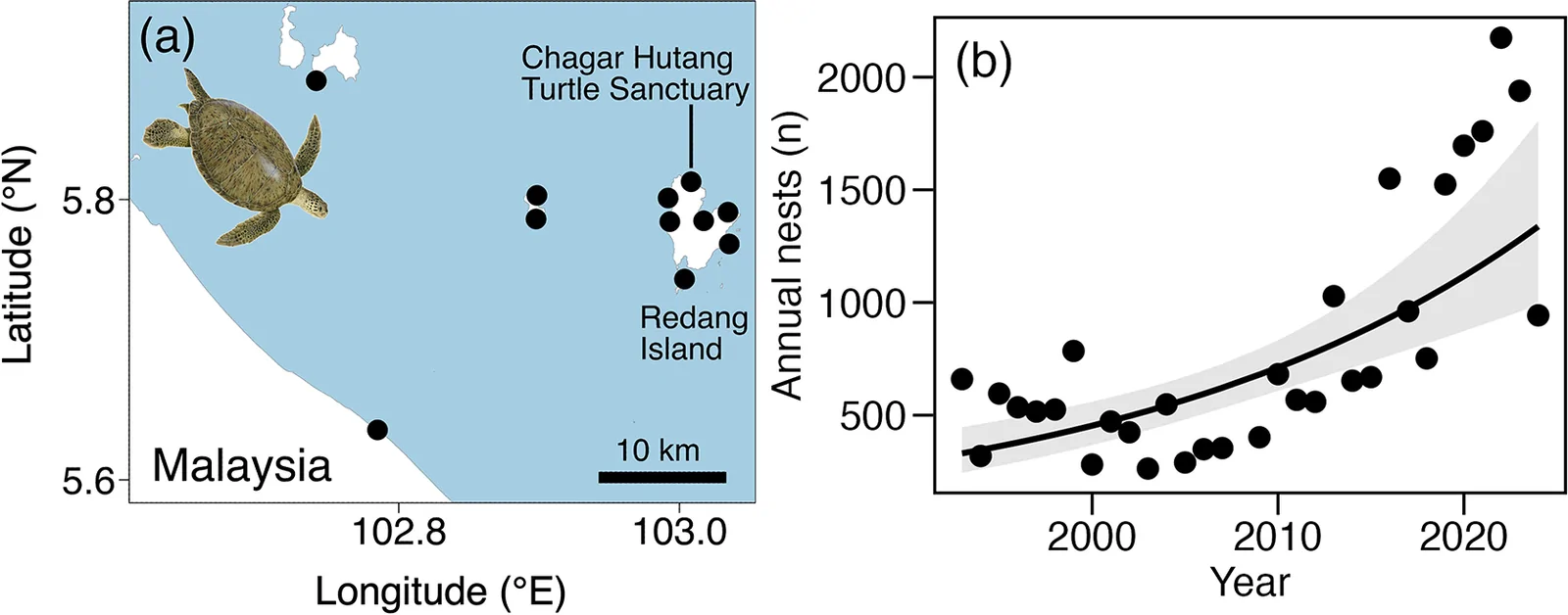
(a) Green turtles (*C. mydas*) at Chagar Hutang Turtle Sanctuary were monitored from 1993 to 2024. Turtles nest at multiple beaches on islands and mainland coasts (black points). However, the nesting beach at Chagar Hutang Turtle Sanctuary is the only dedicated tagging and recapture programme, occurring since 1993. (b) Annual nest numbers have increased over the monitoring programme, with the largest number of nests reported in the most recent years. The solid line represents the predicted exponential trend (*R*^2^ = 0.52, *F*_1,29_ = 30.8, *p* < 0.01) with shaded 95% confidence intervals. Green turtle image provided by NOAA Fisheries. Malaysian coastline derived from OpenStreetMap data OpenStreetMap contributors.

Green turtles were monitored during the nesting season (April to October) at the Chagar Hutang Turtle Sanctuary (5.81°N, 103.01°E) on Redang Island, Malaysia, by nightly foot patrols. The Turtle Sanctuary consists of a short beach (approx. 350 m) where ongoing nightly monitoring has been conducted throughout 1993–2024. During this time, nests are counted, and turtles have been tagged with Inconel flipper tags on both front flippers, with replacement tags applied when needed. The study site and methodology have previously been described [22]. Although nest counts are made on beaches across Redang Island by other groups, the Turtle Sanctuary is the only site where intensive long-term tagging and identification have taken place (figure 1a).

### 2.2. Assessing beach patrol efficiency

There are several reasons why tagged turtles might not be re-sighted, including tags falling off quickly, turtles nesting on unmonitored beaches, or turtles being missed nesting on the study beach (beach patrol efficiency). We gauged the level of beach patrol efficiency by examining the frequency distribution of re-sighting intervals within a season. If turtles were usually re-sighted after one inter-nesting interval, then this suggests high beach patrol efficiency.

### 2.3. Mitigating artefacts in trends in remigration intervals

We examined the inter-nesting and remigration intervals for individually tagged turtles. This was done by counting the number of days between observations for an individual turtle within a year to give the inter-nesting interval. Similarly, we counted the number of years between repeat observations of the same turtle to calculate the remigration interval.

Although individuals might have recorded long (5+ years) remigration intervals, it is feasible that they might nest unobserved on alternative beaches and so are sometimes not identified in a year when they nest, even if there is high beach patrol efficiency on the focal beach. Turtles nesting unobserved would artificially inflate their remigration interval. So when the pattern of remigration intervals for an individual is assessed, some changes might be real whereas others might be sampling artefacts, such as a shift from a 2- to a 4-year remigration interval or a shift from a 3- to a 6-year interval, that is, turtles returning with a remigration interval that is a multiple of their modal interval. We used an index for any systematic change in remigration interval that would not be affected by a turtle being missed nesting in 1 year. So rather than calculate a mean remigration interval each year, which might include these sampling artefacts owing to unobserved nesting, we instead investigated the relative change in remigration intervals from 3 to 4 years, as a switch from 3 to 4 years (or vice versa) is unlikely to be the result of a turtle nesting unobserved, that is, the trend between this ratio probably represents a real change of increasing or decreasing nesting periodicity.

Data were analysed in R v4.5.2 using RStudio v2025.09.2 [23], and maps were produced using QGIS v3.36.2.

## 3. Results

**Figure 2.**
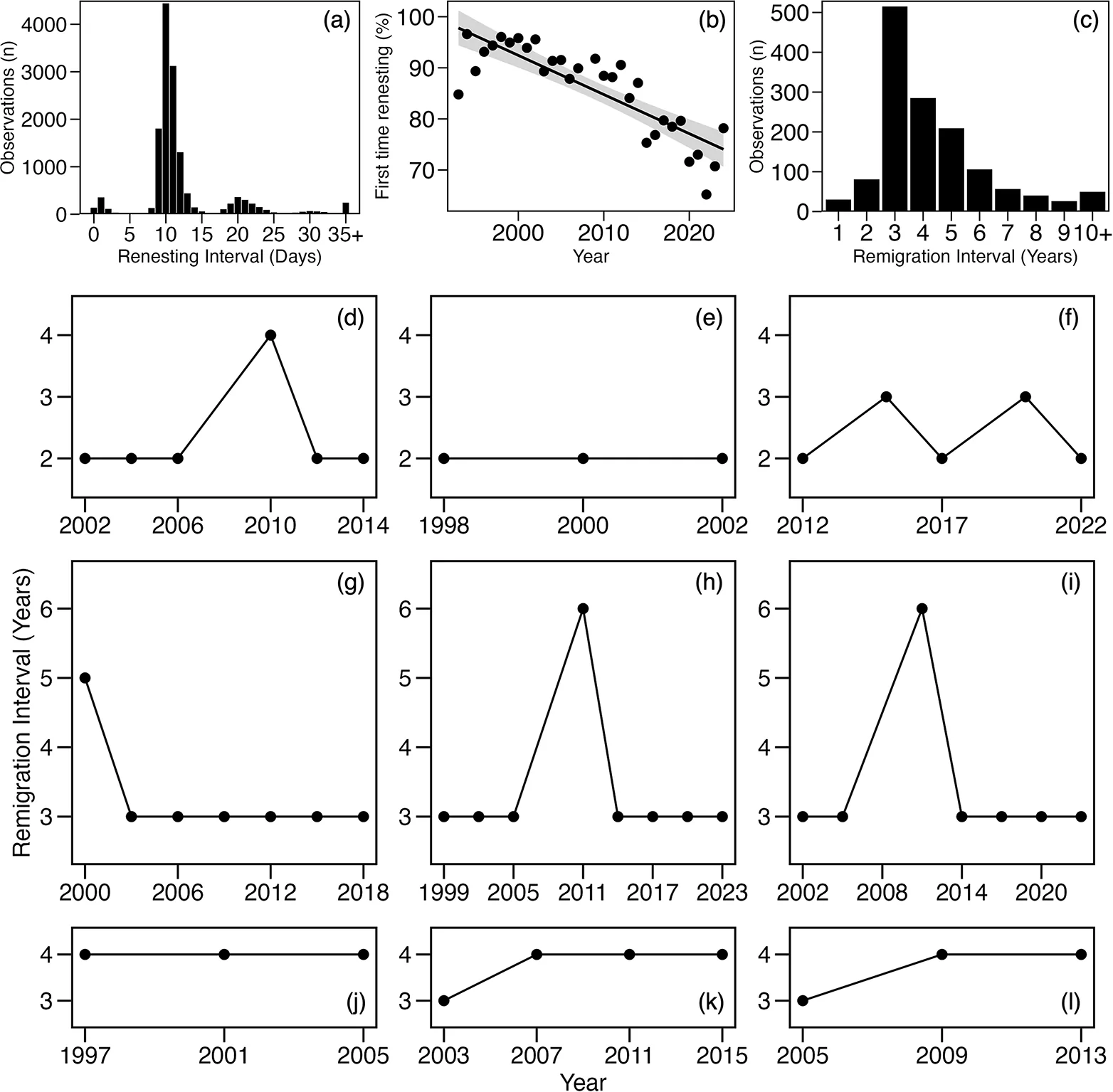
(a) Green turtles had a modal inter-nesting (re-nesting) interval of 10 d, representing the time between consecutive clutches. (b) The proportion of turtles re-nesting for the first time (8–15 d) was compared with the sum of re-nesting intervals (excluding false crawl observations of 0–7 d) for each year, showing a decreasing trend in beach patrol monitoring efficiency but remained more than 70% for the majority of the study period. The solid line represents the predicted linear trend (*R*^2^ = 0.7, *F*_1,29_ = 69.01, *p* < 0.01) with shaded 95% confidence intervals. (c) The modal remigration interval, counting the time between when a turtle was initially recorded and when it was next observed, was 3–4 y. (d–l) Individual remigration intervals of green turtles show stable, switching, and variable patterns in consecutive remigrations.

The annual number of green turtle nests at Chagar Hutang has increased, from around 400 to 500 nests annually at the start of the time series to around 1500–2000 more recently, that is, a three- to fourfold increase over 30 years (Figure 1b). Across 32 years of monitoring, 4609 individual green turtles were tagged. Although a large number of turtles (*n* = 2117) were only seen once, more than half were seen multiple times, with one individual repeatedly seen up to 79 times across 28 years. Turtles seen only once are largely from the recent increase in population numbers, with these individuals being tagged, but they have had insufficient time to remigrate with an interval of 4 or more years. The modal inter-nesting interval was 10 d, with a second peak occurring at 20 d (figure 2a). Turtles with a short (0–7 d) re-sighting interval were those that had emerged onto the beach but did not nest, termed a false-crawl. The study beach was efficiently patrolled to intercept most nesting turtles, but some turtles might have nested elsewhere at alternative sites. We defined the beach patrol efficiency as the percentage of turtles observed re-nesting with an interval in the first mode (8–15 d) versus those observed re-nesting with an interval of more than 15 d. The beach patrol efficiency decreased during the time series (*R*^2^ = 0.7, *F*_1,29_ = 69.01, *p* < 0.01), but remained >70% for the majority of the study period, decreasing 0.77% a year, from 97.8% in 1993 to 74.0% in 2024; that is, turtles had a good chance of being re-sighted if they re-nested on the study beach (figure 2b).

Across years, the modal remigration interval was 3 years (37% of observations), with appreciable numbers of turtles also observed with a 4-year (20% of observations) and 5-year (15% of observations) interval (figure 2c). There were some consistent differences between individuals in their remigration intervals (figure 2d–l). For example, for some individuals there were examples of a recorded remigration interval of 3 years on many occasions and then 5–6 years on rare occasions (figure 2g–i). Other individuals generally had a remigration interval of 2 years and then 4 or more years occasionally (figure 2d). In contrast, individuals were observed to maintain a consistent remigration interval of 2 and 4 years across all records (figure 2e and j), whereas others alternated between 2 and 3 years (figure 2f). In some cases, there is evidence that the remigration interval was increasing, changing from 3 to 4 years (figure 2k and l).

**Figure 3.**
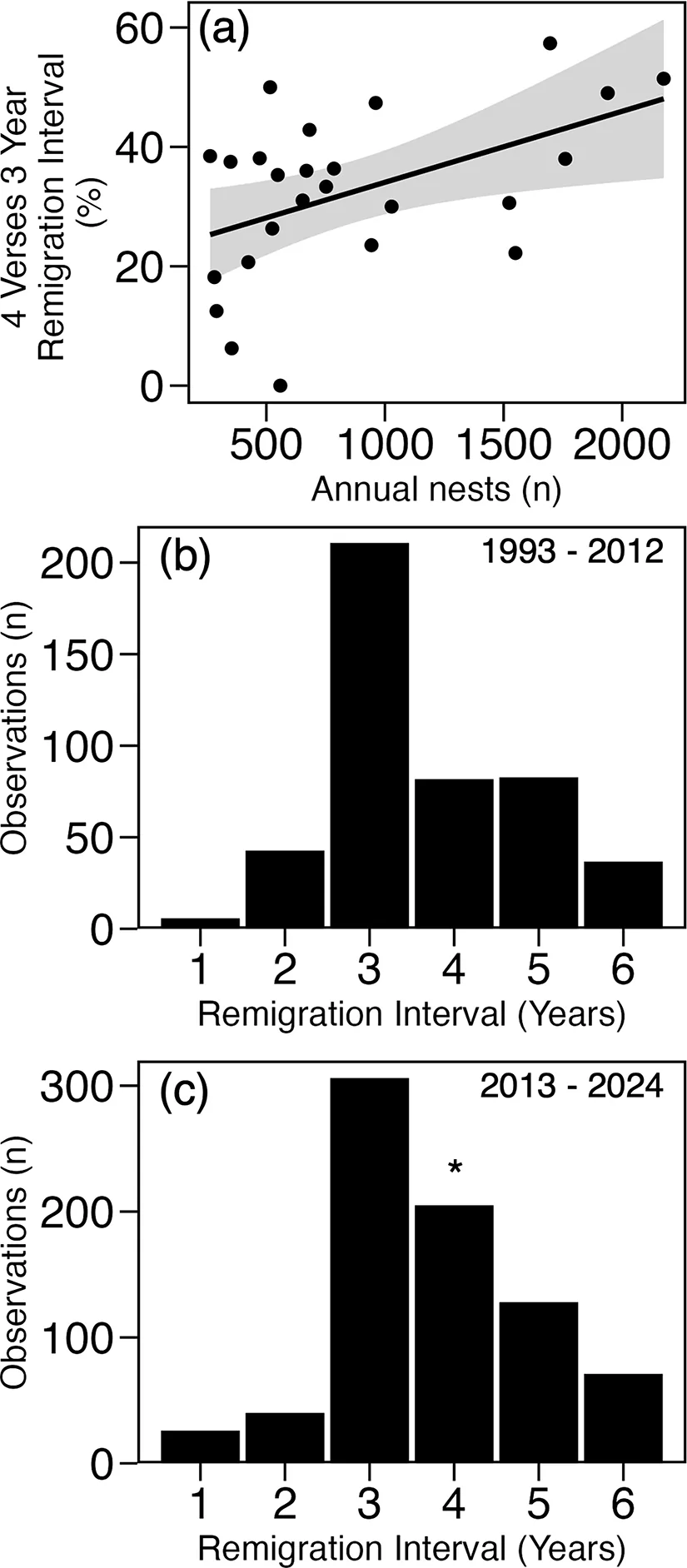
(a) Changes in the annual proportion of 4- versus 3-year remigration intervals as nest numbers increase. Solid line represents the predicted linear trend (*R*^2^ = 0.23, *F*_1,23_ = 6.92, *p* < 0.05) with shaded 95% confidence intervals. (b) Histograms for the remigration intervals (1–6 y) for the first (1993–2012) and (c) the second half (2013–2024) of the study period. There was a significantly (*) greater proportion of turtles returning with a 4-year remigration interval at the end of the study period compared with the start (*X*^2^_4_ = 25.33, *p* < 0.01).

The percentage of 4- versus 3-year remigration intervals increased (*R*^2^ = 0.23, *F*_1,23_ = 6.92, *p* < 0.05) as the annual number of nests increased (figure 3a). Furthermore, the proportion of 4- to 3-year intervals increased significantly (*X*^2^_4_ = 25.33, *p* < 0.01) between the start (1993–2012) and end (2013–2024) of the time series (figure 3b and c). The percentage of turtles returning with either a 3- or 4-year remigration interval was 64% and 66% of all remigration intervals, up to 6 years for 1993–2012 and 2013–2024, respectively. There was no significant difference (*p* = 0.17) between the start and the end of the study for 1-year remigration intervals. This lengthening of the remigration interval will decrease the mean annual per capita reproductive output, as female turtles extend their remigration by an additional year. For example, in the simplest terms, if the percentage of female turtles remigrating with a 3-year interval shifts from 80% to 50% of turtles nesting, then this shift would reduce the mean per capita reproductive output of eggs by 8% for those individuals, as more turtles require an additional year (4-year remigration interval) to reproduce. Such modelling could be extended if other systematic changes in remigration intervals are identified.

## 4. Discussion

The three decades of beach monitoring for nesting green turtles that we report are one of the longest published records of breeding periodicity for this species and highlight the importance of conducting longitudinal research. We report a marked increase in the annual number of green turtle nests over recent decades, in common with many other green turtle nesting sites around the world [4,24,25]. These increases are testimony to the success of conservation efforts. Although the relative numbers of turtles nesting on the different beaches across Redang Island over the last three decades are unknown, the marked increase in nest numbers at the Chagar Hutang Turtle Sanctuary strongly suggests a marked increase in the population. We found clear evidence that accompanying the increase in nest numbers there has been a lengthening of the interval between breeding seasons. This is the first time a systematic long-term change in the remigration interval has been observed in sea turtles, which may reflect the challenges of recording this parameter. Our findings show that the lengthening of the interval between breeding seasons recently reported in cetaceans [13] is not an isolated occurrence among marine megafauna.

Accurately assessing the breeding interval of marine megafauna, including sea turtles, is not straightforward. For cetaceans and seabirds, observations of breeding periodicity have been made by marking individuals (e.g. with tags) or by photo-id (cetaceans) or by reporting the proportion of individuals that breed each year (cetaceans) [13,26]. Across these groups, there are several ways in which artefacts might be introduced to data collected associated with imperfect detection of breeding individuals. For example, it is known that sea turtles may nest on several beaches in the same general area [27]. One suggestion is that individuals have a ‘home’ beach to which they maintain general fidelity and then occasionally they stray to nest on alternative nearby beaches [28]. With this scenario, then even if a single beach is patrolled efficiently and nesting turtles are routinely intercepted and identified there, in some years tagged turtles will nest unobserved on nearby beaches. This scenario probably occurs at the Chagar Hutang Turtle Sanctuary because beach patrol efficiency of the focal beach is high, but there are many alternate nesting beaches nearby. For these reasons, we chose to use the relative abundance of 3- versus 4-year remigration intervals as an index of the mean remigration interval for the population as a whole, because a shift from 3 to 4 years would not be caused by a turtle being missed nesting in 1 year. This method we developed probably has good utility across sea turtles where biases introduced by imperfect detection of nesting individuals are probably universal [29].

Assessing the interval between breeding years is not straightforward across multiple taxa, including birds, marine mammals and turtles. Owing to the difficulties of interpreting what are real remigration intervals and what are artefacts caused by turtles nesting unobserved, there has been debate over whether very long remigration intervals, for example, sometimes 10 years or more, are artefacts or real [30]. However, it is likely that the modal remigration interval is generally recorded accurately in mark-recapture studies. For example, generally long remigration intervals (modes of 3 and 4 years) have been reported for green turtles at Ascension Island, whereas shorter remigration intervals (mode 2 years) have been reported for green turtles nesting in Costa Rica [21]. This variation in remigration intervals across populations probably reflects different trajectories of energy use and acquisition and might be linked to migration distance. Where energy acquisition is faster, turtles probably attain a threshold body condition to breed sooner, and so their remigration interval is shorter [20,21,31,32].

As a corollary to this explanation for differences in the modal remigration interval between populations, the same considerations apply to individuals within a population. For example, we found that some individuals nesting in Redang consistently had shorter remigration intervals than others, and often there was a 1-year lengthening or shortening of an individual's remigration interval. Likewise, such 1-year shifts in remigration interval have been shown elsewhere [33]. These shifts probably reflect changes in the foraging conditions experienced by individuals. It is known that after a nesting season, green turtles can migrate to very dispersed foraging grounds, with each individual tending to have fidelity to a particular foraging ground throughout its adult life [34]. So, we might expect individuals migrating a shorter distance and to a more favourable foraging ground would have a shorter remigration interval than those travelling to more distant and/or less favourable foraging grounds. Recording the migration distance of individuals, or populations, with known remigration intervals can be used to test this prediction [21]. At the same time, conditions on an individual's foraging ground might vary between years. For example, green turtles often feed on seagrass, and the abundance of seagrass might be adversely affected by weather conditions such as storms [34] that might drive a lengthening of the remigration interval.

In common with many other studies, we found that individuals only rarely had a remigration interval of 1 year. Non-annual breeding is the norm for female sea turtles, reflecting the invariant costs of breeding such as the time spent in the nesting area and the energetic costs of egg production and nest excavation [18]. This non-annual breeding in sea turtles is shared by some other groups, including some seabirds and cetaceans, where again the reproductive investment in offspring is high [13,26]. These other taxa are therefore also other likely candidates for where density dependence and/or climate change impacts on foraging success might impact breeding periodicity.

A key finding of our study was evidence for a lengthening of the remigration interval. The most parsimonious explanation for this finding is that it reflects a deterioration of foraging conditions, so that individuals have a slower rate of increase in body condition between breeding seasons. A similar explanation has been proposed to explain reduced breeding reported in cetaceans [13]. The specific reasons for a deterioration of foraging conditions in both turtles and cetaceans are unknown. One possibility is that general impacts of climate change, such as rising sea surface temperature, are affecting food availability. For example, green turtles feed on seagrass, and the abundance of seagrass has been negatively affected by climate change at some sites [35]. Lagged relationships between climate indices and nesting activity reported previously in this region further support the idea that remigration interval variation reflects environmentally mediated changes in energy accumulation rates rather than immediate nesting-site conditions [36]. Another possibility is that increasing numbers in recovering populations might be sufficient to produce density-dependent reductions in foraging success. For green turtles on foraging grounds, increased turtle densities have co-occurred with a decline in seagrass coverage and a decrease in the body condition and growth rate of individuals [37,38]. In a changing climate, large marine vertebrates are subject to many threats, of which some may be mitigated [39–41]. Disentangling the relative roles of density dependence versus climate-induced declines on forage availability could be tested by examining changes in remigration interval for breeding sites around the world and seeing whether lengthening of this interval is linked to the rate of increase in nesting numbers.

The population age structure through time might potentially affect the remigration intervals observed, for example, if young and older turtles return with differing intervals. Reproductive senescence has been observed in many taxa, typically showing that aging individuals have reduced reproductive output. However, contrary to other large herbivores [42], green turtles show no evidence of reproductive senescence [19], a conclusion in accord with our findings that showed no systematic change in the remigration intervals of individuals over time. Hence, lengthening breeding intervals in recent years is unlikely to be owing to reproductive senescence of a few older individuals in the population.

The lengthening of the interval between breeding seasons that we report may have consequences for population recoveries. All else being equal, a longer interval between breeding years will decrease the mean annual reproductive output of individuals and so act to reduce the rate of population size increases. However, where there are marked increases in nesting numbers, as we reported, this may outweigh a reduced per capita reproductive output. Understanding the reasons for a changing breeding periodicity and the interaction between a changing breeding periodicity and population recoveries are important goals both for sea turtles and other marine megafauna, such as cetaceans. Our study emphasizes the importance of long-term studies to understand population trajectories in recovering populations across marine megafauna.

## Supplementary Material



## Data Availability

Data are accessible as electronic supplementary material [43].
