# Supplementary material for: Changes in breeding periodicity of a long-lived vertebrate over 30 years
Source: R Soc Open Sci. 2026 Sep 9;13(9):rsos260642. doi: 10.1098/rsos.260642 (PMC13561040; doi:10.1098/rsos.260642)
Supplement: Supplementary file 1 [file rsos.260642.zip › rsos.260642/rsos260642_si_001.pdf]

Title: Changes in breeding periodicity of a long-lived vertebrate over 30-years

Jared J. Tromp*, Mohd Uzair Rusli, David T. Booth and Graeme C. Hays
*Corresponding author E-mail: j.tromp@deakin.edu.au

Supplementary table 1. Green sea turtle nest counts monitored at Chagar Hutang Turtle Sanctuary (5.81 N, 103.01_E) on Redang Island, Malaysia by nightly foot patrols during the nesting season (April to October).

TurtleID	Date
1993G001	3/6/1993
1993G001	4/6/1993
1993G001	13/6/1993
1993G002	3/6/1993
1993G002	15/6/1993
1993G002	28/6/1993
1993G003	3/6/1993
1993G004	3/6/1993
1993G004	12/6/1993
1993G004	21/6/1993
1993G004	30/6/1993
1993G005	3/6/1993
1993G005	15/6/1993
1993G005	25/7/1993
1993G006	26/7/1993
1993G006	17/8/1993
1993G007	3/6/1993
1993G007	15/6/1993
1993G008	5/6/1993
1993G008	23/6/1993
1993G008	11/7/1993
1993G009	5/6/1993
1993G009	15/6/1993
1993G010	5/6/1993
1993G010	14/6/1993
1993G010	24/6/1993
1993G010	5/7/1993
1993G011	5/6/1993
1993G012	5/6/1993
1993G012	14/6/1993
1993G012	23/6/1993
1993G012	1/7/1993
1993G012	10/7/1993
1993G013	5/6/1993
1993G014	5/6/1993
1993G014	14/6/1993
1993G014	23/6/1993

1993G015	5/6/1993
1993G015	30/6/1993
1993G015	10/7/1993
1993G015	21/7/1993
1993G015	2/8/1993
1993G015	13/8/1993
1993G016	6/6/1993
1993G017	6/6/1993
1993G017	17/6/1993
1993G017	28/6/1993
1993G017	8/7/1993
1993G017	19/7/1993
1993G017	29/7/1993
1993G017	8/8/1993
1993G017	2/9/1993
1993G018	6/6/1993
1993G018	13/6/1993
1993G018	17/6/1993
1993G019	6/6/1993
1993G020	6/6/1993
1993G021	7/6/1993
1993G022	8/6/1993
1993G022	23/7/1993
1993G023	7/6/1993
1993G023	18/6/1993
1993G023	28/6/1993
1993G023	8/7/1993
1993G023	18/7/1993
1993G023	27/7/1993
1993G023	6/8/1993
1993G023	16/8/1993
1993G024	7/6/1993
1993G024	29/6/1993
1993G024	9/7/1993
1993G024	20/7/1993
1993G024	30/7/1993
1993G024	11/8/1993
1993G025	8/6/1993
1993G025	19/6/1993
1993G025	30/6/1993
1993G026	9/6/1993
1993G026	19/6/1993
1993G026	8/7/1993
1993G026	16/7/1993
1993G026	15/8/1993
1993G027	8/6/1993

1993G027	28/6/1993
1993G027	8/7/1993
1993G027	17/7/1993
1993G027	19/7/1993
1993G027	28/7/1993
1993G027	7/8/1993
1993G027	17/8/1993
1993G028	10/6/1993
1993G028	20/6/1993
1993G028	1/7/1993
1993G029	10/6/1993
1993G030	10/6/1993
1993G030	20/6/1993
1993G031	10/6/1993
1993G031	20/6/1993
1993G031	30/6/1993
1993G031	11/7/1993
1993G031	22/7/1993
1993G031	2/8/1993
1993G032	10/6/1993
1993G032	20/6/1993
1993G033	10/6/1993
1993G033	22/6/1993
1993G033	2/7/1993
1993G033	11/7/1993
1993G033	22/7/1993
1993G033	1/8/1993
1993G033	12/8/1993
1993G034	11/6/1993
1993G034	2/7/1993
1993G034	24/7/1993
1993G035	10/6/1993
1993G035	28/6/1993
1993G036	22/6/1993
1993G036	3/7/1993
1993G036	15/7/1993
1993G036	15/8/1993
1993G037	12/6/1993
1993G037	22/6/1993
1993G037	3/7/1993
1993G038	11/6/1993
1993G038	21/6/1993
1993G038	1/7/1993
1993G039	22/6/1993
1993G039	22/6/1993
1993G039	1/7/1993

1993G039	2/7/1993
1993G039	12/7/1993
1993G039	23/7/1993
1993G040	13/6/1993
1993G040	23/6/1993
1993G040	3/7/1993
1993G040	12/7/1993
1993G041	23/6/1993
1993G041	4/7/1993
1993G041	13/7/1993
1993G041	23/7/1993
1993G041	3/8/1993
1993G042	6/7/1993
1993G043	12/6/1993
1993G043	23/6/1993
1993G043	4/7/1993
1993G043	6/7/1993
1993G043	16/7/1993
1993G043	27/7/1993
1993G044	12/6/1993
1993G044	23/6/1993
1993G045	13/6/1993
1993G045	22/6/1993
1993G045	24/6/1993
1993G046	13/6/1993
1993G046	30/6/1993
1993G046	1/7/1993
1993G046	19/7/1993
1993G046	29/7/1993
1993G047	23/6/1993
1993G047	4/7/1993
1993G047	16/7/1993
1993G047	27/7/1993
1993G048	4/6/1993
1993G048	14/6/1993
1993G048	24/6/1993
1993G048	6/7/1993
1993G049	3/6/1993
1993G049	25/6/1993
1993G049	6/7/1993
1993G050	7/6/1993
1993G050	17/6/1993
1993G050	26/6/1993
1993G050	6/7/1993
1993G050	4/8/1993
1993G050	13/8/1993

1993G050	14/8/1993
1993G050	15/8/1993
1993G050	25/8/1993
1993G051	25/7/1993
1993G052	1/8/1993
1993G053	9/8/1993
1993G053	21/8/1993
1993G053	31/8/1993
1993G053	12/9/1993
1993G054	24/7/1993
1993G054	14/8/1993
1993G054	24/8/1993
1993G054	3/9/1993
1993G055	25/7/1993
1993G056	4/8/1993
1993G057	23/7/1993
1993G057	3/8/1993
1993G057	12/8/1993
1993G057	22/8/1993
1993G058	13/6/1993
1993G058	25/6/1993
1993G058	6/7/1993
1993G058	16/7/1993
1993G058	6/8/1993
1993G058	17/8/1993
1993G059	7/8/1993
1993G059	16/9/1993
1993G060	8/8/1993
1993G060	19/9/1993
1993G060	29/9/1993
1993G061	2/9/1993
1993G062	28/9/1993
1993G063	31/8/1993
1993G063	10/9/1993
1993G063	20/9/1993
1993G064	15/9/1993
1993G065	11/6/1993
1993G066	15/6/1993
1993G066	25/6/1993
1993G066	5/7/1993
1993G067	14/6/1993
1993G067	24/6/1993
1993G067	24/6/1993
1993G067	6/7/1993
1993G068	16/6/1993
1993G068	27/6/1993

1993G069	6/7/1993
1993G069	17/7/1993
1993G070	16/6/1993
1993G070	27/6/1993
1993G070	6/7/1993
1993G070	16/7/1993
1993G070	25/7/1993
1993G070	4/8/1993
1993G070	13/8/1993
1993G070	24/8/1993
1993G071	4/6/1993
1993G071	24/6/1993
1993G071	4/7/1993
1993G071	23/7/1993
1993G071	3/8/1993
1993G071	13/8/1993
1993G071	2/9/1993
1993G072	15/6/1993
1993G072	26/6/1993
1993G072	7/7/1993
1993G072	17/7/1993
1993G072	28/7/1993
1993G072	7/8/1993
1993G072	16/8/1993
1993G072	28/8/1993
1993G073	28/6/1993
1993G073	8/7/1993
1993G073	19/7/1993
1993G073	28/7/1993
1993G073	6/8/1993
1993G073	25/8/1993
1993G073	3/9/1993
1993G074	27/6/1993
1993G074	8/7/1993
1993G074	20/7/1993
1993G074	30/7/1993
1993G074	9/8/1993
1993G074	16/8/1993
1993G074	30/8/1993
1993G075	8/7/1993
1993G076	7/6/1993
1993G076	18/6/1993
1993G076	28/6/1993
1993G076	8/7/1993
1993G076	19/7/1993
1993G077	8/7/1993

1993G077	19/7/1993
1993G077	29/7/1993
1993G077	8/8/1993
1993G078	8/7/1993
1993G078	7/8/1993
1993G078	17/8/1993
1993G078	27/8/1993
1993G078	7/9/1993
1993G079	10/6/1993
1993G079	21/6/1993
1993G079	30/6/1993
1993G079	10/7/1993
1993G079	20/7/1993
1993G079	21/7/1993
1993G079	31/7/1993
1993G080	20/6/1993
1993G080	30/6/1993
1993G080	10/7/1993
1993G080	20/7/1993
1993G080	10/8/1993
1993G081	1/7/1993
1993G081	11/7/1993
1993G081	21/7/1993
1993G081	30/7/1993
1993G081	10/8/1993
1993G081	30/8/1993
1993G081	9/9/1993
1993G082	22/6/1993
1993G082	2/7/1993
1993G082	12/7/1993
1993G082	22/7/1993
1993G082	23/7/1993
1993G082	2/8/1993
1993G082	12/8/1993
1993G082	22/8/1993
1993G083	21/6/1993
1993G083	1/7/1993
1993G083	11/7/1993
1993G083	23/7/1993
1993G083	3/8/1993
1993G083	15/8/1993
1993G084	11/6/1993
1993G084	22/6/1993
1993G084	2/7/1993
1993G084	12/7/1993
1993G085	6/7/1993

1993G085	16/7/1993
1993G086	6/7/1993
1993G086	26/7/1993
1993G086	5/8/1993
1993G086	14/8/1993
1993G086	25/8/1993
1993G087	6/7/1993
1993G087	7/7/1993
1993G087	8/7/1993
1993G087	18/7/1993
1993G087	28/7/1993
1993G087	18/8/1993
1993G087	28/8/1993
1993G087	7/9/1993
1993G088	16/6/1993
1993G088	7/7/1993
1993G088	17/7/1993
1993G088	27/7/1993
1993G088	7/8/1993
1993G089	15/6/1993
1993G089	27/6/1993
1993G089	7/7/1993
1993G089	18/7/1993
1993G089	29/7/1993
1993G089	9/8/1993
1993G090	23/7/1993
1993G090	5/8/1993
1993G090	29/8/1993
1993G091	17/6/1993
1993G091	7/7/1993
1993G091	17/7/1993
1993G091	29/7/1993
1993G091	12/8/1993
1993G092	14/6/1993
1993G092	25/6/1993
1993G092	5/7/1993
1993G092	15/7/1993
1993G092	24/7/1993
1993G092	3/8/1993
1993G093	4/7/1993
1993G093	15/7/1993
1993G093	5/8/1993
1993G093	16/8/1993
1993G093	27/8/1993
1993G094	28/6/1993
1993G094	8/7/1993

1993G094	18/7/1993
1993G094	17/8/1993
1993G094	25/8/1993
1993G095	21/7/1993
1993G095	1/8/1993
1993G095	11/8/1993
1993G095	21/8/1993
1993G095	31/8/1993
1993G095	9/9/1993
1993G095	20/9/1993
1993G096	5/6/1993
1993G096	18/6/1993
1993G096	28/6/1993
1993G096	8/7/1993
1993G096	18/7/1993
1993G096	30/7/1993
1993G097	22/6/1993
1993G097	3/7/1993
1993G097	19/7/1993
1993G097	11/8/1993
1993G097	22/8/1993
1993G098	16/6/1993
1993G098	25/6/1993
1993G098	5/7/1993
1993G098	16/7/1993
1993G098	5/8/1993
1993G099	22/7/1993
1993G099	1/8/1993
1993G099	11/8/1993
1993G099	20/8/1993
1993G099	18/9/1993
1993G100	19/6/1993
1993G100	9/7/1993
1993G100	19/7/1993
1993G100	29/7/1993
1993G100	19/8/1993
1993G100	28/8/1993
1993G100	8/9/1993
1993G101	23/7/1993
1993G101	4/8/1993
1993G101	22/8/1993
1993G102	20/7/1993
1993G102	31/7/1993
1993G102	12/8/1993
1993G102	22/8/1993
1993G102	3/9/1993

1993G102	13/9/1993
1993G102	22/9/1993
1993G102	24/9/1993
1993G103	20/6/1993
1993G103	30/6/1993
1993G103	9/7/1993
1993G103	20/7/1993
1993G103	19/8/1993
1993G103	30/8/1993
1993G103	9/9/1993
1993G104	10/7/1993
1993G104	20/7/1993
1993G104	30/7/1993
1993G104	9/8/1993
1993G104	20/8/1993
1993G104	2/9/1993
1993G105	23/6/1993
1993G105	3/7/1993
1993G105	16/7/1993
1993G105	26/7/1993
1993G105	6/8/1993
1993G105	19/8/1993
1993G105	27/8/1993
1993G105	6/9/1993
1993G106	16/7/1993
1993G106	5/8/1993
1993G106	15/8/1993
1993G106	25/8/1993
1993G106	4/9/1993
1993G106	14/9/1993
1993G107	21/6/1993
1993G107	30/6/1993
1993G107	10/7/1993
1993G107	20/7/1993
1993G107	30/7/1993
1993G107	9/8/1993
1993G107	2/9/1993
1993G108	12/7/1993
1993G108	23/7/1993
1993G108	2/8/1993
1993G108	11/8/1993
1993G108	21/8/1993
1993G108	18/9/1993
1993G108	28/9/1993
1993G109	13/7/1993
1993G109	23/7/1993

1993G109	3/8/1993
1993G109	13/8/1993
1993G109	24/8/1993
1993G109	2/9/1993
1993G109	13/9/1993
1993G110	20/7/1993
1993G110	30/7/1993
1993G110	10/8/1993
1993G110	21/8/1993
1993G110	29/8/1993
1993G110	7/9/1993
1993G110	16/9/1993
1993G111	13/7/1993
1993G111	23/7/1993
1993G111	3/8/1993
1993G111	14/8/1993
1993G111	4/9/1993
1993G111	14/9/1993
1993G112	20/7/1993
1993G112	30/7/1993
1993G112	9/8/1993
1993G113	24/6/1993
1993G113	4/7/1993
1993G113	14/7/1993
1993G113	23/7/1993
1993G113	24/7/1993
1993G113	2/8/1993
1993G113	12/8/1993
1993G114	15/7/1993
1993G114	25/7/1993
1993G114	5/8/1993
1993G114	15/8/1993
1993G114	25/8/1993
1993G114	5/9/1993
1993G115	24/6/1993
1993G115	5/7/1993
1993G115	15/7/1993
1993G115	23/7/1993
1993G115	2/8/1993
1993G115	11/8/1993
1993G116	3/6/1993
1993G116	13/6/1993
1993G116	23/6/1993
1993G116	4/7/1993
1993G116	14/7/1993
1993G116	16/7/1993

1993G116	27/7/1993
1993G116	7/8/1993
1993G117	5/6/1993
1993G117	5/6/1993
1993G117	15/6/1993
1993G117	26/6/1993
1993G117	5/7/1993
1993G117	16/7/1993
1993G117	26/7/1993
1993G118	6/6/1993
1993G118	25/6/1993
1993G118	6/7/1993
1993G118	7/7/1993
1993G118	17/7/1993
1993G119	17/7/1993
1993G119	7/8/1993
1993G119	26/8/1993
1993G119	5/9/1993
1993G120	1/8/1993
1993G120	11/8/1993
1993G121	31/7/1993
1993G121	11/8/1993
1993G121	22/8/1993
1993G122	11/8/1993
1993G122	2/9/1993
1993G123	22/8/1993
1993G123	10/9/1993
1993G123	20/9/1993
1993G123	29/9/1993
1993G123	29/9/1993
1993G124	3/7/1993
1993G124	16/8/1993
1993G125	6/7/1993
1993G125	16/7/1993
1993G125	26/7/1993
1993G125	15/8/1993
1993G125	26/8/1993
1993G125	5/9/1993
1993G125	15/9/1993
1993G126	6/6/1993
1993G126	15/6/1993
1993G126	24/6/1993
1993G126	3/7/1993
1993G126	12/7/1993
1993G126	22/7/1993
1993G126	3/8/1993

1993G127	4/8/1993
1993G127	22/8/1993
1993G127	2/9/1993
1993G127	11/9/1993
1993G127	22/9/1993
1993G128	26/8/1993
1993G129	14/8/1993
1993G129	24/8/1993
1993G129	2/9/1993
1993G129	11/9/1993
1993G129	29/9/1993
1993G130	17/8/1993
1993G130	26/8/1993
1993G130	6/9/1993
1993G130	17/9/1993
1993G130	28/9/1993
1993G131	17/8/1993
1993G131	26/8/1993
1993G131	19/9/1993
1993G131	29/9/1993
1993G132	10/8/1993
1993G132	2/9/1993
1993G132	15/9/1993
1993G133	4/9/1993
1993G134	13/6/1993
1993G135	6/6/1993
1993G135	16/6/1993
1993G135	7/7/1993
1993G135	18/7/1993
1993G135	8/8/1993
1993G136	4/6/1993
1993G136	15/6/1993
1993G136	25/6/1993
1993G136	5/7/1993
1993G136	16/7/1993
1993G137	6/7/1993
1993G138	20/6/1993
1993G138	21/6/1993
1993G138	17/7/1993
1993G138	13/8/1993
1993G139	17/7/1993
1993G140	18/7/1993
1994G001	18/5/1994
1994G001	28/5/1994
1994G001	29/5/1994
1994G001	9/6/1994

1994G001	18/6/1994
1994G001	28/6/1994
1994G001	9/7/1994
1994G002	18/5/1994
1994G002	30/5/1994
1994G002	9/6/1994
1994G002	10/6/1994
1994G002	22/6/1994
1994G002	2/7/1994
1994G002	14/7/1994
1994G002	26/7/1994
1994G003	31/5/1994
1994G003	9/6/1994
1994G003	18/6/1994
1994G003	27/6/1994
1994G003	6/7/1994
1994G004	24/6/1994
1994G004	5/7/1994
1994G004	15/7/1994
1994G004	26/7/1994
1994G004	5/8/1994
1994G004	15/8/1994
1994G004	25/8/1994
1994G004	26/8/1994
1994G004	8/9/1994
1994G005	23/5/1994
1994G005	2/6/1994
1994G005	14/6/1994
1994G006	28/5/1994
1994G006	8/6/1994
1994G006	18/6/1994
1994G006	28/6/1994
1994G006	8/7/1994
1994G006	18/7/1994
1994G006	28/7/1994
1994G006	8/8/1994
1994G007	18/5/1994
1994G007	30/5/1994
1994G007	10/6/1994
1994G007	22/6/1994
1994G007	2/7/1994
1994G007	12/7/1994
1994G008	25/5/1994
1994G009	19/5/1994
1994G009	29/5/1994
1994G009	8/6/1994

1994G009	29/6/1994
1994G010	16/5/1994
1994G010	26/5/1994
1994G010	6/6/1994
1994G010	16/6/1994
1994G010	26/6/1994
1994G010	7/7/1994
1994G010	18/7/1994
1994G011	16/5/1994
1994G011	26/5/1994
1994G011	6/6/1994
1994G011	16/6/1994
1994G011	26/6/1994
1994G012	16/5/1994
1994G012	26/5/1994
1994G013	16/5/1994
1994G013	26/5/1994
1994G013	4/6/1994
1994G013	13/6/1994
1994G013	22/6/1994
1994G013	2/7/1994
1994G013	11/7/1994
1994G014	17/5/1994
1994G014	26/5/1994
1994G015	16/5/1994
1994G016	17/5/1994
1994G016	26/5/1994
1994G016	5/6/1994
1994G016	15/6/1994
1994G016	25/6/1994
1994G016	6/7/1994
1994G017	17/5/1994
1994G017	26/5/1994
1994G017	4/6/1994
1994G017	14/6/1994
1994G018	17/5/1994
1994G018	11/6/1994
1994G018	22/6/1994
1994G018	4/7/1994
1994G018	16/7/1994
1994G019	27/5/1994
1994G019	2/6/1994
1994G019	16/6/1994
1994G020	31/5/1994
1994G020	10/6/1994
1994G021	7/6/1994

1994G021	17/6/1994
1994G021	27/6/1994
1994G021	28/6/1994
1994G021	29/6/1994
1994G021	9/7/1994
1994G021	21/7/1994
1994G022	22/6/1994
1994G022	6/7/1994
1994G022	17/7/1994
1994G022	27/7/1994
1994G022	7/8/1994
1994G022	17/8/1994
1994G022	27/8/1994
1994G022	9/9/1994
1994G023	4/6/1994
1994G023	13/6/1994
1994G023	23/6/1994
1994G023	3/7/1994
1994G023	12/7/1994
1994G023	22/7/1994
1994G023	24/7/1994
1994G023	3/8/1994
1994G024	4/6/1994
1994G024	14/6/1994
1994G024	27/6/1994
1994G024	7/7/1994
1994G024	18/7/1994
1994G024	29/7/1994
1994G024	9/8/1994
1994G025	1/6/1994
1994G025	12/6/1994
1994G025	22/6/1994
1994G025	2/7/1994
1994G025	11/7/1994
1994G026	19/7/1994
1994G026	29/7/1994
1994G026	9/8/1994
1994G026	18/8/1994
1994G026	27/8/1994
1994G026	6/9/1994
1994G026	16/9/1994
1994G026	27/9/1994
1994G027	6/6/1994
1994G027	15/6/1994
1994G027	26/6/1994
1994G027	6/7/1994

1994G028	8/6/1994
1994G028	19/6/1994
1994G028	28/6/1994
1994G028	8/7/1994
1994G028	18/7/1994
1994G028	28/7/1994
1994G028	6/8/1994
1994G028	27/8/1994
1994G029	7/7/1994
1994G029	18/7/1994
1994G029	30/7/1994
1994G029	9/8/1994
1994G029	20/8/1994
1994G029	21/8/1994
1994G029	31/8/1994
1994G029	11/9/1994
1994G029	22/9/1994
1994G029	23/9/1994
1994G029	4/10/1994
1994G030	29/6/1994
1994G031	14/7/1994
1994G032	5/7/1994
1994G032	16/7/1994
1994G032	27/7/1994
1994G032	7/8/1994
1994G032	18/8/1994
1994G033	30/6/1994
1994G033	11/7/1994
1994G033	21/7/1994
1994G033	31/7/1994
1994G033	10/8/1994
1994G033	21/8/1994
1994G033	2/9/1994
1994G034	26/6/1994
1994G034	8/7/1994
1994G034	21/7/1994
1994G034	1/8/1994
1994G034	12/8/1994
1994G034	26/8/1994
1994G035	7/7/1994
1994G036	11/7/1994
1994G036	23/7/1994
1994G036	4/8/1994
1994G036	17/8/1994
1994G036	30/8/1994
1994G037	20/7/1994

1994G037	1/8/1994
1994G037	11/9/1994
1994G038	11/7/1994
1994G038	23/7/1994
1994G038	3/8/1994
1994G038	14/8/1994
1994G038	25/8/1994
1994G038	5/9/1994
1994G039	20/8/1994
1994G039	31/8/1994
1994G039	11/9/1994
1994G040	18/7/1994
1994G040	23/7/1994
1994G040	29/7/1994
1994G040	10/8/1994
1994G040	21/8/1994
1994G041	21/7/1994
1994G041	30/7/1994
1994G041	9/8/1994
1994G041	19/8/1994
1994G041	28/8/1994
1994G041	29/8/1994
1994G041	8/9/1994
1994G041	18/9/1994
1994G041	27/9/1994
1994G041	6/10/1994
1994G041	16/10/1994
1994G042	18/8/1994
1994G042	27/8/1994
1994G042	6/9/1994
1994G042	16/9/1994
1994G042	26/9/1994
1994G042	7/10/1994
1994G043	19/6/1994
1994G043	28/6/1994
1994G043	8/7/1994
1994G043	18/7/1994
1994G043	28/7/1994
1994G043	7/8/1994
1994G043	17/8/1994
1994G043	27/8/1994
1994G043	6/9/1994
1994G043	20/9/1994
1994G044	10/10/1994
1994G044	20/10/1994
1994G045	5/10/1994

1994G045	15/10/1994
1994G046	6/8/1994
1994G046	17/8/1994
1994G046	29/8/1994
1994G046	10/9/1994
1994G046	22/9/1994
1994G046	5/10/1994
1994G047	31/5/1994
1994G047	13/6/1994
1994G047	24/6/1994
1994G047	5/7/1994
1994G047	18/7/1994
1994G047	29/7/1994
1994G047	12/8/1994
1994G048	30/5/1994
1994G048	9/6/1994
1994G048	18/6/1994
1994G048	28/6/1994
1994G048	8/7/1994
1994G048	19/7/1994
1994G048	30/7/1994
1994G049	31/5/1994
1994G049	10/6/1994
1994G049	11/6/1994
1994G049	21/6/1994
1994G049	30/6/1994
1994G049	10/7/1994
1994G049	19/7/1994
1994G049	21/7/1994
1994G049	30/7/1994
1994G049	31/7/1994
1994G049	10/8/1994
1994G049	21/8/1994
1994G049	22/8/1994
1994G050	23/5/1994
1994G050	2/6/1994
1994G050	12/6/1994
1994G050	22/6/1994
1994G050	11/7/1994
1994G050	23/7/1994
1994G051	17/5/1994
1994G051	18/5/1994
1994G051	27/5/1994
1994G051	6/6/1994
1994G051	16/6/1994
1994G051	27/6/1994

1994G052	27/5/1994
1994G053	23/6/1994
1994G053	23/7/1994
1994G054	26/5/1994
1994G054	8/6/1994
1994G054	27/5/1994
1994G055	30/5/1994
1994G056	22/5/1994
1994G056	31/5/1994
1994G056	9/6/1994
1994G057	22/5/1994
1994G057	2/6/1994
1994G058	21/5/1994
1994G058	31/5/1994
1994G058	9/6/1994
1994G058	19/6/1994
1994G058	29/6/1994
1994G059	17/7/1994
1994G059	30/7/1994
1994G059	12/9/1994
1994G059	24/9/1994
1994G059	6/10/1994
1994G060	20/5/1994
1994G060	30/5/1994
1994G060	31/5/1994
1994G060	10/6/1994
1994G060	20/6/1994
1994G060	30/6/1994
1994G060	1/7/1994
1994G060	12/7/1994
1994G060	13/7/1994
1994G061	30/6/1994
1994G061	12/7/1994
1994G061	25/7/1994
1994G061	16/8/1994
1994G062	29/8/1994
1994G063	26/6/1994
1994G063	6/7/1994
1994G063	17/7/1994
1993G022	8/7/1995
1993G022	28/7/1995
1993G050	26/5/1995
1993G050	5/6/1995
1993G050	6/6/1995
1993G050	16/6/1995
1993G050	25/6/1995

1993G050	4/7/1995
1993G050	13/7/1995
1993G050	22/7/1995
1993G050	31/7/1995
1993G050	10/8/1995
1993G116	14/5/1995
1993G116	26/5/1995
1993G116	16/6/1995
1993G116	26/6/1995
1993G116	6/7/1995
1993G116	15/7/1995
1993G116	25/7/1995
1993G116	4/8/1995
1995G001	9/5/1995
1995G001	21/5/1995
1995G001	1/6/1995
1995G001	12/6/1995
1995G001	22/6/1995
1995G001	3/7/1995
1995G002	7/5/1995
1995G002	20/5/1995
1995G002	30/5/1995
1995G002	27/7/1995
1995G002	9/7/1995
1995G002	30/6/1995
1995G003	7/5/1995
1995G003	19/5/1995
1995G003	31/5/1995
1995G004	7/5/1995
1995G004	18/5/1995
1995G004	28/5/1995
1995G004	8/6/1995
1995G004	18/6/1995
1995G004	28/6/1995
1995G004	12/7/1995
1995G005	13/5/1995
1995G005	23/5/1995
1995G005	2/6/1995
1995G005	11/6/1995
1995G005	20/6/1995
1995G005	28/6/1995
1995G005	7/7/1995
1995G005	16/7/1995
1995G006	2/6/1995
1995G006	12/6/1995
1995G006	22/6/1995

1995G006	1/7/1995
1995G006	11/7/1995
1995G006	20/7/1995
1995G006	29/7/1995
1995G006	8/8/1995
1995G006	17/8/1995
1995G006	29/8/1995
1995G007	8/5/1995
1995G007	19/5/1995
1995G007	30/5/1995
1995G007	12/6/1995
1995G008	9/5/1995
1995G008	20/5/1995
1995G008	2/6/1995
1995G008	12/6/1995
1995G008	22/6/1995
1995G008	4/7/1995
1995G008	16/7/1995
1995G009	12/5/1995
1995G009	22/5/1995
1995G009	2/6/1995
1995G009	13/6/1995
1995G009	22/6/1995
1995G009	3/7/1995
1995G009	14/7/1995
1995G010	9/5/1995
1995G010	19/5/1995
1995G010	30/5/1995
1995G011	12/5/1995
1995G011	21/5/1995
1995G011	31/5/1995
1995G011	13/6/1995
1995G011	14/6/1995
1995G012	10/5/1995
1995G012	31/5/1995
1995G012	10/6/1995
1995G012	21/6/1995
1995G013	12/5/1995
1995G013	21/5/1995
1995G013	30/5/1995
1995G014	12/5/1995
1995G014	23/5/1995
1995G014	2/6/1995
1995G014	12/6/1995
1995G014	22/6/1995
1995G014	2/7/1995

1995G014	13/7/1995
1995G014	23/7/1995
1995G014	2/8/1995
1995G014	14/8/1995
1995G015	12/5/1995
1995G015	14/6/1995
1995G015	23/5/1995
1995G015	3/6/1995
1995G015	8/7/1995
1995G015	25/6/1995
1995G016	14/5/1995
1995G016	2/6/1995
1995G016	13/6/1995
1995G016	25/6/1995
1995G016	24/5/1995
1995G017	1/6/1995
1995G017	11/6/1995
1995G017	19/6/1995
1995G017	27/6/1995
1995G017	17/7/1995
1995G018	16/5/1995
1995G018	26/5/1995
1995G018	4/6/1995
1995G019	16/5/1995
1995G019	28/5/1995
1995G020	20/5/1995
1995G020	30/5/1995
1995G021	16/5/1995
1995G021	26/5/1995
1995G021	6/6/1995
1995G022	18/5/1995
1995G022	30/5/1995
1995G022	10/6/1995
1995G022	20/6/1995
1995G022	30/6/1995
1995G022	12/7/1995
1995G023	15/5/1995
1995G023	24/5/1995
1995G023	2/6/1995
1995G024	13/5/1995
1995G024	23/5/1995
1995G024	2/6/1995
1995G024	11/6/1995
1995G024	21/6/1995
1995G024	2/7/1995
1995G024	13/7/1995

1995G025	13/5/1995
1995G025	24/5/1995
1995G025	3/6/1995
1995G025	15/6/1995
1995G025	25/6/1995
1995G025	26/6/1995
1995G025	7/7/1995
1995G026	14/5/1995
1995G026	23/5/1995
1995G026	2/6/1995
1995G026	13/6/1995
1995G026	22/6/1995
1995G026	1/7/1995
1995G026	11/7/1995
1995G027	22/5/1995
1995G027	1/6/1995
1995G027	11/6/1995
1995G027	21/6/1995
1995G027	1/7/1995
1995G027	11/7/1995
1995G027	22/7/1995
1995G027	10/8/1995
1995G028	13/5/1995
1995G028	23/5/1995
1995G028	24/5/1995
1995G029	15/5/1995
1995G029	27/5/1995
1995G029	29/5/1995
1995G030	14/5/1995
1995G030	24/5/1995
1995G030	13/6/1995
1995G030	23/6/1995
1995G030	3/7/1995
1995G030	15/7/1995
1995G030	28/7/1995
1995G031	29/5/1995
1995G031	11/6/1995
1995G031	4/7/1995
1995G031	14/7/1995
1995G031	26/7/1995
1995G031	6/8/1995
1995G031	17/8/1995
1995G031	29/8/1995
1995G032	11/6/1995
1995G032	21/6/1995
1995G032	30/6/1995

1995G032	10/7/1995
1995G032	19/7/1995
1995G032	29/7/1995
1995G032	7/8/1995
1995G032	17/8/1995
1995G032	28/8/1995
1995G033	13/5/1995
1995G033	22/5/1995
1995G033	24/5/1995
1995G033	2/6/1995
1995G033	12/6/1995
1995G033	21/6/1995
1995G033	2/7/1995
1995G034	21/5/1995
1995G034	2/6/1995
1995G034	13/6/1995
1995G034	23/6/1995
1995G034	3/7/1995
1995G034	14/7/1995
1995G035	24/5/1995
1995G035	4/6/1995
1995G035	16/6/1995
1995G036	19/5/1995
1995G036	29/5/1995
1995G036	7/6/1995
1995G037	30/5/1995
1995G037	9/6/1995
1995G037	19/6/1995
1995G037	28/6/1995
1995G037	8/7/1995
1995G037	16/7/1995
1995G037	25/7/1995
1995G037	4/8/1995
1995G038	24/5/1995
1995G038	4/6/1995
1995G038	14/6/1995
1995G038	24/6/1995
1995G038	4/7/1995
1995G038	14/7/1995
1995G038	2/8/1995
1995G038	23/7/1995
1995G039	31/5/1995
1995G039	9/6/1995
1995G039	18/6/1995
1995G039	27/6/1995
1995G039	6/7/1995

1995G039	15/7/1995
1995G039	24/7/1995
1995G039	11/8/1995
1995G039	21/8/1995
1995G040	24/5/1995
1995G040	2/6/1995
1995G040	11/6/1995
1995G040	30/6/1995
1995G040	11/7/1995
1995G041	27/5/1995
1995G041	7/6/1995
1995G041	18/6/1995
1995G041	27/6/1995
1995G041	28/6/1995
1995G041	8/7/1995
1995G041	18/7/1995
1995G041	29/7/1995
1995G042	26/5/1995
1995G042	16/6/1995
1995G042	6/7/1995
1995G042	17/7/1995
1995G042	28/7/1995
1995G042	8/8/1995
1995G043	11/6/1995
1995G043	13/7/1995
1995G043	1/8/1995
1995G044	29/5/1995
1995G044	9/6/1995
1995G044	19/6/1995
1995G044	29/6/1995
1995G045	30/5/1995
1995G045	10/6/1995
1995G045	29/6/1995
1995G045	9/7/1995
1995G045	20/7/1995
1995G046	2/6/1995
1995G046	12/6/1995
1995G046	21/6/1995
1995G046	1/7/1995
1995G046	11/7/1995
1995G046	20/7/1995
1995G046	30/7/1995
1995G046	8/8/1995
1995G047	2/6/1995
1995G047	4/6/1995
1995G047	15/6/1995

1995G047	25/6/1995
1995G047	5/7/1995
1995G047	15/7/1995
1995G047	25/7/1995
1995G047	4/8/1995
1995G047	15/8/1995
1995G047	27/8/1995
1995G048	16/6/1995
1995G048	26/6/1995
1995G048	5/7/1995
1995G048	14/7/1995
1995G048	23/7/1995
1995G048	1/8/1995
1995G048	11/8/1995
1995G049	3/6/1995
1995G049	14/6/1995
1995G049	25/6/1995
1995G049	6/7/1995
1995G049	16/7/1995
1995G049	29/7/1995
1995G049	8/8/1995
1995G049	20/8/1995
1995G050	25/6/1995
1995G050	4/7/1995
1995G050	6/7/1995
1995G050	16/7/1995
1995G050	25/7/1995
1995G050	4/8/1995
1995G050	14/8/1995
1995G050	25/8/1995
1995G051	4/6/1995
1995G051	15/6/1995
1995G051	4/7/1995
1995G051	24/7/1995
1995G051	2/8/1995
1995G051	12/8/1995
1995G051	21/8/1995
1995G051	31/8/1995
1995G052	11/7/1995
1995G052	21/7/1995
1995G052	31/7/1995
1995G052	10/8/1995
1995G052	1/9/1995
1995G053	5/6/1995
1995G053	16/6/1995
1995G053	25/6/1995

1995G053	4/7/1995
1995G053	13/7/1995
1995G053	22/7/1995
1995G053	1/8/1995
1995G054	13/6/1995
1995G054	25/6/1995
1995G054	6/7/1995
1995G054	16/7/1995
1995G054	26/7/1995
1995G054	6/8/1995
1995G054	17/8/1995
1995G054	28/8/1995
1995G055	13/6/1995
1995G055	22/6/1995
1995G055	4/7/1995
1995G055	13/7/1995
1995G055	23/7/1995
1995G055	11/8/1995
1995G055	22/8/1995
1995G056	18/6/1995
1995G056	1/7/1995
1995G056	13/7/1995
1995G056	23/7/1995
1995G056	1/8/1995
1995G056	12/8/1995
1995G056	23/8/1995
1995G056	3/9/1995
1995G056	14/9/1995
1995G057	13/6/1995
1995G057	25/6/1995
1995G057	6/7/1995
1995G057	15/7/1995
1995G057	25/7/1995
1995G057	4/8/1995
1995G057	15/8/1995
1995G057	27/8/1995
1995G058	21/6/1995
1995G058	2/7/1995
1995G058	13/7/1995
1995G058	24/7/1995
1995G058	13/8/1995
1995G058	22/8/1995
1995G059	24/6/1995
1995G059	5/7/1995
1995G059	16/7/1995
1995G059	25/7/1995

1995G059	16/8/1995
1995G059	25/8/1995
1995G059	5/9/1995
1995G059	16/9/1995
1995G059	26/7/1995
1995G059	7/8/1995
1995G060	3/8/1995
1995G060	15/8/1995
1995G060	25/8/1995
1995G060	7/9/1995
1995G060	8/9/1995
1995G061	25/6/1995
1995G061	5/7/1995
1995G061	15/7/1995
1995G061	2/8/1995
1995G061	12/8/1995
1995G061	21/8/1995
1995G062	1/7/1995
1995G062	14/7/1995
1995G062	25/7/1995
1995G062	26/8/1995
1995G063	26/6/1995
1995G063	7/7/1995
1995G063	17/7/1995
1995G063	26/7/1995
1995G063	6/8/1995
1995G063	18/8/1995
1995G064	28/6/1995
1995G064	19/7/1995
1995G064	9/8/1995
1995G064	10/9/1995
1995G064	9/7/1995
1995G064	19/8/1995
1995G064	29/7/1995
1995G065	29/6/1995
1995G065	9/7/1995
1995G065	20/7/1995
1995G065	29/7/1995
1995G065	1/9/1995
1995G066	22/6/1995
1995G066	23/6/1995
1995G066	4/7/1995
1995G066	14/7/1995
1995G066	25/7/1995
1995G066	4/8/1995
1995G066	14/8/1995

1995G066	4/9/1995
1995G067	9/7/1995
1995G067	30/7/1995
1995G067	21/8/1995
1995G067	1/9/1995
1995G068	13/7/1995
1995G068	23/7/1995
1995G068	1/8/1995
1995G068	11/8/1995
1995G068	21/8/1995
1995G069	6/7/1995
1995G069	28/7/1995
1995G069	8/8/1995
1995G069	19/8/1995
1995G069	29/8/1995
1995G069	10/9/1995
1995G069	21/9/1995
1995G070	3/7/1995
1995G070	14/7/1995
1995G070	25/7/1995
1995G070	4/8/1995
1995G070	15/8/1995
1995G070	27/8/1995
1995G070	8/9/1995
1995G070	19/9/1995
1995G071	7/8/1995
1995G071	18/8/1995
1995G071	2/10/1995
1995G072	18/8/1995
1995G072	29/8/1995
1995G072	31/8/1995
1995G072	22/9/1995
1995G073	11/8/1995
1995G073	13/9/1995
1995G074	22/8/1995
1995G074	24/9/1995
1995G075	22/8/1995
1995G075	2/9/1995
1995G076	6/8/1995
1995G076	16/8/1995
1995G076	26/8/1995
1995G076	5/9/1995
1995G076	15/9/1995
1995G077	24/8/1995
1995G077	3/9/1995
1995G077	14/9/1995

1995G078	22/7/1995
1995G078	2/8/1995
1995G078	13/8/1995
1995G079	29/7/1995
1995G079	9/8/1995
1995G079	21/8/1995
1995G079	1/9/1995
1995G080	25/7/1995
1995G080	4/8/1995
1995G080	14/8/1995
1995G080	2/9/1995
1995G080	23/9/1995
1995G080	3/10/1995
1995G080	15/10/1995
1995G081	19/7/1995
1995G081	29/7/1995
1995G081	9/8/1995
1995G081	29/8/1995
1995G082	21/7/1995
1995G082	31/7/1995
1995G082	12/8/1995
1995G083	18/7/1995
1995G083	30/7/1995
1995G084	25/7/1995
1995G084	4/8/1995
1995G084	14/8/1995
1995G084	23/8/1995
1995G084	13/9/1995
1995G084	24/9/1995
1995G085	21/7/1995
1995G085	31/7/1995
1995G085	11/8/1995
1995G085	29/8/1995
1995G085	9/9/1995
1995G086	21/7/1995
1995G086	12/8/1995
1995G086	23/8/1995
1995G086	14/9/1995
1995G086	15/9/1995
1995G087	4/7/1995
1995G087	14/7/1995
1995G087	3/8/1995
1995G087	14/8/1995
1995G087	15/9/1995
1995G087	29/9/1995
1995G088	1/8/1995

1995G088	12/8/1995
1995G088	4/9/1995
1995G089	8/8/1995
1995G089	18/8/1995
1995G089	29/8/1995
1995G089	8/9/1995
1995G089	19/9/1995
1995G090	4/8/1995
1995G090	14/8/1995
1995G090	15/8/1995
1995G090	25/8/1995
1995G090	5/9/1995
1995G091	27/8/1995
1995G091	8/9/1995
1995G092	14/9/1995
1995G092	3/10/1995
1995G093	7/5/1995
1995G093	17/5/1995
1995G093	27/5/1995
1995G093	28/5/1995
1995G093	6/6/1995
1995G093	15/6/1995
1995G093	25/6/1995
1995G093	4/7/1995
1995G093	14/7/1995
1995G094	1/9/1995
1995G094	6/10/1995
1995G095	25/8/1995
1995G095	5/9/1995
1995G095	15/9/1995
1995G095	24/9/1995
1995G096	14/7/1995
1995G096	23/7/1995
1995G096	1/8/1995
1995G096	11/8/1995
1995G096	20/8/1995
1995G096	8/9/1995
1995G096	18/9/1995
1995G097	15/9/1995
1995G098	18/8/1995
1995G098	30/8/1995
1995G098	3/10/1995
1995G098	12/10/1995
1995G099	22/9/1995
1995G099	3/10/1995
1995G100	24/9/1995

1995G101	14/5/1995
1995G101	24/5/1995
1995G102	4/7/1995
1995G103	17/6/1995
1995G103	26/6/1995
1995G103	5/7/1995
1995G103	15/7/1995
1995G103	12/8/1995
1995G104	22/5/1995
1995G104	11/6/1995
1995G104	22/6/1995
1995G104	1/7/1995
1995G104	12/7/1995
1995G105	9/7/1995
1995G105	18/7/1995
1995G105	29/7/1995
1995G105	8/8/1995
1995G106	2/7/1995
1995G107	16/8/1995
1995G108	3/7/1995
1995G108	12/7/1995
1995G108	21/7/1995
1995G108	30/7/1995
1995G108	8/8/1995
1995G109	16/6/1995
1995G109	26/6/1995
1995G109	6/7/1995
1995G110	16/7/1995
1995G110	28/7/1995
1995G110	8/8/1995
1993G001	10/5/1996
1993G001	19/5/1996
1993G001	29/5/1996
1993G001	7/6/1996
1993G001	15/6/1996
1993G001	23/6/1996
1993G001	2/7/1996
1993G009	14/5/1996
1993G009	24/5/1996
1993G009	3/6/1996
1993G009	14/6/1996
1993G009	25/6/1996
1993G009	5/7/1996
1993G012	22/7/1996
1993G012	3/8/1996
1993G012	15/8/1996

1993G012	24/8/1996
1993G012	2/9/1996
1993G012	11/9/1996
1993G012	22/9/1996
1993G015	3/6/1996
1993G015	14/6/1996
1993G015	24/6/1996
1993G015	5/7/1996
1993G015	25/7/1996
1993G015	5/8/1996
1993G021	7/6/1996
1993G021	29/6/1996
1993G023	6/6/1996
1993G023	17/6/1996
1993G023	27/6/1996
1993G023	7/7/1996
1993G023	17/7/1996
1993G023	27/7/1996
1993G023	5/8/1996
1993G023	15/8/1996
1993G023	27/8/1996
1993G026	3/6/1996
1993G026	22/6/1996
1993G026	2/7/1996
1993G026	11/7/1996
1993G026	20/7/1996
1993G026	28/7/1996
1993G026	6/8/1996
1993G026	15/8/1996
1993G026	26/8/1996
1993G028	11/5/1996
1993G028	20/5/1996
1993G028	30/5/1996
1993G028	8/6/1996
1993G028	17/6/1996
1993G028	27/6/1996
1993G028	7/7/1996
1993G028	17/7/1996
1993G030	11/6/1996
1993G030	23/6/1996
1993G030	4/7/1996
1993G030	5/8/1996
1993G037	15/5/1996
1993G037	7/6/1996
1993G037	17/6/1996
1993G037	27/6/1996

1993G037	8/7/1996
1993G037	19/7/1996
1993G043	12/6/1996
1993G043	23/6/1996
1993G043	4/7/1996
1993G043	5/7/1996
1993G043	15/7/1996
1993G043	24/7/1996
1993G043	25/7/1996
1993G043	4/8/1996
1993G043	15/8/1996
1993G046	8/5/1996
1993G046	17/5/1996
1993G046	27/5/1996
1993G046	5/6/1996
1993G046	15/6/1996
1993G046	24/6/1996
1993G046	25/6/1996
1993G046	4/7/1996
1993G048	11/5/1996
1993G048	21/5/1996
1993G048	1/6/1996
1993G048	13/6/1996
1993G072	18/7/1996
1993G072	29/7/1996
1993G072	8/8/1996
1993G072	18/8/1996
1993G072	29/8/1996
1993G072	8/9/1996
1993G072	17/9/1996
1993G072	28/9/1996
1993G072	9/10/1996
1993G076	15/5/1996
1993G076	25/5/1996
1993G076	5/6/1996
1993G076	15/6/1996
1993G076	25/6/1996
1993G076	5/7/1996
1993G089	18/7/1996
1993G089	30/7/1996
1993G089	8/8/1996
1993G089	10/8/1996
1993G092	31/5/1996
1993G092	9/6/1996
1993G092	18/6/1996
1993G092	28/6/1996

1993G092	7/7/1996
1993G092	16/7/1996
1993G092	27/7/1996
1993G092	6/8/1996
1993G097	31/5/1996
1993G097	11/6/1996
1993G097	22/6/1996
1993G097	3/7/1996
1993G097	14/7/1996
1993G097	24/7/1996
1993G097	3/8/1996
1993G097	14/8/1996
1993G103	30/6/1996
1993G103	10/7/1996
1993G103	20/7/1996
1993G103	30/7/1996
1993G103	9/8/1996
1993G103	18/8/1996
1993G103	29/8/1996
1993G103	7/9/1996
1993G105	30/6/1996
1993G105	10/7/1996
1993G105	20/7/1996
1993G105	31/7/1996
1993G105	9/8/1996
1993G105	20/8/1996
1993G105	31/8/1996
1993G105	9/9/1996
1993G105	19/9/1996
1993G111	21/6/1996
1993G111	2/7/1996
1993G111	11/7/1996
1993G111	20/7/1996
1993G111	21/7/1996
1993G111	31/7/1996
1993G111	10/8/1996
1993G111	20/8/1996
1993G111	30/8/1996
1993G111	10/9/1996
1993G112	21/5/1996
1993G112	1/6/1996
1993G112	11/6/1996
1993G112	22/6/1996
1993G112	4/7/1996
1993G112	13/7/1996
1993G112	22/7/1996

1993G112	1/8/1996
1993G112	9/8/1996
1993G117	8/5/1996
1993G117	9/6/1996
1993G117	20/6/1996
1993G117	30/6/1996
1993G117	1/7/1996
1993G117	22/7/1996
1993G118	16/5/1996
1993G118	27/5/1996
1993G118	5/6/1996
1993G118	24/6/1996
1993G118	4/7/1996
1993G118	14/7/1996
1993G118	24/7/1996
1993G122	19/7/1996
1993G122	1/8/1996
1993G122	12/8/1996
1993G122	22/8/1996
1993G122	2/9/1996
1993G122	14/9/1996
1993G125	13/7/1996
1993G125	23/7/1996
1993G125	2/8/1996
1993G125	13/8/1996
1993G125	14/8/1996
1993G125	30/8/1996
1993G125	8/9/1996
1993G125	18/9/1996
1993G125	30/9/1996
1993G125	8/10/1996
1993G129	9/7/1996
1993G129	18/7/1996
1993G129	5/8/1996
1993G129	14/8/1996
1993G129	23/8/1996
1993G129	1/9/1996
1993G129	2/9/1996
1993G130	5/8/1996
1993G130	15/8/1996
1993G130	25/8/1996
1993G130	26/8/1996
1993G130	15/9/1996
1993G130	4/10/1996
1993G130	14/10/1996
1993G133	10/6/1996

1996G001	7/5/1996
1996G002	12/10/1996
1996G003	8/5/1996
1996G003	18/5/1996
1996G003	29/5/1996
1996G003	9/6/1996
1996G003	19/6/1996
1996G003	1/7/1996
1996G003	12/7/1996
1996G004	26/5/1996
1996G004	7/6/1996
1996G004	16/6/1996
1996G004	5/7/1996
1996G004	16/7/1996
1996G005	14/5/1996
1996G005	23/5/1996
1996G005	1/6/1996
1996G005	11/6/1996
1996G006	10/5/1996
1996G007	10/5/1996
1996G007	19/5/1996
1996G007	29/5/1996
1996G007	8/6/1996
1996G007	18/6/1996
1996G007	28/6/1996
1996G007	8/7/1996
1996G008	10/5/1996
1996G008	23/5/1996
1996G008	5/6/1996
1996G008	17/6/1996
1996G008	28/6/1996
1996G009	10/5/1996
1996G009	20/5/1996
1996G009	1/6/1996
1996G009	10/6/1996
1996G009	19/6/1996
1996G009	28/6/1996
1996G009	7/7/1996
1996G009	16/7/1996
1996G009	25/7/1996
1996G010	3/6/1996
1996G010	22/6/1996
1996G010	2/7/1996
1996G010	11/7/1996
1996G010	20/7/1996
1996G010	31/7/1996

1996G010	9/8/1996
1996G010	19/8/1996
1996G011	10/5/1996
1996G011	21/5/1996
1996G011	2/6/1996
1996G011	14/6/1996
1996G012	11/5/1996
1996G012	23/5/1996
1996G013	13/5/1996
1996G013	25/5/1996
1996G013	6/6/1996
1996G013	15/6/1996
1996G013	25/6/1996
1996G013	5/7/1996
1996G013	16/7/1996
1996G014	12/5/1996
1996G014	22/5/1996
1996G014	3/6/1996
1996G015	11/5/1996
1996G015	21/5/1996
1996G015	1/6/1996
1996G015	11/6/1996
1996G015	21/6/1996
1996G015	2/7/1996
1996G016	15/6/1996
1996G016	25/6/1996
1996G016	5/7/1996
1996G016	15/7/1996
1996G016	25/7/1996
1996G016	3/8/1996
1996G016	13/8/1996
1996G016	22/8/1996
1996G016	2/9/1996
1996G017	13/8/1996
1996G017	23/8/1996
1996G017	2/9/1996
1996G017	11/9/1996
1996G017	12/9/1996
1996G017	21/9/1996
1996G017	30/9/1996
1996G017	9/10/1996
1996G018	15/5/1996
1996G018	25/5/1996
1996G018	5/6/1996
1996G018	14/6/1996
1996G018	23/6/1996

1996G018	2/7/1996
1996G018	3/7/1996
1996G018	12/7/1996
1996G019	25/6/1996
1996G019	5/7/1996
1996G019	16/7/1996
1996G019	25/7/1996
1996G019	3/8/1996
1996G019	13/8/1996
1996G019	23/8/1996
1996G020	31/5/1996
1996G020	10/6/1996
1996G020	20/6/1996
1996G020	30/6/1996
1996G020	9/7/1996
1996G020	18/7/1996
1996G020	28/7/1996
1996G021	23/5/1996
1996G021	3/6/1996
1996G021	13/6/1996
1996G021	14/6/1996
1996G021	26/6/1996
1996G021	5/7/1996
1996G021	15/7/1996
1996G021	25/7/1996
1996G022	20/5/1996
1996G022	30/5/1996
1996G022	9/6/1996
1996G022	18/6/1996
1996G022	27/6/1996
1996G022	6/7/1996
1996G022	15/7/1996
1996G022	24/7/1996
1996G022	2/8/1996
1996G022	12/8/1996
1996G022	22/8/1996
1996G023	5/7/1996
1996G023	16/7/1996
1996G023	27/7/1996
1996G023	7/8/1996
1996G023	19/8/1996
1996G023	29/8/1996
1996G023	30/8/1996
1996G023	10/9/1996
1996G023	21/9/1996
1996G024	14/5/1996

1996G024	24/5/1996
1996G024	3/6/1996
1996G024	14/6/1996
1996G024	25/6/1996
1996G024	4/7/1996
1996G024	14/7/1996
1996G024	24/7/1996
1996G024	5/8/1996
1996G025	28/9/1996
1996G025	7/10/1996
1996G026	9/6/1996
1996G026	21/6/1996
1996G026	2/7/1996
1996G026	14/7/1996
1996G026	3/8/1996
1996G026	14/8/1996
1996G027	28/9/1996
1996G027	9/10/1996
1996G028	18/6/1996
1996G028	8/7/1996
1996G029	6/6/1996
1996G029	18/6/1996
1996G029	2/7/1996
1996G029	12/7/1996
1996G029	2/8/1996
1996G029	12/8/1996
1996G029	23/8/1996
1996G030	12/6/1996
1996G030	22/6/1996
1996G030	4/7/1996
1996G030	14/7/1996
1996G030	23/7/1996
1996G030	24/7/1996
1996G030	2/8/1996
1996G030	12/8/1996
1996G031	7/6/1996
1996G031	17/6/1996
1996G031	28/6/1996
1996G032	8/6/1996
1996G032	19/6/1996
1996G032	29/6/1996
1996G032	8/7/1996
1996G032	19/7/1996
1996G032	31/7/1996
1996G033	20/6/1996
1996G033	30/6/1996

1996G033	20/7/1996
1996G033	1/8/1996
1996G034	8/6/1996
1996G034	19/6/1996
1996G034	30/6/1996
1996G034	10/7/1996
1996G034	20/7/1996
1996G034	31/7/1996
1996G034	10/8/1996
1996G034	20/8/1996
1996G034	31/8/1996
1996G034	9/9/1996
1996G035	23/6/1996
1996G035	5/7/1996
1996G035	26/7/1996
1996G035	6/8/1996
1996G035	17/8/1996
1996G035	27/8/1996
1996G035	7/9/1996
1996G036	13/8/1996
1996G036	23/8/1996
1996G036	2/9/1996
1996G036	11/9/1996
1996G036	20/9/1996
1996G036	22/9/1996
1996G036	1/10/1996
1996G036	12/10/1996
1996G037	20/9/1996
1996G038	8/7/1996
1996G038	19/7/1996
1996G038	20/7/1996
1996G038	31/7/1996
1996G038	21/8/1996
1996G038	30/8/1996
1996G038	1/9/1996
1996G038	10/9/1996
1996G039	12/7/1996
1996G039	22/7/1996
1996G039	1/8/1996
1996G040	8/8/1996
1996G040	18/8/1996
1996G040	29/8/1996
1996G040	7/9/1996
1996G040	27/9/1996
1996G041	7/7/1996
1996G041	17/7/1996

1996G041	27/7/1996
1996G041	28/7/1996
1996G041	6/8/1996
1996G041	25/8/1996
1996G041	4/9/1996
1996G041	14/9/1996
1996G041	24/9/1996
1996G042	3/10/1996
1996G042	12/10/1996
1996G043	15/10/1996
1996G044	27/6/1996
1996G044	8/7/1996
1996G044	18/7/1996
1996G045	4/8/1996
1996G045	14/8/1996
1996G045	1/9/1996
1996G045	28/9/1996
1996G045	7/10/1996
1996G045	17/10/1996
1996G046	2/6/1996
1996G046	12/6/1996
1996G046	22/6/1996
1996G046	3/7/1996
1996G046	12/7/1996
1996G046	22/7/1996
1996G046	2/8/1996
1996G047	13/9/1996
1996G047	3/10/1996
1996G047	14/10/1996
1996G048	23/6/1996
1996G048	4/7/1996
1996G048	14/7/1996
1996G048	23/7/1996
1996G048	2/8/1996
1996G048	11/8/1996
1996G048	21/8/1996
1996G048	1/9/1996
1996G049	1/10/1996
1996G049	11/10/1996
1996G050	18/8/1996
1996G050	29/8/1996
1996G050	19/9/1996
1996G050	30/9/1996
1996G050	10/10/1996
1996G050	11/10/1996
1996G051	6/5/1996

1996G051	18/5/1996
1996G051	29/5/1996
1996G051	8/6/1996
1996G051	19/6/1996
1996G051	30/6/1996
1996G051	11/7/1996
1996G051	22/7/1996
1996G051	2/8/1996
1996G052	7/5/1996
1996G052	18/5/1996
1996G052	29/5/1996
1996G052	9/6/1996
1996G053	7/5/1996
1996G053	19/5/1996
1996G053	31/5/1996
1996G053	10/6/1996
1996G053	20/6/1996
1996G053	1/7/1996
1996G054	24/8/1996
1996G054	3/9/1996
1996G054	13/9/1996
1996G054	3/10/1996
1996G055	3/9/1996
1996G056	9/6/1996
1996G056	20/7/1996
1996G057	23/6/1996
1996G058	5/7/1996
1996G058	15/7/1996
1996G058	25/7/1996
1996G059	29/6/1996
1996G059	10/7/1996
1996G059	20/7/1996
1996G059	30/7/1996
1996G059	9/8/1996
1996G060	2/7/1996
1996G060	12/7/1996
1996G060	22/7/1996
1996G060	1/8/1996
1996G060	13/8/1996
1996G060	25/8/1996
1993G004	7/5/1997
1993G004	17/5/1997
1993G004	27/5/1997
1993G004	5/6/1997
1993G004	15/6/1997
1993G004	24/6/1997

1993G004	5/7/1997
1993G016	6/5/1997
1993G016	15/5/1997
1993G016	25/5/1997
1993G016	3/6/1997
1993G016	14/6/1997
1993G022	26/6/1997
1993G022	19/7/1997
1993G029	13/5/1997
1993G029	22/5/1997
1993G029	31/5/1997
1993G029	10/6/1997
1993G029	19/6/1997
1993G029	20/6/1997
1993G029	29/6/1997
1993G029	9/7/1997
1993G058	18/6/1997
1993G058	19/6/1997
1993G058	30/6/1997
1993G058	22/7/1997
1993G058	3/8/1997
1993G058	13/8/1997
1993G058	24/8/1997
1993G060	17/7/1997
1993G060	28/9/1997
1993G073	23/6/1997
1993G073	3/7/1997
1993G073	13/7/1997
1993G073	23/7/1997
1993G073	2/8/1997
1993G073	12/8/1997
1993G073	21/8/1997
1993G073	31/8/1997
1993G073	10/9/1997
1993G073	20/9/1997
1993G073	2/10/1997
1993G074	23/7/1997
1993G074	2/8/1997
1993G074	12/8/1997
1993G074	23/8/1997
1993G074	2/9/1997
1993G104	10/7/1997
1993G104	21/7/1997
1993G104	22/7/1997
1993G104	11/8/1997
1993G104	21/8/1997

1993G107	29/6/1997
1993G107	9/7/1997
1993G107	19/7/1997
1993G107	29/7/1997
1993G107	8/8/1997
1993G107	19/8/1997
1993G108	25/7/1997
1993G108	4/8/1997
1993G108	14/8/1997
1993G108	24/8/1997
1993G108	15/9/1997
1993G108	25/9/1997
1993G116	20/5/1997
1993G116	29/5/1997
1993G116	10/6/1997
1993G116	21/6/1997
1993G116	1/7/1997
1993G116	11/7/1997
1993G116	22/7/1997
1993G116	2/8/1997
1993G116	12/8/1997
1994G006	26/6/1997
1994G006	6/7/1997
1994G006	16/7/1997
1994G006	16/7/1997
1994G006	26/7/1997
1994G006	5/8/1997
1994G006	15/8/1997
1994G006	25/8/1997
1994G006	5/9/1997
1994G016	15/5/1997
1994G016	25/5/1997
1994G016	4/6/1997
1994G016	16/6/1997
1994G016	26/6/1997
1994G016	7/7/1997
1994G017	12/5/1997
1994G017	21/5/1997
1994G017	31/5/1997
1994G017	9/6/1997
1994G017	18/6/1997
1994G017	29/6/1997
1994G022	22/7/1997
1994G022	1/8/1997
1994G022	12/8/1997
1994G022	2/9/1997

1994G025	13/6/1997
1994G025	23/6/1997
1994G025	23/6/1997
1994G025	12/7/1997
1994G025	13/7/1997
1994G025	23/7/1997
1994G025	1/8/1997
1994G025	12/8/1997
1994G032	6/5/1997
1994G032	16/5/1997
1994G032	26/5/1997
1994G032	5/6/1997
1994G032	15/6/1997
1994G038	9/7/1997
1994G038	20/7/1997
1994G038	30/7/1997
1994G038	10/8/1997
1994G038	21/8/1997
1994G038	31/8/1997
1994G038	12/9/1997
1994G040	7/5/1997
1994G040	12/7/1997
1994G040	5/8/1997
1994G040	18/8/1997
1994G040	28/8/1997
1994G040	8/9/1997
1994G048	10/5/1997
1994G048	20/5/1997
1994G048	29/5/1997
1994G048	8/6/1997
1994G048	18/6/1997
1994G048	28/6/1997
1994G048	8/7/1997
1994G048	19/7/1997
1994G060	6/5/1997
1994G060	19/5/1997
1994G060	29/5/1997
1994G060	9/6/1997
1994G060	9/6/1997
1994G060	20/6/1997
1994G060	30/6/1997
1994G060	11/7/1997
1994G060	22/7/1997
1995G016	9/7/1997
1995G016	20/7/1997
1995G016	31/7/1997

1995G016	21/8/1997
1995G016	1/9/1997
1995G016	12/9/1997
1995G016	22/9/1997
1995G016	2/10/1997
1995G016	13/10/1997
1995G075	26/5/1997
1995G075	5/6/1997
1995G075	14/6/1997
1995G075	24/6/1997
1995G075	4/7/1997
1995G075	13/7/1997
1995G075	23/7/1997
1995G075	3/8/1997
1997G001	6/5/1997
1997G002	6/5/1997
1997G002	16/5/1997
1997G002	26/5/1997
1997G003	16/9/1997
1997G003	25/9/1997
1997G003	4/10/1997
1997G003	14/10/1997
1997G004	8/5/1997
1997G005	9/5/1997
1997G006	10/5/1997
1997G006	19/5/1997
1997G007	10/5/1997
1997G007	19/5/1997
1997G007	30/5/1997
1997G008	11/5/1997
1997G008	23/5/1997
1997G009	11/5/1997
1997G009	21/5/1997
1997G009	30/5/1997
1997G009	9/6/1997
1997G010	11/5/1997
1997G011	12/5/1997
1997G011	22/5/1997
1997G011	1/6/1997
1997G011	11/6/1997
1997G011	21/6/1997
1997G011	22/6/1997
1997G011	2/7/1997
1997G012	12/5/1997
1997G012	22/5/1997
1997G012	2/6/1997

1997G012	14/6/1997
1997G012	15/6/1997
1997G012	25/6/1997
1997G012	5/7/1997
1997G012	15/7/1997
1997G013	13/5/1997
1997G013	23/5/1997
1997G013	1/6/1997
1997G013	2/6/1997
1997G013	13/6/1997
1997G014	14/5/1997
1997G014	24/5/1997
1997G014	3/6/1997
1997G014	14/6/1997
1997G015	14/5/1997
1997G015	24/5/1997
1997G015	6/6/1997
1997G016	16/5/1997
1997G016	28/5/1997
1997G016	10/6/1997
1997G016	22/6/1997
1997G017	16/5/1997
1997G017	27/5/1997
1997G017	7/6/1997
1997G017	18/6/1997
1997G017	27/6/1997
1997G017	7/7/1997
1997G017	17/7/1997
1997G017	27/7/1997
1997G018	17/5/1997
1997G018	15/6/1997
1997G018	16/6/1997
1997G019	20/5/1997
1997G019	30/5/1997
1997G019	11/6/1997
1997G020	21/5/1997
1997G020	12/6/1997
1997G020	23/6/1997
1997G020	4/7/1997
1997G021	22/5/1997
1997G021	23/5/1997
1997G021	1/6/1997
1997G021	11/6/1997
1997G021	12/6/1997
1997G021	21/6/1997
1997G021	1/7/1997

1997G022	22/5/1997
1997G022	1/6/1997
1997G022	2/6/1997
1997G022	13/6/1997
1997G022	23/6/1997
1997G022	24/6/1997
1997G022	5/7/1997
1997G022	16/7/1997
1997G022	8/8/1997
1997G023	8/10/1997
1997G024	24/5/1997
1997G025	27/5/1997
1997G025	8/6/1997
1997G025	21/6/1997
1997G025	22/6/1997
1997G025	23/6/1997
1997G025	5/7/1997
1997G026	27/5/1997
1997G026	6/6/1997
1997G026	17/6/1997
1997G026	27/6/1997
1997G026	7/7/1997
1997G026	17/7/1997
1997G026	28/7/1997
1997G026	7/8/1997
1997G026	20/8/1997
1997G027	28/5/1997
1997G027	11/6/1997
1997G027	23/6/1997
1997G027	5/7/1997
1997G027	17/7/1997
1997G027	30/7/1997
1997G028	31/5/1997
1997G028	12/6/1997
1997G028	24/6/1997
1997G028	4/7/1997
1997G028	15/7/1997
1997G028	26/7/1997
1997G028	5/8/1997
1997G028	17/8/1997
1997G029	3/6/1997
1997G029	14/6/1997
1997G029	24/6/1997
1997G029	25/6/1997
1997G029	5/7/1997
1997G029	14/7/1997

1997G029	24/7/1997
1997G029	3/8/1997
1997G029	13/8/1997
1997G030	6/6/1997
1997G030	18/6/1997
1997G030	29/6/1997
1997G030	10/7/1997
1997G030	22/7/1997
1997G031	8/6/1997
1997G031	18/6/1997
1997G031	29/6/1997
1997G031	9/7/1997
1997G031	19/7/1997
1997G031	20/7/1997
1997G031	30/7/1997
1997G032	10/6/1997
1997G032	22/6/1997
1997G032	2/7/1997
1997G032	22/7/1997
1997G032	2/8/1997
1997G033	11/6/1997
1997G033	21/6/1997
1997G033	24/6/1997
1997G033	15/7/1997
1997G034	11/6/1997
1997G034	23/6/1997
1997G034	4/7/1997
1997G034	14/7/1997
1997G034	24/7/1997
1997G034	4/8/1997
1997G035	11/6/1997
1997G035	22/6/1997
1997G035	2/7/1997
1997G035	13/7/1997
1997G035	23/7/1997
1997G035	3/8/1997
1997G036	13/6/1997
1997G036	23/6/1997
1997G036	3/7/1997
1997G036	13/7/1997
1997G036	23/7/1997
1997G036	1/8/1997
1997G036	10/8/1997
1997G036	21/8/1997
1997G036	31/8/1997
1997G036	2/9/1997

1997G037	14/6/1997
1997G037	24/6/1997
1997G037	4/7/1997
1997G037	13/7/1997
1997G037	25/7/1997
1997G037	3/8/1997
1997G038	19/6/1997
1997G038	29/6/1997
1997G038	9/7/1997
1997G038	20/7/1997
1997G038	30/7/1997
1997G038	9/8/1997
1997G038	20/8/1997
1997G039	21/6/1997
1997G039	1/7/1997
1997G039	11/7/1997
1997G039	21/7/1997
1997G039	31/7/1997
1997G039	10/8/1997
1997G039	20/8/1997
1997G040	22/6/1997
1997G040	4/7/1997
1997G040	14/7/1997
1997G040	24/7/1997
1997G040	3/8/1997
1997G040	13/8/1997
1997G040	23/8/1997
1997G041	1/7/1997
1997G041	11/7/1997
1997G041	21/7/1997
1997G041	31/7/1997
1997G041	9/8/1997
1997G041	20/8/1997
1997G042	3/7/1997
1997G042	14/7/1997
1997G042	25/7/1997
1997G042	4/8/1997
1997G042	14/8/1997
1997G042	14/8/1997
1997G042	16/8/1997
1997G042	27/8/1997
1997G042	8/9/1997
1997G043	5/7/1997
1997G043	15/7/1997
1997G043	26/7/1997
1997G043	5/8/1997

1997G043	16/8/1997
1997G043	25/8/1997
1997G043	5/9/1997
1997G043	16/9/1997
1997G043	16/9/1997
1997G044	5/7/1997
1997G044	16/7/1997
1997G044	26/7/1997
1997G044	4/8/1997
1997G044	14/8/1997
1997G044	24/8/1997
1997G045	6/8/1997
1997G045	16/8/1997
1997G045	27/8/1997
1997G045	5/9/1997
1997G045	15/9/1997
1997G046	8/7/1997
1997G046	19/7/1997
1997G046	29/7/1997
1997G046	8/8/1997
1997G046	18/8/1997
1997G046	19/8/1997
1997G046	29/8/1997
1997G046	8/9/1997
1997G047	11/7/1997
1997G047	22/7/1997
1997G047	3/8/1997
1997G047	14/8/1997
1997G047	24/8/1997
1997G047	3/9/1997
1997G047	14/9/1997
1997G048	13/7/1997
1997G048	23/7/1997
1997G048	3/8/1997
1997G048	14/8/1997
1997G048	27/8/1997
1997G048	30/8/1997
1997G049	15/7/1997
1997G049	4/8/1997
1997G049	14/8/1997
1997G049	24/8/1997
1997G049	4/9/1997
1997G050	16/7/1997
1997G050	26/7/1997
1997G050	5/8/1997
1997G050	15/8/1997

1997G050	24/8/1997
1997G050	3/9/1997
1997G050	4/9/1997
1997G050	23/9/1997
1997G051	21/7/1997
1997G051	1/8/1997
1997G051	13/8/1997
1997G052	22/7/1997
1997G052	2/8/1997
1997G052	13/8/1997
1997G052	24/8/1997
1997G052	4/9/1997
1997G052	16/9/1997
1997G053	29/7/1997
1997G053	10/8/1997
1997G053	20/8/1997
1997G053	10/9/1997
1997G053	20/9/1997
1997G053	1/10/1997
1997G054	4/8/1997
1997G054	16/8/1997
1997G054	17/8/1997
1997G054	29/8/1997
1997G054	30/8/1997
1997G054	31/8/1997
1997G054	23/9/1997
1997G055	10/8/1997
1997G055	22/8/1997
1997G055	11/9/1997
1997G055	2/10/1997
1997G055	12/10/1997
1997G055	14/10/1997
1997G056	21/8/1997
1997G056	31/8/1997
1997G056	11/9/1997
1997G056	21/9/1997
1997G056	1/10/1997
1997G056	11/10/1997
1997G057	26/8/1997
1997G057	6/9/1997
1997G057	16/9/1997
1997G057	25/9/1997
1997G057	4/10/1997
1997G057	14/10/1997
1997G058	3/9/1997
1997G058	5/9/1997

1997G058	18/9/1997
1997G058	30/9/1997
1997G058	13/10/1997
1997G059	22/6/1997
1997G060	22/6/1997
1997G060	5/7/1997
1997G060	18/7/1997
1997G060	30/7/1997
1997G060	10/8/1997
1997G060	21/8/1997
1997G061	30/6/1997
1997G061	10/7/1997
1997G062	11/8/1997
1997G062	21/8/1997
1997G062	1/9/1997
1997G062	12/9/1997
1997G062	23/9/1997
1997G063	6/7/1997
1997G063	16/7/1997
1997G063	27/7/1997
1997G063	6/8/1997
1997G063	18/8/1997
1997G063	29/8/1997
1997G063	11/9/1997
1997G063	12/9/1997
1997G064	9/7/1997
1997G064	21/7/1997
1997G064	1/8/1997
1993G008	3/7/1998
1993G008	14/7/1998
1993G008	15/7/1998
1993G008	25/7/1998
1993G008	3/8/1998
1993G008	13/8/1998
1993G008	22/8/1998
1993G008	23/8/1998
1993G008	11/9/1998
1993G008	21/9/1998
1993G039	25/5/1998
1993G039	4/6/1998
1993G039	6/6/1998
1993G039	16/6/1998
1993G039	25/6/1998
1993G039	5/7/1998
1993G039	14/7/1998
1993G039	15/7/1998

1993G039	15/7/1998
1993G039	16/7/1998
1993G039	26/7/1998
1993G039	26/7/1998
1993G039	27/7/1998
1993G039	5/8/1998
1993G039	7/8/1998
1993G050	12/5/1998
1993G050	22/5/1998
1993G050	31/5/1998
1993G050	10/6/1998
1993G050	19/6/1998
1993G050	28/6/1998
1993G050	7/7/1998
1993G050	16/7/1998
1993G050	25/7/1998
1993G050	4/8/1998
1993G057	1/6/1998
1993G057	12/6/1998
1993G057	29/6/1998
1993G057	9/7/1998
1993G057	10/7/1998
1993G057	19/7/1998
1993G057	28/7/1998
1993G057	7/8/1998
1993G057	7/8/1998
1993G057	8/8/1998
1993G057	18/8/1998
1993G057	29/8/1998
1993G076	19/6/1998
1993G076	29/6/1998
1993G076	30/6/1998
1993G076	13/7/1998
1993G076	14/7/1998
1993G076	24/7/1998
1993G076	4/8/1998
1993G076	15/8/1998
1993G076	16/8/1998
1993G078	5/6/1998
1993G078	14/6/1998
1993G078	23/6/1998
1993G078	3/7/1998
1993G078	4/7/1998
1993G078	13/7/1998
1993G078	22/7/1998
1993G078	1/8/1998

1993G085	6/7/1998
1993G096	18/6/1998
1993G096	29/6/1998
1993G096	30/6/1998
1993G096	30/6/1998
1993G096	9/7/1998
1993G096	12/7/1998
1993G096	21/7/1998
1993G096	21/7/1998
1993G096	30/7/1998
1993G096	31/7/1998
1993G096	10/8/1998
1993G096	18/8/1998
1993G096	19/8/1998
1993G096	29/8/1998
1993G111	4/6/1998
1993G111	14/6/1998
1993G111	23/6/1998
1993G111	3/7/1998
1993G111	25/7/1998
1993G111	4/8/1998
1994G010	16/4/1998
1994G010	28/4/1998
1994G032	3/6/1998
1994G032	14/6/1998
1994G032	25/6/1998
1994G032	4/7/1998
1994G032	14/7/1998
1994G032	14/7/1998
1994G049	21/5/1998
1994G049	29/6/1998
1994G051	15/4/1998
1994G051	25/4/1998
1994G051	5/5/1998
1994G051	15/5/1998
1994G051	25/5/1998
1994G051	4/6/1998
1994G051	13/6/1998
1994G051	22/6/1998
1994G052	8/4/1998
1994G052	19/4/1998
1994G052	2/5/1998
1994G052	14/5/1998
1994G052	3/6/1998
1994G058	29/4/1998
1994G058	11/5/1998

1994G058	21/5/1998
1994G058	30/5/1998
1994G058	10/6/1998
1994G058	19/6/1998
1994G058	29/6/1998
1994G058	29/6/1998
1994G058	9/7/1998
1994G058	10/7/1998
1995G004	10/5/1998
1995G004	11/5/1998
1995G004	23/5/1998
1995G004	2/6/1998
1995G004	12/6/1998
1995G004	22/6/1998
1995G004	23/6/1998
1995G004	3/7/1998
1995G004	13/7/1998
1995G004	24/7/1998
1995G013	9/4/1998
1995G013	18/4/1998
1995G013	27/4/1998
1995G013	5/5/1998
1995G033	17/4/1998
1995G033	27/4/1998
1995G033	6/5/1998
1995G033	14/5/1998
1995G033	22/5/1998
1995G040	4/6/1998
1995G040	13/6/1998
1995G040	22/6/1998
1995G040	30/6/1998
1995G040	9/7/1998
1995G048	28/6/1998
1995G048	9/7/1998
1995G048	18/7/1998
1995G048	27/7/1998
1995G048	6/8/1998
1995G048	18/8/1998
1995G053	20/6/1998
1995G053	29/6/1998
1995G053	8/7/1998
1995G053	17/7/1998
1995G053	26/7/1998
1995G053	7/8/1998
1995G056	2/6/1998
1995G056	16/6/1998

1995G056	26/6/1998
1995G056	27/6/1998
1995G056	7/7/1998
1995G056	17/7/1998
1995G056	26/7/1998
1995G056	27/7/1998
1995G056	6/8/1998
1995G056	6/8/1998
1995G093	23/4/1998
1995G093	3/5/1998
1995G093	13/5/1998
1995G093	22/5/1998
1995G093	31/5/1998
1995G093	9/6/1998
1995G093	18/6/1998
1995G093	26/6/1998
1995G093	5/7/1998
1995G093	14/7/1998
1995G093	15/7/1998
1995G095	22/8/1998
1995G095	31/8/1998
1995G095	9/9/1998
1995G095	9/9/1998
1995G095	19/9/1998
1995G095	28/9/1998
1995G095	28/9/1998
1995G095	8/10/1998
1995G095	17/10/1998
1995G101	12/4/1998
1995G101	22/4/1998
1995G101	3/5/1998
1995G103	8/6/1998
1995G103	10/8/1998
1995G104	19/4/1998
1995G104	11/5/1998
1995G104	21/5/1998
1995G104	30/5/1998
1995G107	24/6/1998
1995G107	6/7/1998
1995G107	17/7/1998
1995G107	27/7/1998
1995G107	7/8/1998
1996G003	29/5/1998
1996G003	9/6/1998
1996G003	19/6/1998
1996G003	29/6/1998

1996G003	10/7/1998
1996G003	20/7/1998
1996G003	29/7/1998
1996G003	11/8/1998
1996G034	14/4/1998
1996G034	23/4/1998
1996G034	3/5/1998
1996G034	13/5/1998
1996G034	23/5/1998
1996G034	4/6/1998
1996G045	8/4/1998
1996G045	17/4/1998
1996G045	26/4/1998
1996G045	5/5/1998
1996G045	14/5/1998
1996G045	24/5/1998
1998G001	10/4/1998
1998G001	21/4/1998
1998G001	3/5/1998
1998G001	13/5/1998
1998G001	24/5/1998
1998G002	10/4/1998
1998G002	21/4/1998
1998G002	2/5/1998
1998G002	12/5/1998
1998G002	22/5/1998
1998G002	2/6/1998
1998G002	12/6/1998
1998G003	11/4/1998
1998G003	21/4/1998
1998G003	2/5/1998
1998G003	12/5/1998
1998G003	1/6/1998
1998G003	11/6/1998
1998G003	21/6/1998
1998G003	1/7/1998
1998G004	11/4/1998
1998G004	22/4/1998
1998G004	4/5/1998
1998G005	12/4/1998
1998G005	21/4/1998
1998G005	1/5/1998
1998G005	2/5/1998
1998G005	12/5/1998
1998G006	12/4/1998
1998G006	23/4/1998

1998G006	24/4/1998
1998G006	3/5/1998
1998G006	13/5/1998
1998G006	23/5/1998
1998G006	1/6/1998
1998G006	10/6/1998
1998G006	19/6/1998
1998G006	29/6/1998
1998G007	13/4/1998
1998G007	25/4/1998
1998G007	18/5/1998
1998G007	30/5/1998
1998G007	31/5/1998
1998G008	13/4/1998
1998G008	22/4/1998
1998G008	1/5/1998
1998G008	11/5/1998
1998G008	20/5/1998
1998G008	29/5/1998
1998G008	8/6/1998
1998G009	14/4/1998
1998G009	26/4/1998
1998G009	9/5/1998
1998G010	14/4/1998
1998G010	25/4/1998
1998G010	27/4/1998
1998G010	28/4/1998
1998G010	8/5/1998
1998G010	18/5/1998
1998G010	28/5/1998
1998G010	8/6/1998
1998G010	30/6/1998
1998G011	14/4/1998
1998G011	25/4/1998
1998G011	6/5/1998
1998G011	16/5/1998
1998G011	27/5/1998
1998G012	17/4/1998
1998G013	14/4/1998
1998G013	24/4/1998
1998G013	4/5/1998
1998G014	28/4/1998
1998G014	11/5/1998
1998G014	22/5/1998
1998G014	2/6/1998
1998G014	12/6/1998

1998G014	23/6/1998
1998G014	6/7/1998
1998G015	2/5/1998
1998G015	12/5/1998
1998G015	22/5/1998
1998G015	31/5/1998
1998G015	9/6/1998
1998G015	18/6/1998
1998G015	27/6/1998
1998G016	4/5/1998
1998G016	14/5/1998
1998G016	25/5/1998
1998G016	4/6/1998
1998G016	13/6/1998
1998G016	23/6/1998
1998G016	3/7/1998
1998G016	3/7/1998
1998G016	4/7/1998
1998G016	13/7/1998
1998G016	14/7/1998
1998G016	14/7/1998
1998G016	15/7/1998
1998G016	26/7/1998
1998G017	11/5/1998
1998G017	21/5/1998
1998G017	31/5/1998
1998G017	11/6/1998
1998G017	21/6/1998
1998G017	1/7/1998
1998G017	11/7/1998
1998G017	12/7/1998
1998G017	21/7/1998
1998G017	22/7/1998
1998G017	22/7/1998
1998G017	2/8/1998
1998G018	12/5/1998
1998G018	22/5/1998
1998G018	1/6/1998
1998G018	10/6/1998
1998G018	19/6/1998
1998G018	29/6/1998
1998G019	12/5/1998
1998G020	15/5/1998
1998G020	27/5/1998
1998G020	8/6/1998
1998G020	17/6/1998

1998G020	26/6/1998
1998G020	5/7/1998
1998G020	15/7/1998
1998G021	18/5/1998
1998G021	27/5/1998
1998G021	8/6/1998
1998G021	17/6/1998
1998G021	17/6/1998
1998G021	26/6/1998
1998G021	27/6/1998
1998G021	7/7/1998
1998G021	17/7/1998
1998G021	27/7/1998
1998G021	28/7/1998
1998G022	20/5/1998
1998G022	29/5/1998
1998G022	9/6/1998
1998G022	18/6/1998
1998G022	27/6/1998
1998G022	7/7/1998
1998G022	17/7/1998
1998G022	26/7/1998
1998G022	26/7/1998
1998G023	21/5/1998
1998G023	29/5/1998
1998G023	1/6/1998
1998G023	11/6/1998
1998G023	21/6/1998
1998G023	30/6/1998
1998G023	10/7/1998
1998G024	27/5/1998
1998G024	8/6/1998
1998G024	18/6/1998
1998G024	28/6/1998
1998G024	28/6/1998
1998G024	30/6/1998
1998G024	30/6/1998
1998G024	12/7/1998
1998G024	21/7/1998
1998G024	1/8/1998
1998G024	12/8/1998
1998G025	28/5/1998
1998G025	18/6/1998
1998G025	29/6/1998
1998G025	9/7/1998
1998G025	10/7/1998

1998G026	9/6/1998
1998G026	19/6/1998
1998G026	29/6/1998
1998G026	8/7/1998
1998G026	17/7/1998
1998G026	26/7/1998
1998G026	5/8/1998
1998G027	2/6/1998
1998G028	3/6/1998
1998G028	12/6/1998
1998G028	22/6/1998
1998G028	23/6/1998
1998G028	2/7/1998
1998G028	12/7/1998
1998G028	22/7/1998
1998G029	7/6/1998
1998G029	16/6/1998
1998G029	25/6/1998
1998G029	4/7/1998
1998G029	14/7/1998
1998G029	14/7/1998
1998G029	23/7/1998
1998G029	24/7/1998
1998G029	2/8/1998
1998G030	8/6/1998
1998G030	9/7/1998
1998G031	13/6/1998
1998G031	23/6/1998
1998G031	3/7/1998
1998G031	13/7/1998
1998G031	22/7/1998
1998G031	24/7/1998
1998G031	4/8/1998
1998G031	13/8/1998
1998G031	23/8/1998
1998G031	3/9/1998
1998G031	14/9/1998
1998G032	28/6/1998
1998G032	8/7/1998
1998G032	9/7/1998
1998G032	18/7/1998
1998G033	29/6/1998
1998G033	11/7/1998
1998G033	20/7/1998
1998G033	31/7/1998
1998G033	12/8/1998

1998G034	29/6/1998
1998G034	29/6/1998
1998G035	8/7/1998
1998G035	19/7/1998
1998G035	20/7/1998
1998G035	29/7/1998
1998G035	8/8/1998
1998G035	11/8/1998
1998G035	22/8/1998
1998G035	2/9/1998
1998G036	9/7/1998
1998G036	20/7/1998
1998G036	29/7/1998
1998G036	9/8/1998
1998G037	10/7/1998
1998G037	19/7/1998
1998G037	20/7/1998
1998G037	30/7/1998
1998G037	10/8/1998
1998G037	20/8/1998
1998G037	21/8/1998
1998G037	31/8/1998
1998G037	11/9/1998
1998G038	11/7/1998
1998G039	12/7/1998
1998G039	23/7/1998
1998G039	24/7/1998
1998G039	3/8/1998
1998G039	14/8/1998
1998G039	14/8/1998
1998G040	15/7/1998
1998G040	26/7/1998
1998G041	16/7/1998
1998G041	26/7/1998
1998G041	5/8/1998
1998G041	6/8/1998
1998G041	27/8/1998
1998G041	5/9/1998
1998G041	15/9/1998
1998G041	15/9/1998
1998G041	16/9/1998
1998G042	17/7/1998
1998G043	17/7/1998
1998G043	28/7/1998
1998G043	7/8/1998
1998G043	18/8/1998

1998G044	19/7/1998
1998G045	20/7/1998
1998G045	1/8/1998
1998G045	12/8/1998
1998G045	23/8/1998
1998G045	4/9/1998
1998G045	14/9/1998
1998G046	21/7/1998
1998G046	30/7/1998
1998G046	10/8/1998
1998G046	18/8/1998
1998G046	27/8/1998
1998G046	5/9/1998
1998G046	14/9/1998
1998G046	24/9/1998
1998G047	26/7/1998
1998G048	2/8/1998
1998G048	12/8/1998
1998G049	10/8/1998
1998G050	11/8/1998
1998G051	24/8/1998
1998G051	4/9/1998
1998G051	13/9/1998
1998G051	14/9/1998
1998G051	24/9/1998
1998G051	4/10/1998
1998G052	25/8/1998
1998G052	22/9/1998
1998G052	2/10/1998
1998G052	12/10/1998
1998G053	17/6/1998
1998G054	10/9/1998
1998G055	15/10/1998
1993G125	22/6/1999
1993G130	15/9/1999
1999G017	8/5/1999
1999G017	10/5/1999
1999G050	25/8/1999
1993G007	19/4/1999
1993G007	2/5/1999
1993G007	12/5/1999
1993G007	23/5/1999
1993G007	3/6/1999
1993G007	15/6/1999
1993G007	26/6/1999
1993G007	27/6/1999

1993G007	9/7/1999
1993G009	7/6/1999
1993G009	18/6/1999
1993G009	29/6/1999
1993G009	9/7/1999
1993G009	19/7/1999
1993G009	29/7/1999
1993G019	18/7/1999
1993G019	30/7/1999
1993G019	19/8/1999
1993G019	30/8/1999
1993G022	19/8/1999
1993G022	30/8/1999
1993G022	10/9/1999
1993G022	20/9/1999
1993G023	1/7/1999
1993G023	12/7/1999
1993G023	23/7/1999
1993G023	2/8/1999
1993G023	3/8/1999
1993G023	13/8/1999
1993G023	24/8/1999
1993G023	3/9/1999
1993G023	15/9/1999
1993G028	11/4/1999
1993G028	21/4/1999
1993G028	1/5/1999
1993G028	11/5/1999
1993G028	20/5/1999
1993G028	30/5/1999
1993G028	10/6/1999
1993G028	22/6/1999
1993G028	2/7/1999
1993G030	2/7/1999
1993G030	13/7/1999
1993G037	5/7/1999
1993G037	16/7/1999
1993G037	27/7/1999
1993G037	7/8/1999
1993G037	19/8/1999
1993G037	1/9/1999
1993G066	4/6/1999
1993G066	16/6/1999
1993G066	28/6/1999
1993G066	9/7/1999
1993G066	11/7/1999

1993G066	23/7/1999
1993G071	22/6/1999
1993G071	2/7/1999
1993G071	12/7/1999
1993G071	23/7/1999
1993G071	2/8/1999
1993G071	12/8/1999
1993G071	21/8/1999
1993G071	31/8/1999
1993G071	10/9/1999
1993G077	8/6/1999
1993G077	19/6/1999
1993G077	30/6/1999
1993G077	10/7/1999
1993G077	20/7/1999
1993G077	21/7/1999
1993G077	31/7/1999
1993G077	11/8/1999
1993G087	22/7/1999
1993G087	11/8/1999
1993G087	31/8/1999
1993G087	10/9/1999
1993G087	21/9/1999
1993G087	1/10/1999
1993G088	1/7/1999
1993G088	12/7/1999
1993G088	23/7/1999
1993G088	3/8/1999
1993G088	13/8/1999
1993G088	23/8/1999
1993G092	5/5/1999
1993G092	14/5/1999
1993G092	24/5/1999
1993G092	3/6/1999
1993G092	13/6/1999
1993G092	24/6/1999
1993G092	24/6/1999
1993G092	4/7/1999
1993G093	24/7/1999
1993G093	4/8/1999
1993G098	10/5/1999
1993G098	28/6/1999
1993G098	9/7/1999
1993G098	20/7/1999
1993G098	31/7/1999
1993G103	20/6/1999

1993G103	30/6/1999
1993G103	11/7/1999
1993G103	22/7/1999
1993G103	3/8/1999
1993G103	12/8/1999
1993G103	21/8/1999
1993G103	31/8/1999
1993G103	10/9/1999
1993G103	21/9/1999
1993G105	22/6/1999
1993G105	3/7/1999
1993G105	15/7/1999
1993G105	27/7/1999
1993G105	6/8/1999
1993G105	17/8/1999
1993G105	28/8/1999
1993G105	8/9/1999
1993G105	20/9/1999
1993G109	29/7/1999
1993G112	8/6/1999
1993G112	8/6/1999
1993G112	20/6/1999
1993G112	11/7/1999
1993G112	20/7/1999
1993G112	22/7/1999
1993G112	10/8/1999
1993G112	12/8/1999
1993G112	22/8/1999
1993G112	1/9/1999
1993G116	3/6/1999
1993G116	14/6/1999
1993G116	25/6/1999
1993G116	26/6/1999
1993G116	7/7/1999
1993G116	17/7/1999
1993G116	18/7/1999
1993G116	29/7/1999
1993G116	30/7/1999
1993G116	10/8/1999
1993G116	20/8/1999
1993G122	10/7/1999
1993G122	23/7/1999
1993G122	3/8/1999
1993G122	14/8/1999
1993G122	26/8/1999
1993G122	5/9/1999

1993G122	15/9/1999
1993G122	26/9/1999
1993G125	3/7/1999
1993G125	13/7/1999
1993G125	24/7/1999
1993G125	3/8/1999
1993G125	14/8/1999
1993G125	24/8/1999
1993G125	2/9/1999
1993G125	12/9/1999
1993G125	24/9/1999
1993G129	12/8/1999
1993G129	21/8/1999
1993G129	31/8/1999
1993G129	9/9/1999
1993G129	7/10/1999
1993G129	16/10/1999
1993G130	16/8/1999
1993G130	26/9/1999
1993G130	26/9/1999
1993G130	6/10/1999
1993G130	7/10/1999
1993G130	7/10/1999
1993G130	17/10/1999
1994G013	6/7/1999
1994G013	9/7/1999
1994G013	11/7/1999
1994G013	13/7/1999
1994G013	19/7/1999
1994G013	23/7/1999
1994G013	24/7/1999
1994G013	10/8/1999
1994G013	12/8/1999
1994G013	14/8/1999
1994G013	16/8/1999
1994G013	17/8/1999
1994G013	19/8/1999
1994G019	30/5/1999
1994G019	9/6/1999
1994G019	23/6/1999
1994G019	3/7/1999
1994G019	13/7/1999
1994G019	17/7/1999
1994G019	28/7/1999
1994G019	7/8/1999
1994G019	7/8/1999

1994G019	9/8/1999
1994G029	30/6/1999
1994G029	1/7/1999
1994G029	25/7/1999
1994G029	5/8/1999
1994G029	15/8/1999
1994G029	27/8/1999
1994G029	6/9/1999
1994G029	16/9/1999
1994G029	27/9/1999
1994G046	7/8/1999
1994G046	18/8/1999
1994G046	30/8/1999
1994G046	9/9/1999
1994G046	21/9/1999
1994G046	3/10/1999
1994G051	29/5/1999
1994G051	8/6/1999
1994G051	9/6/1999
1994G051	20/6/1999
1994G051	30/6/1999
1994G051	10/7/1999
1994G051	10/7/1999
1994G051	22/7/1999
1994G063	9/5/1999
1994G063	19/5/1999
1994G063	29/5/1999
1994G063	8/6/1999
1994G063	19/6/1999
1994G063	29/6/1999
1994G063	9/7/1999
1994G063	22/7/1999
1995G001	30/5/1999
1995G001	11/6/1999
1995G001	22/6/1999
1995G001	2/7/1999
1995G001	14/7/1999
1995G012	28/6/1999
1995G020	27/4/1999
1995G020	7/5/1999
1995G020	17/5/1999
1995G020	27/5/1999
1995G020	5/6/1999
1995G020	7/6/1999
1995G020	17/6/1999
1995G020	28/6/1999

1995G020	8/7/1999
1995G020	19/7/1999
1995G020	21/7/1999
1995G023	19/4/1999
1995G023	29/4/1999
1995G023	8/5/1999
1995G023	17/5/1999
1995G023	26/5/1999
1995G029	1/4/1999
1995G029	12/4/1999
1995G029	22/4/1999
1995G029	4/5/1999
1995G032	28/7/1999
1995G032	7/8/1999
1995G032	18/8/1999
1995G032	29/8/1999
1995G032	8/9/1999
1995G032	18/9/1999
1995G032	28/9/1999
1995G038	9/6/1999
1995G038	21/6/1999
1995G038	13/7/1999
1995G038	24/7/1999
1995G038	4/8/1999
1995G038	14/8/1999
1995G046	8/6/1999
1995G046	20/6/1999
1995G046	1/7/1999
1995G046	11/7/1999
1995G046	23/7/1999
1995G046	2/8/1999
1995G046	11/8/1999
1995G046	22/8/1999
1995G046	1/9/1999
1995G065	9/8/1999
1995G065	19/8/1999
1995G065	29/8/1999
1995G065	8/9/1999
1995G065	19/9/1999
1995G065	29/9/1999
1995G065	29/9/1999
1995G065	19/10/1999
1995G069	14/7/1999
1995G069	27/7/1999
1995G069	6/8/1999
1995G069	16/8/1999

1995G069	27/8/1999
1995G069	6/9/1999
1995G069	16/9/1999
1995G099	28/6/1999
1995G099	9/7/1999
1995G099	21/7/1999
1995G099	23/7/1999
1995G099	24/7/1999
1995G099	4/8/1999
1995G099	15/8/1999
1995G099	27/8/1999
1995G099	8/9/1999
1995G109	15/7/1999
1995G109	28/7/1999
1996G007	27/4/1999
1996G007	8/5/1999
1996G007	18/5/1999
1996G007	29/5/1999
1996G007	8/6/1999
1996G007	9/6/1999
1996G007	21/6/1999
1996G009	26/5/1999
1996G009	29/5/1999
1996G009	10/6/1999
1996G009	21/6/1999
1996G009	1/7/1999
1996G009	2/7/1999
1996G009	3/7/1999
1996G009	12/7/1999
1996G009	22/7/1999
1996G009	1/8/1999
1996G009	11/8/1999
1996G009	21/8/1999
1996G010	28/6/1999
1996G010	8/7/1999
1996G010	17/7/1999
1996G010	28/7/1999
1996G010	6/8/1999
1996G010	7/8/1999
1996G010	17/8/1999
1996G010	27/8/1999
1996G010	6/9/1999
1996G012	31/3/1999
1996G012	11/4/1999
1996G012	22/4/1999
1996G012	3/5/1999

1996G012	14/5/1999
1996G012	26/5/1999
1996G012	29/5/1999
1996G012	12/6/1999
1996G021	16/4/1999
1996G021	26/4/1999
1996G021	8/5/1999
1996G021	18/5/1999
1996G021	29/5/1999
1996G021	8/6/1999
1996G021	20/6/1999
1996G021	1/7/1999
1996G024	7/4/1999
1996G024	16/4/1999
1996G024	26/4/1999
1996G038	1/6/1999
1996G038	12/6/1999
1996G038	12/6/1999
1996G038	23/6/1999
1996G038	24/6/1999
1996G038	3/7/1999
1996G038	12/7/1999
1996G038	23/7/1999
1996G038	2/8/1999
1996G038	12/8/1999
1996G038	23/8/1999
1996G046	19/5/1999
1996G046	31/5/1999
1996G046	10/6/1999
1996G046	20/6/1999
1996G046	21/6/1999
1996G046	22/6/1999
1996G046	3/7/1999
1996G046	13/7/1999
1996G046	24/7/1999
1996G046	26/7/1999
1996G046	6/8/1999
1999G001	1/4/1999
1999G001	14/4/1999
1999G001	27/4/1999
1999G001	9/5/1999
1999G001	12/5/1999
1999G002	4/4/1999
1999G002	15/4/1999
1999G003	5/4/1999
1999G003	16/4/1999

1999G003	28/4/1999
1999G003	8/5/1999
1999G003	20/5/1999
1999G003	30/5/1999
1999G003	11/6/1999
1999G004	6/4/1999
1999G004	19/4/1999
1999G004	29/4/1999
1999G004	9/5/1999
1999G004	19/5/1999
1999G004	29/5/1999
1999G004	8/6/1999
1999G004	19/6/1999
1999G004	1/7/1999
1999G005	7/4/1999
1999G005	16/4/1999
1999G005	27/4/1999
1999G005	6/5/1999
1999G005	15/5/1999
1999G005	24/5/1999
1999G005	2/6/1999
1999G006	7/4/1999
1999G006	18/4/1999
1999G006	28/4/1999
1999G006	7/5/1999
1999G006	8/5/1999
1999G006	17/5/1999
1999G006	27/5/1999
1999G006	6/6/1999
1999G006	20/6/1999
1999G007	9/4/1999
1999G007	20/4/1999
1999G007	1/5/1999
1999G007	11/5/1999
1999G007	21/5/1999
1999G007	31/5/1999
1999G007	10/6/1999
1999G007	21/6/1999
1999G008	17/4/1999
1999G008	28/4/1999
1999G008	8/5/1999
1999G008	17/5/1999
1999G008	27/5/1999
1999G008	6/6/1999
1999G008	17/6/1999
1999G008	18/6/1999

1999G009	20/4/1999
1999G009	30/4/1999
1999G009	19/5/1999
1999G009	29/5/1999
1999G009	7/6/1999
1999G009	17/6/1999
1999G010	20/4/1999
1999G010	2/5/1999
1999G010	12/5/1999
1999G010	23/5/1999
1999G010	3/6/1999
1999G010	16/6/1999
1999G011	9/5/1999
1999G011	20/5/1999
1999G011	8/6/1999
1999G012	21/4/1999
1999G012	5/5/1999
1999G012	18/5/1999
1999G013	22/4/1999
1999G013	1/5/1999
1999G013	13/5/1999
1999G013	24/5/1999
1999G013	3/6/1999
1999G013	14/6/1999
1999G014	26/4/1999
1999G014	7/5/1999
1999G014	17/5/1999
1999G014	27/5/1999
1999G014	5/6/1999
1999G014	16/6/1999
1999G014	17/6/1999
1999G014	28/6/1999
1999G014	9/7/1999
1999G014	20/7/1999
1999G014	1/8/1999
1999G015	26/4/1999
1999G015	6/5/1999
1999G015	16/5/1999
1999G015	26/5/1999
1999G015	27/5/1999
1999G015	5/6/1999
1999G015	15/6/1999
1999G015	15/6/1999
1999G015	25/6/1999
1999G015	25/6/1999
1999G016	2/5/1999

1999G016	15/5/1999
1999G016	26/5/1999
1999G016	27/5/1999
1999G016	7/6/1999
1999G016	19/6/1999
1999G016	3/7/1999
1999G017	12/5/1999
1999G017	24/5/1999
1999G017	3/6/1999
1999G017	17/6/1999
1999G017	29/6/1999
1999G017	30/6/1999
1999G017	13/7/1999
1999G018	15/5/1999
1999G018	25/5/1999
1999G018	5/6/1999
1999G018	16/6/1999
1999G018	27/6/1999
1999G018	7/7/1999
1999G018	8/7/1999
1999G018	19/7/1999
1999G018	10/8/1999
1999G019	15/5/1999
1999G019	24/5/1999
1999G019	4/6/1999
1999G019	16/6/1999
1999G019	26/6/1999
1999G019	6/7/1999
1999G019	7/7/1999
1999G019	18/7/1999
1999G020	17/5/1999
1999G020	28/5/1999
1999G020	9/6/1999
1999G020	21/6/1999
1999G020	2/7/1999
1999G020	13/7/1999
1999G020	24/7/1999
1999G021	18/5/1999
1999G021	31/5/1999
1999G021	12/6/1999
1999G021	23/6/1999
1999G021	3/7/1999
1999G021	14/7/1999
1999G022	18/5/1999
1999G022	29/5/1999
1999G022	8/6/1999

1999G022	19/6/1999
1999G022	30/6/1999
1999G022	10/7/1999
1999G022	21/7/1999
1999G022	31/7/1999
1999G023	20/5/1999
1999G023	2/6/1999
1999G023	16/6/1999
1999G023	27/6/1999
1999G023	28/6/1999
1999G023	9/7/1999
1999G023	20/7/1999
1999G023	21/7/1999
1999G023	22/7/1999
1999G023	2/8/1999
1999G023	14/8/1999
1999G023	16/8/1999
1999G024	21/5/1999
1999G024	31/5/1999
1999G024	31/5/1999
1999G024	10/6/1999
1999G024	21/6/1999
1999G024	22/6/1999
1999G024	3/7/1999
1999G024	14/7/1999
1999G024	24/7/1999
1999G024	25/7/1999
1999G024	4/8/1999
1999G025	21/5/1999
1999G026	22/5/1999
1999G026	1/6/1999
1999G026	23/6/1999
1999G027	25/5/1999
1999G027	17/6/1999
1999G027	17/6/1999
1999G027	28/6/1999
1999G027	9/7/1999
1999G027	19/7/1999
1999G027	20/7/1999
1999G027	31/7/1999
1999G027	21/8/1999
1999G027	31/8/1999
1999G027	12/9/1999
1999G028	27/5/1999
1999G028	17/6/1999
1999G028	28/6/1999

1999G029	28/5/1999
1999G029	8/6/1999
1999G029	8/6/1999
1999G029	12/6/1999
1999G029	20/6/1999
1999G029	30/6/1999
1999G029	10/7/1999
1999G029	21/7/1999
1999G030	29/5/1999
1999G030	9/6/1999
1999G030	21/6/1999
1999G030	2/7/1999
1999G030	14/7/1999
1999G030	25/7/1999
1999G031	31/5/1999
1999G031	11/6/1999
1999G031	22/6/1999
1999G031	2/7/1999
1999G031	3/7/1999
1999G031	14/7/1999
1999G032	15/6/1999
1999G032	26/6/1999
1999G032	28/6/1999
1999G032	8/7/1999
1999G032	18/7/1999
1999G032	19/7/1999
1999G032	29/7/1999
1999G032	30/7/1999
1999G032	19/8/1999
1999G032	30/8/1999
1999G033	16/6/1999
1999G033	27/6/1999
1999G033	9/7/1999
1999G033	20/7/1999
1999G033	1/8/1999
1999G034	17/6/1999
1999G034	8/7/1999
1999G034	17/7/1999
1999G034	27/7/1999
1999G034	6/8/1999
1999G034	17/8/1999
1999G035	18/6/1999
1999G035	28/6/1999
1999G035	29/6/1999
1999G035	9/7/1999
1999G035	9/7/1999

1999G035	10/7/1999
1999G035	20/7/1999
1999G035	30/7/1999
1999G035	19/8/1999
1999G035	29/8/1999
1999G035	29/8/1999
1999G035	9/9/1999
1999G036	21/6/1999
1999G036	3/7/1999
1999G036	15/7/1999
1999G036	27/7/1999
1999G036	8/8/1999
1999G036	19/8/1999
1999G036	30/8/1999
1999G037	21/6/1999
1999G037	3/7/1999
1999G037	13/7/1999
1999G037	14/7/1999
1999G037	24/7/1999
1999G037	3/8/1999
1999G037	14/8/1999
1999G038	6/7/1999
1999G038	16/7/1999
1999G038	28/7/1999
1999G038	7/8/1999
1999G038	17/8/1999
1999G038	27/8/1999
1999G038	6/9/1999
1999G039	22/6/1999
1999G039	6/7/1999
1999G039	17/7/1999
1999G039	28/7/1999
1999G039	9/8/1999
1999G039	11/8/1999
1999G039	22/8/1999
1999G039	2/9/1999
1999G039	13/9/1999
1999G040	23/6/1999
1999G040	3/7/1999
1999G040	13/7/1999
1999G040	23/7/1999
1999G040	3/8/1999
1999G041	23/6/1999
1999G041	3/7/1999
1999G041	13/7/1999
1999G041	24/7/1999

1999G042	26/6/1999
1999G042	6/7/1999
1999G042	16/7/1999
1999G042	21/8/1999
1999G043	27/6/1999
1999G043	8/7/1999
1999G043	18/7/1999
1999G043	29/7/1999
1999G043	29/7/1999
1999G043	8/8/1999
1999G043	19/8/1999
1999G044	7/7/1999
1999G044	18/7/1999
1999G044	19/7/1999
1999G044	31/7/1999
1999G044	11/8/1999
1999G044	12/8/1999
1999G044	23/8/1999
1999G045	10/7/1999
1999G045	21/7/1999
1999G046	12/7/1999
1999G046	24/7/1999
1999G046	5/8/1999
1999G047	21/7/1999
1999G047	1/8/1999
1999G047	12/8/1999
1999G047	23/8/1999
1999G048	22/7/1999
1999G048	2/8/1999
1999G048	13/8/1999
1999G048	22/8/1999
1999G048	1/9/1999
1999G048	2/9/1999
1999G048	12/9/1999
1999G048	23/9/1999
1999G049	23/7/1999
1999G049	1/8/1999
1999G049	11/8/1999
1999G049	20/8/1999
1999G049	30/8/1999
1999G049	30/8/1999
1999G049	30/8/1999
1999G049	8/9/1999
1999G049	18/9/1999
1999G049	28/9/1999
1999G050	24/7/1999

1999G050	13/8/1999
1999G050	15/8/1999
1999G050	3/9/1999
1999G050	24/9/1999
1999G050	4/10/1999
1999G051	4/8/1999
1999G051	16/8/1999
1999G051	27/8/1999
1999G051	5/9/1999
1999G051	15/9/1999
1999G052	5/8/1999
1999G053	7/8/1999
1999G053	18/8/1999
1999G054	7/8/1999
1999G054	18/8/1999
1999G055	1/9/1999
1999G055	11/9/1999
1999G055	22/9/1999
1999G055	12/10/1999
1999G056	12/8/1999
1999G056	21/8/1999
1999G056	30/8/1999
1999G056	31/8/1999
1999G056	31/8/1999
1999G056	9/9/1999
1999G056	19/9/1999
1999G056	28/9/1999
1999G056	7/10/1999
1999G057	14/8/1999
1999G057	23/8/1999
1999G058	17/8/1999
1999G058	28/8/1999
1999G058	10/9/1999
1999G058	20/9/1999
1999G058	30/9/1999
1999G058	10/10/1999
1999G059	18/8/1999
1999G060	27/8/1999
1999G060	6/9/1999
1999G060	16/9/1999
1999G060	16/9/1999
1999G060	27/9/1999
1999G060	7/10/1999
1999G060	18/10/1999
1999G061	30/8/1999
1999G061	1/10/1999

1999G061	3/10/1999
1999G061	4/10/1999
1999G061	4/10/1999
1999G061	14/10/1999
1999G062	26/9/1999
1999G062	5/10/1999
1999G062	6/10/1999
1999G062	16/10/1999
1999G063	1/9/1999
1999G064	22/9/1999
1999G065	25/9/1999
1999g065	4/10/1999
1999G065	14/10/1999
1999G066	28/9/1999
1999G066	8/10/1999
1999G066	18/10/1999
1999G066	19/10/1999
1999G067	1/10/1999
1999G067	12/10/1999
1999G067	22/10/1999
1999G068	14/10/1999
1993G001	8/5/2000
1993G001	18/5/2000
1993G001	28/5/2000
1993G001	6/6/2000
1993G001	15/6/2000
1993G001	23/6/2000
1993G001	4/7/2000
1993G001	15/7/2000
1993G001	17/5/2000
1993G081	7/4/2000
1993G081	20/4/2000
1993G081	2/5/2000
1993G081	13/5/2000
1993G081	24/5/2000
1993G081	4/6/2000
1993G117	25/5/2000
1993G117	4/6/2000
1993G117	13/6/2000
1993G117	23/6/2000
1993G117	4/7/2000
1993G117	14/7/2000
1993G117	3/5/2000
1993G117	14/5/2000
1993G117	25/7/2000
1994G006	16/6/2000

1994G006	27/6/2000
1994G006	8/7/2000
1994G006	18/7/2000
1994G006	28/7/2000
1994G006	7/8/2000
1994G006	18/8/2000
1994G006	29/8/2000
1994G006	10/9/2000
1994G016	10/4/2000
1994G016	2/5/2000
1994G016	12/5/2000
1994G016	22/5/2000
1994G016	20/4/2000
1994G022	20/7/2000
1994G025	4/6/2000
1994G025	17/6/2000
1994G025	27/6/2000
1994G025	7/7/2000
1994G025	16/7/2000
1994G025	25/7/2000
1994G025	25/7/2000
1994G025	26/7/2000
1994G025	4/8/2000
1995G008	20/4/2000
1995G008	2/5/2000
1995G008	23/5/2000
1995G008	2/6/2000
1995G008	12/6/2000
1995G008	12/5/2000
1995G008	25/6/2000
1995G016	3/5/2000
1995G016	14/5/2000
1995G016	25/5/2000
1995G016	5/6/2000
1995G016	15/6/2000
1995G016	25/6/2000
1995G016	16/7/2000
1995G016	25/7/2000
1995G016	4/8/2000
1995G016	15/8/2000
1995G016	28/8/2000
1995G016	24/5/2000
1995G016	5/7/2000
1995G021	22/5/2000
1995G021	1/6/2000
1995G021	12/6/2000

1995G021	23/6/2000
1995G021	3/7/2000
1995G021	14/7/2000
1995G021	25/7/2000
1995G024	28/4/2000
1995G024	8/5/2000
1995G024	31/5/2000
1995G024	10/6/2000
1995G024	21/6/2000
1995G024	12/7/2000
1995G024	23/7/2000
1995G024	19/4/2000
1995G024	18/5/2000
1995G024	1/7/2000
1995G049	17/6/2000
1995G049	7/7/2000
1995G049	18/7/2000
1995G049	27/7/2000
1995G049	8/8/2000
1995G049	19/8/2000
1995G049	31/8/2000
1995G049	6/8/2000
1995G057	27/6/2000
1995G057	10/7/2000
1995G057	12/8/2000
1995G057	24/8/2000
1995G057	16/6/2000
1995G057	1/8/2000
1995G076	19/6/2000
1995G076	30/6/2000
1995G076	10/7/2000
1995G076	20/7/2000
1995G076	31/7/2000
1995G076	10/8/2000
1995G080	22/6/2000
1995G080	3/7/2000
1995G080	14/7/2000
1995G080	24/7/2000
1995G080	3/8/2000
1995G080	23/5/2000
1995G080	2/6/2000
1995G080	13/6/2000
1995G084	10/8/2000
1995G084	22/8/2000
1995G084	1/9/2000
1995G084	11/9/2000

1995G084	22/9/2000
1995G084	3/10/2000
1995G084	13/10/2000
1996G045	6/8/2000
1996G045	16/8/2000
1996G045	25/8/2000
1996G045	4/9/2000
1996G045	14/9/2000
1996G045	23/9/2000
1996G045	28/7/2000
1996G045	3/10/2000
1996G045	3/10/2000
1996G045	13/10/2000
1997G006	29/5/2000
1997G006	29/6/2000
1997G006	8/7/2000
1997G006	9/6/2000
1997G006	19/6/2000
1997G006	18/7/2000
1997G006	18/7/2000
1997G022	27/5/2000
1997G022	7/6/2000
1997G022	16/6/2000
1997G022	27/6/2000
1997G022	6/7/2000
1997G022	17/7/2000
1997G022	26/5/2000
1997G022	26/6/2000
1997G025	16/6/2000
1997G025	29/6/2000
1997G025	10/7/2000
1997G025	22/7/2000
1997G025	2/8/2000
1997G025	13/8/2000
1997G025	23/8/2000
1997G025	3/9/2000
1997G025	4/9/2000
1997G064	9/5/2000
1997G064	19/5/2000
1997G064	29/5/2000
1997G064	20/6/2000
1997G064	1/7/2000
1997G064	9/6/2000
1997G064	12/7/2000
1998G049	23/5/2000
1998G049	23/6/2000

1998G049	4/7/2000
1998G049	1/6/2000
1998G049	15/7/2000
2000G001	7/4/2000
2000G002	19/4/2000
2000G002	12/5/2000
2000G002	24/5/2000
2000G002	29/4/2000
2000G002	2/5/2000
2000G003	27/4/2000
2000G003	7/5/2000
2000G004	15/4/2000
2000G004	27/4/2000
2000G004	8/5/2000
2000G006	25/4/2000
2000G006	6/5/2000
2000G006	25/5/2000
2000G006	3/6/2000
2000G006	26/6/2000
2000G006	8/7/2000
2000G006	16/5/2000
2000G007	2/5/2000
2000G007	24/5/2000
2000G007	5/6/2000
2000G007	13/5/2000
2000G008	19/5/2000
2000G008	8/5/2000
2000G009	23/5/2000
2000G009	1/6/2000
2000G009	22/6/2000
2000G009	2/7/2000
2000G009	13/7/2000
2000G009	1/8/2000
2000G009	16/8/2000
2000G009	12/6/2000
2000G009	22/7/2000
2000G009	14/8/2000
2000G010	24/5/2000
2000G010	3/6/2000
2000G010	15/6/2000
2000G010	26/6/2000
2000G010	17/7/2000
2000G010	1/8/2000
2000G010	6/7/2000
2000G011	26/5/2000
2000G011	16/6/2000

2000G011	27/6/2000
2000G011	5/6/2000
2000G012	31/5/2000
2000G012	10/6/2000
2000G012	21/6/2000
2000G012	12/7/2000
2000G012	16/8/2000
2000G012	2/7/2000
2000G012	23/7/2000
2000G012	2/8/2000
2000G013	1/6/2000
2000G014	12/6/2000
2000G014	24/6/2000
2000G014	5/7/2000
2000G014	16/7/2000
2000G014	6/8/2000
2000G014	20/8/2000
2000G014	1/6/2000
2000G014	27/7/2000
2000G015	14/6/2000
2000G015	3/6/2000
2000G016	11/6/2000
2000G016	20/6/2000
2000G016	29/6/2000
2000G016	9/7/2000
2000G017	16/6/2000
2000G017	27/6/2000
2000G017	20/7/2000
2000G017	9/7/2000
2000G018	17/6/2000
2000G018	10/7/2000
2000G019	16/6/2000
2000G019	27/6/2000
2000G019	7/7/2000
2000G020	18/6/2000
2000G020	30/6/2000
2000G020	10/7/2000
2000G021	19/6/2000
2000G021	1/7/2000
2000G021	15/7/2000
2000G021	26/7/2000
2000G021	7/8/2000
2000G021	20/8/2000
2000G022	19/6/2000
2000G022	30/6/2000
2000G022	12/8/2000

2000G022	10/7/2000
2000G022	1/8/2000
2000G023	30/6/2000
2000G023	20/6/2000
2000G024	15/7/2000
2000G024	4/8/2000
2000G024	23/8/2000
2000G024	2/9/2000
2000G024	13/9/2000
2000G024	2/10/2000
2000G025	4/8/2000
2000G025	16/8/2000
2000G025	25/8/2000
2000G025	4/9/2000
2000G025	14/9/2000
2000G025	24/9/2000
2000G025	3/10/2000
2000G025	4/10/2000
2000G026	21/8/2000
2000G026	1/9/2000
2000G027	11/9/2000
1993G012	20/7/2001
1993G016	7/5/2001
1993G016	17/5/2001
1993G016	28/5/2001
1993G016	7/6/2001
1993G016	19/6/2001
1993G016	2/7/2001
1993G026	23/5/2001
1993G026	2/6/2001
1993G026	11/6/2001
1993G026	20/6/2001
1993G026	30/6/2001
1993G026	13/7/2001
1993G026	22/7/2001
1993G026	31/7/2001
1993G026	11/8/2001
1993G050	6/6/2001
1993G050	17/6/2001
1993G050	27/6/2001
1993G050	7/7/2001
1993G050	17/7/2001
1993G050	14/8/2001
1993G073	10/6/2001
1993G073	21/6/2001
1993G073	11/7/2001

1993G073	20/7/2001
1993G073	30/7/2001
1993G073	8/8/2001
1993G073	16/8/2001
1993G073	25/8/2001
1993G073	4/9/2001
1993G073	5/9/2001
1993G104	25/6/2001
1993G104	6/7/2001
1993G104	14/7/2001
1993G111	2/6/2001
1993G111	22/6/2001
1993G111	2/7/2001
1993G111	12/7/2001
1993G111	23/7/2001
1993G111	2/8/2001
1993G111	11/8/2001
1993G111	23/8/2001
1993G140	14/6/2001
1993G140	15/7/2001
1995G028	10/4/2001
1995G028	23/4/2001
1995G028	4/5/2001
1995G028	14/5/2001
1995G028	29/5/2001
1995G039	29/5/2001
1995G039	7/6/2001
1995G039	16/6/2001
1995G039	26/6/2001
1995G039	6/7/2001
1995G039	7/7/2001
1995G039	16/7/2001
1995G039	25/7/2001
1995G039	2/8/2001
1995G039	11/8/2001
1995G050	11/6/2001
1995G050	23/6/2001
1995G050	23/6/2001
1995G050	3/7/2001
1995G050	13/7/2001
1995G050	24/7/2001
1995G050	3/8/2001
1995G050	14/8/2001
1995G052	20/6/2001
1995G052	1/7/2001
1995G052	12/7/2001

1995G052	11/8/2001
1995G052	21/8/2001
1995G052	1/9/2001
1995G052	11/9/2001
1995G053	12/7/2001
1995G053	21/7/2001
1995G053	30/7/2001
1995G053	8/8/2001
1995G053	18/8/2001
1995G053	28/8/2001
1995G093	13/4/2001
1995G093	23/4/2001
1995G093	2/5/2001
1995G093	11/5/2001
1995G093	20/5/2001
1995G093	30/5/2001
1995G093	9/6/2001
1995G093	18/6/2001
1995G093	21/6/2001
1995G093	2/7/2001
1995G093	11/7/2001
1995G093	12/7/2001
1995G093	22/7/2001
1995G095	30/8/2001
1995G095	9/9/2001
1995G095	18/9/2001
1995G095	28/9/2001
1995G095	9/10/2001
1995G095	18/10/2001
1995G103	28/6/2001
1995G103	9/7/2001
1995G103	20/7/2001
1995G103	4/8/2001
1995G104	19/4/2001
1995G104	29/4/2001
1995G104	13/5/2001
1996G011	20/5/2001
1996G011	31/5/2001
1996G011	10/6/2001
1996G011	22/6/2001
1996G011	2/7/2001
1996G011	13/7/2001
1996G011	24/7/2001
1996G011	4/8/2001
1996G011	16/8/2001
1996G027	8/4/2001

1996G027	19/4/2001
1996G027	21/4/2001
1996G027	1/5/2001
1996G027	12/5/2001
1996G027	23/5/2001
1996G027	2/6/2001
1996G027	14/6/2001
1996G028	15/6/2001
1996G028	26/6/2001
1996G028	7/7/2001
1996G028	17/7/2001
1996G028	27/7/2001
1996G028	6/8/2001
1996G028	15/8/2001
1996G028	26/8/2001
1996G033	27/5/2001
1996G033	10/7/2001
1996G034	14/7/2001
1996G034	25/7/2001
1996G034	4/8/2001
1996G034	6/8/2001
1996G034	16/8/2001
1996G034	26/8/2001
1996G034	5/9/2001
1996G034	15/9/2001
1996G034	25/9/2001
1996G040	16/7/2001
1996G040	28/7/2001
1996G040	7/8/2001
1996G040	24/8/2001
1996G040	3/9/2001
1996G043	19/9/2001
1996G043	29/9/2001
1996G043	9/10/2001
1996G043	18/10/2001
1997G003	17/8/2001
1997G003	19/8/2001
1997G003	28/8/2001
1997G003	7/9/2001
1997G003	8/9/2001
1997G003	9/9/2001
1997G003	19/9/2001
1997G003	27/9/2001
1997G003	7/10/2001
1997G003	8/10/2001
1997G003	17/10/2001

1997G015	19/4/2001
1997G015	29/4/2001
1997G015	9/5/2001
1997G015	19/5/2001
1997G015	31/5/2001
1997G037	26/4/2001
1997G037	7/5/2001
1997G037	8/5/2001
1997G037	17/5/2001
1997G037	26/5/2001
1997G037	5/6/2001
1997G037	13/6/2001
1997G040	19/7/2001
1997G040	29/7/2001
1997G040	30/7/2001
1997G040	18/8/2001
1997G040	27/8/2001
1997G040	6/9/2001
1997G055	25/8/2001
1997G055	5/9/2001
1997G055	17/9/2001
1997G055	15/9/2001
1997G055	27/9/2001
1997G055	8/10/2001
1997G055	9/10/2001
1998G011	4/4/2001
1998G011	15/4/2001
1998G011	25/4/2001
1998G011	6/5/2001
1998G011	16/5/2001
1998G011	27/5/2001
1998G011	28/5/2001
1998G011	9/6/2001
1998G015	28/4/2001
1998G015	9/5/2001
1998G015	18/5/2001
1998G015	28/5/2001
1998G015	6/6/2001
1998G015	16/6/2001
1998G015	26/6/2001
1998G015	27/6/2001
1998G018	1/5/2001
1998G018	11/5/2001
1998G018	21/5/2001
1998G018	31/5/2001
1998G018	9/6/2001

1998G018	19/6/2001
1998G018	29/6/2001
1998G020	28/5/2001
1998G020	8/6/2001
1998G020	17/6/2001
1998G020	27/6/2001
1998G020	8/7/2001
1998G020	20/7/2001
1998G020	29/7/2001
1998G020	31/7/2001
1998G041	23/7/2001
1998G041	2/8/2001
1998G041	12/8/2001
1998G041	22/8/2001
1998G041	2/9/2001
1998G041	11/9/2001
1998G041	20/9/2001
2001G001	7/4/2001
2001G001	17/4/2001
2001G001	26/4/2001
2001G001	6/5/2001
2001G001	15/5/2001
2001G001	25/5/2001
2001G001	4/6/2001
2001G001	15/6/2001
2001G002	9/4/2001
2001G002	18/4/2001
2001G002	19/4/2001
2001G003	9/4/2001
2001G003	22/5/2001
2001G004	10/4/2001
2001G004	20/4/2001
2001G004	2/5/2001
2001G004	12/5/2001
2001G004	3/6/2001
2001G004	15/6/2001
2001G005	13/4/2001
2001G005	24/4/2001
2001G005	6/5/2001
2001G005	3/6/2001
2001G006	13/4/2001
2001G006	22/4/2001
2001G006	2/5/2001
2001G006	11/5/2001
2001G006	20/5/2001
2001G006	2/6/2001

2001G007	17/4/2001
2001G007	28/4/2001
2001G007	8/5/2001
2001G007	19/5/2001
2001G007	29/5/2001
2001G007	8/6/2001
2001G007	19/6/2001
2001G008	22/4/2001
2001G008	4/5/2001
2001G008	14/5/2001
2001G008	24/5/2001
2001G008	4/6/2001
2001G008	13/6/2001
2001G008	24/6/2001
2001G008	12/7/2001
2001G009	25/4/2001
2001G009	6/5/2001
2001G009	17/5/2001
2001G009	27/5/2001
2001G009	6/6/2001
2001G010	25/4/2001
2001G010	8/5/2001
2001G010	20/5/2001
2001G010	2/6/2001
2001G010	14/6/2001
2001G010	26/6/2001
2001G010	8/7/2001
2001G010	20/7/2001
2001G010	2/8/2001
2001G011	12/5/2001
2001G011	22/5/2001
2001G011	2/6/2001
2001G011	12/6/2001
2001G011	22/6/2001
2001G011	3/7/2001
2001G011	14/7/2001
2001G012	16/5/2001
2001G012	27/5/2001
2001G012	7/6/2001
2001G012	18/6/2001
2001G012	19/6/2001
2001G012	29/6/2001
2001G012	14/7/2001
2001G012	23/7/2001
2001G013	16/5/2001
2001G013	30/5/2001

2001G013	11/6/2001
2001G013	23/6/2001
2001G013	5/7/2001
2001G013	17/7/2001
2001G014	21/5/2001
2001G014	3/6/2001
2001G014	14/6/2001
2001G014	25/6/2001
2001G014	6/7/2001
2001G014	7/7/2001
2001G014	17/7/2001
2001G014	30/7/2001
2001G015	21/5/2001
2001G015	31/5/2001
2001G015	10/6/2001
2001G015	11/6/2001
2001G015	22/6/2001
2001G015	3/7/2001
2001G015	25/7/2001
2001G016	22/5/2001
2001G016	1/6/2001
2001G016	11/6/2001
2001G016	1/7/2001
2001G016	12/7/2001
2001G017	26/5/2001
2001G017	6/6/2001
2001G017	21/6/2001
2001G017	2/7/2001
2001G017	13/7/2001
2001G017	24/7/2001
2001G017	2/8/2001
2001G017	12/8/2001
2001G017	22/8/2001
2001G017	2/9/2001
2001G018	29/5/2001
2001G019	31/5/2001
2001G019	19/6/2001
2001G019	28/6/2001
2001G019	8/7/2001
2001G019	17/7/2001
2001G019	26/7/2001
2001G019	27/7/2001
2001G019	5/8/2001
2001G019	15/8/2001
2001G020	31/5/2001
2001G020	14/6/2001

2001G021	4/6/2001
2001G021	15/6/2001
2001G021	26/6/2001
2001G021	29/6/2001
2001G021	12/7/2001
2001G021	23/7/2001
2001G021	3/8/2001
2001G022	4/6/2001
2001G022	14/6/2001
2001G022	25/6/2001
2001G022	6/7/2001
2001G022	16/7/2001
2001G022	26/7/2001
2001G022	6/8/2001
2001G023	6/6/2001
2001G023	16/6/2001
2001G023	27/6/2001
2001G023	7/7/2001
2001G023	17/7/2001
2001G023	28/7/2001
2001G024	7/6/2001
2001G024	18/6/2001
2001G024	30/6/2001
2001G024	13/7/2001
2001G024	24/7/2001
2001G024	4/8/2001
2001G024	15/8/2001
2001G024	25/8/2001
2001G025	22/6/2001
2001G025	4/7/2001
2001G025	15/7/2001
2001G025	16/7/2001
2001G025	27/7/2001
2001G025	7/8/2001
2001G025	17/8/2001
2001G025	29/8/2001
2001G025	9/9/2001
2001G025	20/9/2001
2001G026	22/6/2001
2001G026	4/7/2001
2001G027	27/6/2001
2001G027	7/7/2001
2001G027	17/7/2001
2001G027	27/7/2001
2001G027	28/7/2001
2001G027	6/8/2001

2001G027	15/8/2001
2001G027	27/8/2001
2001G027	9/9/2001
2001G028	10/7/2001
2001G028	20/7/2001
2001G028	30/7/2001
2001G028	10/8/2001
2001G029	4/7/2001
2001G029	25/7/2001
2001G029	9/8/2001
2001G030	12/7/2001
2001G030	14/8/2001
2001G031	18/7/2001
2001G031	30/7/2001
2001G031	31/7/2001
2001G031	11/8/2001
2001G031	21/8/2001
2001G031	1/9/2001
2001G031	11/9/2001
2001G031	21/9/2001
2001G031	3/10/2001
2001G032	21/7/2001
2001G032	30/7/2001
2001G032	9/8/2001
2001G032	18/8/2001
2001G032	5/9/2001
2001G032	15/9/2001
2001G032	24/9/2001
2001G033	29/7/2001
2001G033	9/8/2001
2001G033	18/8/2001
2001G033	27/8/2001
2001G033	5/9/2001
2001G034	31/7/2001
2001G034	11/8/2001
2001G034	22/8/2001
2001G034	3/9/2001
2001G035	5/8/2001
2001G035	17/8/2001
2001G035	27/8/2001
2001G035	8/9/2001
2001G035	18/9/2001
2001G035	28/9/2001
2001G035	30/9/2001
2001G035	12/10/2001
2001G036	5/8/2001

2001G036	18/8/2001
2001G036	29/8/2001
2001G036	10/9/2001
2001G036	20/9/2001
2001G036	1/10/2001
2001G037	9/8/2001
2001G037	3/9/2001
2001G038	11/8/2001
2001G038	10/9/2001
2001G038	22/9/2001
2001G038	23/9/2001
2001G039	25/8/2001
2001G039	4/9/2001
2001G039	14/9/2001
2001G039	24/9/2001
2001G039	7/10/2001
2001G039	17/10/2001
2001G040	26/8/2001
2001G041	30/8/2001
2001G042	6/9/2001
2001G042	18/9/2001
2001G042	29/9/2001
2001G043	13/9/2001
2001G043	25/9/2001
2001G043	8/10/2001
2001G044	16/9/2001
2001G044	5/10/2001
2001G044	14/10/2001
2001G045	22/9/2001
2001G045	13/10/2001
2001G046	30/9/2001
2001G047	7/7/2001
1993G028	28/4/2002
1993G028	8/5/2002
1993G028	19/5/2002
1993G028	30/5/2002
1993G028	8/6/2002
1993G028	17/6/2002
1993G028	28/6/2002
1993G028	11/7/2002
1993G057	8/5/2002
1993G057	19/5/2002
1993G057	29/5/2002
1993G057	7/6/2002
1993G057	17/6/2002
1993G057	27/6/2002

1993G057	6/7/2002
1993G057	16/7/2002
1993G057	26/7/2002
1993G057	5/8/2002
1993G057	16/8/2002
1993G103	1/6/2002
1993G103	12/6/2002
1993G103	21/6/2002
1993G103	30/6/2002
1993G103	10/7/2002
1993G103	21/7/2002
1993G103	30/7/2002
1993G103	9/8/2002
1993G103	20/8/2002
1993G105	26/5/2002
1993G105	6/6/2002
1993G105	16/6/2002
1993G105	26/6/2002
1993G105	6/7/2002
1993G105	16/7/2002
1993G105	26/7/2002
1993G105	5/8/2002
1993G105	15/8/2002
1993G112	29/5/2002
1993G112	9/6/2002
1993G112	19/6/2002
1993G112	29/6/2002
1993G112	9/7/2002
1993G112	20/7/2002
1993G112	29/7/2002
1993G112	7/8/2002
1993G112	17/8/2002
1993G122	10/6/2002
1993G122	21/6/2002
1993G122	1/7/2002
1993G122	12/7/2002
1993G122	3/8/2002
1993G122	15/8/2002
1993G122	26/8/2002
1993G123	19/8/2002
1993G123	30/8/2002
1993G123	9/9/2002
1993G123	19/9/2002
1994G006	21/5/2002
1994G006	2/6/2002
1994G006	12/6/2002

1994G006	22/6/2002
1994G006	1/7/2002
1994G006	11/7/2002
1994G006	21/7/2002
1994G040	7/4/2002
1994G040	17/4/2002
1994G040	26/4/2002
1994G040	16/5/2002
1994G040	26/5/2002
1994G040	4/6/2002
1994G040	13/6/2002
1994G060	15/4/2002
1994G060	26/4/2002
1994G060	7/5/2002
1994G060	18/5/2002
1994G060	29/5/2002
1994G060	8/6/2002
1994G060	18/6/2002
1994G060	29/6/2002
1995G020	7/5/2002
1995G020	18/5/2002
1995G020	28/5/2002
1995G020	7/6/2002
1995G020	17/6/2002
1995G020	28/6/2002
1995G020	8/7/2002
1995G029	1/4/2002
1995G029	2/4/2002
1995G029	14/4/2002
1995G029	14/4/2002
1995G029	27/4/2002
1995G029	9/5/2002
1995G040	29/5/2002
1995G040	8/6/2002
1995G040	18/6/2002
1995G040	28/6/2002
1995G040	7/7/2002
1995G040	17/7/2002
1995G041	10/6/2002
1995G041	20/6/2002
1995G041	1/7/2002
1995G041	11/7/2002
1995G041	22/7/2002
1995G041	2/8/2002
1996G009	19/5/2002
1996G009	28/5/2002

1996G009	6/6/2002
1996G009	17/6/2002
1996G009	27/6/2002
1996G009	6/7/2002
1996G009	16/7/2002
1996G009	26/7/2002
1996G009	5/8/2002
1996G021	19/6/2002
1996G021	29/6/2002
1996G021	10/7/2002
1996G021	21/7/2002
1996G038	26/5/2002
1996G038	5/6/2002
1996G038	15/6/2002
1996G038	24/6/2002
1996G038	12/7/2002
1996G038	21/7/2002
1996G038	31/7/2002
1996G038	10/8/2002
1996G045	12/4/2002
1996G045	22/4/2002
1996G045	2/5/2002
1996G045	12/5/2002
1996G045	31/5/2002
1996G045	9/6/2002
1996G045	18/6/2002
1996G045	28/6/2002
1997G008	28/6/2002
1997G008	7/7/2002
1997G008	18/7/2002
1997G008	28/7/2002
1997G008	7/8/2002
1997G008	16/8/2002
1997G008	26/8/2002
1997G031	6/6/2002
1997G031	17/6/2002
1997G031	27/6/2002
1997G031	6/7/2002
1997G031	16/7/2002
1997G031	25/7/2002
1997G031	3/8/2002
1998G004	11/4/2002
1998G004	22/4/2002
1998G004	2/5/2002
1998G004	13/5/2002
1998G004	23/5/2002

1998G004	3/6/2002
1998G004	14/6/2002
1998G007	14/4/2002
1998G007	26/4/2002
1998G007	6/5/2002
1998G007	18/5/2002
1998G007	28/5/2002
1998G007	9/6/2002
1998G034	20/6/2002
1998G043	3/6/2002
1998G043	14/6/2002
1999G002	9/4/2002
1999G002	20/4/2002
1999G002	1/5/2002
1999G002	13/5/2002
1999G002	24/5/2002
1999G002	5/6/2002
1999G002	22/6/2002
1999G003	7/4/2002
1999G003	18/4/2002
1999G003	29/4/2002
1999G003	10/5/2002
1999G003	21/5/2002
1999G003	1/6/2002
1999G003	12/6/2002
1999G005	4/4/2002
1999G005	14/4/2002
1999G005	23/4/2002
1999G005	2/5/2002
1999G005	12/5/2002
1999G008	6/4/2002
1999G008	17/4/2002
1999G008	30/4/2002
1999G008	10/5/2002
1999G008	21/5/2002
1999G008	1/6/2002
1999G008	11/6/2002
1999G009	12/4/2002
1999G009	23/4/2002
1999G009	3/5/2002
1999G009	13/5/2002
1999G009	23/5/2002
1999G014	8/4/2002
1999G014	19/4/2002
1999G014	29/4/2002
1999G014	9/5/2002

1999G014	19/5/2002
1999G014	28/5/2002
1999G014	7/6/2002
1999G014	17/6/2002
1999G014	27/6/2002
1999G014	7/7/2002
1999G016	27/5/2002
1999G016	7/6/2002
1999G016	18/6/2002
1999G016	8/7/2002
1999G016	20/7/2002
1999G019	6/5/2002
1999G019	17/5/2002
1999G019	28/5/2002
1999G019	7/6/2002
1999G019	17/6/2002
1999G019	28/6/2002
1999G019	8/7/2002
1999G024	15/5/2002
1999G024	25/5/2002
1999G024	4/6/2002
1999G024	14/6/2002
1999G024	25/6/2002
1999G024	4/7/2002
1999G024	13/7/2002
1999G031	30/4/2002
1999G031	12/5/2002
1999G031	23/5/2002
1999G031	2/6/2002
1999G031	13/6/2002
1999G031	23/6/2002
1999G031	3/7/2002
1999G031	13/7/2002
1999G031	25/7/2002
1999G035	27/6/2002
1999G035	8/7/2002
1999G035	18/7/2002
1999G035	28/7/2002
1999G035	6/8/2002
1999G035	16/8/2002
1999G035	25/8/2002
1999G040	13/5/2002
1999G040	26/5/2002
1999G040	5/6/2002
1999G040	15/6/2002
1999G040	26/6/2002

1999G040	6/7/2002
1999G040	16/7/2002
1999G040	26/7/2002
1999G055	23/8/2002
2000G016	2/6/2002
2000G016	12/6/2002
2000G016	21/6/2002
2000G016	29/6/2002
2000G016	7/7/2002
2000G016	16/7/2002
2000G016	25/7/2002
2002G001	1/4/2002
2002G001	12/4/2002
2002G002	3/4/2002
2002G002	15/4/2002
2002G002	25/4/2002
2002G002	5/5/2002
2002G002	16/5/2002
2002G002	27/5/2002
2002G003	4/4/2002
2002G003	14/4/2002
2002G003	24/4/2002
2002G003	3/5/2002
2002G003	13/5/2002
2002G003	25/5/2002
2002G004	4/4/2002
2002G004	15/4/2002
2002G004	26/4/2002
2002G004	7/5/2002
2002G004	18/5/2002
2002G004	29/5/2002
2002G005	4/4/2002
2002G005	14/4/2002
2002G005	24/4/2002
2002G005	10/5/2002
2002G006	12/4/2002
2002G006	22/4/2002
2002G006	2/5/2002
2002G006	12/5/2002
2002G006	22/5/2002
2002G006	1/6/2002
2002G006	11/6/2002
2002G006	22/6/2002
2002G006	12/7/2002
2002G007	12/4/2002
2002G007	25/4/2002

2002G007	4/5/2002
2002G007	14/5/2002
2002G007	26/5/2002
2002G008	12/4/2002
2002G008	24/4/2002
2002G008	5/5/2002
2002G008	18/5/2002
2002G008	29/5/2002
2002G009	12/4/2002
2002G010	21/4/2002
2002G010	2/5/2002
2002G010	12/5/2002
2002G010	22/5/2002
2002G010	10/6/2002
2002G011	4/5/2002
2002G011	14/5/2002
2002G011	25/5/2002
2002G011	5/6/2002
2002G012	11/5/2002
2002G013	16/5/2002
2002G013	26/5/2002
2002G013	15/6/2002
2002G013	26/6/2002
2002G013	6/7/2002
2002G013	17/7/2002
2002G013	29/7/2002
2002G014	26/5/2002
2002G014	16/6/2002
2002G014	28/6/2002
2002G015	28/5/2002
2002G015	7/6/2002
2002G015	17/6/2002
2002G015	27/6/2002
2002G015	7/7/2002
2002G015	18/7/2002
2002G015	28/7/2002
2002G015	7/8/2002
2002G015	17/8/2002
2002G016	29/5/2002
2002G016	10/6/2002
2002G016	24/6/2002
2002G016	5/7/2002
2002G016	16/7/2002
2002G016	27/7/2002
2002G017	31/5/2002
2002G017	10/6/2002

2002G017	20/6/2002
2002G017	30/6/2002
2002G017	10/7/2002
2002G017	21/7/2002
2002G017	30/7/2002
2002G017	11/8/2002
2002G018	7/6/2002
2002G018	17/6/2002
2002G018	27/6/2002
2002G018	6/7/2002
2002G018	16/7/2002
2002G018	5/8/2002
2002G018	15/8/2002
2002G019	14/6/2002
2002G019	27/6/2002
2002G019	9/7/2002
2002G019	20/7/2002
2002G019	11/8/2002
2002G020	17/6/2002
2002G020	29/6/2002
2002G020	11/7/2002
2002G020	23/7/2002
2002G020	2/8/2002
2002G020	15/8/2002
2002G021	18/6/2002
2002G021	29/6/2002
2002G021	11/7/2002
2002G021	23/7/2002
2002G021	7/8/2002
2002G022	18/6/2002
2002G022	29/6/2002
2002G022	9/7/2002
2002G022	20/7/2002
2002G022	29/7/2002
2002G022	8/8/2002
2002G022	17/8/2002
2002G023	19/6/2002
2002G023	30/6/2002
2002G023	10/7/2002
2002G023	20/7/2002
2002G023	31/7/2002
2002G023	10/8/2002
2002G023	21/8/2002
2002G024	3/7/2002
2002G024	26/7/2002
2002G024	6/8/2002

2002G024	15/8/2002
2002G024	26/8/2002
2002G024	5/9/2002
2002G024	18/9/2002
2002G025	7/7/2002
2002G026	11/7/2002
2002G026	21/7/2002
2002G027	12/7/2002
2002G027	26/7/2002
2002G027	6/8/2002
2002G027	17/8/2002
2002G028	23/7/2002
2002G029	26/7/2002
2002G030	8/7/2002
2002G030	27/8/2002
2002G030	7/9/2002
2002G031	29/7/2002
2002G031	9/8/2002
2002G031	23/8/2002
2002G031	2/9/2002
2002G032	3/8/2002
2002G032	15/8/2002
2002G033	5/8/2002
2002G033	16/8/2002
2002G033	27/8/2002
2002G033	7/9/2002
2002G034	7/8/2002
2002G034	19/8/2002
2002G035	9/8/2002
2002G035	23/8/2002
2002G035	4/9/2002
2002G036	10/8/2002
1993G041	18/7/2003
1993G041	28/7/2003
1993G041	7/8/2003
1993G041	28/8/2003
1993G041	17/7/2003
1993G041	18/8/2003
1993G087	30/8/2003
1993G087	19/7/2003
1993G087	10/9/2003
1993G089	3/9/2003
1993G089	24/8/2003
1993G089	26/9/2003
1993G089	7/10/2003
1993G111	27/7/2003

1993G111	7/8/2003
1993G111	18/8/2003
1993G111	28/8/2003
1993G111	28/8/2003
1993G111	7/9/2003
1993G111	8/9/2003
1993G117	11/5/2003
1993G117	22/5/2003
1993G117	31/5/2003
1993G117	11/6/2003
1993G117	20/6/2003
1993G117	30/6/2003
1993G117	10/7/2003
1993G119	14/7/2003
1993G119	23/7/2003
1993G119	2/8/2003
1993G119	31/8/2003
1993G119	12/8/2003
1993G119	30/8/2003
1993G119	10/9/2003
1994G016	8/4/2003
1994G016	19/4/2003
1994G016	28/4/2003
1994G016	8/5/2003
1994G016	29/5/2003
1994G063	30/4/2003
1994G063	10/5/2003
1994G063	21/5/2003
1994G063	31/5/2003
1994G063	1/7/2003
1994G063	12/7/2003
1994G063	10/6/2003
1994G063	20/6/2003
1995G024	25/4/2003
1995G024	6/5/2003
1995G024	18/5/2003
1995G024	28/5/2003
1995G024	9/6/2003
1995G024	19/6/2003
1995G024	29/6/2003
1995G024	9/7/2003
1995G024	19/7/2003
1995G067	31/7/2003
1995G067	12/8/2003
1995G067	21/8/2003
1995G067	3/9/2003

1995G067	14/9/2003
1995G067	13/10/2003
1995G071	4/7/2003
1995G071	16/7/2003
1995G071	17/8/2003
1995G071	27/8/2003
1995G071	26/7/2003
1995G071	5/8/2003
1995G071	6/8/2003
1995G071	5/9/2003
1995G080	16/8/2003
1995G080	5/8/2003
1995G080	26/8/2003
1995G080	4/9/2003
1996G059	5/8/2003
1996G059	16/8/2003
1996G059	23/7/2003
1996G059	4/8/2003
1996G059	26/8/2003
1996G059	19/9/2003
1997G025	28/7/2003
1997G025	11/8/2003
1997G025	21/8/2003
1997G025	31/8/2003
1997G025	10/8/2003
1997G025	24/9/2003
1998G005	2/6/2003
1998G005	13/6/2003
1998G005	22/6/2003
1998G005	13/7/2003
1998G005	23/5/2003
1998G005	12/6/2003
1998G005	2/7/2003
1998G030	15/6/2003
1998G030	26/6/2003
1998G030	6/7/2003
1998G030	16/7/2003
1998G030	7/8/2003
1998G030	28/7/2003
1998G030	27/8/2003
1998G030	7/9/2003
1998G035	14/8/2003
1998G035	3/8/2003
1998G035	5/9/2003
1998G035	14/9/2003
1998G035	27/9/2003

1999G001	9/5/2003
1999G032	18/5/2003
1999G032	28/5/2003
1999G032	7/6/2003
1999G032	17/6/2003
1999G032	6/7/2003
1999G032	26/6/2003
1999G032	29/6/2003
1999G033	22/6/2003
1999G033	2/7/2003
1999G033	14/7/2003
1999G033	25/7/2003
1999G033	13/7/2003
2000G010	3/6/2003
2000G010	13/6/2003
2000G010	22/6/2003
2000G010	2/7/2003
2000G010	13/7/2003
2000G010	22/7/2003
2000G010	2/8/2003
2000G015	16/7/2003
2000G015	25/7/2003
2000G015	15/8/2003
2000G024	3/8/2003
2000G024	14/8/2003
2000G024	24/8/2003
2000G024	3/9/2003
2000G024	13/9/2003
2000G024	23/9/2003
2000G024	13/10/2003
2001G013	10/7/2003
2001G013	2/8/2003
2001G013	14/8/2003
2001G013	27/8/2003
2001G013	21/7/2003
2001G013	2/8/2003
2001G013	7/9/2003
2001G013	19/9/2003
2001G013	15/10/2003
2001G014	19/7/2003
2001G014	31/7/2003
2001G014	2/9/2003
2001G014	11/8/2003
2001G014	12/9/2003
2001G043	3/8/2003
2001G043	17/8/2003

2001G043	27/8/2003
2001G043	7/9/2003
2003G001	11/4/2003
2003G001	22/4/2003
2003G001	3/5/2003
2003G001	16/5/2003
2003G001	7/6/2003
2003G001	17/6/2003
2003G001	29/6/2003
2003G001	29/4/2003
2003G001	17/7/2003
2003G001	27/5/2003
2003G001	28/6/2003
2003G002	19/4/2003
2003G002	30/4/2003
2003G002	11/5/2003
2003G002	3/6/2003
2003G002	15/6/2003
2003G002	23/5/2003
2003G003	22/4/2003
2003G003	1/5/2003
2003G004	15/5/2003
2003G004	26/5/2003
2003G004	6/6/2003
2003G004	16/6/2003
2003G004	26/6/2003
2003G004	6/7/2003
2003G004	16/7/2003
2003G004	26/7/2003
2003G004	5/8/2003
2003G004	15/8/2003
2003G004	25/8/2003
2003G004	25/7/2003
2003G005	15/5/2003
2003G005	25/5/2003
2003G005	21/6/2003
2003G005	3/6/2003
2003G005	12/6/2003
2003G005	2/7/2003
2003G006	22/5/2003
2003G006	1/6/2003
2003G006	22/6/2003
2003G006	4/7/2003
2003G006	16/7/2003
2003G006	26/7/2003
2003G006	18/8/2003

2003G006	13/6/2003
2003G006	7/7/2003
2003G007	2/6/2003
2003G007	11/6/2003
2003G008	8/6/2003
2003G008	18/6/2003
2003G008	27/6/2003
2003G008	7/7/2003
2003G008	18/7/2003
2003G009	5/7/2003
2003G009	17/7/2003
2003G009	30/7/2003
2003G010	6/7/2003
2003G010	20/7/2003
2003G010	2/8/2003
2003G010	18/7/2003
2003G011	17/7/2003
2003G012	21/7/2003
2003G012	1/8/2003
2003G012	11/8/2003
2003G012	20/8/2003
2003G012	25/8/2003
2003G012	30/8/2003
2003G012	8/9/2003
2003G012	18/9/2003
2003G013	21/7/2003
2003G013	1/8/2003
2003G014	23/7/2003
2003G014	3/8/2003
2003G014	4/9/2003
2003G015	28/7/2003
2003G015	6/8/2003
2003G015	16/8/2003
2003G015	15/8/2003
2003G015	3/9/2003
2003G015	12/9/2003
2003G015	22/9/2003
2003G015	17/10/2003
2003G016	5/8/2003
2003G016	16/8/2003
2003G016	26/8/2003
2003G016	5/9/2003
2003G016	5/9/2003
2003G017	5/8/2003
2003G017	17/8/2003
2003G017	19/8/2003

2003G017	30/8/2003
2003G017	11/9/2003
2003G017	22/9/2003
2003G018	12/8/2003
2003G018	22/8/2003
2003G018	1/9/2003
2003G018	10/9/2003
2003G018	12/9/2003
2003G018	8/10/2003
2003G019	15/8/2003
2003G019	13/9/2003
2003G020	24/8/2003
2003G020	4/9/2003
2003G021	14/9/2003
1993G008	29/4/2004
1993G008	9/5/2004
1993G008	27/5/2004
1993G008	5/6/2004
1993G008	15/6/2004
1993G008	24/6/2004
1993G008	3/7/2004
1993G008	12/7/2004
1993G008	21/7/2004
1993G019	19/7/2004
1993G019	1/8/2004
1993G019	11/8/2004
1993G019	21/8/2004
1993G019	31/8/2004
1993G019	11/9/2004
1993G019	21/9/2004
1993G019	29/7/2004
1993G026	23/5/2004
1993G026	1/6/2004
1993G026	11/6/2004
1993G026	21/6/2004
1993G026	30/6/2004
1993G026	9/7/2004
1993G026	20/7/2004
1993G026	29/7/2004
1993G026	8/8/2004
1993G026	20/8/2004
1993G039	26/5/2004
1993G039	7/6/2004
1993G039	17/6/2004
1993G039	27/6/2004
1993G039	8/7/2004

1993G039	18/7/2004
1993G039	28/7/2004
1993G039	9/8/2004
1993G039	5/6/2004
1993G050	16/5/2004
1993G112	1/6/2004
1993G112	11/6/2004
1993G112	21/6/2004
1993G112	30/6/2004
1993G112	10/7/2004
1993G112	20/7/2004
1993G112	8/8/2004
1993G112	29/7/2004
1993G140	9/7/2004
1994G004	8/7/2004
1994G004	19/7/2004
1994G004	27/6/2004
1994G004	26/6/2004
1994G004	18/7/2004
1994G013	28/6/2004
1994G013	3/7/2004
1994G013	28/7/2004
1994G013	28/7/2004
1994G013	30/7/2004
1994G029	11/6/2004
1994G029	23/6/2004
1994G029	5/7/2004
1994G029	16/7/2004
1994G029	7/8/2004
1994G029	17/8/2004
1994G029	27/8/2004
1994G029	29/8/2004
1994G029	8/9/2004
1994G029	26/8/2004
1994G046	1/7/2004
1994G046	11/7/2004
1994G046	24/7/2004
1994G046	5/8/2004
1994G046	16/8/2004
1994G046	28/8/2004
1994G052	26/4/2004
1994G052	9/5/2004
1994G052	19/5/2004
1994G052	31/5/2004
1994G052	30/6/2004
1994G052	8/5/2004

1995G012	21/7/2004
1995G021	15/4/2004
1995G021	26/4/2004
1995G021	6/5/2004
1995G021	16/5/2004
1995G021	26/5/2004
1995G087	9/7/2004
1995G087	10/8/2004
1995G087	21/8/2004
1995G087	31/7/2004
1995G104	26/4/2004
1995G104	6/5/2004
1995G104	4/7/2004
1995G107	6/6/2004
1995G107	28/6/2004
1995G107	10/7/2004
1995G107	20/7/2004
1995G107	1/8/2004
1995G107	9/7/2004
1996G012	19/4/2004
1996G012	3/5/2004
1996G012	14/5/2004
1996G012	25/5/2004
1996G012	6/6/2004
1996G012	19/6/2004
1996G012	4/7/2004
1996G012	1/5/2004
1996G012	2/5/2004
1996G022	23/5/2004
1996G022	2/6/2004
1996G022	12/6/2004
1996G022	22/6/2004
1996G022	2/7/2004
1996G022	11/7/2004
1996G022	21/7/2004
1996G022	31/7/2004
1996G022	10/8/2004
1996G024	21/4/2004
1996G024	30/4/2004
1996G024	9/5/2004
1996G024	18/5/2004
1996G024	27/5/2004
1996G024	6/6/2004
1996G024	17/6/2004
1996G025	12/8/2004
1996G028	28/6/2004

1996G028	11/7/2004
1996G028	22/7/2004
1996G028	1/8/2004
1996G028	10/8/2004
1996G028	20/8/2004
1996G028	9/9/2004
1996G028	20/9/2004
1997G006	21/4/2004
1997G006	1/5/2004
1997G050	20/7/2004
1997G050	29/7/2004
1997G050	7/8/2004
1997G050	17/8/2004
1997G050	27/8/2004
1997G050	4/9/2004
1997G050	13/9/2004
1997G050	22/9/2004
1997G055	4/8/2004
1997G055	15/8/2004
1997G055	15/9/2004
1997G055	25/9/2004
1997G064	23/5/2004
1997G064	3/6/2004
1997G064	13/6/2004
1997G064	24/6/2004
1997G064	6/7/2004
1997G064	28/7/2004
1998G004	3/6/2004
1998G004	16/6/2004
1998G004	9/7/2004
1998G004	19/7/2004
1998G004	29/7/2004
1998G004	8/8/2004
1998G004	18/8/2004
1998G004	29/8/2004
1998G020	21/5/2004
1998G020	31/5/2004
1998G020	10/6/2004
1998G020	20/6/2004
1998G020	10/7/2004
1998G020	19/7/2004
1998G020	31/7/2004
1998G028	12/6/2004
1998G028	22/6/2004
1998G028	3/7/2004
1998G028	13/7/2004

1998G028	23/7/2004
1998G028	3/8/2004
1998G028	14/8/2004
1998G028	26/8/2004
1999G010	23/4/2004
1999G010	5/5/2004
1999G010	15/5/2004
1999G010	26/5/2004
1999G010	5/6/2004
1999G010	17/6/2004
1999G010	27/6/2004
1999G010	10/7/2004
1999G018	2/5/2004
1999G018	12/5/2004
1999G018	22/5/2004
1999G018	1/6/2004
1999G018	20/6/2004
1999G018	29/6/2004
1999G018	10/7/2004
1999G018	20/7/2004
1999G018	30/4/2004
1999G018	9/7/2004
1999G042	22/4/2004
1999G042	4/5/2004
1999G042	14/5/2004
1999G042	26/5/2004
1999G042	5/6/2004
1999G042	15/6/2004
1999G042	25/6/2004
1999G042	7/7/2004
1999G042	19/7/2004
1999G065	30/8/2004
1999G065	9/9/2004
1999G065	11/9/2004
1999G065	22/9/2004
2000G011	9/9/2004
2000G011	20/9/2004
2000G016	10/6/2004
2000G016	20/6/2004
2000G016	29/6/2004
2000G016	8/7/2004
2000G016	18/7/2004
2000G016	6/8/2004
2000G021	7/5/2004
2000G021	20/5/2004
2000G021	13/6/2004

2000G021	25/6/2004
2000G025	6/8/2004
2000G025	16/8/2004
2000G025	25/8/2004
2000G025	4/9/2004
2000G025	13/9/2004
2000G025	22/9/2004
2001G001	15/4/2004
2001G001	25/4/2004
2001G001	4/5/2004
2001G001	14/5/2004
2001G001	24/5/2004
2001G001	24/4/2004
2001G004	20/4/2004
2001G004	1/5/2004
2001G004	12/5/2004
2001G004	23/5/2004
2001G004	3/6/2004
2001G004	13/6/2004
2001G004	25/6/2004
2001G005	23/4/2004
2001G005	3/5/2004
2001G005	14/5/2004
2001G005	25/5/2004
2001G036	22/7/2004
2001G036	5/8/2004
2001G036	16/8/2004
2001G036	7/9/2004
2001G036	18/9/2004
2001G036	28/9/2004
2001G036	4/8/2004
2004G001	16/4/2004
2004G001	28/4/2004
2004G001	9/5/2004
2004G001	21/5/2004
2004G001	22/5/2004
2004G001	31/5/2004
2004G001	10/6/2004
2004G001	21/6/2004
2004G001	4/7/2004
2004G001	27/4/2004
2004G002	18/4/2004
2004G002	28/4/2004
2004G003	20/4/2004
2004G003	30/4/2004
2004G003	1/5/2004

2004G003	12/5/2004
2004G003	22/5/2004
2004G003	1/6/2004
2004G003	10/6/2004
2004G004	21/4/2004
2004G004	30/4/2004
2004G004	10/5/2004
2004G005	21/4/2004
2004G005	30/4/2004
2004G005	10/5/2004
2004G006	22/4/2004
2004G007	23/4/2004
2004G007	3/5/2004
2004G007	13/5/2004
2004G007	23/5/2004
2004G007	3/6/2004
2004G008	24/4/2004
2004G008	4/5/2004
2004G008	14/5/2004
2004G008	24/5/2004
2004G008	3/6/2004
2004G008	13/6/2004
2004G008	23/6/2004
2004G009	24/4/2004
2004G009	6/5/2004
2004G009	17/5/2004
2004G009	30/5/2004
2004G010	25/4/2004
2004G011	25/4/2004
2004G011	6/5/2004
2004G012	25/4/2004
2004G013	27/4/2004
2004G013	9/5/2004
2004G013	21/5/2004
2004G013	1/6/2004
2004G014	1/5/2004
2004G014	10/5/2004
2004G014	29/5/2004
2004G014	7/6/2004
2004G014	17/6/2004
2004G014	27/6/2004
2004G015	8/5/2004
2004G015	18/5/2004
2004G015	27/5/2004
2004G015	16/6/2004
2004G015	6/7/2004

2004G015	17/7/2004
2004G016	9/5/2004
2004G017	11/5/2004
2004G017	2/6/2004
2004G018	12/5/2004
2004G018	23/5/2004
2004G018	2/6/2004
2004G018	22/6/2004
2004G018	27/7/2004
2004G019	16/5/2004
2004G019	27/5/2004
2004G019	8/6/2004
2004G019	30/6/2004
2004G019	10/7/2004
2004G019	29/6/2004
2004G020	8/5/2004
2004G020	17/5/2004
2004G020	26/5/2004
2004G020	5/6/2004
2004G020	15/6/2004
2004G020	24/6/2004
2004G020	4/7/2004
2004G020	6/5/2004
2004G021	21/5/2004
2004G021	22/5/2004
2004G021	1/6/2004
2004G021	11/6/2004
2004G021	21/6/2004
2004G021	1/7/2004
2004G021	11/7/2004
2004G021	21/7/2004
2004G021	1/8/2004
2004G022	23/5/2004
2004G022	2/6/2004
2004G022	12/6/2004
2004G022	22/6/2004
2004G022	2/7/2004
2004G022	11/7/2004
2004G022	21/7/2004
2004G023	23/5/2004
2004G023	1/6/2004
2004G023	11/6/2004
2004G023	21/6/2004
2004G023	14/7/2004
2004G023	24/7/2004
2004G024	24/5/2004

2004G024	5/6/2004
2004G024	16/6/2004
2004G024	27/6/2004
2004G024	8/8/2004
2004G024	19/8/2004
2004G024	31/8/2004
2004G024	18/8/2004
2004G025	25/5/2004
2004G026	28/5/2004
2004G026	7/6/2004
2004G026	17/6/2004
2004G026	27/6/2004
2004G026	10/7/2004
2004G026	19/7/2004
2004G026	29/7/2004
2004G027	30/5/2004
2004G027	8/6/2004
2004G027	18/6/2004
2004G027	27/6/2004
2004G027	8/7/2004
2004G028	3/6/2004
2004G028	14/6/2004
2004G028	25/6/2004
2004G028	6/7/2004
2004G028	17/7/2004
2004G028	24/6/2004
2004G028	29/7/2004
2004G029	6/6/2004
2004G029	17/6/2004
2004G029	28/6/2004
2004G029	27/7/2004
2004G030	9/6/2004
2004G030	20/6/2004
2004G030	29/6/2004
2004G030	10/7/2004
2004G030	30/7/2004
2004G030	8/8/2004
2004G030	18/8/2004
2004G030	28/8/2004
2004G030	8/9/2004
2004G031	9/6/2004
2004G031	22/6/2004
2004G031	2/7/2004
2004G031	11/7/2004
2004G031	21/7/2004
2004G031	31/7/2004

2004G031	10/8/2004
2004G032	11/6/2004
2004G032	21/6/2004
2004G032	30/6/2004
2004G033	13/8/2004
2004G033	13/6/2004
2004G034	18/6/2004
2004G034	29/6/2004
2004G034	10/7/2004
2004G034	22/7/2004
2004G034	13/8/2004
2004G034	24/8/2004
2004G034	4/9/2004
2004G034	21/7/2004
2004G034	2/8/2004
2004G035	20/6/2004
2004G035	2/7/2004
2004G035	12/7/2004
2004G035	22/7/2004
2004G035	2/8/2004
2004G035	12/8/2004
2004G035	22/8/2004
2004G036	21/6/2004
2004G036	30/6/2004
2004G036	10/7/2004
2004G036	20/7/2004
2004G036	31/7/2004
2004G036	10/8/2004
2004G037	24/6/2004
2004G037	4/7/2004
2004G037	15/7/2004
2004G037	5/8/2004
2004G037	15/8/2004
2004G037	25/8/2004
2004G037	5/9/2004
2004G037	27/7/2004
2004G038	27/6/2004
2004G038	8/7/2004
2004G038	18/7/2004
2004G038	28/7/2004
2004G038	8/8/2004
2004G038	7/8/2004
2004G039	30/6/2004
2004G040	2/7/2004
2004G040	12/7/2004
2004G040	3/8/2004

2004G040	15/8/2004
2004G040	14/8/2004
2004G041	5/7/2004
2004G041	16/7/2004
2004G041	8/8/2004
2004G042	5/7/2004
2004G043	9/7/2004
2004G043	19/7/2004
2004G043	28/7/2004
2004G043	7/8/2004
2004G043	16/8/2004
2004G043	26/8/2004
2004G043	5/9/2004
2004G044	9/7/2004
2004G044	20/7/2004
2004G044	31/7/2004
2004G044	11/8/2004
2004G044	22/8/2004
2004G044	4/9/2004
2004G044	4/9/2004
2004G045	9/7/2004
2004G045	19/7/2004
2004G045	29/7/2004
2004G045	8/8/2004
2004G045	18/8/2004
2004G045	29/8/2004
2004G045	16/7/2004
2004G045	18/8/2004
2004G046	10/7/2004
2004G047	12/7/2004
2004G047	22/7/2004
2004G047	1/8/2004
2004G047	11/8/2004
2004G047	22/8/2004
2004G047	3/9/2004
2004G048	16/7/2004
2004G048	26/7/2004
2004G048	27/7/2004
2004G048	8/8/2004
2004G048	19/8/2004
2004G048	30/8/2004
2004G049	18/7/2004
2004G049	28/7/2004
2004G050	20/7/2004
2004G050	3/8/2004
2004G050	14/8/2004

2004G050	24/8/2004
2004G050	6/9/2004
2004G050	16/9/2004
2004G050	27/9/2004
2004G051	21/7/2004
2004G051	1/8/2004
2004G051	14/8/2004
2004G052	31/7/2004
2004G052	10/8/2004
2004G052	20/8/2004
2004G053	1/8/2004
2004G053	11/8/2004
2004G053	23/8/2004
2004G054	15/8/2004
2004G054	26/8/2004
2004G054	6/9/2004
2004G054	26/9/2004
2004G055	4/7/2004
2004G056	7/8/2004
2004G056	18/8/2004
2004G056	30/8/2004
2004G056	12/9/2004
2004G057	11/8/2004
2004G057	2/9/2004
2004G057	24/9/2004
2004G057	8/10/2004
2004G057	12/10/2004
2004G058	18/8/2004
2004G058	31/8/2004
2004G059	10/7/2004
2004G059	13/8/2004
2004G059	3/9/2004
2004G059	11/8/2004
2004G059	23/8/2004
2004G060	25/8/2004
2004G060	6/9/2004
2004G060	17/9/2004
2004G060	29/9/2004
1993G073	9/6/2005
1993G073	19/6/2005
1993G073	29/6/2005
1993G073	8/7/2005
1993G073	17/7/2005
1993G073	26/7/2005
1993G073	4/8/2005
1993G073	22/8/2005

1993G073	8/6/2005
1993G073	8/7/2005
1993G073	26/7/2005
1993G073	31/8/2005
1993G077	24/5/2005
1993G077	3/6/2005
1993G077	14/6/2005
1993G077	25/6/2005
1993G077	4/7/2005
1993G077	14/7/2005
1993G077	24/7/2005
1993G077	2/8/2005
1993G077	12/8/2005
1993G077	13/6/2005
1993G077	24/6/2005
1993G103	14/6/2005
1993G103	25/6/2005
1993G103	5/7/2005
1993G103	14/7/2005
1993G103	23/7/2005
1993G103	2/8/2005
1993G103	11/8/2005
1993G103	21/8/2005
1993G103	4/7/2005
1993G111	12/6/2005
1993G111	23/6/2005
1993G111	3/7/2005
1993G111	13/7/2005
1993G111	23/7/2005
1993G111	2/8/2005
1993G111	12/8/2005
1993G111	23/8/2005
1993G111	25/8/2005
1993G111	22/6/2005
1993G122	14/6/2005
1993G122	26/6/2005
1993G122	7/7/2005
1993G122	18/7/2005
1993G122	28/7/2005
1993G122	8/8/2005
1993G122	19/8/2005
1993G122	29/8/2005
1993G122	26/6/2005
1994G016	23/6/2005
1994G016	7/7/2005
1994G016	18/7/2005

1994G016	7/8/2005
1994G016	19/8/2005
1994G016	28/7/2005
1994G016	30/8/2005
1994G033	10/8/2005
1994G033	21/8/2005
1994G033	31/8/2005
1994G033	20/8/2005
1995G040	13/6/2005
1995G095	3/9/2005
1995G095	13/9/2005
1995G095	1/10/2005
1995G095	10/10/2005
1995G095	11/10/2005
1995G095	21/10/2005
1995G095	3/9/2005
1995G095	10/10/2005
1996G009	17/5/2005
1996G009	28/5/2005
1996G009	6/6/2005
1996G009	15/6/2005
1996G009	24/6/2005
1996G009	3/7/2005
1996G009	12/7/2005
1996G009	22/7/2005
1996G009	31/7/2005
1996G009	27/5/2005
1996G009	27/5/2005
1996G009	21/7/2005
1996G021	20/5/2005
1996G021	31/5/2005
1996G021	11/6/2005
1996G021	22/6/2005
1996G021	2/7/2005
1997G043	2/7/2005
1997G043	12/7/2005
1997G043	23/7/2005
1997G043	3/8/2005
1997G043	24/8/2005
1997G043	4/9/2005
1997G043	23/8/2005
1999G005	13/4/2005
1999G005	22/4/2005
1999G005	2/5/2005
1999G005	11/5/2005
1999G005	20/5/2005

1999G008	9/5/2005
1999G008	21/5/2005
1999G008	21/6/2005
1999G008	1/7/2005
1999G008	11/7/2005
1999G008	22/7/2005
1999G008	19/6/2005
1999G008	20/6/2005
1999G014	11/4/2005
1999G024	11/5/2005
1999G024	22/5/2005
1999G024	1/6/2005
1999G024	10/6/2005
1999G024	21/6/2005
1999G024	1/7/2005
1999G024	11/7/2005
1999G024	21/7/2005
1999G024	8/5/2005
1999G024	10/5/2005
1999G024	20/5/2005
1999G024	31/5/2005
1999G024	19/6/2005
1999G061	19/8/2005
1999G061	30/8/2005
1999G061	10/9/2005
1999G061	20/9/2005
1999G061	29/9/2005
1999G061	8/10/2005
1999G061	9/9/2005
1999G062	30/9/2005
1999G062	29/9/2005
1999G062	10/10/2005
2001G014	25/7/2005
2001G014	5/8/2005
2001G014	16/8/2005
2001G014	30/8/2005
2001G014	11/9/2005
2001G014	1/10/2005
2001G014	12/7/2005
2001G014	28/8/2005
2001G014	29/8/2005
2001G014	29/8/2005
2001G014	10/9/2005
2001G014	11/9/2005
2002G003	13/4/2005
2002G003	23/4/2005

2002G003	2/5/2005
2002G005	14/4/2005
2002G005	23/4/2005
2002G005	2/5/2005
2002G005	11/5/2005
2002G006	3/5/2005
2002G006	14/5/2005
2002G006	24/5/2005
2002G006	2/6/2005
2002G006	12/6/2005
2002G006	22/6/2005
2002G006	2/7/2005
2002G006	22/4/2005
2002G010	24/7/2005
2002G010	3/8/2005
2002G010	12/8/2005
2002G010	22/8/2005
2002G010	3/9/2005
2002G010	14/7/2005
2002G010	2/9/2005
2002G020	13/6/2005
2002G020	25/6/2005
2002G020	5/7/2005
2002G020	17/7/2005
2002G020	28/7/2005
2002G020	30/5/2005
2002G020	31/5/2005
2002G020	25/6/2005
2002G020	15/7/2005
2002G020	9/8/2005
2005G001	11/4/2005
2005G001	22/4/2005
2005G001	3/5/2005
2005G001	13/5/2005
2005G001	24/5/2005
2005G001	3/6/2005
2005G001	14/6/2005
2005G001	23/5/2005
2005G001	24/5/2005
2005G002	15/4/2005
2005G002	25/4/2005
2005G002	6/5/2005
2005G003	18/4/2005
2005G003	30/4/2005
2005G003	30/4/2005
2005G004	24/4/2005

2005G004	3/5/2005
2005G004	13/5/2005
2005G004	23/5/2005
2005G004	1/6/2005
2005G004	10/6/2005
2005G005	28/4/2005
2005G005	8/5/2005
2005G005	17/5/2005
2005G005	27/5/2005
2005G005	5/6/2005
2005G005	25/6/2005
2005G005	4/7/2005
2005G005	24/7/2005
2005G005	7/5/2005
2005G005	5/6/2005
2005G005	14/6/2005
2005G005	24/6/2005
2005G006	10/5/2005
2005G007	30/5/2005
2005G007	9/6/2005
2005G007	1/7/2005
2005G007	22/7/2005
2005G007	9/6/2005
2005G008	2/6/2005
2005G008	15/6/2005
2005G008	27/6/2005
2005G008	7/7/2005
2005G008	17/7/2005
2005G008	28/7/2005
2005G008	9/6/2005
2005G009	5/6/2005
2005G010	9/6/2005
2005G010	21/6/2005
2005G010	2/7/2005
2005G011	14/6/2005
2005G011	28/6/2005
2005G011	11/7/2005
2005G011	21/8/2005
2005G011	31/8/2005
2005G011	1/8/2005
2005G011	30/8/2005
2005G012	11/7/2005
2005G012	4/8/2005
2005G012	16/8/2005
2005G012	16/6/2005
2005G012	24/7/2005

2005G012	3/8/2005
2005G012	15/8/2005
2005G013	23/6/2005
2005G013	4/7/2005
2005G013	14/7/2005
2005G013	24/7/2005
2005G013	3/8/2005
2005G013	13/8/2005
2005G013	24/8/2005
2005G013	13/8/2005
2005G013	24/8/2005
2005G014	29/6/2005
2005G014	8/7/2005
2005G014	19/7/2005
2005G014	29/7/2005
2005G014	8/8/2005
2005G014	18/8/2005
2005G014	27/8/2005
2005G014	6/9/2005
2005G014	19/9/2005
2005G014	18/7/2005
2005G014	7/8/2005
2005G014	7/8/2005
2005G014	8/8/2005
2005G015	3/7/2005
2005G015	15/7/2005
2005G016	28/7/2005
2005G016	9/8/2005
2005G016	1/9/2005
2005G016	13/9/2005
2005G016	2/10/2005
2005G017	19/8/2005
2005G017	30/8/2005
2005G017	22/9/2005
2005G017	14/10/2005
2005G018	22/8/2005
2005G018	2/9/2005
2005G018	14/9/2005
2005G018	24/9/2005
2005G018	4/10/2005
2005G018	2/9/2005
2005G018	2/9/2005
2005G019	1/9/2005
2005G019	13/9/2005
2005G019	23/9/2005
2005G019	12/10/2005

2005G020	23/9/2005
2005G020	4/10/2005
2005G020	15/10/2005
2005G020	26/10/2005
2005G020	3/10/2005
2005G020	25/10/2005
1993G088	26/6/2006
1993G088	6/7/2006
1993G088	16/7/2006
1993G088	27/7/2006
1993G088	5/8/2006
1994G006	12/7/2006
1994G006	24/7/2006
1994G006	4/8/2006
1994G006	26/8/2006
1994G006	5/9/2006
1994G022	14/7/2006
1994G022	13/8/2006
1994G022	13/9/2006
1994G022	23/9/2006
1994G040	1/4/2006
1994G040	10/4/2006
1994G040	19/4/2006
1994G040	29/4/2006
1994G040	18/5/2006
1994G040	28/5/2006
1994G063	12/5/2006
1994G063	22/5/2006
1994G063	1/6/2006
1994G063	22/6/2006
1994G063	2/7/2006
1994G063	14/7/2006
1994G063	12/6/2006
1995G024	23/4/2006
1995G024	5/5/2006
1995G024	26/5/2006
1995G024	6/6/2006
1995G024	18/6/2006
1995G024	20/7/2006
1995G024	21/4/2006
1995G024	22/4/2006
1995G039	11/6/2006
1995G039	20/6/2006
1995G039	29/6/2006
1995G039	9/7/2006
1995G039	18/7/2006

1995G039	27/7/2006
1995G039	5/8/2006
1995G039	13/8/2006
1995G039	23/8/2006
1995G039	17/7/2006
1995G049	1/7/2006
1995G049	13/7/2006
1995G049	24/7/2006
1995G049	3/8/2006
1995G049	14/8/2006
1995G049	26/8/2006
1995G049	27/6/2006
1995G049	30/6/2006
1995G049	23/7/2006
1995G050	22/6/2006
1995G050	13/7/2006
1995G050	22/7/2006
1995G050	1/8/2006
1995G050	10/8/2006
1996G011	31/5/2006
1996G011	12/6/2006
1996G011	3/7/2006
1996G011	23/7/2006
1996G011	2/8/2006
1996G011	14/8/2006
1996G011	12/6/2006
1996G011	22/6/2006
1996G011	13/7/2006
1996G011	13/7/2006
1997G025	11/6/2006
1997G025	21/6/2006
1997G025	3/7/2006
1997G025	15/7/2006
1997G025	26/7/2006
1997G025	6/8/2006
1997G025	30/8/2006
1997G025	12/9/2006
1997G025	11/9/2006
1998G023	19/5/2006
1998G023	29/5/2006
1998G023	6/6/2006
1998G023	16/6/2006
1998G023	25/6/2006
1998G023	3/7/2006
1998G023	17/5/2006
1998G034	10/6/2006

1999G003	18/4/2006
1999G003	1/5/2006
1999G003	11/5/2006
1999G003	1/6/2006
1999G003	12/6/2006
1999G003	23/6/2006
1999G003	4/7/2006
1999G003	29/4/2006
1999G033	10/7/2006
1999G050	4/9/2006
1999G050	15/9/2006
1999G050	25/9/2006
1999G050	6/10/2006
1999G050	14/9/2006
1999G057	16/6/2006
1999G057	4/7/2006
1999G057	14/7/2006
2000G010	3/9/2006
2000G010	16/6/2006
2000G010	18/6/2006
2000G010	18/6/2006
2000G010	20/6/2006
2000G010	22/6/2006
2000G010	25/6/2006
2000G010	26/6/2006
2000G010	26/6/2006
2000G010	27/6/2006
2000G010	27/6/2006
2000G010	28/6/2006
2000G010	2/7/2006
2000G010	15/7/2006
2000G010	16/7/2006
2000G010	17/7/2006
2000G010	19/8/2006
2000G010	21/8/2006
2000G010	22/8/2006
2000G014	9/6/2006
2000G014	22/6/2006
2000G014	2/7/2006
2000G014	4/8/2006
2000G014	16/8/2006
2000G014	24/7/2006
2000G016	6/6/2006
2000G016	16/6/2006
2000G016	4/7/2006
2000G016	14/7/2006

2000G016	23/7/2006
2000G016	2/8/2006
2000G016	11/8/2006
2000G016	11/8/2006
2001G010	1/6/2006
2001G010	12/6/2006
2001G010	3/7/2006
2001G010	26/7/2006
2001G010	5/8/2006
2001G010	14/7/2006
2001G013	4/6/2006
2001G013	12/7/2006
2001G013	22/7/2006
2001G013	2/8/2006
2001G013	12/8/2006
2001G013	17/6/2006
2001G034	13/7/2006
2001G034	24/7/2006
2001G034	4/8/2006
2001G035	2/4/2006
2001G035	14/4/2006
2001G035	26/4/2006
2001G035	6/5/2006
2001G035	16/5/2006
2001G035	28/5/2006
2001G035	25/4/2006
2001G035	26/5/2006
2001G043	16/7/2006
2002G004	13/4/2006
2002G004	23/4/2006
2002G004	4/5/2006
2002G004	14/5/2006
2002G004	25/5/2006
2002G004	5/6/2006
2002G004	5/6/2006
2002G013	7/6/2006
2002G013	17/6/2006
2002G013	27/6/2006
2002G013	8/7/2006
2002G013	18/7/2006
2002G013	27/7/2006
2002G013	7/8/2006
2002G013	18/8/2006
2002G038	8/5/2006
2003G001	1/5/2006
2003G001	13/5/2006

2003G001	24/5/2006
2003G001	2/6/2006
2003G001	13/6/2006
2003G001	23/6/2006
2003G001	15/7/2006
2003G001	23/5/2006
2003G001	12/6/2006
2003G001	3/7/2006
2003G001	15/7/2006
2003G003	1/7/2006
2003G003	11/7/2006
2003G003	28/8/2006
2003G008	12/6/2006
2003G008	22/6/2006
2003G008	2/7/2006
2003G008	12/7/2006
2003G008	23/7/2006
2004G034	21/6/2006
2004G039	21/4/2006
2006G001	24/3/2006
2006G001	6/4/2006
2006G001	16/4/2006
2006G001	27/4/2006
2006G001	7/5/2006
2006G001	16/5/2006
2006G001	27/5/2006
2006G001	7/6/2006
2006G002	28/3/2006
2006G002	18/4/2006
2006G002	29/4/2006
2006G002	10/5/2006
2006G003	27/3/2006
2006G004	30/3/2006
2006G005	1/4/2006
2006G005	11/4/2006
2006G005	21/4/2006
2006G005	2/5/2006
2006G005	11/5/2006
2006G005	30/4/2006
2006G005	1/5/2006
2006G005	11/5/2006
2006G006	5/4/2006
2006G006	25/4/2006
2006G006	4/5/2006
2006G006	14/5/2006
2006G006	12/6/2006

2006G006	15/4/2006
2006G006	13/5/2006
2006G007	8/4/2006
2006G007	2/5/2006
2006G007	27/5/2006
2006G008	19/4/2006
2006G008	29/4/2006
2006G009	27/4/2006
2006G009	8/5/2006
2006G009	18/5/2006
2006G009	29/5/2006
2006G009	8/6/2006
2006G009	18/6/2006
2006G009	28/6/2006
2006G010	18/5/2006
2006G010	6/6/2006
2006G010	16/6/2006
2006G010	27/6/2006
2006G011	21/5/2006
2006G011	2/6/2006
2006G011	22/6/2006
2006G011	4/7/2006
2006G011	12/6/2006
2006G012	21/5/2006
2006G012	2/7/2006
2006G012	13/7/2006
2006G012	25/7/2006
2006G012	1/6/2006
2006G013	27/5/2006
2006G013	8/6/2006
2006G014	31/5/2006
2006G014	21/6/2006
2006G014	3/7/2006
2006G014	15/7/2006
2006G014	11/6/2006
2006G014	2/7/2006
2006G014	15/7/2006
2006G015	1/6/2006
2006G015	12/6/2006
2006G015	22/6/2006
2006G015	2/7/2006
2006G015	12/7/2006
2006G015	22/7/2006
2006G015	22/6/2006
2006G015	21/7/2006
2006G015	1/8/2006

2006G016	9/6/2006
2006G016	19/6/2006
2006G016	22/7/2006
2006G016	12/8/2006
2006G016	12/7/2006
2006G016	1/8/2006
2006G017	26/8/2006
2006G017	20/9/2006
2006G018	12/6/2006
2006G018	2/7/2006
2006G018	14/7/2006
2006G018	21/6/2006
2006G018	2/7/2006
2006G019	16/6/2006
2006G020	17/6/2006
2006G020	28/6/2006
2006G020	9/7/2006
2006G020	18/7/2006
2006G020	19/7/2006
2006G021	21/6/2006
2006G021	12/7/2006
2006G021	22/7/2006
2006G021	21/7/2006
2006G021	31/7/2006
2006G022	24/6/2006
2006G022	15/7/2006
2006G022	26/7/2006
2006G022	4/8/2006
2006G022	4/8/2006
2006G022	14/8/2006
2006G023	26/6/2006
2006G024	19/6/2006
2006G025	28/6/2006
2006G025	19/7/2006
2006G025	29/7/2006
2006G025	9/8/2006
2006G025	20/8/2006
2006G026	23/9/2006
2006G027	2/10/2006
2006G028	13/7/2006
2006G028	5/8/2006
2006G028	14/8/2006
2006G028	25/8/2006
2006G028	24/7/2006
2006G029	13/7/2006
2006G029	23/7/2006

2006G030	13/7/2006
2006G030	23/7/2006
2006G030	2/8/2006
2006G030	12/8/2006
2006G030	13/7/2006
2006G030	12/8/2006
2006G031	14/7/2006
2006G031	24/7/2006
2006G031	2/8/2006
2006G032	16/7/2006
2006G033	25/7/2006
2006G033	4/8/2006
2006G033	13/8/2006
2006G033	23/8/2006
2006G033	23/8/2006
2006G034	2/7/2006
2006G035	3/8/2006
2006G035	14/8/2006
2006G035	5/9/2006
2006G035	16/9/2006
2006G035	26/9/2006
2006G035	25/8/2006
2006G035	26/8/2006
2006G036	10/8/2006
2006G037	12/8/2006
2006G038	2/10/2006
2006G039	21/9/2006
2006G039	3/10/2006
2006G039	2/10/2006
2006G002	24/3/2006
1993G008	27/5/2007
1993G008	6/6/2007
1993G008	16/6/2007
1993G008	26/6/2007
1993G008	5/7/2007
1993G008	14/7/2007
1993G008	23/7/2007
1993G008	13/8/2007
1993G008	23/8/2007
1993G026	23/5/2007
1993G026	2/6/2007
1993G026	11/6/2007
1993G026	20/6/2007
1993G026	30/6/2007
1993G026	8/7/2007
1993G026	18/7/2007

1993G026	26/7/2007
1993G026	15/8/2007
1993G050	21/5/2007
1993G050	10/6/2007
1993G050	30/6/2007
1993G057	3/3/2007
1993G057	14/3/2007
1993G057	25/3/2007
1993G057	5/4/2007
1993G057	15/4/2007
1993G057	25/4/2007
1993G057	5/5/2007
1993G057	16/5/2007
1993G057	26/5/2007
1993G071	10/6/2007
1993G071	20/6/2007
1993G071	2/7/2007
1993G071	11/7/2007
1993G071	21/7/2007
1993G071	31/7/2007
1993G071	11/8/2007
1993G071	21/8/2007
1993G071	1/9/2007
1993G071	11/9/2007
1993G078	6/7/2007
1993G078	16/7/2007
1993G078	25/7/2007
1993G078	3/8/2007
1993G078	13/8/2007
1993G078	24/8/2007
1993G078	4/9/2007
1993G112	19/6/2007
1993G140	29/7/2007
1993G140	17/8/2007
1994G060	29/5/2007
1994G060	9/6/2007
1994G060	20/6/2007
1994G060	1/7/2007
1994G060	10/7/2007
1994G060	21/7/2007
1994G060	5/8/2007
1995G021	7/5/2007
1995G021	18/5/2007
1995G021	29/5/2007
1995G021	8/6/2007
1995G021	17/6/2007

1995G021	27/6/2007
1995G021	7/7/2007
1995G021	18/7/2007
1995G023	11/5/2007
1995G023	21/5/2007
1995G023	30/5/2007
1995G023	8/6/2007
1995G023	17/6/2007
1995G023	6/7/2007
1998G004	30/5/2007
1998G004	10/6/2007
1998G004	20/6/2007
1998G004	2/7/2007
1998G004	11/7/2007
1998G004	1/8/2007
1998G004	11/8/2007
1998G004	22/8/2007
1998G020	13/5/2007
1998G020	23/5/2007
1998G020	1/6/2007
1998G020	10/6/2007
1998G020	19/6/2007
1998G020	30/6/2007
1998G020	8/7/2007
1998G020	18/7/2007
2000G019	23/4/2007
2000G019	5/5/2007
2000G019	15/5/2007
2000G019	4/6/2007
2000G019	14/6/2007
2000G019	26/6/2007
2000G019	8/7/2007
2000G020	13/7/2007
2000G021	5/5/2007
2000G021	17/5/2007
2000G021	28/5/2007
2000G021	7/6/2007
2000G021	30/6/2007
2000G024	18/6/2007
2000G024	30/6/2007
2000G024	9/7/2007
2000G024	19/7/2007
2000G024	28/7/2007
2000G024	7/8/2007
2000G024	17/8/2007
2000G024	27/8/2007

2000G025	26/8/2007
2000G025	5/9/2007
2000G025	14/9/2007
2000G025	23/9/2007
2000G025	3/10/2007
2000G025	23/10/2007
2000G026	20/6/2007
2000G026	2/7/2007
2000G026	11/7/2007
2000G026	22/7/2007
2000G026	3/8/2007
2001G001	26/4/2007
2001G001	6/5/2007
2001G001	16/5/2007
2001G001	26/5/2007
2001G001	4/6/2007
2001G001	13/6/2007
2001G001	23/6/2007
2001G001	2/7/2007
2001G001	11/7/2007
2001G005	12/3/2007
2001G005	25/3/2007
2001G005	17/4/2007
2001G005	27/4/2007
2001G005	8/5/2007
2001G005	21/5/2007
2001G005	1/6/2007
2002G024	9/8/2007
2002G024	20/8/2007
2002G024	30/8/2007
2002G024	9/9/2007
2002G024	23/9/2007
2002G024	4/10/2007
2002G024	13/10/2007
2002G024	25/10/2007
2002G026	22/4/2007
2002G026	2/5/2007
2002G026	22/5/2007
2002G030	9/8/2007
2002G030	19/8/2007
2002G030	31/8/2007
2002G030	9/9/2007
2002G032	20/7/2007
2004G007	8/3/2007
2004G007	22/3/2007
2004G007	5/4/2007

2004G007	17/4/2007
2004G007	30/4/2007
2004G007	12/5/2007
2004G007	5/6/2007
2004G015	9/5/2007
2004G015	19/5/2007
2004G015	30/5/2007
2004G015	9/6/2007
2004G015	19/6/2007
2004G015	28/6/2007
2004G015	7/7/2007
2004G015	18/7/2007
2004G051	18/6/2007
2004G051	8/7/2007
2004G051	18/7/2007
2004G051	31/7/2007
2004G051	8/9/2007
2007G001	10/3/2007
2007G001	22/3/2007
2007G002	27/3/2007
2007G002	19/4/2007
2007G002	1/5/2007
2007G002	11/5/2007
2007G002	22/5/2007
2007G003	2/4/2007
2007G003	13/4/2007
2007G003	23/4/2007
2007G003	12/5/2007
2007G003	22/5/2007
2007G003	1/6/2007
2007G003	11/6/2007
2007G004	5/4/2007
2007G004	15/4/2007
2007G004	26/4/2007
2007G004	7/5/2007
2007G004	17/5/2007
2007G004	27/5/2007
2007G004	6/6/2007
2007G005	10/4/2007
2007G005	21/4/2007
2007G005	1/5/2007
2007G005	10/5/2007
2007G005	19/5/2007
2007G005	28/5/2007
2007G005	7/6/2007
2007G006	10/4/2007

2007G007	21/4/2007
2007G007	11/5/2007
2007G007	22/5/2007
2007G007	1/6/2007
2007G007	11/6/2007
2007G007	21/6/2007
2007G008	23/4/2007
2007G009	23/4/2007
2007G009	5/5/2007
2007G009	15/5/2007
2007G009	28/5/2007
2007G009	7/6/2007
2007G009	17/6/2007
2007G009	2/7/2007
2007G010	28/4/2007
2007G010	10/5/2007
2007G010	20/5/2007
2007G010	31/5/2007
2007G010	10/6/2007
2007G011	1/5/2007
2007G011	23/5/2007
2007G011	3/6/2007
2007G011	14/6/2007
2007G011	23/6/2007
2007G011	3/7/2007
2007G011	14/7/2007
2007G011	25/7/2007
2007G012	3/5/2007
2007G012	9/6/2007
2007G012	18/6/2007
2007G013	11/5/2007
2007G014	11/5/2007
2007G015	11/5/2007
2007G015	24/5/2007
2007G015	2/6/2007
2007G015	11/6/2007
2007G015	21/6/2007
2007G015	3/7/2007
2007G016	12/5/2007
2007G016	25/5/2007
2007G017	16/5/2007
2007G017	27/5/2007
2007G017	6/6/2007
2007G017	16/6/2007
2007G017	26/6/2007
2007G017	6/7/2007

2007G018	20/5/2007
2007G018	31/5/2007
2007G018	11/6/2007
2007G018	22/6/2007
2007G018	2/7/2007
2007G018	11/7/2007
2007G018	21/7/2007
2007G018	1/8/2007
2007G019	20/5/2007
2007G019	31/5/2007
2007G019	13/6/2007
2007G019	23/6/2007
2007G019	4/7/2007
2007G019	15/7/2007
2007G019	24/7/2007
2007G020	24/5/2007
2007G020	13/6/2007
2007G020	23/6/2007
2007G020	4/7/2007
2007G020	15/7/2007
2007G021	30/5/2007
2007G021	9/6/2007
2007G021	19/6/2007
2007G021	9/7/2007
2007G021	19/7/2007
2007G021	28/7/2007
2007G021	29/7/2007
2007G021	8/8/2007
2007G021	19/8/2007
2007G021	30/8/2007
2007G022	1/6/2007
2007G022	21/6/2007
2007G022	2/7/2007
2007G023	2/6/2007
2007G023	12/6/2007
2007G023	22/6/2007
2007G023	3/7/2007
2007G023	23/7/2007
2007G023	2/8/2007
2007G023	12/8/2007
2007G023	23/8/2007
2007G023	3/9/2007
2007G024	13/6/2007
2007G024	22/6/2007
2007G024	2/7/2007
2007G024	10/7/2007

2007G024	19/7/2007
2007G024	28/7/2007
2007G024	6/8/2007
2007G024	17/8/2007
2007G025	13/6/2007
2007G025	21/6/2007
2007G025	1/7/2007
2007G026	14/6/2007
2007G026	23/6/2007
2007G026	3/7/2007
2007G026	12/7/2007
2007G026	21/7/2007
2007G026	1/8/2007
2007G026	12/8/2007
2007G027	20/6/2007
2007G027	1/7/2007
2007G027	11/7/2007
2007G027	23/7/2007
2007G027	2/8/2007
2007G027	13/8/2007
2007G028	22/6/2007
2007G028	4/7/2007
2007G028	28/7/2007
2007G029	27/6/2007
2007G029	17/7/2007
2007G029	29/7/2007
2007G030	2/7/2007
2007G030	12/7/2007
2007G030	23/7/2007
2007G030	3/8/2007
2007G030	14/8/2007
2007G030	3/9/2007
2007G030	14/9/2007
2007G031	7/7/2007
2007G031	19/7/2007
2007G031	1/8/2007
2007G031	11/8/2007
2007G031	22/8/2007
2007G031	2/9/2007
2007G032	11/7/2007
2007G033	14/7/2007
2007G033	26/7/2007
2007G033	5/8/2007
2007G033	16/8/2007
2007G034	18/7/2007
2007G035	20/7/2007

2007G035	1/8/2007
2007G035	13/8/2007
2007G035	6/9/2007
2007G035	16/9/2007
2007G036	23/7/2007
2007G036	3/8/2007
2007G036	14/8/2007
2007G036	25/8/2007
2007G036	5/9/2007
2007G036	15/9/2007
2007G036	27/9/2007
2007G036	10/10/2007
2007G037	3/8/2007
2007G037	13/8/2007
2007G037	1/9/2007
2007G038	25/8/2007
1997G032	18/8/2008
2002G005	10/5/2008
2004G030	17/8/2008
2008G018	10/5/2008
2008G022	6/5/2008
2008G029	3/5/2008
2008G033	6/5/2008
2008G035	10/5/2008
2008G037	11/5/2008
1994G022	14/7/2009
1994G022	24/7/2009
1994G022	3/8/2009
1994G022	14/8/2009
1994G022	23/8/2009
1994G022	2/9/2009
1994G046	5/8/2009
1994G046	17/8/2009
1994G046	28/8/2009
1994G046	18/9/2009
1994G046	4/8/2009
1994G046	6/9/2009
1994G046	7/9/2009
1995G024	18/4/2009
1995G024	30/4/2009
1995G024	12/5/2009
1995G024	22/5/2009
1995G024	2/6/2009
1995G024	12/6/2009
1995G024	23/6/2009
1995G024	13/7/2009

1995G024	23/7/2009
1995G109	2/7/2009
1996G011	28/5/2009
1996G011	9/6/2009
1996G011	19/6/2009
1996G011	29/6/2009
1996G011	9/7/2009
1996G011	19/7/2009
1996G011	30/7/2009
1996G018	17/4/2009
1996G018	28/4/2009
1996G018	8/5/2009
1996G018	28/5/2009
1996G018	6/6/2009
1996G018	16/6/2009
1996G018	25/6/2009
1996G018	4/7/2009
1996G018	14/7/2009
1997G031	14/6/2009
1997G031	25/6/2009
1997G031	14/7/2009
1997G031	23/7/2009
1997G031	3/8/2009
1997G050	15/7/2009
1997G050	26/7/2009
1997G050	4/8/2009
1997G050	14/8/2009
1997G050	23/8/2009
1997G050	31/8/2009
1997G050	10/9/2009
1997G050	18/9/2009
1997G050	26/9/2009
1997G050	6/10/2009
1997G050	22/8/2009
1998G005	4/5/2009
1998G005	16/5/2009
1998G005	26/5/2009
1998G005	5/6/2009
1998G005	15/6/2009
1998G005	25/6/2009
1999G018	18/5/2009
1999G042	29/3/2009
1999G042	12/4/2009
1999G042	22/4/2009
1999G042	2/5/2009
1999G042	13/5/2009

1999G042	25/5/2009
1999G042	5/6/2009
1999G042	15/6/2009
1999G042	26/6/2009
1999G042	7/7/2009
1999G065	6/9/2009
1999G065	18/9/2009
1999G065	28/9/2009
1999G065	5/9/2009
2000G011	2/2/2009
2000G011	15/2/2009
2000G011	1/3/2009
2000G011	13/3/2009
2001G035	20/5/2009
2001G035	31/5/2009
2001G035	10/6/2009
2001G035	20/6/2009
2001G035	30/6/2009
2001G035	10/7/2009
2001G035	22/7/2009
2001G036	18/6/2009
2001G036	29/6/2009
2001G036	10/7/2009
2001G036	22/7/2009
2001G036	2/8/2009
2001G036	13/8/2009
2001G036	23/8/2009
2002G006	24/4/2009
2002G006	5/5/2009
2002G006	15/5/2009
2002G006	26/5/2009
2002G006	4/6/2009
2002G006	14/6/2009
2002G006	4/7/2009
2002G010	13/7/2009
2002G010	23/7/2009
2002G010	3/8/2009
2002G010	25/8/2009
2002G010	5/9/2009
2002G010	4/9/2009
2004G002	21/7/2009
2004G002	31/7/2009
2004G002	9/8/2009
2004G002	19/8/2009
2004G002	6/9/2009
2004G009	18/5/2009

2004G009	29/5/2009
2004G010	18/5/2009
2004G011	9/5/2009
2004G014	3/4/2009
2004G014	14/4/2009
2004G014	4/5/2009
2004G014	13/5/2009
2004G014	22/5/2009
2004G014	1/6/2009
2004G014	11/6/2009
2004G046	4/5/2009
2004G046	14/5/2009
2004G046	24/5/2009
2004G046	4/6/2009
2004G046	13/6/2009
2004G046	23/6/2009
2004G046	2/7/2009
2004G051	26/7/2009
2004G051	28/8/2009
2004G051	7/9/2009
2006G001	12/4/2009
2006G001	23/4/2009
2006G001	3/5/2009
2006G021	14/5/2009
2006G021	25/5/2009
2006G021	4/6/2009
2008G014	18/9/2009
2008G078	17/1/2009
2008G078	27/1/2009
2008G078	12/2/2009
2008G078	26/2/2009
2008G079	5/1/2009
2008G079	17/1/2009
2008G079	29/1/2009
2008G079	9/2/2009
2008G079	20/2/2009
2008G079	3/3/2009
2009G001	10/1/2009
2009G001	23/1/2009
2009G001	5/2/2009
2009G001	20/2/2009
2009G002	12/1/2009
2009G002	26/1/2009
2009G002	7/2/2009
2009G002	18/2/2009
2009G002	6/3/2009

2009G003	22/1/2009
2009G003	3/2/2009
2009G003	15/2/2009
2009G003	27/2/2009
2009G003	12/3/2009
2009G003	24/3/2009
2009G003	3/4/2009
2009G004	11/2/2009
2009G004	23/2/2009
2009G004	7/3/2009
2009G004	18/3/2009
2009G004	30/3/2009
2009G004	11/4/2009
2009G005	20/2/2009
2009G005	5/3/2009
2009G005	17/3/2009
2009G005	28/3/2009
2009G005	7/4/2009
2009G005	18/4/2009
2009G005	29/4/2009
2009G006	16/3/2009
2009G006	28/3/2009
2009G006	8/4/2009
2009G006	19/4/2009
2009G006	28/4/2009
2009G007	20/3/2009
2009G007	31/3/2009
2009G007	11/4/2009
2009G007	21/4/2009
2009G008	20/3/2009
2009G009	21/3/2009
2009G009	1/4/2009
2009G009	12/4/2009
2009G009	23/4/2009
2009G009	4/5/2009
2009G010	23/3/2009
2009G010	2/4/2009
2009G010	12/4/2009
2009G010	21/4/2009
2009G010	3/5/2009
2009G011	2/4/2009
2009G011	13/4/2009
2009G011	23/4/2009
2009G011	3/5/2009
2009G011	14/5/2009
2009G012	3/4/2009

2009G012	13/4/2009
2009G012	22/4/2009
2009G012	2/5/2009
2009G013	4/4/2009
2009G014	8/4/2009
2009G014	18/4/2009
2009G015	8/4/2009
2009G015	19/4/2009
2009G015	9/5/2009
2009G015	20/5/2009
2009G015	30/5/2009
2009G015	9/6/2009
2009G015	19/6/2009
2009G015	2/7/2009
2009G016	10/4/2009
2009G016	20/4/2009
2009G016	29/4/2009
2009G016	9/5/2009
2009G016	19/5/2009
2009G016	29/5/2009
2009G017	14/4/2009
2009G017	25/4/2009
2009G018	26/4/2009
2009G018	8/5/2009
2009G018	20/5/2009
2009G019	30/4/2009
2009G019	12/5/2009
2009G019	5/6/2009
2009G019	15/6/2009
2009G019	8/7/2009
2009G019	22/5/2009
2009G019	26/6/2009
2009G019	6/7/2009
2009G020	4/5/2009
2009G020	14/5/2009
2009G020	23/5/2009
2009G020	1/6/2009
2009G020	11/6/2009
2009G021	5/5/2009
2009G021	15/5/2009
2009G021	25/5/2009
2009G021	3/6/2009
2009G021	13/6/2009
2009G021	23/6/2009
2009G021	4/7/2009
2009G022	9/5/2009

2009G023	16/5/2009
2009G023	26/5/2009
2009G024	18/5/2009
2009G024	26/5/2009
2009G024	5/6/2009
2009G024	14/6/2009
2009G024	23/6/2009
2009G024	5/7/2009
2009G025	23/5/2009
2009G025	10/7/2009
2009G025	21/7/2009
2009G025	31/7/2009
2009G026	29/5/2009
2009G026	9/6/2009
2009G026	20/6/2009
2009G026	21/7/2009
2009G027	29/5/2009
2009G027	11/6/2009
2009G027	22/6/2009
2009G027	1/7/2009
2009G027	13/7/2009
2009G027	21/6/2009
2009G028	1/6/2009
2009G028	11/6/2009
2009G028	22/6/2009
2009G028	2/7/2009
2009G028	14/7/2009
2009G028	17/7/2009
2009G029	15/6/2009
2009G029	27/6/2009
2009G029	8/7/2009
2009G029	30/7/2009
2009G030	15/6/2009
2009G030	4/8/2009
2009G030	18/8/2009
2009G031	20/6/2009
2009G031	29/6/2009
2009G031	10/7/2009
2009G031	19/7/2009
2009G031	30/7/2009
2009G031	9/8/2009
2009G032	22/6/2009
2009G032	5/7/2009
2009G032	17/7/2009
2009G032	28/7/2009
2009G032	8/8/2009

2009G032	20/8/2009
2009G032	31/8/2009
2009G033	26/6/2009
2009G033	7/7/2009
2009G033	17/7/2009
2009G033	27/7/2009
2009G033	18/8/2009
2009G033	11/9/2009
2009G033	25/6/2009
2009G033	6/8/2009
2009G034	27/6/2009
2009G034	9/7/2009
2009G034	19/7/2009
2009G034	29/7/2009
2009G034	8/8/2009
2009G034	18/8/2009
2009G034	27/8/2009
2009G034	6/9/2009
2009G034	17/9/2009
2009G035	3/7/2009
2009G035	13/7/2009
2009G036	5/7/2009
2009G036	16/7/2009
2009G036	27/7/2009
2009G036	20/8/2009
2009G037	7/7/2009
2009G037	17/7/2009
2009G037	27/7/2009
2009G037	7/8/2009
2009G037	17/8/2009
2009G037	27/8/2009
2009G037	6/9/2009
2009G038	8/7/2009
2009G038	27/7/2009
2009G038	6/8/2009
2009G038	16/8/2009
2009G038	26/8/2009
2009G039	14/7/2009
2009G039	25/7/2009
2009G039	4/8/2009
2009G039	14/8/2009
2009G039	24/8/2009
2009G039	23/7/2009
2009G040	14/7/2009
2009G040	26/7/2009
2009G040	5/8/2009

2009G040	16/8/2009
2009G040	26/8/2009
2009G041	16/7/2009
2009G041	27/7/2009
2009G041	6/8/2009
2009G041	16/8/2009
2009G041	26/8/2009
2009G041	6/9/2009
2009G042	25/7/2009
2009G042	4/8/2009
2009G042	14/8/2009
2009G042	23/8/2009
2009G042	1/9/2009
2009G043	26/7/2009
2009G044	27/7/2009
2009G044	7/8/2009
2009G044	19/8/2009
2009G044	9/9/2009
2009G044	21/9/2009
2009G045	30/7/2009
2009G046	30/7/2009
2009G046	23/8/2009
2009G046	5/9/2009
2009G047	31/7/2009
2009G047	10/8/2009
2009G047	1/9/2009
2009G048	31/7/2009
2009G048	21/9/2009
2009G049	2/8/2009
2009G049	12/8/2009
2009G049	22/8/2009
2009G049	2/9/2009
2009G049	22/9/2009
2009G049	12/9/2009
2009G050	6/8/2009
2009G051	10/8/2009
2009G051	22/8/2009
2009G052	20/8/2009
2009G052	11/9/2009
2009G052	1/10/2009
2009G052	12/10/2009
2009G053	20/8/2009
2009G053	31/8/2009
2009G053	9/9/2009
2009G053	18/9/2009
2009G053	27/9/2009

2009G053	7/10/2009
2009G053	19/10/2009
2009G054	6/9/2009
2009G055	8/9/2009
2009G055	20/9/2009
2009G055	2/10/2009
2009G055	23/10/2009
2009G055	4/11/2009
2009G056	10/9/2009
2009G057	22/9/2009
2009G058	24/9/2009
2009G059	12/10/2009
2009G059	22/10/2009
1993G001	4/6/2010
1993G026	26/4/2010
1993G026	7/5/2010
1993G026	16/5/2010
1993G026	4/6/2010
1993G026	13/6/2010
1993G026	21/6/2010
1993G026	2/7/2010
1993G026	13/7/2010
1993G039	27/5/2010
1993G039	5/6/2010
1993G039	15/6/2010
1993G039	24/6/2010
1993G039	4/7/2010
1993G039	14/7/2010
1993G039	27/7/2010
1993G041	30/6/2010
1993G041	9/7/2010
1993G041	19/7/2010
1993G041	29/7/2010
1993G041	8/8/2010
1993G104	4/10/2010
1993G104	17/10/2010
1995G087	31/5/2010
1995G087	11/6/2010
1995G087	3/7/2010
1995G087	25/7/2010
1995G087	14/8/2010
1995G104	1/5/2010
1997G018	1/3/2010
1997G018	11/3/2010
1997G018	25/3/2010
1997G018	5/4/2010

1997G048	5/7/2010
1997G048	16/7/2010
1997G048	29/7/2010
1997G048	9/8/2010
1997G048	20/8/2010
1998G004	15/6/2010
1998G004	26/6/2010
1998G004	16/7/2010
1998G004	26/7/2010
1998G004	5/8/2010
1998G004	15/8/2010
1998G020	21/4/2010
1998G020	30/4/2010
1998G020	11/5/2010
1998G020	22/5/2010
1998G020	30/5/2010
1998G020	9/6/2010
1998G020	19/6/2010
1998G020	29/6/2010
1998G020	17/6/2010
1998G023	26/4/2010
1998G023	6/5/2010
1998G023	14/5/2010
1998G023	23/5/2010
1998G023	1/6/2010
1998G023	9/6/2010
1998G023	18/6/2010
1998G023	1/6/2010
1998G030	9/7/2010
1998G030	20/7/2010
1998G030	31/7/2010
1998G030	9/8/2010
1998G030	10/8/2010
1998G030	19/8/2010
1998G030	27/8/2010
1998G030	6/9/2010
2000G010	21/6/2010
2000G010	2/7/2010
2000G010	16/7/2010
2000G010	27/7/2010
2000G010	6/8/2010
2000G010	16/8/2010
2000G010	27/8/2010
2000G010	6/6/2010
2000G016	19/5/2010
2000G016	28/5/2010

2000G016	5/6/2010
2000G016	22/6/2010
2000G016	1/7/2010
2000G016	10/7/2010
2000G016	14/6/2010
2000G019	7/5/2010
2000G019	17/5/2010
2000G019	27/5/2010
2000G019	7/6/2010
2000G019	16/6/2010
2000G019	26/6/2010
2000G019	7/7/2010
2000G019	5/6/2010
2001G001	25/4/2010
2001G001	5/5/2010
2001G001	14/5/2010
2001G001	24/5/2010
2001G001	2/6/2010
2001G001	11/6/2010
2001G001	20/6/2010
2001G001	29/6/2010
2001G005	5/2/2010
2001G005	16/2/2010
2001G005	28/2/2010
2001G005	11/3/2010
2001G005	21/3/2010
2001G005	1/4/2010
2001G005	22/4/2010
2001G013	23/6/2010
2001G013	15/7/2010
2001G013	27/7/2010
2001G013	18/8/2010
2001G031	7/8/2010
2002G024	21/8/2010
2002G024	30/8/2010
2002G024	19/9/2010
2002G024	7/10/2010
2003G001	2/6/2010
2003G001	13/6/2010
2003G001	23/6/2010
2003G001	3/7/2010
2003G001	15/7/2010
2003G001	26/7/2010
2003G001	7/8/2010
2003G001	18/8/2010
2004G054	16/6/2010

2004G059	16/6/2010
2004G059	28/6/2010
2004G059	7/7/2010
2004G059	8/8/2010
2004G059	19/8/2010
2005G008	19/5/2010
2005G008	19/6/2010
2005G008	30/6/2010
2005G008	10/7/2010
2006G002	7/4/2010
2006G002	29/4/2010
2006G002	9/5/2010
2006G002	18/5/2010
2006G002	27/5/2010
2006G002	5/6/2010
2006G002	15/6/2010
2006G002	26/6/2010
2006G006	10/4/2010
2006G006	19/4/2010
2006G006	8/5/2010
2006G006	17/5/2010
2006G006	26/5/2010
2006G006	4/6/2010
2006G022	18/4/2010
2006G036	17/8/2010
2006G036	29/8/2010
2007G012	10/4/2010
2007G012	20/4/2010
2007G012	1/5/2010
2007G012	12/5/2010
2007G012	3/6/2010
2007G012	15/6/2010
2007G019	10/5/2010
2007G019	20/5/2010
2007G019	30/5/2010
2007G019	9/6/2010
2007G020	7/5/2010
2007G020	6/6/2010
2007G020	16/6/2010
2007G020	27/6/2010
2007G020	7/7/2010
2007G022	23/4/2010
2007G037	23/5/2010
2007G037	1/6/2010
2008G001	24/4/2010
2008G001	4/5/2010

2008G001	14/5/2010
2008G001	23/5/2010
2008G001	2/6/2010
2008G001	11/6/2010
2008G001	30/6/2010
2008G004	24/10/2010
2008G079	13/7/2010
2008G079	24/7/2010
2008G079	25/8/2010
2008G079	6/9/2010
2009G007	25/8/2010
2009G007	16/9/2010
2009G007	26/9/2010
2009G007	5/10/2010
2009G007	16/10/2010
2009G007	26/10/2010
2010G001	10/1/2010
2010G001	20/1/2010
2010G001	31/1/2010
2010G001	11/2/2010
2010G001	21/2/2010
2010G001	3/3/2010
2010G002	28/1/2010
2010G002	8/2/2010
2010G002	19/2/2010
2010G002	2/3/2010
2010G002	13/3/2010
2010G002	23/3/2010
2010G002	3/4/2010
2010G002	14/4/2010
2010G003	10/2/2010
2010G004	13/2/2010
2010G004	5/3/2010
2010G004	15/3/2010
2010G004	26/3/2010
2010G004	6/4/2010
2010G004	17/4/2010
2010G005	16/2/2010
2010G005	4/3/2010
2010G005	4/4/2010
2010G005	14/4/2010
2010G005	3/5/2010
2010G006	23/2/2010
2010G006	6/3/2010
2010G006	17/3/2010
2010G006	27/3/2010

2010G006	6/4/2010
2010G006	16/4/2010
2010G006	24/4/2010
2010G007	3/3/2010
2010G007	15/3/2010
2010G007	26/3/2010
2010G008	3/3/2010
2010G008	15/3/2010
2010G008	6/4/2010
2010G008	25/4/2010
2010G008	14/5/2010
2010G009	4/3/2010
2010G009	14/3/2010
2010G009	26/3/2010
2010G009	5/4/2010
2010G009	27/4/2010
2010G009	7/5/2010
2010G009	16/5/2010
2010G010	6/3/2010
2010G010	16/3/2010
2010G010	27/3/2010
2010G010	6/4/2010
2010G010	17/4/2010
2010G010	23/6/2010
2010G011	6/3/2010
2010G011	16/3/2010
2010G011	29/3/2010
2010G011	9/4/2010
2010G012	9/3/2010
2010G012	31/3/2010
2010G013	18/3/2010
2010G013	1/4/2010
2010G013	12/4/2010
2010G013	24/4/2010
2010G013	2/5/2010
2010G013	12/5/2010
2010G013	22/5/2010
2010G013	2/6/2010
2010G014	19/3/2010
2010G014	29/3/2010
2010G014	8/4/2010
2010G014	17/4/2010
2010G015	9/4/2010
2010G015	19/4/2010
2010G016	20/3/2010
2010G017	20/3/2010

2010G017	31/3/2010
2010G017	10/4/2010
2010G017	19/4/2010
2010G017	30/4/2010
2010G017	11/5/2010
2010G018	21/3/2010
2010G018	1/4/2010
2010G018	11/4/2010
2010G018	3/5/2010
2010G018	13/5/2010
2010G018	25/5/2010
2010G018	7/6/2010
2010G019	23/3/2010
2010G019	4/4/2010
2010G019	13/4/2010
2010G019	22/4/2010
2010G019	1/5/2010
2010G019	11/5/2010
2010G019	20/5/2010
2010G019	31/5/2010
2010G020	25/3/2010
2010G020	4/4/2010
2010G020	15/4/2010
2010G020	4/5/2010
2010G020	14/5/2010
2010G020	24/5/2010
2010G020	3/6/2010
2010G020	25/4/2010
2010G021	26/3/2010
2010G021	6/4/2010
2010G021	16/4/2010
2010G021	26/4/2010
2010G021	6/5/2010
2010G021	15/5/2010
2010G022	26/3/2010
2010G022	7/4/2010
2010G022	17/4/2010
2010G022	28/4/2010
2010G022	8/5/2010
2010G022	18/5/2010
2010G022	29/5/2010
2010G023	1/4/2010
2010G023	13/4/2010
2010G023	6/5/2010
2010G023	16/5/2010
2010G023	4/6/2010

2010G024	4/4/2010
2010G025	4/4/2010
2010G025	15/4/2010
2010G025	24/4/2010
2010G025	3/5/2010
2010G025	13/5/2010
2010G025	23/5/2010
2010G025	2/6/2010
2010G026	4/4/2010
2010G026	23/5/2010
2010G027	5/4/2010
2010G027	16/4/2010
2010G027	25/4/2010
2010G027	14/5/2010
2010G028	5/4/2010
2010G028	16/4/2010
2010G028	27/4/2010
2010G028	8/5/2010
2010G028	17/5/2010
2010G028	26/5/2010
2010G028	5/6/2010
2010G029	5/4/2010
2010G029	15/4/2010
2010G029	24/4/2010
2010G029	3/5/2010
2010G030	8/4/2010
2010G030	8/5/2010
2010G030	31/5/2010
2010G030	29/5/2010
2010G031	9/4/2010
2010G031	19/4/2010
2010G031	30/4/2010
2010G031	10/5/2010
2010G031	19/5/2010
2010G031	1/6/2010
2010G031	11/6/2010
2010G032	9/4/2010
2010G032	19/4/2010
2010G032	29/4/2010
2010G032	11/5/2010
2010G032	20/5/2010
2010G032	31/5/2010
2010G032	11/6/2010
2010G033	10/4/2010
2010G033	19/4/2010
2010G033	30/4/2010

2010G033	23/5/2010
2010G034	13/4/2010
2010G034	23/4/2010
2010G034	3/5/2010
2010G034	25/5/2010
2010G035	14/4/2010
2010G036	14/4/2010
2010G036	3/5/2010
2010G036	13/5/2010
2010G036	24/5/2010
2010G036	24/5/2010
2010G037	17/4/2010
2010G038	18/4/2010
2010G038	29/4/2010
2010G039	22/4/2010
2010G039	2/5/2010
2010G040	22/4/2010
2010G040	2/5/2010
2010G040	12/5/2010
2010G040	22/5/2010
2010G040	1/6/2010
2010G040	11/6/2010
2010G040	20/6/2010
2010G040	1/7/2010
2010G041	22/4/2010
2010G041	6/5/2010
2010G041	17/5/2010
2010G042	22/4/2010
2010G042	5/5/2010
2010G042	16/5/2010
2010G042	28/5/2010
2010G042	8/6/2010
2010G042	24/6/2010
2010G042	5/7/2010
2010G042	19/7/2010
2010G042	6/6/2010
2010G043	24/4/2010
2010G043	5/5/2010
2010G043	17/5/2010
2010G043	27/5/2010
2010G043	10/6/2010
2010G043	22/6/2010
2010G044	25/4/2010
2010G045	25/4/2010
2010G045	5/5/2010
2010G045	15/5/2010

2010g045	14/6/2010
2010G045	4/6/2010
2010G046	28/4/2010
2010G046	8/5/2010
2010G046	17/5/2010
2010G046	27/5/2010
2010G046	5/6/2010
2010G046	15/6/2010
2010G046	25/6/2010
2010G046	6/7/2010
2010G046	25/6/2010
2010G047	28/4/2010
2010G047	8/5/2010
2010G047	18/5/2010
2010G047	27/5/2010
2010G047	5/6/2010
2010G047	14/6/2010
2010G047	24/6/2010
2010G048	29/4/2010
2010G048	10/5/2010
2010G048	21/5/2010
2010G048	13/6/2010
2010G048	24/6/2010
2010G048	16/7/2010
2010G048	1/6/2010
2010G049	29/4/2010
2010G050	30/4/2010
2010G051	2/5/2010
2010G051	12/5/2010
2010G051	23/5/2010
2010G051	2/6/2010
2010G051	11/6/2010
2010G051	20/6/2010
2010G051	29/6/2010
2010G051	8/7/2010
2010G051	1/6/2010
2010G052	2/5/2010
2010G052	13/5/2010
2010G052	22/5/2010
2010G052	2/6/2010
2010G052	11/6/2010
2010G052	19/6/2010
2010G052	7/7/2010
2010G052	17/7/2010
2010G052	1/6/2010
2010G053	3/5/2010

2010G053	13/5/2010
2010G053	25/5/2010
2010G053	3/6/2010
2010G053	13/6/2010
2010G053	26/6/2010
2010G053	3/7/2010
2010G053	3/6/2010
2010G054	4/5/2010
2010G054	15/5/2010
2010G054	16/6/2010
2010G054	27/6/2010
2010G054	7/7/2010
2010G055	4/5/2010
2010G055	8/6/2010
2010G055	20/6/2010
2010G055	2/7/2010
2010G056	8/5/2010
2010G056	23/5/2010
2010G056	3/6/2010
2010G056	13/6/2010
2010G056	24/6/2010
2010G057	9/5/2010
2010G057	18/5/2010
2010G057	17/6/2010
2010G057	27/6/2010
2010G057	7/7/2010
2010G057	17/7/2010
2010G057	15/6/2010
2010G058	10/5/2010
2010G058	23/5/2010
2010G058	2/6/2010
2010G058	13/6/2010
2010G058	22/6/2010
2010G058	3/7/2010
2010G059	10/5/2010
2010G059	25/5/2010
2010G059	5/6/2010
2010G059	16/6/2010
2010G059	27/6/2010
2010G059	8/7/2010
2010G059	31/7/2010
2010G060	10/5/2010
2010G060	19/5/2010
2010G060	28/5/2010
2010G060	5/6/2010
2010G060	15/6/2010

2010G060	25/6/2010
2010G060	5/6/2010
2010G061	12/5/2010
2010G062	13/5/2010
2010G062	22/5/2010
2010G062	1/6/2010
2010G062	11/6/2010
2010G063	14/5/2010
2010G064	14/5/2010
2010G065	15/5/2010
2010G066	16/5/2010
2010G066	17/6/2010
2010G066	26/5/2010
2010G067	17/5/2010
2010G067	28/5/2010
2010G067	7/6/2010
2010G067	19/6/2010
2010G068	18/5/2010
2010G069	19/5/2010
2010G069	13/6/2010
2010G069	22/6/2010
2010G069	5/7/2010
2010G069	15/7/2010
2010G069	26/7/2010
2010G069	31/5/2010
2010G070	20/5/2010
2010G070	29/5/2010
2010G070	15/6/2010
2010G070	23/6/2010
2010G070	6/6/2010
2010G071	20/5/2010
2010G071	31/5/2010
2010G071	9/6/2010
2010G071	10/7/2010
2010G071	21/7/2010
2010G071	4/8/2010
2010G071	14/5/2010
2010G071	30/5/2010
2010G071	29/6/2010
2010G072	20/5/2010
2010G073	23/5/2010
2010G073	2/6/2010
2010G073	12/6/2010
2010G074	23/5/2010
2010G074	4/6/2010
2010G075	24/5/2010

2010G075	3/6/2010
2010G075	13/6/2010
2010G075	22/6/2010
2010G075	3/7/2010
2010G075	15/7/2010
2010G075	2/6/2010
2010G076	25/5/2010
2010G076	3/6/2010
2010G076	14/6/2010
2010G077	26/5/2010
2010G078	28/5/2010
2010G078	5/7/2010
2010G078	15/7/2010
2010G078	25/6/2010
2010G079	28/5/2010
2010G080	29/5/2010
2010G080	9/6/2010
2010G080	18/6/2010
2010G081	30/5/2010
2010G081	10/6/2010
2010G081	21/6/2010
2010G081	2/7/2010
2010G082	30/5/2010
2010G082	10/6/2010
2010G082	20/6/2010
2010G082	30/6/2010
2010G082	11/7/2010
2010G082	30/7/2010
2010G082	9/8/2010
2010G082	19/8/2010
2010G082	28/8/2010
2010G082	7/9/2010
2010G083	1/6/2010
2010G083	14/6/2010
2010G083	23/6/2010
2010G083	2/7/2010
2010G083	12/7/2010
2010G083	21/7/2010
2010G084	1/6/2010
2010G084	10/6/2010
2010G084	19/6/2010
2010G084	28/6/2010
2010G084	7/7/2010
2010G084	15/7/2010
2010G084	24/7/2010
2010G084	2/8/2010

2010G084	11/8/2010
2010G085	2/6/2010
2010G086	3/6/2010
2010G086	13/6/2010
2010G086	2/7/2010
2010G086	13/7/2010
2010G086	2/8/2010
2010G087	4/6/2010
2010G087	16/6/2010
2010G087	27/6/2010
2010G087	7/7/2010
2010G087	17/7/2010
2010G087	27/7/2010
2010G087	7/8/2010
2010G089	5/6/2010
2010G089	16/6/2010
2010G089	9/7/2010
2010G089	20/7/2010
2010G090	5/6/2010
2010G090	15/6/2010
2010G090	24/6/2010
2010G090	25/6/2010
2010G091	5/6/2010
2010G091	16/6/2010
2010G092	10/6/2010
2010G092	22/6/2010
2010G092	4/7/2010
2010G092	16/7/2010
2010G092	27/7/2010
2010G093	15/6/2010
2010G094	15/6/2010
2010G094	27/6/2010
2010G094	8/7/2010
2010G094	19/7/2010
2010G094	31/7/2010
2010G095	16/6/2010
2010G096	16/6/2010
2010G096	29/6/2010
2010G097	16/6/2010
2010G097	27/6/2010
2010G097	6/7/2010
2010G097	16/7/2010
2010G097	26/7/2010
2010G097	5/8/2010
2010G097	28/8/2010
2010G098	17/6/2010

2010G098	29/6/2010
2010G098	8/7/2010
2010G098	18/7/2010
2010G099	18/6/2010
2010G099	13/7/2010
2010G099	24/7/2010
2010G099	4/8/2010
2010G099	15/8/2010
2010G100	19/6/2010
2010G100	28/6/2010
2010G100	8/7/2010
2010G100	18/7/2010
2010G100	6/8/2010
2010G100	16/8/2010
2010G101	21/6/2010
2010G101	8/7/2010
2010G103	23/6/2010
2010G103	6/7/2010
2010G103	14/7/2010
2010G103	24/7/2010
2010G103	4/8/2010
2010G104	28/6/2010
2010G104	10/7/2010
2010G105	3/7/2010
2010G106	10/7/2010
2010G106	22/7/2010
2010G107	13/7/2010
2010G107	24/7/2010
2010G107	3/8/2010
2010G107	13/8/2010
2010G108	26/7/2010
2010G108	7/8/2010
2010G108	20/8/2010
2010G109	27/7/2010
2010G109	5/8/2010
2010G110	27/7/2010
2010G110	17/8/2010
2010G110	28/8/2010
2010G110	7/9/2010
2010G111	8/8/2010
2010G111	18/8/2010
2010G111	28/8/2010
2010G112	9/8/2010
2010G113	14/8/2010
2010G113	24/8/2010
2010G114	14/8/2010

2010G114	27/8/2010
2010G114	8/9/2010
2010G115	25/8/2010
2010G116	11/9/2010
1993G019	5/8/2011
1993G019	16/8/2011
1993G019	26/8/2011
1993G019	6/9/2011
1993G019	16/9/2011
1993G019	27/9/2011
1993G050	15/5/2011
1993G050	27/5/2011
1993G111	6/7/2011
1993G111	18/7/2011
1993G111	28/7/2011
1993G111	7/8/2011
1993G111	17/8/2011
1993G111	27/8/2011
1993G111	7/9/2011
1993G111	17/9/2011
1993G140	11/9/2011
1994G053	4/8/2011
1995G011	17/7/2011
1995G011	19/8/2011
1995G011	29/8/2011
1995G011	8/9/2011
1995G011	19/9/2011
1995G011	30/9/2011
1995G039	29/6/2011
1995G039	18/7/2011
1995G039	4/8/2011
1995G039	13/8/2011
1995G039	30/8/2011
1995G039	8/9/2011
1996G009	3/6/2011
1996G009	13/6/2011
1996G009	22/6/2011
1996G009	2/7/2011
1996G009	12/7/2011
1996G009	21/7/2011
1996G009	30/7/2011
1996G009	8/8/2011
1996G009	17/8/2011
1996G021	24/6/2011
1996G021	5/8/2011
1999G002	1/4/2011

1999G010	23/4/2011
1999G010	5/6/2011
1999G010	17/5/2011
1999G010	28/5/2011
1999G010	9/6/2011
1999G010	20/6/2011
1999G010	4/7/2011
1999G024	17/5/2011
1999G024	27/5/2011
1999G024	27/6/2011
1999G024	7/7/2011
2000G012	16/5/2011
2000G024	3/7/2011
2000G024	13/7/2011
2000G024	23/7/2011
2000G024	2/8/2011
2000G024	21/8/2011
2000G024	30/8/2011
2000G024	11/8/2011
2001G036	18/4/2011
2002G003	14/3/2011
2002G003	26/3/2011
2002G003	6/4/2011
2002G003	17/4/2011
2002G003	6/5/2011
2002G003	15/5/2011
2002G003	26/4/2011
2002G023	10/8/2011
2003G008	21/7/2011
2004G030	6/8/2011
2004G030	16/8/2011
2004G030	6/9/2011
2004G043	20/8/2011
2004G043	29/8/2011
2004G043	8/9/2011
2004G043	26/9/2011
2006G012	13/3/2011
2006G012	26/3/2011
2006G012	7/4/2011
2006G012	20/4/2011
2006G012	1/5/2011
2006G012	12/5/2011
2006G012	23/5/2011
2006G015	28/5/2011
2006G015	6/6/2011
2006G015	16/6/2011

2006G015	26/6/2011
2006G015	6/7/2011
2006G015	18/7/2011
2006G015	28/7/2011
2006G025	26/5/2011
2006G025	19/6/2011
2006G025	1/7/2011
2006G025	12/7/2011
2006G025	22/7/2011
2006G025	10/8/2011
2006G025	22/8/2011
2006G025	31/7/2011
2006G034	31/5/2011
2006G034	11/6/2011
2006G034	21/6/2011
2006G034	2/8/2011
2006G034	11/8/2011
2006G034	20/8/2011
2006G039	13/9/2011
2006G039	24/9/2011
2006G039	5/10/2011
2006G039	17/10/2011
2006G039	30/10/2011
2007G002	10/4/2011
2007G005	26/2/2011
2007G005	8/3/2011
2007G005	19/3/2011
2007G005	31/3/2011
2007G005	10/4/2011
2007G005	20/4/2011
2007G005	30/4/2011
2007G005	9/5/2011
2007G031	6/5/2011
2007G031	18/5/2011
2007G031	29/5/2011
2007G031	9/6/2011
2007G031	20/6/2011
2007G031	30/6/2011
2007G031	10/7/2011
2007G031	5/5/2011
2008G001	28/7/2011
2008G001	7/8/2011
2008G001	16/8/2011
2008G001	26/8/2011
2008G001	4/9/2011
2008G001	14/9/2011

2008G001	25/9/2011
2008G002	5/4/2011
2008G002	6/4/2011
2008G002	19/4/2011
2008G002	1/5/2011
2008G006	5/4/2011
2008G006	18/5/2011
2008G006	28/5/2011
2008G006	17/6/2011
2008G006	8/5/2011
2008G007	18/2/2011
2008G007	15/3/2011
2008G007	25/3/2011
2008G007	6/4/2011
2008G007	17/4/2011
2008G007	27/4/2011
2008G007	8/5/2011
2008G007	2/3/2011
2008G011	9/2/2011
2008G011	20/2/2011
2008G011	6/3/2011
2008G011	17/3/2011
2008G011	27/3/2011
2008G011	7/4/2011
2008G014	6/2/2011
2008G014	17/2/2011
2008G017	8/4/2011
2008G017	2/5/2011
2008G021	10/5/2011
2008G021	21/5/2011
2008G021	31/5/2011
2008G063	2/7/2011
2008G063	13/7/2011
2008G063	25/7/2011
2008G063	4/8/2011
2008G063	15/8/2011
2008G063	25/8/2011
2008G063	5/9/2011
2008G063	16/9/2011
2009G044	27/5/2011
2009G044	9/6/2011
2009G044	22/6/2011
2009G044	3/7/2011
2009G044	15/7/2011
2011G001	3/1/2011
2011G001	15/1/2011

2011G001	27/1/2011
2011G001	11/2/2011
2011G001	23/2/2011
2011G001	8/3/2011
2011G001	21/3/2011
2011G001	31/3/2011
2011G002	22/1/2011
2011G002	14/2/2011
2011G002	26/2/2011
2011G003	5/2/2011
2011G003	16/2/2011
2011G003	27/2/2011
2011G003	10/3/2011
2011G003	3/4/2011
2011G003	14/4/2011
2011G004	8/2/2011
2011G004	20/2/2011
2011G004	5/3/2011
2011G004	15/3/2011
2011G004	25/3/2011
2011G004	5/4/2011
2011G004	17/4/2011
2011G004	27/4/2011
2011G005	9/2/2011
2011G005	22/2/2011
2011G005	6/3/2011
2011G005	20/4/2011
2011G005	2/5/2011
2011G005	12/5/2011
2011G006	13/2/2011
2011G006	26/2/2011
2011G007	22/2/2011
2011G007	13/3/2011
2011G008	28/2/2011
2011G008	13/3/2011
2011G008	23/3/2011
2011G008	4/4/2011
2011G008	14/4/2011
2011G008	25/4/2011
2011G008	6/5/2011
2011G008	18/5/2011
2011G009	3/3/2011
2011G009	16/3/2011
2011G009	28/3/2011
2011G009	9/4/2011
2011G009	20/4/2011

2011G009	3/5/2011
2011G009	25/5/2011
2011G009	6/6/2011
2011G010	10/3/2011
2011G010	22/3/2011
2011G010	13/4/2011
2011G010	6/5/2011
2011G011	13/3/2011
2011G011	23/3/2011
2011G011	5/4/2011
2011G011	18/4/2011
2011G011	3/5/2011
2011G012	13/3/2011
2011G012	23/3/2011
2011G013	15/3/2011
2011G013	26/3/2011
2011G013	5/4/2011
2011G013	15/4/2011
2011G013	27/4/2011
2011G014	16/3/2011
2011G014	27/3/2011
2011G015	19/3/2011
2011G015	29/3/2011
2011G015	12/4/2011
2011G015	24/4/2011
2011G016	27/3/2011
2011G017	29/3/2011
2011G018	1/4/2011
2011G018	11/4/2011
2011G018	21/4/2011
2011G019	3/4/2011
2011G019	14/4/2011
2011G019	25/4/2011
2011G019	5/5/2011
2011G019	25/5/2011
2011G019	3/6/2011
2011G019	13/6/2011
2011G019	23/6/2011
2011G019	3/7/2011
2011G019	15/5/2011
2011G020	4/4/2011
2011G020	16/4/2011
2011G020	28/4/2011
2011G020	10/5/2011
2011G021	4/4/2011
2011G021	16/4/2011

2011G021	8/5/2011
2011G021	19/5/2011
2011G021	27/4/2011
2011G022	7/4/2011
2011G022	29/4/2011
2011G023	9/4/2011
2011G023	20/4/2011
2011G023	29/4/2011
2011G023	9/5/2011
2011G023	19/5/2011
2011G023	28/5/2011
2011G024	9/4/2011
2011G025	9/4/2011
2011G025	20/4/2011
2011G026	10/4/2011
2011G027	10/4/2011
2011G027	21/4/2011
2011G027	2/5/2011
2011G028	11/4/2011
2011G028	22/4/2011
2011G029	11/4/2011
2011G029	24/4/2011
2011G030	11/4/2011
2011G031	11/4/2011
2011G031	24/4/2011
2011G031	6/5/2011
2011G031	18/5/2011
2011G031	28/5/2011
2011G031	9/6/2011
2011G032	13/4/2011
2011G033	14/4/2011
2011G034	20/4/2011
2011G034	1/5/2011
2011G034	15/5/2011
2011G034	8/6/2011
2011G034	19/6/2011
2011G034	3/7/2011
2011G034	26/5/2011
2011G035	20/4/2011
2011G036	22/4/2011
2011G036	2/5/2011
2011G036	12/5/2011
2011G036	22/5/2011
2011G036	31/5/2011
2011G036	10/6/2011
2011G037	25/4/2011

2011G037	22/5/2011
2011G037	3/6/2011
2011G038	27/4/2011
2011G038	18/5/2011
2011G038	29/5/2011
2011G039	27/4/2011
2011G039	23/5/2011
2011G039	3/6/2011
2011G039	16/6/2011
2011G040	27/4/2011
2011G040	7/5/2011
2011G040	27/5/2011
2011G040	5/6/2011
2011G040	15/6/2011
2011G040	4/7/2011
2011G041	29/4/2011
2011g041	10/5/2011
2011G041	20/5/2011
2011G041	30/5/2011
2011G041	20/6/2011
2011G042	1/5/2011
2011G042	10/5/2011
2011G043	1/5/2011
2011G043	11/5/2011
2011G044	6/5/2011
2011G044	18/5/2011
2011G044	28/5/2011
2011G044	8/6/2011
2011G044	19/6/2011
2011G044	30/6/2011
2011G044	12/7/2011
2011G044	22/7/2011
2011G044	2/8/2011
2011G045	6/5/2011
2011G045	28/5/2011
2011G045	8/6/2011
2011G045	19/6/2011
2011G046	8/5/2011
2011G047	10/5/2011
2011G048	11/5/2011
2011G048	21/5/2011
2011G048	30/5/2011
2011G048	9/6/2011
2011G048	20/6/2011
2011G048	1/7/2011
2011G049	14/5/2011

2011G049	25/5/2011
2011G049	4/6/2011
2011G049	14/6/2011
2011G049	23/6/2011
2011G049	3/7/2011
2011G049	12/7/2011
2011G050	16/5/2011
2011G050	25/5/2011
2011G050	6/6/2011
2011G051	20/5/2011
2011G051	31/5/2011
2011G051	12/6/2011
2011G052	23/5/2011
2011G052	4/6/2011
2011G052	28/6/2011
2011G052	9/7/2011
2011G052	20/7/2011
2011G052	17/6/2011
2011G053	23/5/2011
2011G054	24/5/2011
2011G054	2/6/2011
2011G054	3/7/2011
2011G054	13/7/2011
2011G054	22/7/2011
2011G055	25/5/2011
2011G056	26/5/2011
2011G056	18/6/2011
2011G056	29/6/2011
2011G056	11/7/2011
2011G056	22/7/2011
2011G056	2/8/2011
2011G056	13/8/2011
2011G057	27/5/2011
2011G058	4/6/2011
2011G058	12/6/2011
2011G058	22/6/2011
2011G058	2/7/2011
2011G058	11/7/2011
2011G058	20/7/2011
2011G058	7/8/2011
2011G058	17/8/2011
2011G059	5/6/2011
2011G059	14/6/2011
2011G059	24/6/2011
2011G060	5/6/2011
2011G060	17/6/2011

2011G060	28/6/2011
2011G060	18/7/2011
2011G060	29/7/2011
2011G060	8/8/2011
2011G060	18/8/2011
2011G060	29/8/2011
2011G061	10/6/2011
2011G061	21/6/2011
2011G061	3/7/2011
2011G061	13/7/2011
2011G061	24/7/2011
2011G061	3/8/2011
2011G061	15/8/2011
2011G062	12/6/2011
2011G062	3/7/2011
2011G062	13/7/2011
2011G062	3/8/2011
2011G062	14/8/2011
2011G062	21/6/2011
2011G063	14/6/2011
2011G064	15/6/2011
2011G064	26/6/2011
2011G064	7/7/2011
2011G064	18/7/2011
2011G064	28/7/2011
2011G064	7/8/2011
2011G064	17/8/2011
2011G064	28/8/2011
2011G065	16/6/2011
2011G065	27/6/2011
2011G065	19/7/2011
2011G065	8/8/2011
2011G065	18/8/2011
2011G065	29/8/2011
2011G066	17/6/2011
2011G066	1/7/2011
2011G066	12/7/2011
2011G066	25/7/2011
2011G067	18/6/2011
2011G067	21/7/2011
2011G068	23/6/2011
2011G069	23/6/2011
2011G069	3/7/2011
2011G069	12/7/2011
2011G069	21/7/2011
2011G069	30/7/2011

2011G069	8/8/2011
2011G069	17/8/2011
2011G070	23/6/2011
2011G070	5/7/2011
2011G070	18/7/2011
2011G071	29/6/2011
2011G072	29/6/2011
2011G072	9/7/2011
2011G072	19/7/2011
2011G072	31/7/2011
2011G072	12/8/2011
2011G072	22/8/2011
2011G073	1/7/2011
2011G073	10/7/2011
2011G073	20/7/2011
2011G073	31/7/2011
2011G074	3/7/2011
2011G074	25/7/2011
2011G074	4/8/2011
2011G074	16/8/2011
2011G075	8/7/2011
2011G075	19/7/2011
2011G075	30/7/2011
2011G075	12/8/2011
2011G075	22/8/2011
2011G075	13/9/2011
2011G075	24/9/2011
2011G075	5/10/2011
2011G075	14/10/2011
2011G075	25/10/2011
2011G076	9/7/2011
2011G076	20/7/2011
2011G077	10/7/2011
2011G077	20/7/2011
2011G077	30/7/2011
2011G078	11/7/2011
2011G078	20/7/2011
2011G078	30/7/2011
2011G079	11/7/2011
2011G080	12/7/2011
2011G081	20/7/2011
2011G081	10/8/2011
2011G081	22/8/2011
2011G081	5/9/2011
2011G081	17/9/2011
2011G081	30/7/2011

2011G081	11/8/2011
2011G082	20/7/2011
2011G082	2/8/2011
2011G083	23/7/2011
2011G083	3/8/2011
2011G083	15/8/2011
2011G083	27/8/2011
2011G084	23/7/2011
2011G084	5/8/2011
2011G084	15/8/2011
2011G084	5/9/2011
2011G084	15/9/2011
2011G085	28/7/2011
2011G086	30/7/2011
2011G086	10/8/2011
2011G086	22/8/2011
2011G086	3/9/2011
2011G086	14/9/2011
2011G086	26/9/2011
2011G086	7/10/2011
2011G086	27/10/2011
2011G087	6/8/2011
2011G087	17/8/2011
2011G087	29/8/2011
2011G088	12/8/2011
2011G089	17/8/2011
2011G090	20/8/2011
2011G090	31/8/2011
2011G090	20/9/2011
2011G090	12/10/2011
2011G090	22/10/2011
2011G091	22/8/2011
2011G091	12/9/2011
2011G091	22/9/2011
2011G091	2/10/2011
2011G092	25/8/2011
2011G093	28/8/2011
2011G093	1/11/2011
2011G093	11/11/2011
2011G094	28/8/2011
2011G094	7/9/2011
2011G094	18/9/2011
2011G094	29/9/2011
2011G094	10/10/2011
2011G095	4/9/2011
2011G096	15/9/2011

2011G096	16/10/2011
2011G096	29/10/2011
2011G097	16/9/2011
2011G097	28/9/2011
2011G097	9/10/2011
2011G097	9/11/2011
2011G097	19/11/2011
2011G097	30/11/2011
2011G098	26/9/2011
2011G098	6/10/2011
2011G098	17/10/2011
2011G098	31/10/2011
2011G099	26/9/2011
2011G099	18/10/2011
2011G099	29/11/2011
2011G100	9/11/2011
2011G100	19/11/2011
2011G100	29/11/2011
1994G022	18/7/2012
1994G022	7/8/2012
1994G022	17/8/2012
1994G022	26/8/2012
1994G022	6/9/2012
1994G022	15/9/2012
1994G022	26/9/2012
1995G024	18/4/2012
1995G024	29/4/2012
1995G024	10/5/2012
1995G024	21/5/2012
1995G024	11/6/2012
1995G024	3/7/2012
1995G024	13/7/2012
1995G024	24/7/2012
1995G095	5/9/2012
1995G095	6/9/2012
1995G095	16/9/2012
1995G095	27/9/2012
1995G095	6/10/2012
1995G095	3/11/2012
1995G095	14/11/2012
2000G011	15/7/2012
2000G011	27/7/2012
2000G011	6/8/2012
2000G011	17/8/2012
2000G011	6/9/2012
2000G011	17/9/2012

2000G011	28/9/2012
2000G016	5/6/2012
2000G016	14/6/2012
2000G016	23/6/2012
2000G016	3/7/2012
2000G016	11/7/2012
2000G016	20/7/2012
2000G016	29/7/2012
2000G026	25/5/2012
2000G026	5/6/2012
2000G026	15/6/2012
2000G026	26/6/2012
2000G026	7/7/2012
2000G026	17/7/2012
2000G026	7/8/2012
2001G005	12/4/2012
2001G005	24/4/2012
2001G005	7/5/2012
2001G005	19/5/2012
2001G005	31/5/2012
2001G005	11/6/2012
2001G005	23/6/2012
2001G005	4/7/2012
2001G013	7/6/2012
2001G013	19/6/2012
2001G013	1/7/2012
2001G013	11/7/2012
2001G013	23/7/2012
2001G013	13/8/2012
2001G031	26/5/2012
2003G001	14/7/2012
2003G001	24/7/2012
2003G001	4/8/2012
2004G047	21/7/2012
2004G047	31/7/2012
2004G047	11/8/2012
2004G047	20/8/2012
2004G047	30/8/2012
2004G047	8/9/2012
2004G051	15/8/2012
2004G051	25/8/2012
2006G021	12/4/2012
2006G021	23/4/2012
2006G021	3/5/2012
2006G021	13/5/2012
2006G021	24/5/2012

2006G021	3/6/2012
2006G021	14/6/2012
2006G021	24/6/2012
2006G021	4/7/2012
2007G017	13/5/2012
2007G017	23/5/2012
2007G017	2/6/2012
2007G017	12/6/2012
2007G017	22/6/2012
2007G026	4/6/2012
2007G026	3/8/2012
2007G026	14/8/2012
2007G026	24/8/2012
2007G030	30/6/2012
2007G030	10/7/2012
2007G030	21/7/2012
2007G030	31/7/2012
2007G030	11/8/2012
2007G030	11/9/2012
2007G030	21/9/2012
2008G012	5/4/2012
2008G012	16/4/2012
2008G012	28/4/2012
2008G012	9/5/2012
2008G012	21/5/2012
2008G012	2/6/2012
2008G012	14/6/2012
2008G012	7/7/2012
2008G026	19/9/2012
2008G040	30/5/2012
2008G040	10/6/2012
2008G041	5/6/2012
2008G041	15/6/2012
2008G041	26/6/2012
2008G069	8/5/2012
2008G069	20/5/2012
2008G069	30/5/2012
2008G069	9/6/2012
2008G069	18/6/2012
2008G069	27/6/2012
2009G003	18/1/2012
2009G003	29/1/2012
2009G003	9/2/2012
2009G003	21/2/2012
2009G003	4/3/2012
2009G003	14/3/2012

2009G003	25/3/2012
2009G003	5/4/2012
2009G003	17/4/2012
2009G005	26/2/2012
2009G005	19/3/2012
2009G005	29/3/2012
2009G005	8/4/2012
2009G005	29/4/2012
2009G005	8/5/2012
2009G005	18/5/2012
2009G006	6/2/2012
2009G006	28/2/2012
2009G006	10/3/2012
2009G006	21/3/2012
2009G006	31/3/2012
2009G006	10/3/2012
2009G011	26/4/2012
2009G011	20/5/2012
2009G035	27/4/2012
2009G035	11/5/2012
2009G035	22/5/2012
2009G035	12/6/2012
2009G035	23/6/2012
2009G035	3/7/2012
2009G035	13/7/2012
2009G039	29/6/2012
2009G049	11/7/2012
2009G049	23/7/2012
2009G049	13/8/2012
2009G049	24/8/2012
2009G049	3/9/2012
2009G049	13/9/2012
2009G049	26/9/2012
2009G059	17/7/2012
2009G059	28/7/2012
2009G059	19/8/2012
2009G059	30/8/2012
2010G015	28/5/2012
2010G015	8/6/2012
2010G015	19/6/2012
2010G015	29/6/2012
2010G015	9/7/2012
2010G015	20/7/2012
2010G015	30/7/2012
2010G015	9/8/2012
2010G015	20/8/2012

2012G001	15/1/2012
2012G001	26/1/2012
2012G001	12/2/2012
2012G001	24/2/2012
2012G002	17/1/2012
2012G002	29/1/2012
2012G002	10/2/2012
2012G002	23/2/2012
2012G002	6/3/2012
2012G003	7/2/2012
2012G003	18/2/2012
2012G004	10/2/2012
2012G004	24/2/2012
2012G004	6/3/2012
2012G004	18/3/2012
2012G004	21/4/2012
2012G005	29/2/2012
2012G005	11/3/2012
2012G005	24/3/2012
2012G006	5/3/2012
2012G006	20/3/2012
2012G006	13/4/2012
2012G006	26/4/2012
2012G006	8/5/2012
2012G006	21/5/2012
2012G006	2/6/2012
2012G007	10/3/2012
2012G007	22/3/2012
2012G007	2/4/2012
2012G007	14/4/2012
2012G007	26/4/2012
2012G007	8/5/2012
2012G007	21/5/2012
2012G008	12/3/2012
2012G009	13/3/2012
2012G010	14/3/2012
2012G011	17/3/2012
2012G011	28/3/2012
2012G011	9/4/2012
2012G012	19/3/2012
2012G012	30/3/2012
2012G013	21/3/2012
2012G013	1/4/2012
2012G013	13/4/2012
2012G013	23/4/2012
2012G013	13/5/2012

2012G013	23/5/2012
2012G013	3/5/2012
2012G014	22/3/2012
2012G014	4/4/2012
2012G014	30/4/2012
2012G014	13/5/2012
2012G014	27/5/2012
2012G015	26/3/2012
2012G015	19/4/2012
2012G015	29/4/2012
2012G015	10/5/2012
2012G015	21/5/2012
2012G016	27/3/2012
2012G016	7/4/2012
2012G016	18/4/2012
2012G016	28/4/2012
2012G016	8/5/2012
2012G016	20/5/2012
2012G017	31/3/2012
2012G017	11/4/2012
2012G017	22/4/2012
2012G018	2/4/2012
2012G018	13/4/2012
2012G018	23/4/2012
2012G018	2/5/2012
2012G018	11/5/2012
2012G018	22/5/2012
2012G019	6/4/2012
2012G019	16/4/2012
2012G019	26/4/2012
2012G019	7/5/2012
2012G019	17/5/2012
2012G020	6/4/2012
2012G020	17/4/2012
2012G020	27/4/2012
2012G020	9/5/2012
2012G020	20/5/2012
2012G020	1/6/2012
2012G021	6/4/2012
2012G021	18/4/2012
2012G021	28/4/2012
2012G021	9/5/2012
2012G021	19/5/2012
2012G021	31/5/2012
2012G021	10/6/2012
2012G022	7/4/2012

2012G022	18/4/2012
2012G022	28/4/2012
2012G022	9/5/2012
2012G022	20/5/2012
2012G023	7/4/2012
2012G024	7/4/2012
2012G024	18/4/2012
2012G024	27/4/2012
2012G024	7/5/2012
2012G024	16/5/2012
2012G025	9/4/2012
2012G025	20/4/2012
2012G025	30/4/2012
2012G025	11/5/2012
2012G025	22/5/2012
2012G025	2/6/2012
2012G025	13/6/2012
2012G026	11/4/2012
2012G026	24/4/2012
2012G026	5/5/2012
2012G026	15/5/2012
2012G026	25/5/2012
2012G026	4/6/2012
2012G026	14/6/2012
2012G026	26/6/2012
2012G027	13/4/2012
2012G027	26/4/2012
2012G027	9/5/2012
2012G027	20/5/2012
2012G027	30/5/2012
2012G028	14/4/2012
2012G028	25/4/2012
2012G028	5/5/2012
2012G028	15/5/2012
2012G028	26/5/2012
2012G028	6/6/2012
2012G028	17/6/2012
2012G029	20/4/2012
2012G029	29/4/2012
2012G029	8/5/2012
2012G029	17/5/2012
2012G029	27/5/2012
2012G030	21/4/2012
2012G030	1/5/2012
2012G030	5/6/2012
2012G031	25/4/2012

2012G031	14/5/2012
2012G031	24/5/2012
2012G031	4/6/2012
2012G031	14/6/2012
2012G031	24/6/2012
2012G031	5/7/2012
2012G032	26/4/2012
2012G032	8/5/2012
2012G032	22/5/2012
2012G032	3/6/2012
2012G032	14/6/2012
2012G032	25/6/2012
2012G032	5/7/2012
2012G032	16/7/2012
2012G032	28/7/2012
2012G033	18/5/2012
2012G034	28/4/2012
2012G034	8/5/2012
2012G035	30/4/2012
2012G035	11/5/2012
2012G035	21/5/2012
2012G035	30/5/2012
2012G036	5/5/2012
2012G036	20/5/2012
2012G036	1/6/2012
2012G036	12/6/2012
2012G036	24/6/2012
2012G036	28/7/2012
2012G036	9/8/2012
2012G037	5/5/2012
2012G037	1/6/2012
2012G038	7/5/2012
2012G038	10/6/2012
2012G039	10/5/2012
2012G039	21/5/2012
2012G039	1/6/2012
2012G039	12/6/2012
2012G039	20/6/2012
2012G039	8/7/2012
2012G039	17/7/2012
2012G039	27/7/2012
2012G039	10/6/2012
2012G040	10/5/2012
2012G040	2/6/2012
2012G040	13/6/2012
2012G040	24/6/2012

2012G040	5/7/2012
2012G041	11/5/2012
2012G041	23/5/2012
2012G041	5/6/2012
2012G041	17/6/2012
2012G041	28/6/2012
2012G042	12/5/2012
2012G042	22/5/2012
2012G042	2/6/2012
2012G042	12/6/2012
2012G042	22/6/2012
2012G042	2/7/2012
2012G043	26/5/2012
2012G043	5/6/2012
2012G043	14/6/2012
2012G043	23/6/2012
2012G043	2/7/2012
2012G043	11/7/2012
2012G043	21/7/2012
2012G044	29/5/2012
2012G044	11/6/2012
2012G044	22/6/2012
2012G044	3/7/2012
2012G044	15/8/2012
2012G045	29/5/2012
2012G045	9/6/2012
2012G045	21/6/2012
2012G045	2/7/2012
2012G045	3/8/2012
2012G046	30/5/2012
2012G047	2/6/2012
2012G047	13/6/2012
2012G047	22/6/2012
2012G047	2/7/2012
2012G047	11/7/2012
2012G048	4/6/2012
2012G048	14/6/2012
2012G048	25/6/2012
2012G048	5/7/2012
2012G048	15/7/2012
2012G048	26/7/2012
2012G048	5/8/2012
2012G049	4/6/2012
2012G049	15/6/2012
2012G049	26/6/2012
2012G049	8/7/2012

2012G050	4/6/2012
2012G050	15/6/2012
2012G051	4/6/2012
2012G052	6/6/2012
2012G052	17/6/2012
2012G052	26/6/2012
2012G052	5/7/2012
2012G052	23/7/2012
2012G052	1/8/2012
2012G052	12/8/2012
2012G053	6/6/2012
2012G053	17/6/2012
2012G053	29/6/2012
2012G054	6/6/2012
2012G054	20/6/2012
2012G054	2/7/2012
2012G054	12/7/2012
2012G054	23/7/2012
2012G054	3/8/2012
2012G054	14/8/2012
2012G055	7/6/2012
2012G055	14/6/2012
2012G056	8/6/2012
2012G057	10/6/2012
2012G057	22/6/2012
2012G057	3/7/2012
2012G057	14/7/2012
2012G057	25/7/2012
2012G057	5/8/2012
2012G057	17/8/2012
2012G057	29/8/2012
2012G058	10/6/2012
2012G058	20/6/2012
2012G058	29/6/2012
2012G058	8/7/2012
2012G058	19/7/2012
2012G059	13/6/2012
2012G060	13/6/2012
2012G060	24/6/2012
2012G060	4/7/2012
2012G060	14/7/2012
2012G060	24/7/2012
2012G060	4/8/2012
2012G061	17/6/2012
2012G061	30/6/2012
2012G061	11/7/2012

2012G061	21/7/2012
2012G061	31/7/2012
2012G061	11/8/2012
2012G062	22/6/2012
2012G062	3/7/2012
2012G062	12/7/2012
2012G062	23/7/2012
2012G062	2/8/2012
2012G062	12/8/2012
2012G063	23/6/2012
2012G063	18/7/2012
2012G063	28/7/2012
2012G063	7/8/2012
2012G063	18/8/2012
2012G063	27/8/2012
2012G063	6/9/2012
2012G064	24/6/2012
2012G064	23/7/2012
2012G065	24/6/2012
2012G066	25/6/2012
2012G066	7/7/2012
2012G066	18/7/2012
2012G066	30/7/2012
2012G067	28/6/2012
2012G068	29/6/2012
2012G068	9/7/2012
2012G069	29/6/2012
2012G069	9/7/2012
2012G069	19/7/2012
2012G069	30/7/2012
2012G069	11/8/2012
2012G070	2/7/2012
2012G071	2/7/2012
2012G071	12/7/2012
2012G071	23/7/2012
2012G072	9/7/2012
2012G072	19/7/2012
2012G073	14/7/2012
2012G073	24/7/2012
2012G073	5/8/2012
2012G074	19/7/2012
2012G074	2/9/2012
2012G075	19/7/2012
2012G075	19/7/2012
2012G076	19/7/2012
2012G076	1/8/2012

2012G076	13/8/2012
2012G077	20/7/2012
2012G078	21/7/2012
2012G078	1/8/2012
2012G078	12/8/2012
2012G079	21/7/2012
2012G079	3/8/2012
2012G079	15/8/2012
2012G079	27/8/2012
2012G080	23/7/2012
2012G080	4/8/2012
2012G080	15/8/2012
2012G080	26/8/2012
2012G080	5/9/2012
2012G080	17/9/2012
2012G081	24/7/2012
2012G082	25/7/2012
2012G083	26/7/2012
2012G083	7/8/2012
2012G083	18/8/2012
2012G083	28/8/2012
2012G083	7/9/2012
2012G083	17/9/2012
2012G083	27/9/2012
2012G083	7/10/2012
2012G084	26/7/2012
2012G084	5/8/2012
2012G084	15/8/2012
2012G084	26/8/2012
2012G085	7/8/2012
2012G085	14/8/2012
2012G085	25/8/2012
2012G085	6/9/2012
2012G086	3/8/2012
2012G087	3/8/2012
2012G088	3/8/2012
2012G088	15/8/2012
2012G088	26/8/2012
2012G088	6/9/2012
2012G089	7/8/2012
2012G089	17/8/2012
2012G089	27/8/2012
2012G089	6/9/2012
2012G089	17/9/2012
2012G089	3/10/2012
2012G090	10/8/2012

2012G090	21/8/2012
2012G090	2/9/2012
2012G090	22/9/2012
2012G090	4/10/2012
2012G091	18/8/2012
2012G091	29/8/2012
2012G092	19/8/2012
2012G092	11/9/2012
2012G092	21/9/2012
2012G092	23/10/2012
2012G092	2/11/2012
2012G093	28/8/2012
2012G093	10/9/2012
2012G094	9/9/2012
2012G095	15/9/2012
2012G096	17/9/2012
2012G096	27/9/2012
2012G096	6/10/2012
2012G096	19/10/2012
2012G097	27/9/2012
2012G098	5/10/2012
2012G098	15/10/2012
2012G098	4/11/2012
2012G098	16/11/2012
1993G015	15/5/2013
1993G015	28/5/2013
1993G026	19/4/2013
1993G026	12/5/2013
1993G026	11/6/2013
1993G026	19/6/2013
1993G026	29/6/2013
1993G026	8/7/2013
1993G026	18/7/2013
1993G026	21/5/2013
1993G077	30/4/2013
1993G077	18/6/2013
1994G060	6/5/2013
1994G060	18/5/2013
1994G060	29/5/2013
1994G060	9/6/2013
1994G060	19/6/2013
1994G060	30/6/2013
1994G060	11/7/2013
1994G060	22/7/2013
1994G060	3/8/2013
1995G023	11/5/2013

1995G023	21/5/2013
1995G023	31/5/2013
1995G023	9/6/2013
1995G023	18/6/2013
1995G023	27/6/2013
1995G023	7/7/2013
1996G011	1/7/2013
1996G011	11/7/2013
1996G011	22/7/2013
1996G011	1/8/2013
1996G011	22/8/2013
1996G028	7/6/2013
1996G028	17/6/2013
1996G028	28/6/2013
1996G028	8/7/2013
1996G028	27/7/2013
1996G028	7/8/2013
1998G005	28/4/2013
1998G005	8/5/2013
1998G005	18/5/2013
1998G005	8/6/2013
1998G005	18/6/2013
1998G005	28/6/2013
1999G005	9/5/2013
1999G005	18/5/2013
1999G005	28/5/2013
1999G005	6/6/2013
1999G016	28/4/2013
1999G016	9/5/2013
1999G016	11/5/2013
1999G016	31/5/2013
1999G016	10/6/2013
2002G003	27/3/2013
2002G003	6/4/2013
2002G003	15/4/2013
2002G003	24/4/2013
2002G003	4/5/2013
2002G003	13/5/2013
2002G006	5/5/2013
2002G006	16/5/2013
2002G006	26/5/2013
2002G006	5/6/2013
2002G006	24/6/2013
2002G006	14/7/2013
2002G006	15/5/2013
2003G014	24/7/2013

2003G014	4/8/2013
2003G014	16/8/2013
2003G014	27/8/2013
2003G014	20/9/2013
2004G002	24/7/2013
2004G002	22/8/2013
2004G002	31/8/2013
2004G002	18/9/2013
2004G010	29/6/2013
2004G018	4/5/2013
2004G018	14/5/2013
2004G018	25/5/2013
2004G018	3/6/2013
2004G018	12/6/2013
2004G018	22/6/2013
2004G018	1/7/2013
2004G018	21/7/2013
2004G036	7/7/2013
2004G036	16/7/2013
2004G036	26/7/2013
2004G036	4/8/2013
2004G036	13/8/2013
2004G036	24/8/2013
2005G008	23/5/2013
2005G008	2/6/2013
2005G008	12/6/2013
2005G008	22/6/2013
2005G008	13/7/2013
2005G008	24/7/2013
2006G003	29/3/2013
2006G022	22/5/2013
2006G029	9/6/2013
2006G029	1/7/2013
2007G009	10/4/2013
2007G009	20/4/2013
2007G009	12/5/2013
2007G009	2/6/2013
2007G009	13/6/2013
2007G009	26/6/2013
2007G009	22/5/2013
2007G020	17/5/2013
2007G020	27/5/2013
2007G020	6/6/2013
2007G020	15/6/2013
2007G020	5/7/2013
2007G020	25/6/2013

2008G004	1/7/2013
2008G004	12/7/2013
2008G004	24/7/2013
2008G004	3/8/2013
2008G004	24/8/2013
2008G004	3/9/2013
2008G036	19/4/2013
2008G036	28/4/2013
2008G036	7/5/2013
2008G036	18/5/2013
2008G036	17/5/2013
2008G045	24/4/2013
2008G045	5/5/2013
2008G045	15/5/2013
2008G045	26/5/2013
2008G045	4/6/2013
2008G045	15/6/2013
2008G045	26/6/2013
2008G046	24/4/2013
2008G046	4/5/2013
2008G046	13/5/2013
2008G046	12/6/2013
2008G046	22/6/2013
2008G046	23/7/2013
2008G046	13/7/2013
2008G056	28/5/2013
2008G056	8/6/2013
2008G056	19/6/2013
2008G056	29/6/2013
2008G056	10/7/2013
2008G056	20/7/2013
2008G060	6/7/2013
2008G060	26/7/2013
2008G060	5/8/2013
2008G060	16/7/2013
2008G068	8/4/2013
2008G068	20/4/2013
2008G068	1/5/2013
2008G068	11/5/2013
2008G068	21/5/2013
2008G068	9/6/2013
2008G068	18/6/2013
2008G068	28/6/2013
2008G068	9/7/2013
2009G002	29/1/2013
2009G002	10/2/2013

2009G002	22/2/2013
2009G002	4/3/2013
2009G002	16/3/2013
2009G007	10/3/2013
2009G007	30/4/2013
2009G010	31/3/2013
2009G010	10/4/2013
2009G010	21/4/2013
2009G010	1/5/2013
2009G010	19/5/2013
2009G010	29/5/2013
2009G010	8/6/2013
2009G010	18/6/2013
2009G020	15/4/2013
2009G020	25/4/2013
2009G020	5/5/2013
2009G020	14/5/2013
2009G020	24/5/2013
2009G020	3/6/2013
2009G020	13/6/2013
2009G020	24/6/2013
2009G020	4/7/2013
2009G037	30/5/2013
2009G037	9/6/2013
2009G037	20/6/2013
2009G037	1/7/2013
2009G037	11/7/2013
2009G037	2/8/2013
2009G040	7/6/2013
2009G040	16/6/2013
2009G040	29/6/2013
2009G040	9/7/2013
2009G040	29/7/2013
2009G040	28/6/2013
2009G044	1/7/2013
2009G044	12/7/2013
2009G044	23/7/2013
2009G044	23/8/2013
2009G044	17/9/2013
2009G053	20/8/2013
2009G053	30/8/2013
2009G053	20/9/2013
2009G053	9/10/2013
2009G053	19/10/2013
2009G053	10/11/2013
2009G053	20/11/2013

2009G056	27/4/2013
2009G056	7/5/2013
2009G056	27/5/2013
2009G056	7/6/2013
2009G056	18/6/2013
2010G002	13/2/2013
2010G002	24/2/2013
2010G002	10/3/2013
2010G002	20/3/2013
2010G002	30/3/2013
2010G002	9/4/2013
2010G002	19/4/2013
2010G002	29/4/2013
2010G002	9/5/2013
2010G004	13/2/2013
2010G004	8/3/2013
2010G004	30/3/2013
2010G005	21/3/2013
2010G005	2/4/2013
2010G005	22/4/2013
2010G005	2/5/2013
2010G005	12/5/2013
2010G005	1/6/2013
2010G014	22/1/2013
2010G014	12/2/2013
2010G014	23/2/2013
2010G014	25/3/2013
2010G017	18/5/2013
2010G017	28/5/2013
2010G017	6/6/2013
2010G017	15/6/2013
2010G017	25/6/2013
2010G017	5/7/2013
2010G017	15/7/2013
2010G018	6/3/2013
2010G018	17/3/2013
2010G018	28/3/2013
2010G018	8/4/2013
2010G018	29/4/2013
2010G022	10/3/2013
2010G022	20/3/2013
2010G022	31/3/2013
2010G022	10/4/2013
2010G022	19/4/2013
2010G022	9/5/2013
2010G025	16/3/2013

2010G025	28/3/2013
2010G025	7/4/2013
2010G025	17/4/2013
2010G025	25/4/2013
2010G025	27/4/2013
2010G025	6/5/2013
2010G025	17/5/2013
2010G028	31/3/2013
2010G028	11/4/2013
2010G028	14/5/2013
2010G028	24/5/2013
2010G028	3/6/2013
2010G028	14/6/2013
2010G032	9/5/2013
2010G032	19/5/2013
2010G032	28/5/2013
2010G032	6/6/2013
2010G032	17/6/2013
2010G032	27/6/2013
2010G032	8/7/2013
2010G032	18/7/2013
2010G032	29/7/2013
2010G036	13/3/2013
2010G040	11/3/2013
2010G040	23/3/2013
2010G040	3/4/2013
2010G040	14/4/2013
2010G040	6/5/2013
2010G040	17/5/2013
2010G043	14/4/2013
2010G043	25/4/2013
2010G043	5/5/2013
2010G043	15/5/2013
2010G043	26/5/2013
2010G043	6/6/2013
2010G043	16/6/2013
2010G043	28/6/2013
2010G056	29/5/2013
2010G056	10/6/2013
2010G056	21/6/2013
2010G056	1/7/2013
2010G056	11/7/2013
2010G056	22/7/2013
2010G056	5/8/2013
2010G057	4/5/2013
2010G057	27/5/2013

2010G057	15/6/2013
2010G057	5/7/2013
2010G057	15/7/2013
2010G057	15/7/2013
2010G057	25/6/2013
2010G062	9/5/2013
2010G062	28/5/2013
2010G062	6/6/2013
2010G062	16/6/2013
2010G062	28/6/2013
2010G062	9/7/2013
2010G071	22/5/2013
2010G071	25/6/2013
2010G071	6/7/2013
2010G071	15/7/2013
2010G071	7/8/2013
2010G071	2/6/2013
2010G079	3/3/2013
2010G079	14/3/2013
2010G084	12/5/2013
2010G084	30/5/2013
2010G084	10/6/2013
2010G084	19/6/2013
2010G084	29/6/2013
2010G084	18/7/2013
2010G084	26/7/2013
2010G084	6/8/2013
2010G089	23/4/2013
2010G089	3/5/2013
2010G089	13/5/2013
2010G089	2/6/2013
2010G089	12/6/2013
2010G100	15/8/2013
2010G100	25/8/2013
2010G100	16/9/2013
2010G100	9/10/2013
2010G100	20/10/2013
2010G100	1/11/2013
2010G100	13/11/2013
2010G100	9/12/2013
2010G107	31/5/2013
2010G107	12/6/2013
2010G107	24/6/2013
2010G107	3/7/2013
2010G107	14/7/2013
2010G107	25/7/2013

2010G107	14/7/2013
2013G001	21/1/2013
2013G001	1/2/2013
2013G001	14/2/2013
2013G001	28/2/2013
2013G001	12/3/2013
2013G001	28/3/2013
2013G002	21/1/2013
2013G002	31/1/2013
2013G003	26/1/2013
2013G003	8/3/2013
2013G003	23/3/2013
2013G004	31/1/2013
2013G005	6/2/2013
2013G005	17/2/2013
2013G006	9/2/2013
2013G006	20/2/2013
2013G006	3/3/2013
2013G006	14/3/2013
2013G006	28/3/2013
2013G006	8/4/2013
2013G007	28/2/2013
2013G007	12/3/2013
2013G008	6/3/2013
2013G008	20/3/2013
2013G008	1/4/2013
2013G008	13/4/2013
2013G008	25/4/2013
2013G008	7/5/2013
2013G008	19/5/2013
2013G009	14/3/2013
2013G009	12/5/2013
2013G009	4/6/2013
2013G010	15/3/2013
2013G010	27/3/2013
2013G010	6/4/2013
2013G010	15/4/2013
2013G010	26/4/2013
2013G010	6/5/2013
2013G010	17/5/2013
2013G010	27/5/2013
2013G011	15/3/2013
2013G011	30/3/2013
2013G011	10/4/2013
2013G011	24/4/2013
2013G011	6/5/2013

2013G011	18/5/2013
2013G011	30/5/2013
2013G011	10/6/2013
2013G011	24/6/2013
2013G012	16/3/2013
2013G012	28/3/2013
2013G012	6/4/2013
2013G012	16/4/2013
2013G012	27/4/2013
2013G013	20/3/2013
2013G014	23/3/2013
2013G014	3/4/2013
2013G014	14/4/2013
2013G015	23/3/2013
2013G016	25/3/2013
2013G017	26/3/2013
2013G017	5/4/2013
2013G017	14/4/2013
2013G017	24/4/2013
2013G017	4/5/2013
2013G017	13/5/2013
2013G017	2/6/2013
2013G017	12/6/2013
2013G018	26/3/2013
2013G018	6/4/2013
2013G018	28/4/2013
2013G018	10/5/2013
2013G019	28/3/2013
2013G019	17/4/2013
2013G019	27/4/2013
2013G019	7/5/2013
2013G019	17/5/2013
2013G019	27/5/2013
2013G019	8/6/2013
2013G020	28/3/2013
2013G020	12/4/2013
2013G020	27/5/2013
2013G020	6/6/2013
2013G020	17/6/2013
2013G021	28/3/2013
2013G021	9/4/2013
2013G021	18/4/2013
2013G021	28/4/2013
2013G021	17/5/2013
2013G022	30/3/2013
2013G022	10/4/2013

2013G022	1/5/2013
2013G023	1/4/2013
2013G023	13/4/2013
2013G023	23/4/2013
2013G023	3/5/2013
2013G023	13/5/2013
2013G024	1/4/2013
2013G024	18/4/2013
2013G024	30/4/2013
2013G024	21/5/2013
2013G024	30/5/2013
2013G024	10/6/2013
2013G025	1/4/2013
2013G025	23/4/2013
2013G026	2/4/2013
2013G026	12/4/2013
2013G026	4/5/2013
2013G026	23/5/2013
2013G027	3/4/2013
2013G027	13/4/2013
2013G027	23/4/2013
2013G027	4/5/2013
2013G027	14/5/2013
2013G027	23/5/2013
2013G027	2/6/2013
2013G027	13/6/2013
2013G028	4/4/2013
2013G028	14/4/2013
2013G028	24/4/2013
2013G028	5/5/2013
2013G029	6/4/2013
2013G029	18/4/2013
2013G029	29/4/2013
2013G029	9/5/2013
2013G029	20/5/2013
2013G029	31/5/2013
2013G029	12/6/2013
2013G029	25/6/2013
2013G030	6/4/2013
2013G030	27/4/2013
2013G030	7/5/2013
2013G030	17/5/2013
2013G030	27/5/2013
2013G030	8/6/2013
2013G031	7/4/2013
2013G031	18/4/2013

2013G031	27/4/2013
2013G031	7/5/2013
2013G031	17/5/2013
2013G032	7/4/2013
2013G032	17/4/2013
2013G032	28/4/2013
2013G032	8/5/2013
2013G033	7/4/2013
2013G033	24/5/2013
2013G033	17/6/2013
2013G034	8/4/2013
2013G034	19/4/2013
2013G034	30/4/2013
2013G034	11/5/2013
2013G034	1/6/2013
2013G035	8/4/2013
2013G035	21/4/2013
2013G035	3/5/2013
2013G035	12/5/2013
2013G035	2/6/2013
2013G035	30/6/2013
2013G035	11/7/2013
2013G036	10/4/2013
2013G036	20/4/2013
2013G036	30/4/2013
2013G036	11/5/2013
2013G036	20/5/2013
2013G036	29/5/2013
2013G037	10/4/2013
2013G037	22/4/2013
2013G037	2/5/2013
2013G037	13/5/2013
2013G037	23/5/2013
2013G037	1/6/2013
2013G037	12/6/2013
2013G037	23/6/2013
2013G038	14/4/2013
2013G038	25/4/2013
2013G038	5/5/2013
2013G039	15/4/2013
2013G039	25/4/2013
2013G039	5/5/2013
2013G039	17/5/2013
2013G040	15/4/2013
2013G040	26/4/2013
2013G040	5/5/2013

2013G040	14/5/2013
2013G040	23/5/2013
2013G040	1/6/2013
2013G040	12/6/2013
2013G041	16/4/2013
2013G041	27/4/2013
2013G042	16/4/2013
2013G042	27/4/2013
2013G042	6/5/2013
2013G042	16/5/2013
2013G042	26/5/2013
2013G042	5/6/2013
2013G042	15/6/2013
2013G042	27/6/2013
2013G042	9/7/2013
2013G043	17/4/2013
2013G043	29/4/2013
2013G043	9/5/2013
2013G043	19/5/2013
2013G043	29/5/2013
2013G043	18/6/2013
2013G043	21/7/2013
2013G043	19/5/2013
2013G044	17/4/2013
2013G045	17/4/2013
2013G045	28/4/2013
2013G045	7/5/2013
2013G045	16/5/2013
2013G045	26/5/2013
2013G045	3/6/2013
2013G046	18/4/2013
2013G046	30/4/2013
2013G046	12/5/2013
2013G046	22/5/2013
2013G046	13/6/2013
2013G047	19/4/2013
2013G047	3/5/2013
2013G047	14/5/2013
2013G047	26/5/2013
2013G048	20/4/2013
2013G049	20/4/2013
2013G050	20/4/2013
2013G051	21/4/2013
2013G051	1/5/2013
2013G051	11/5/2013
2013G051	21/5/2013

2013G051	31/5/2013
2013G051	9/6/2013
2013G051	2/7/2013
2013G052	21/4/2013
2013G052	1/5/2013
2013G052	11/5/2013
2013G053	21/4/2013
2013G053	1/5/2013
2013G053	10/5/2013
2013G053	20/5/2013
2013G053	30/5/2013
2013G053	8/6/2013
2013G053	20/5/2013
2013G054	21/4/2013
2013G054	1/5/2013
2013G054	11/5/2013
2013G054	21/5/2013
2013G054	30/5/2013
2013G054	8/6/2013
2013G054	18/6/2013
2013G055	22/4/2013
2013G055	29/5/2013
2013G055	8/6/2013
2013G055	18/6/2013
2013G055	29/6/2013
2013G055	9/7/2013
2013G055	5/5/2013
2013G056	22/4/2013
2013G056	30/4/2013
2013G056	9/5/2013
2013G056	19/5/2013
2013G057	23/4/2013
2013G057	3/5/2013
2013G057	13/5/2013
2013G057	23/5/2013
2013G057	3/6/2013
2013G057	13/6/2013
2013G058	24/4/2013
2013G058	13/5/2013
2013G058	24/5/2013
2013G058	2/6/2013
2013G058	12/6/2013
2013G058	24/6/2013
2013G058	5/7/2013
2013G059	28/4/2013
2013G059	8/5/2013

2013G059	19/5/2013
2013G059	28/5/2013
2013G059	6/6/2013
2013G059	17/5/2013
2013G060	29/4/2013
2013G060	10/5/2013
2013G060	20/5/2013
2013G060	8/6/2013
2013G060	18/6/2013
2013G061	29/4/2013
2013G062	29/4/2013
2013G062	11/5/2013
2013G062	19/5/2013
2013G062	29/5/2013
2013G062	8/6/2013
2013G062	19/6/2013
2013G062	29/6/2013
2013G062	10/7/2013
2013G062	22/7/2013
2013G063	29/4/2013
2013G063	10/5/2013
2013G063	22/5/2013
2013G064	30/4/2013
2013G064	12/5/2013
2013G064	22/5/2013
2013G064	31/5/2013
2013G064	10/6/2013
2013G064	20/6/2013
2013G065	1/5/2013
2013G066	1/5/2013
2013G066	11/5/2013
2013G066	30/5/2013
2013G066	10/6/2013
2013G067	3/5/2013
2013G067	14/5/2013
2013G067	26/5/2013
2013G067	18/6/2013
2013G067	29/6/2013
2013G067	11/7/2013
2013G068	3/5/2013
2013G068	12/5/2013
2013G068	23/5/2013
2013G068	1/6/2013
2013G069	4/5/2013
2013G069	14/5/2013
2013G069	4/6/2013

2013G069	14/6/2013
2013G069	24/6/2013
2013G069	3/7/2013
2013G069	24/5/2013
2013G070	5/5/2013
2013G070	15/5/2013
2013G070	26/5/2013
2013G070	4/6/2013
2013G071	6/5/2013
2013G071	15/5/2013
2013G071	24/5/2013
2013G071	3/6/2013
2013G071	12/6/2013
2013G071	21/6/2013
2013G071	30/6/2013
2013G071	10/7/2013
2013G071	20/7/2013
2013G072	8/5/2013
2013G072	19/5/2013
2013G072	30/5/2013
2013G072	8/6/2013
2013G072	17/6/2013
2013G072	28/6/2013
2013G072	9/7/2013
2013G073	9/5/2013
2013G073	29/5/2013
2013G073	8/6/2013
2013G074	10/5/2013
2013G074	20/5/2013
2013G074	29/5/2013
2013G074	8/6/2013
2013G074	19/6/2013
2013G074	19/5/2013
2013G075	11/5/2013
2013G075	20/5/2013
2013G075	28/5/2013
2013G075	7/6/2013
2013G075	15/6/2013
2013G075	25/6/2013
2013G076	12/5/2013
2013G077	12/5/2013
2013G078	14/5/2013
2013G079	14/5/2013
2013G079	28/5/2013
2013G079	6/6/2013
2013G079	16/6/2013

2013G079	27/6/2013
2013G079	17/7/2013
2013G080	14/5/2013
2013G081	14/5/2013
2013G081	25/5/2013
2013G081	3/6/2013
2013G081	12/6/2013
2013G081	21/6/2013
2013G081	30/6/2013
2013G082	17/5/2013
2013G082	27/5/2013
2013G083	17/5/2013
2013G083	6/6/2013
2013G084	18/5/2013
2013G084	27/5/2013
2013G084	7/6/2013
2013G085	18/5/2013
2013G085	8/6/2013
2013G086	18/5/2013
2013G086	31/5/2013
2013G087	18/5/2013
2013G087	18/6/2013
2013G087	29/6/2013
2013G087	9/7/2013
2013G088	18/5/2013
2013G088	29/5/2013
2013G088	13/6/2013
2013G088	5/7/2013
2013G088	16/7/2013
2013G088	28/7/2013
2013G088	8/8/2013
2013G089	19/5/2013
2013G090	23/5/2013
2013G090	3/6/2013
2013G090	12/6/2013
2013G090	29/6/2013
2013G091	24/5/2013
2013G091	2/6/2013
2013G091	12/6/2013
2013G091	21/6/2013
2013G091	1/7/2013
2013G091	11/7/2013
2013G091	20/7/2013
2013G092	24/5/2013
2013G092	4/6/2013
2013G092	14/6/2013

2013G093	27/5/2013
2013G093	5/6/2013
2013G093	15/6/2013
2013G093	27/6/2013
2013G093	26/6/2013
2013G094	27/5/2013
2013G094	6/6/2013
2013G094	15/6/2013
2013G094	26/6/2013
2013G094	10/7/2013
2013G094	19/7/2013
2013G095	28/5/2013
2013G095	7/6/2013
2013G095	16/6/2013
2013G095	8/7/2013
2013G095	19/7/2013
2013G095	30/7/2013
2013G096	28/5/2013
2013G096	10/6/2013
2013G096	19/6/2013
2013G097	28/5/2013
2013G098	28/5/2013
2013G099	28/5/2013
2013G100	29/5/2013
2013G100	8/6/2013
2013G101	30/5/2013
2013G101	8/6/2013
2013G101	18/6/2013
2013G101	28/6/2013
2013G101	8/7/2013
2013G101	18/7/2013
2013G101	28/7/2013
2013G101	7/8/2013
2013G102	30/5/2013
2013G103	1/6/2013
2013G103	12/6/2013
2013G103	24/6/2013
2013G103	12/7/2013
2013G104	2/6/2013
2013G104	13/6/2013
2013G104	24/6/2013
2013G104	5/7/2013
2013G104	17/7/2013
2013G104	27/7/2013
2013G104	18/8/2013
2013G105	2/6/2013

2013G105	11/7/2013
2013G105	24/7/2013
2013G106	2/6/2013
2013G106	14/6/2013
2013G106	27/6/2013
2013G106	21/7/2013
2013G107	3/6/2013
2013G107	12/6/2013
2013G108	4/6/2013
2013G108	25/6/2013
2013G108	6/7/2013
2013G108	16/7/2013
2013G108	6/8/2013
2013G109	4/6/2013
2013G109	15/6/2013
2013G109	25/6/2013
2013G109	14/7/2013
2013G109	24/7/2013
2013G109	3/8/2013
2013G110	5/6/2013
2013G111	5/6/2013
2013G112	6/6/2013
2013G113	6/6/2013
2013G114	7/6/2013
2013G114	17/6/2013
2013G114	29/6/2013
2013G114	20/7/2013
2013G114	1/8/2013
2013G115	8/6/2013
2013G115	30/6/2013
2013G115	11/7/2013
2013G115	21/7/2013
2013G115	11/7/2013
2013G116	9/6/2013
2013G116	2/7/2013
2013G116	21/6/2013
2013G117	10/6/2013
2013G117	19/6/2013
2013G117	29/6/2013
2013G117	9/7/2013
2013G117	18/7/2013
2013G117	28/7/2013
2013G117	7/8/2013
2013G118	10/6/2013
2013G118	1/7/2013
2013G118	13/7/2013

2013G118	6/8/2013
2013G118	19/8/2013
2013G118	31/8/2013
2013G119	10/6/2013
2013G119	19/6/2013
2013G119	29/6/2013
2013G119	9/7/2013
2013G119	19/7/2013
2013G120	10/6/2013
2013G121	10/6/2013
2013G121	21/6/2013
2013G121	5/7/2013
2013G122	12/6/2013
2013G122	25/6/2013
2013G123	12/6/2013
2013G124	13/6/2013
2013G124	25/6/2013
2013G125	13/6/2013
2013G126	14/6/2013
2013G126	5/7/2013
2013G127	15/6/2013
2013G128	15/6/2013
2013G129	13/6/2013
2013G129	24/6/2013
2013G129	2/6/2013
2013G129	12/6/2013
2013G130	16/6/2013
2013G130	27/6/2013
2013G130	8/7/2013
2013G130	16/7/2013
2013G130	27/7/2013
2013G130	6/8/2013
2013G130	15/8/2013
2013G130	25/8/2013
2013G130	4/9/2013
2013G131	18/6/2013
2013G131	28/6/2013
2013G132	18/6/2013
2013G132	30/6/2013
2013G132	24/7/2013
2013G132	5/8/2013
2013G132	7/8/2013
2013G133	19/6/2013
2013G133	9/7/2013
2013G134	20/6/2013
2013G134	19/6/2013

2013G135	21/6/2013
2013G136	21/6/2013
2013G136	5/7/2013
2013G136	16/7/2013
2013G136	3/9/2013
2013G137	21/6/2013
2013G137	1/7/2013
2013G137	11/7/2013
2013G137	20/7/2013
2013G137	18/8/2013
2013G138	23/6/2013
2013G139	24/6/2013
2013G139	5/7/2013
2013G140	25/6/2013
2013G140	4/7/2013
2013G140	14/7/2013
2013G140	26/7/2013
2013G140	6/8/2013
2013G141	25/6/2013
2013G141	5/7/2013
2013G141	16/7/2013
2013G141	26/7/2013
2013G141	5/8/2013
2013G141	15/8/2013
2013G141	27/8/2013
2013G142	26/6/2013
2013G143	27/6/2013
2013G144	27/6/2013
2013G145	27/6/2013
2013G146	28/6/2013
2013G147	29/6/2013
2013G147	23/7/2013
2013G147	4/8/2013
2013G148	29/6/2013
2013G148	12/7/2013
2013G148	24/7/2013
2013G148	4/8/2013
2013G148	18/8/2013
2013G149	29/6/2013
2013G149	22/7/2013
2013G149	4/8/2013
2013G149	28/8/2013
2013G149	9/9/2013
2013G150	30/6/2013
2013G150	12/7/2013
2013G151	1/7/2013

2013G152	3/7/2013
2013G152	26/7/2013
2013G152	6/8/2013
2013G152	16/8/2013
2013G152	29/8/2013
2013G153	3/7/2013
2013G154	11/7/2013
2013G154	22/7/2013
2013G154	1/8/2013
2013G154	12/8/2013
2013G155	12/7/2013
2013G155	18/8/2013
2013G155	31/8/2013
2013G155	12/9/2013
2013G156	12/7/2013
2013G156	23/7/2013
2013G156	1/8/2013
2013G156	11/8/2013
2013G156	20/8/2013
2013G156	30/8/2013
2013G157	12/7/2013
2013G159	17/5/2013
2013G160	14/7/2013
2013G160	3/8/2013
2013G160	13/8/2013
2013G160	2/9/2013
2013G160	11/9/2013
2013G161	17/7/2013
2013G162	21/7/2013
2013G163	21/7/2013
2013G163	15/9/2013
2013G164	23/7/2013
2013G164	3/8/2013
2013G164	23/8/2013
2013G164	5/9/2013
2013G165	28/7/2013
2013G165	17/8/2013
2013G165	17/9/2013
2013G165	28/9/2013
2013G166	7/8/2013
2013G166	15/8/2013
2013G166	24/8/2013
2013G166	2/9/2013
2013G166	11/9/2013
2013G166	21/9/2013
2013G167	15/8/2013

2013G168	21/8/2013
2013G168	10/9/2013
2013G168	20/9/2013
2013G168	29/9/2013
2013G168	9/10/2013
2013G168	19/10/2013
2013G168	29/10/2013
2013G169	25/8/2013
2013G169	14/9/2013
2013G169	23/9/2013
2013G169	3/10/2013
2013G170	23/9/2013
2013G170	3/10/2013
2013G170	18/10/2013
2013G170	28/10/2013
2013G170	18/11/2013
2013G171	27/9/2013
2013G171	9/10/2013
2013G171	20/10/2013
2013G171	1/11/2013
2013G171	13/11/2013
2013G171	9/12/2013
2013G172	11/10/2013
2013G172	5/11/2013
2013G172	15/11/2013
2013G173	11/10/2013
2013G173	4/11/2013
1995G006	13/8/2014
1995G006	24/8/2014
1995G006	2/9/2014
1996G009	1/6/2014
1996G009	11/6/2014
1996G009	21/6/2014
1996G009	1/7/2014
1996G009	10/7/2014
1996G009	19/7/2014
1996G009	6/8/2014
1996G009	16/8/2014
1996G065	19/8/2014
1996G065	31/8/2014
1997G043	15/6/2014
1997G043	26/6/2014
1997G043	6/7/2014
1997G043	16/7/2014
1997G043	26/7/2014
1997G043	5/8/2014

1997G043	17/8/2014
1999G009	21/6/2014
1999G010	18/4/2014
1999G010	29/4/2014
1999G010	10/5/2014
1999G010	21/5/2014
1999G010	1/6/2014
1999G010	13/6/2014
1999G024	26/4/2014
1999G024	6/5/2014
1999G024	16/5/2014
1999G024	25/5/2014
1999G024	26/5/2014
1999G024	5/6/2014
1999G024	15/6/2014
1999G024	4/7/2014
2000G016	25/5/2014
2000G016	3/6/2014
2000G016	12/6/2014
2000G016	21/6/2014
2000G016	30/6/2014
2000G016	8/7/2014
2000G016	18/7/2014
2000G016	26/7/2014
2001G036	21/7/2014
2001G036	13/8/2014
2001G036	3/9/2014
2003G001	13/6/2014
2003G001	24/6/2014
2003G001	6/7/2014
2003G001	16/7/2014
2003G001	26/7/2014
2003G001	6/8/2014
2003G001	17/8/2014
2003G008	16/6/2014
2004G049	29/5/2014
2004G049	8/6/2014
2004G049	17/6/2014
2004G049	27/6/2014
2004G049	7/7/2014
2004G049	16/7/2014
2004G049	26/7/2014
2006G012	15/4/2014
2006G012	27/4/2014
2006G012	7/5/2014
2006G012	18/5/2014

2006G012	28/5/2014
2006G012	7/6/2014
2006G037	28/6/2014
2006G037	7/7/2014
2006G037	9/7/2014
2006G037	19/7/2014
2006G037	20/8/2014
2006G037	29/8/2014
2006G037	9/8/2014
2007G012	24/4/2014
2007G012	5/5/2014
2007G012	18/5/2014
2007G012	29/5/2014
2007G012	22/6/2014
2008G002	28/2/2014
2008G002	6/4/2014
2008G002	17/4/2014
2008G002	30/4/2014
2008G002	30/4/2014
2008G002	13/5/2014
2008G003	31/8/2014
2008G006	28/3/2014
2008G006	28/3/2014
2008G006	8/4/2014
2008G006	16/5/2014
2008G006	26/5/2014
2008G006	6/6/2014
2008G021	20/4/2014
2008G021	1/5/2014
2008G021	10/5/2014
2008G021	20/5/2014
2008G021	30/5/2014
2008G021	8/6/2014
2008G021	19/6/2014
2008G063	20/7/2014
2008G063	31/7/2014
2008G063	23/8/2014
2008G063	3/9/2014
2010G006	1/3/2014
2010G006	14/3/2014
2010G006	26/3/2014
2010G006	6/4/2014
2010G006	6/4/2014
2010G006	27/4/2014
2010G006	17/5/2014
2010G021	7/5/2014

2010G021	18/5/2014
2010G021	8/6/2014
2010G027	10/3/2014
2010G027	21/3/2014
2010G027	1/4/2014
2010G027	11/4/2014
2010G027	21/4/2014
2010G027	1/5/2014
2010G027	12/5/2014
2010G027	21/5/2014
2010G030	24/4/2014
2010G030	4/5/2014
2010G030	15/5/2014
2010G030	25/5/2014
2010G030	4/6/2014
2010G030	15/6/2014
2010G030	27/6/2014
2010G060	17/5/2014
2010G060	29/5/2014
2010G060	8/6/2014
2010G060	17/6/2014
2010G060	27/6/2014
2010G060	6/7/2014
2010G060	16/7/2014
2010G060	2/8/2014
2010G060	12/8/2014
2010G075	4/5/2014
2010G075	16/5/2014
2010G075	26/5/2014
2010G075	4/6/2014
2010G075	14/6/2014
2010G075	15/6/2014
2010G075	24/6/2014
2010G075	28/6/2014
2010G075	3/7/2014
2010G075	13/7/2014
2010G075	23/7/2014
2010G083	17/6/2014
2010G083	27/6/2014
2010G083	6/7/2014
2010G083	5/8/2014
2010G095	28/6/2014
2011G003	23/3/2014
2011G003	4/4/2014
2011G003	15/4/2014
2011G003	26/4/2014

2011G003	6/5/2014
2011G010	4/3/2014
2011G010	16/3/2014
2011G010	27/3/2014
2011G010	7/4/2014
2011G010	21/4/2014
2011G010	15/5/2014
2011G010	16/5/2014
2011G010	17/5/2014
2011G010	28/5/2014
2011G015	10/5/2014
2011G015	21/5/2014
2011G015	1/6/2014
2011G015	12/6/2014
2011G015	22/6/2014
2011G015	3/7/2014
2011G018	18/3/2014
2011G018	30/3/2014
2011G018	9/4/2014
2011G018	19/4/2014
2011G018	4/5/2014
2011G018	23/5/2014
2011G018	13/6/2014
2011G031	28/3/2014
2011G031	29/3/2014
2011G031	10/4/2014
2011G031	3/5/2014
2011G031	13/5/2014
2011G031	23/5/2014
2011G031	3/6/2014
2011G048	10/4/2014
2011G048	12/5/2014
2011G060	7/6/2014
2011G060	19/6/2014
2011G060	30/6/2014
2011G060	11/7/2014
2011G060	10/8/2014
2011G060	21/8/2014
2011G064	28/3/2014
2011G064	8/4/2014
2011G064	18/4/2014
2011G064	28/4/2014
2011G064	7/5/2014
2011G064	17/5/2014
2011G064	27/5/2014
2011G066	20/3/2014

2011G066	2/4/2014
2011G066	16/4/2014
2011G082	11/4/2014
2011G082	24/4/2014
2011G082	25/4/2014
2011G082	17/5/2014
2011G082	28/5/2014
2011G082	7/6/2014
2011G082	18/6/2014
2014G001	8/1/2014
2014G001	4/2/2014
2014G001	28/3/2014
2014G001	30/3/2014
2014G001	13/4/2014
2014G002	8/1/2014
2014G002	30/3/2014
2014G003	13/1/2014
2014G003	25/1/2014
2014G003	7/2/2014
2014G004	21/1/2014
2014G004	6/2/2014
2014G005	21/1/2014
2014G006	31/1/2014
2014G006	15/2/2014
2014G006	28/2/2014
2014G006	15/3/2014
2014G006	29/3/2014
2014G007	10/2/2014
2014G007	21/2/2014
2014G008	15/2/2014
2014G008	1/3/2014
2014G008	9/4/2014
2014G008	20/4/2014
2014G009	24/2/2014
2014G009	8/3/2014
2014G009	20/3/2014
2014G009	21/3/2014
2014G009	23/3/2014
2014G009	4/4/2014
2014G009	14/4/2014
2014G009	14/4/2014
2014G009	25/4/2014
2014G010	25/2/2014
2014G010	7/3/2014
2014G011	9/3/2014
2014G011	24/3/2014

2014G011	4/4/2014
2014G011	16/4/2014
2014G011	27/4/2014
2014G012	14/3/2014
2014G012	6/4/2014
2014G012	17/4/2014
2014G012	30/4/2014
2014G013	17/3/2014
2014G013	28/3/2014
2014G013	7/4/2014
2014G013	18/4/2014
2014G013	7/5/2014
2014G014	17/3/2014
2014G015	18/3/2014
2014G015	1/4/2014
2014G015	12/4/2014
2014G015	25/4/2014
2014G015	6/5/2014
2014G015	16/5/2014
2014G015	28/5/2014
2014G015	8/6/2014
2014G016	18/3/2014
2014G016	11/4/2014
2014G016	3/5/2014
2014G016	16/5/2014
2014G017	20/3/2014
2014G017	5/5/2014
2014G018	21/3/2014
2014G018	2/4/2014
2014G018	12/4/2014
2014G018	23/4/2014
2014G018	14/5/2014
2014G019	22/3/2014
2014G020	23/3/2014
2014G020	4/4/2014
2014G020	14/4/2014
2014G020	25/4/2014
2014G020	5/5/2014
2014G020	13/5/2014
2014G020	23/5/2014
2014G021	26/3/2014
2014G021	6/4/2014
2014G021	18/4/2014
2014G022	26/3/2014
2014G023	31/3/2014
2014G023	11/4/2014

2014G023	4/5/2014
2014G023	14/5/2014
2014G023	23/5/2014
2014G023	1/6/2014
2014G023	12/6/2014
2014G024	2/4/2014
2014G025	2/4/2014
2014G026	3/4/2014
2014G026	17/4/2014
2014G026	28/4/2014
2014G026	18/5/2014
2014G026	18/5/2014
2014G026	9/6/2014
2014G027	5/4/2014
2014G027	17/4/2014
2014G027	29/4/2014
2014G027	10/5/2014
2014G027	20/5/2014
2014G027	31/5/2014
2014G027	11/6/2014
2014G028	6/4/2014
2014G028	16/4/2014
2014G028	19/4/2014
2014G028	30/4/2014
2014G028	17/5/2014
2014G028	26/5/2014
2014G028	5/6/2014
2014G029	7/4/2014
2014G029	19/4/2014
2014G029	30/4/2014
2014G030	7/4/2014
2014G031	9/4/2014
2014G032	10/4/2014
2014G032	22/4/2014
2014G032	2/5/2014
2014G032	11/5/2014
2014G032	20/5/2014
2014G032	29/5/2014
2014G032	8/6/2014
2014G033	12/4/2014
2014G033	23/4/2014
2014G033	4/5/2014
2014G034	14/4/2014
2014G034	25/4/2014
2014G034	5/5/2014
2014G034	15/5/2014

2014G034	25/5/2014
2014G034	4/6/2014
2014G034	14/6/2014
2014G034	24/6/2014
2014G034	4/7/2014
2014G035	14/4/2014
2014G035	28/4/2014
2014G035	9/5/2014
2014G035	20/5/2014
2014G036	15/4/2014
2014G036	28/4/2014
2014G036	13/5/2014
2014G036	23/5/2014
2014G036	3/6/2014
2014G036	14/6/2014
2014G036	15/6/2014
2014G036	27/6/2014
2014G036	10/7/2014
2014G037	16/4/2014
2014G037	27/4/2014
2014G038	16/4/2014
2014G038	27/4/2014
2014G038	6/5/2014
2014G038	16/5/2014
2014G038	26/5/2014
2014G038	5/6/2014
2014G038	15/6/2014
2014G039	17/4/2014
2014G039	26/4/2014
2014G039	6/5/2014
2014G039	16/5/2014
2014G040	20/4/2014
2014G040	1/5/2014
2014G040	13/5/2014
2014G041	21/4/2014
2014G041	30/4/2014
2014G041	1/5/2014
2014G041	10/5/2014
2014G041	20/5/2014
2014G041	30/5/2014
2014G041	7/6/2014
2014G041	17/6/2014
2014G042	21/4/2014
2014G042	1/5/2014
2014G042	11/5/2014
2014G042	21/5/2014

2014G042	12/6/2014
2014G043	23/4/2014
2014G043	3/5/2014
2014G043	23/5/2014
2014G043	1/6/2014
2014G043	12/6/2014
2014G043	23/6/2014
2014G044	24/4/2014
2014G044	6/5/2014
2014G045	27/4/2014
2014G045	8/5/2014
2014G045	18/5/2014
2014G045	30/5/2014
2014G046	27/4/2014
2014G046	21/5/2014
2014G047	28/4/2014
2014G047	9/5/2014
2014G047	20/5/2014
2014G047	12/6/2014
2014G048	30/4/2014
2014G048	12/5/2014
2014G049	3/5/2014
2014G049	15/5/2014
2014G049	26/5/2014
2014G049	11/6/2014
2014G050	4/5/2014
2014G050	16/5/2014
2014G050	26/5/2014
2014G050	7/6/2014
2014G050	16/6/2014
2014G050	28/6/2014
2014G051	4/5/2014
2014G051	16/5/2014
2014G051	28/5/2014
2014G051	17/6/2014
2014G051	28/6/2014
2014G052	5/5/2014
2014G052	15/5/2014
2014G052	23/5/2014
2014G052	11/6/2014
2014G052	23/6/2014
2014G053	6/5/2014
2014G053	17/5/2014
2014G053	28/5/2014
2014G053	10/6/2014
2014G053	22/6/2014

2014G053	4/7/2014
2014G053	16/7/2014
2014G054	6/5/2014
2014G054	16/5/2014
2014G054	28/5/2014
2014G054	7/6/2014
2014G054	18/6/2014
2014G055	8/5/2014
2014G055	7/6/2014
2014G056	8/5/2014
2014G057	10/5/2014
2014G057	23/5/2014
2014G057	3/6/2014
2014G057	14/6/2014
2014G057	15/6/2014
2014G057	19/7/2014
2014G058	13/5/2014
2014G058	25/5/2014
2014G058	6/6/2014
2014G058	19/6/2014
2014G058	29/6/2014
2014G058	12/7/2014
2014G059	13/5/2014
2014G060	15/5/2014
2014G060	25/5/2014
2014G060	21/7/2014
2014G061	15/5/2014
2014G061	26/5/2014
2014G061	16/6/2014
2014G061	29/6/2014
2014G062	16/5/2014
2014G062	26/5/2014
2014G063	16/5/2014
2014G063	28/5/2014
2014G063	9/6/2014
2014G064	17/5/2014
2014G065	17/5/2014
2014G066	17/5/2014
2014G066	29/5/2014
2014G066	8/6/2014
2014G066	10/6/2014
2014G066	20/6/2014
2014G066	30/6/2014
2014G066	11/7/2014
2014G067	21/5/2014
2014G067	31/5/2014

2014G067	9/6/2014
2014G068	24/5/2014
2014G068	4/6/2014
2014G068	14/6/2014
2014G068	24/6/2014
2014G069	24/5/2014
2014G069	4/6/2014
2014G069	15/6/2014
2014G069	27/6/2014
2014G069	11/7/2014
2014G070	25/5/2014
2014G071	25/5/2014
2014G071	5/6/2014
2014G071	16/6/2014
2014G071	27/6/2014
2014G071	6/7/2014
2014G072	26/5/2014
2014G072	16/6/2014
2014G072	28/6/2014
2014G072	7/7/2014
2014G072	18/7/2014
2014G073	28/5/2014
2014G073	10/6/2014
2014G073	21/6/2014
2014G073	3/7/2014
2014G073	14/7/2014
2014G073	24/7/2014
2014G073	4/8/2014
2014G073	15/8/2014
2014G074	9/6/2014
2014G074	19/6/2014
2014G075	29/5/2014
2014G076	1/6/2014
2014G076	12/6/2014
2014G078	2/6/2014
2014G078	14/6/2014
2014G078	26/6/2014
2014G079	5/6/2014
2014G079	19/6/2014
2014G079	29/6/2014
2014G079	12/7/2014
2014G080	7/6/2014
2014G081	7/6/2014
2014G081	18/6/2014
2014G081	28/6/2014
2014G081	8/7/2014

2014G081	18/7/2014
2014G081	28/7/2014
2014G081	8/8/2014
2014G082	7/6/2014
2014G082	19/6/2014
2014G083	8/6/2014
2014G083	21/6/2014
2014G083	2/7/2014
2014G083	15/7/2014
2014G083	25/7/2014
2014G083	5/8/2014
2014G084	8/6/2014
2014G084	19/6/2014
2014G084	29/6/2014
2014G084	8/7/2014
2014G084	19/7/2014
2014G084	30/7/2014
2014G085	12/6/2014
2014G086	14/6/2014
2014G086	4/7/2014
2014G086	14/7/2014
2014G086	23/7/2014
2014G086	2/8/2014
2014G087	15/6/2014
2014G087	18/7/2014
2014G087	9/8/2014
2014G088	17/6/2014
2014G088	28/6/2014
2014G089	18/6/2014
2014G089	29/6/2014
2014G089	8/7/2014
2014G090	20/6/2014
2014G090	30/6/2014
2014G090	10/7/2014
2014G090	19/7/2014
2014G090	29/7/2014
2014G090	7/8/2014
2014G090	17/8/2014
2014G091	21/6/2014
2014G091	14/7/2014
2014G092	21/6/2014
2014G092	2/7/2014
2014G093	17/6/2014
2014G093	8/7/2014
2014G093	18/7/2014
2014G093	29/7/2014

2014G093	9/8/2014
2014G094	26/6/2014
2014G094	7/7/2014
2014G094	20/8/2014
2014G095	29/6/2014
2014G096	7/7/2014
2014G096	18/7/2014
2014G096	7/8/2014
2014G096	17/8/2014
2014G096	27/8/2014
2014G097	12/7/2014
2014G097	23/7/2014
2014G097	13/8/2014
2014G097	24/8/2014
2014G098	12/7/2014
2014G098	1/8/2014
2014G098	11/8/2014
2014G098	21/8/2014
2014G098	31/8/2014
2014G099	13/7/2014
2014G100	15/7/2014
2014G100	13/8/2014
2014G101	15/7/2014
2014G101	26/7/2014
2014G101	6/8/2014
2014G102	18/7/2014
2014G102	27/7/2014
2014G102	8/8/2014
2014G102	18/8/2014
2014G103	20/7/2014
2014G103	31/7/2014
2014G103	13/8/2014
2014G104	21/7/2014
2014G104	1/8/2014
2014G104	13/8/2014
2014G105	27/7/2014
2014G105	6/8/2014
2014G105	18/8/2014
2014G106	28/7/2014
2014G106	7/8/2014
2014G107	28/7/2014
2014G107	20/8/2014
2014G108	28/7/2014
2014G108	7/8/2014
2014G108	16/8/2014
2014G108	25/8/2014

2014G109	30/7/2014
2014G109	18/8/2014
2014G109	27/8/2014
2014G109	5/9/2014
2014G110	2/8/2014
2014G111	6/8/2014
2014G111	19/8/2014
2014G112	8/8/2014
2014G112	19/8/2014
2014G112	29/8/2014
2014G113	9/8/2014
2014G113	20/8/2014
2014G113	30/8/2014
2014G114	10/8/2014
2014G115	12/8/2014
2014G115	22/8/2014
2014G115	1/9/2014
2014G116	14/8/2014
2014G116	24/8/2014
2014G116	4/9/2014
2014G117	16/8/2014
2014G118	18/8/2014
2014G119	21/8/2014
2014G120	26/8/2014
2014G120	6/9/2014
2014G121	28/8/2014
2014G122	30/8/2014
2014G123	30/8/2014
2014G124	31/8/2014
2014G125	4/9/2014
2014G126	5/9/2014
1993G122	29/6/2015
1993G122	9/7/2015
1993G122	19/7/2015
1993G122	30/7/2015
1993G122	9/8/2015
1993G122	20/8/2015
1994G002	17/7/2015
1994G002	27/7/2015
1994G002	6/8/2015
1994G002	5/9/2015
1994G002	15/9/2015
1994G002	26/9/2015
1995G015	16/7/2015
1995G015	27/7/2015
1995G024	24/4/2015

1995G024	7/5/2015
1995G024	27/5/2015
1995G024	16/6/2015
1995G024	26/6/2015
1995G024	6/7/2015
1995G095	2/9/2015
1995G095	12/9/2015
1995G095	22/9/2015
1995G095	22/9/2015
1995G095	2/10/2015
1995G095	25/10/2015
1998G020	19/5/2015
1998G020	16/6/2015
1998G020	26/6/2015
1998G020	16/7/2015
1998G020	28/7/2015
1998G030	25/8/2015
1998G030	12/9/2015
2000G024	12/7/2015
2000G024	1/8/2015
2000G024	10/8/2015
2000G024	20/8/2015
2000G024	9/9/2015
2001G005	1/3/2015
2001G005	6/4/2015
2001G005	20/5/2015
2006G015	16/5/2015
2006G015	27/5/2015
2006G015	6/6/2015
2006G015	16/6/2015
2006G015	26/6/2015
2006G015	6/7/2015
2006G034	21/6/2015
2006G034	11/7/2015
2006G034	21/7/2015
2007G032	7/7/2015
2007G032	19/7/2015
2007G032	15/8/2015
2008G012	21/3/2015
2008G012	13/4/2015
2008G012	25/4/2015
2008G012	30/5/2015
2008G012	23/6/2015
2008G050	24/5/2015
2008G050	4/6/2015
2008G050	12/6/2015

2008G050	21/6/2015
2008G050	9/7/2015
2008G050	19/7/2015
2008G050	29/7/2015
2009G008	30/7/2015
2009G014	27/2/2015
2009G014	10/4/2015
2009G029	3/5/2015
2009G029	14/5/2015
2009G029	24/5/2015
2009G029	4/6/2015
2009G029	13/6/2015
2009G029	25/6/2015
2009G030	13/6/2015
2009G030	24/6/2015
2009G030	15/7/2015
2009G030	27/7/2015
2009G030	8/8/2015
2009G035	12/5/2015
2009G035	1/7/2015
2009G035	21/7/2015
2009G035	2/8/2015
2009G046	4/9/2015
2009G047	15/7/2015
2009G059	7/7/2015
2009G059	18/7/2015
2009G059	29/7/2015
2009G059	8/8/2015
2009G059	19/8/2015
2009G059	29/8/2015
2010G010	31/3/2015
2010G010	10/4/2015
2010G010	2/5/2015
2010G010	2/6/2015
2010G015	9/3/2015
2010G015	20/3/2015
2010G015	31/3/2015
2010G015	10/4/2015
2010G015	21/4/2015
2010G015	3/5/2015
2010G023	25/6/2015
2010G023	4/7/2015
2010G023	14/7/2015
2010G023	24/7/2015
2010G053	6/6/2015
2010G095	26/5/2015

2010G095	2/7/2015
2010G108	18/6/2015
2010G108	30/6/2015
2010G108	11/7/2015
2010G108	24/7/2015
2010G108	3/8/2015
2010G108	26/8/2015
2011G023	13/4/2015
2011G023	3/5/2015
2011G023	12/5/2015
2011G023	21/5/2015
2011G023	31/5/2015
2011G040	17/5/2015
2011G040	27/5/2015
2011G040	14/6/2015
2011G040	23/6/2015
2011G040	12/7/2015
2011G040	21/7/2015
2011G040	31/7/2015
2011G044	25/4/2015
2011G044	27/5/2015
2011G044	7/6/2015
2011G044	17/6/2015
2011G044	28/6/2015
2011G044	9/7/2015
2011G044	20/7/2015
2011G050	26/7/2015
2011G050	16/8/2015
2011G050	4/9/2015
2011G050	14/9/2015
2011G050	24/9/2015
2011G050	5/10/2015
2011G054	18/5/2015
2011G054	27/5/2015
2011G054	7/6/2015
2011G054	16/6/2015
2011G054	25/6/2015
2011G054	5/7/2015
2011G054	15/7/2015
2011G054	3/8/2015
2011G059	25/8/2015
2012G002	18/3/2015
2012G002	2/6/2015
2012G002	12/6/2015
2012G002	23/6/2015
2012G002	3/7/2015

2012G002	14/7/2015
2012G002	29/7/2015
2012G003	7/3/2015
2012G003	21/3/2015
2012G003	1/4/2015
2012G003	13/4/2015
2012G012	1/4/2015
2012G012	12/4/2015
2012G012	25/4/2015
2012G014	31/3/2015
2012G014	8/5/2015
2012G014	20/5/2015
2012G014	1/6/2015
2012G014	12/6/2015
2012G021	21/3/2015
2012G021	4/5/2015
2012G021	5/6/2015
2012G022	22/5/2015
2012G029	6/4/2015
2012G029	15/4/2015
2012G029	25/4/2015
2012G029	6/5/2015
2012G036	24/4/2015
2012G036	6/5/2015
2012G036	19/5/2015
2012G036	1/6/2015
2012G036	12/6/2015
2012G036	23/6/2015
2012G036	5/7/2015
2012G036	16/7/2015
2012G036	28/7/2015
2012G088	22/7/2015
2012G088	20/8/2015
2012G088	9/9/2015
2012G088	19/9/2015
2013G062	28/6/2015
2013G062	8/7/2015
2013G062	29/7/2015
2013G062	7/8/2015
2013G062	17/8/2015
2013G062	29/8/2015
2014G062	17/6/2015
2015G001	29/1/2015
2015G001	10/2/2015
2015G001	20/2/2015
2015G001	7/3/2015

2015G001	20/3/2015
2015G002	1/2/2015
2015G002	14/2/2015
2015G002	13/3/2015
2015G002	26/3/2015
2015G002	9/4/2015
2015G003	20/2/2015
2015G003	1/3/2015
2015G003	13/3/2015
2015G003	26/3/2015
2015G004	3/3/2015
2015G004	12/3/2015
2015G004	2/4/2015
2015G005	4/3/2015
2015G006	8/3/2015
2015G006	23/3/2015
2015G007	9/3/2015
2015G007	30/3/2015
2015G007	28/4/2015
2015G008	13/3/2015
2015G008	24/3/2015
2015G008	7/5/2015
2015G008	17/5/2015
2015G009	19/3/2015
2015G009	31/3/2015
2015G010	21/3/2015
2015G010	12/4/2015
2015G010	24/4/2015
2015G011	24/3/2015
2015G011	5/4/2015
2015G012	29/3/2015
2015G012	17/5/2015
2015G012	28/5/2015
2015G013	29/3/2015
2015G014	30/3/2015
2015G014	12/4/2015
2015G014	23/5/2015
2015G015	4/4/2015
2015G016	5/4/2015
2015G016	16/4/2015
2015G017	6/4/2015
2015G017	16/4/2015
2015G017	28/4/2015
2015G017	18/5/2015
2015G018	13/4/2015
2015G018	6/5/2015

2015G018	26/5/2015
2015G018	7/6/2015
2015G018	17/6/2015
2015G018	6/7/2015
2015G019	19/4/2015
2015G019	30/4/2015
2015G020	25/4/2015
2015G020	26/5/2015
2015G020	5/6/2015
2015G020	25/6/2015
2015G021	25/4/2015
2015G021	9/6/2015
2015G022	30/4/2015
2015G022	13/5/2015
2015G022	23/5/2015
2015G022	3/6/2015
2015G022	13/6/2015
2015G022	23/6/2015
2015G022	3/7/2015
2015G022	14/7/2015
2015G022	24/7/2015
2015G022	4/8/2015
2015G023	30/4/2015
2015G023	10/5/2015
2015G023	31/5/2015
2015G023	30/6/2015
2015G023	9/7/2015
2015G023	19/7/2015
2015G024	1/5/2015
2015G024	2/6/2015
2015G024	12/6/2015
2015G024	2/7/2015
2015G025	23/5/2015
2015G025	4/6/2015
2015G025	25/6/2015
2015G025	6/7/2015
2015G025	7/8/2015
2015G026	12/5/2015
2015G026	2/6/2015
2015G026	11/6/2015
2015G026	21/6/2015
2015G026	3/7/2015
2015G026	10/7/2015
2015G027	13/5/2015
2015G027	16/6/2015
2015G027	26/6/2015

2015G027	7/7/2015
2015G027	19/7/2015
2015G028	17/5/2015
2015G028	1/6/2015
2015G028	5/7/2015
2015G029	18/5/2015
2015G029	15/6/2015
2015G030	18/5/2015
2015G030	31/5/2015
2015G030	11/6/2015
2015G030	22/6/2015
2015G030	4/7/2015
2015G030	14/7/2015
2015G030	24/7/2015
2015G030	4/8/2015
2015G030	15/8/2015
2015G031	19/5/2015
2015G031	29/5/2015
2015G031	7/6/2015
2015G031	17/6/2015
2015G031	7/7/2015
2015G031	18/7/2015
2015G032	19/5/2015
2015G033	20/5/2015
2015G033	3/6/2015
2015G033	28/6/2015
2015G033	10/7/2015
2015G033	23/7/2015
2015G033	7/8/2015
2015G034	25/5/2015
2015G034	4/6/2015
2015G034	25/6/2015
2015G034	6/7/2015
2015G035	27/5/2015
2015G035	20/6/2015
2015G035	30/6/2015
2015G035	14/7/2015
2015G036	1/6/2015
2015G036	1/7/2015
2015G036	11/7/2015
2015G036	1/8/2015
2015G036	21/7/2015
2015G037	1/6/2015
2015G037	12/6/2015
2015G037	22/6/2015
2015G037	21/7/2015

2015G037	31/7/2015
2015G037	11/8/2015
2015G037	30/8/2015
2015G038	1/6/2015
2015G038	30/6/2015
2015G039	1/6/2015
2015G039	2/7/2015
2015G040	2/6/2015
2015G040	5/6/2015
2015G041	5/6/2015
2015G041	26/6/2015
2015G041	7/7/2015
2015G041	18/7/2015
2015G042	5/6/2015
2015G042	14/6/2015
2015G042	3/7/2015
2015G042	19/7/2015
2015G042	11/8/2015
2015G043	6/6/2015
2015G043	16/6/2015
2015G043	25/6/2015
2015G043	5/7/2015
2015G043	16/7/2015
2015G043	26/7/2015
2015G043	7/8/2015
2015G043	7/8/2015
2015G044	17/5/2015
2015G044	6/6/2015
2015G044	17/6/2015
2015G044	26/6/2015
2015G044	6/7/2015
2015G044	26/7/2015
2015G044	5/8/2015
2015G044	16/8/2015
2015G045	6/6/2015
2015G045	16/6/2015
2015G045	25/6/2015
2015G045	7/7/2015
2015G045	18/7/2015
2015G045	29/7/2015
2015G046	8/6/2015
2015G046	20/6/2015
2015G046	2/7/2015
2015G046	12/7/2015
2015G046	5/8/2015
2015G046	16/8/2015

2015G046	27/8/2015
2015G046	8/9/2015
2015G047	9/6/2015
2015G047	2/7/2015
2015G048	11/6/2015
2015G048	21/6/2015
2015G048	10/7/2015
2015G048	19/7/2015
2015G048	29/7/2015
2015G048	17/8/2015
2015G049	11/6/2015
2015G049	3/7/2015
2015G049	14/7/2015
2015G049	24/7/2015
2015G049	3/8/2015
2015G049	14/8/2015
2015G049	13/7/2015
2015G050	11/6/2015
2015G050	19/6/2015
2015G050	28/6/2015
2015G050	7/7/2015
2015G050	15/7/2015
2015G050	25/7/2015
2015G050	4/8/2015
2015G051	12/6/2015
2015G051	1/7/2015
2015G051	11/7/2015
2015G051	20/7/2015
2015G051	31/7/2015
2015G051	10/8/2015
2015G052	22/6/2015
2015G053	12/6/2015
2015G053	12/6/2015
2015G053	16/6/2015
2015G053	22/6/2015
2015G053	12/7/2015
2015G053	22/7/2015
2015G053	2/8/2015
2015G054	13/6/2015
2015G055	14/6/2015
2015G055	27/6/2015
2015G055	27/6/2015
2015G055	17/7/2015
2015G055	28/7/2015
2015G055	7/8/2015
2015G056	14/6/2015

2015G056	26/6/2015
2015G056	7/7/2015
2015G056	18/7/2015
2015G056	28/7/2015
2015G056	29/8/2015
2015G056	7/8/2015
2015G058	16/6/2015
2015G058	29/6/2015
2015G058	12/7/2015
2015G058	24/8/2015
2015G059	17/6/2015
2015G059	11/8/2015
2015G060	17/6/2015
2015G060	26/6/2015
2015G060	5/7/2015
2015G061	18/6/2015
2015G061	8/7/2015
2015G061	22/7/2015
2015G062	19/6/2015
2015G062	23/6/2015
2015G062	18/7/2015
2015G063	20/6/2015
2015G063	30/6/2015
2015G063	11/7/2015
2015G063	23/7/2015
2015G063	2/8/2015
2015G063	23/8/2015
2015G064	20/6/2015
2015G066	23/6/2015
2015G066	4/7/2015
2015G066	14/7/2015
2015G066	25/7/2015
2015G066	4/8/2015
2015G066	28/8/2015
2015G067	26/6/2015
2015G069	26/6/2015
2015G069	9/7/2015
2015G069	19/7/2015
2015G069	9/8/2015
2015G069	20/8/2015
2015G069	31/8/2015
2015G070	29/6/2015
2015G071	30/6/2015
2015G072	30/6/2015
2015G073	4/7/2015
2015G073	15/7/2015

2015G073	27/7/2015
2015G074	4/7/2015
2015G074	16/7/2015
2015G074	26/7/2015
2015G074	6/8/2015
2015G074	30/8/2015
2015G074	10/9/2015
2015G074	2/10/2015
2015G074	12/10/2015
2015G074	22/10/2015
2015G075	4/7/2015
2015G075	17/7/2015
2015G075	29/7/2015
2015G076	4/7/2015
2015G076	15/7/2015
2015G076	26/7/2015
2015G076	15/8/2015
2015G076	2/9/2015
2015G077	4/7/2015
2015G077	26/7/2015
2015G077	4/8/2015
2015G077	14/8/2015
2015G078	5/7/2015
2015G079	5/7/2015
2015G079	16/7/2015
2015G080	5/7/2015
2015G080	15/7/2015
2015G080	25/7/2015
2015G080	3/8/2015
2015G080	12/8/2015
2015G080	21/8/2015
2015G080	1/9/2015
2015G081	5/7/2015
2015G081	29/7/2015
2015G081	8/8/2015
2015G081	19/8/2015
2015G081	28/8/2015
2015G081	7/9/2015
2015G081	17/9/2015
2015G081	27/9/2015
2015G082	5/7/2015
2015G083	6/7/2015
2015G084	19/7/2015
2015G084	30/7/2015
2015G084	9/8/2015
2015G084	19/8/2015

2015G085	7/7/2015
2015G085	7/7/2015
2015G085	29/7/2015
2015G086	7/7/2015
2015G086	7/8/2015
2015G086	17/8/2015
2015G086	26/8/2015
2015G086	5/9/2015
2015G086	16/9/2015
2015G086	27/9/2015
2015G087	7/7/2015
2015G087	17/7/2015
2015G087	28/7/2015
2015G087	7/8/2015
2015G087	17/8/2015
2015G087	7/9/2015
2015G087	20/9/2015
2015G087	30/9/2015
2015G088	8/7/2015
2015G088	18/7/2015
2015G088	5/8/2015
2015G089	9/7/2015
2015G089	20/7/2015
2015G090	9/7/2015
2015G091	9/7/2015
2015G091	19/7/2015
2015G091	30/7/2015
2015G091	19/8/2015
2015G091	30/8/2015
2015G092	10/7/2015
2015G092	19/7/2015
2015G092	29/7/2015
2015G092	8/8/2015
2015G093	11/7/2015
2015G094	12/7/2015
2015G094	23/7/2015
2015G094	16/8/2015
2015G094	26/8/2015
2015G095	12/7/2015
2015G095	21/7/2015
2015G095	2/8/2015
2015G096	12/7/2015
2015G097	14/7/2015
2015G098	16/7/2015
2015G098	29/7/2015
2015G099	17/7/2015

2015G099	27/7/2015
2015G099	31/7/2015
2015G099	5/8/2015
2015G099	10/8/2015
2015G099	15/8/2015
2015G099	24/8/2015
2015G099	3/9/2015
2015G100	18/7/2015
2015G100	1/9/2015
2015G101	19/7/2015
2015G101	30/7/2015
2015G102	21/7/2015
2015G103	21/7/2015
2015G104	22/7/2015
2015G104	2/8/2015
2015G105	22/7/2015
2015G105	3/8/2015
2015G106	23/7/2015
2015G106	3/8/2015
2015G107	29/7/2015
2015G107	7/8/2015
2015G107	7/9/2015
2015G108	31/7/2015
2015G109	31/7/2015
2015G109	11/8/2015
2015G110	31/7/2015
2015G111	31/7/2015
2015G111	12/8/2015
2015G111	21/8/2015
2015G111	31/8/2015
2015G111	10/9/2015
2015G111	20/9/2015
2015G112	1/8/2015
2015G112	11/8/2015
2015G112	3/9/2015
2015G113	2/8/2015
2015G113	24/8/2015
2015G114	3/8/2015
2015G115	5/8/2015
2015G116	8/8/2015
2015G116	19/8/2015
2015G117	8/8/2015
2015G117	21/8/2015
2015G117	2/10/2015
2015G118	10/8/2015
2015G118	20/8/2015

2015G118	31/8/2015
2015G118	10/9/2015
2015G118	20/9/2015
2015G118	1/10/2015
2015G118	12/10/2015
2015G119	11/8/2015
2015G119	22/8/2015
2015G119	1/9/2015
2015G119	21/9/2015
2015G119	1/10/2015
2015G120	12/8/2015
2015G120	26/8/2015
2015G120	5/9/2015
2015G120	14/9/2015
2015G120	24/9/2015
2015G120	5/10/2015
2015G121	16/8/2015
2015G122	16/8/2015
2015G122	26/8/2015
2015G122	7/9/2015
2015G122	17/9/2015
2015G123	18/8/2015
2015G124	17/8/2015
2015G124	21/8/2015
2015G124	2/9/2015
2015G124	9/9/2015
2015G125	21/8/2015
2015G126	24/8/2015
2015G126	4/9/2015
2015G126	14/9/2015
2015G127	30/8/2015
2015G127	9/9/2015
2015G128	31/8/2015
2015G128	11/9/2015
2015G128	23/9/2015
2015G129	8/9/2015
2015G130	26/9/2015
2015G130	19/10/2015
2015G131	8/10/2015
2015G132	26/6/2015
2015G133	13/9/2015
1994G060	8/7/2016
1994G060	19/7/2016
1994G060	29/7/2016
1994G060	8/8/2016
1994G060	9/8/2016

1994G060	20/8/2016
1994G060	31/8/2016
1997G018	24/4/2016
1997G032	13/8/2016
1997G032	24/8/2016
1997G032	3/9/2016
1997G032	13/9/2016
1998G005	16/5/2016
1998G005	28/5/2016
1998G005	7/6/2016
1998G005	17/6/2016
1998G005	26/6/2016
1998G005	6/7/2016
1998G043	25/5/2016
1999G005	24/3/2016
1999G005	13/4/2016
1999G005	12/5/2016
1999G005	22/5/2016
1999G015	16/6/2016
1999G015	23/7/2016
2000G010	8/6/2016
2000G010	19/6/2016
2000G010	4/7/2016
2000G010	15/7/2016
2000G010	27/7/2016
2000G010	5/8/2016
2000G010	15/8/2016
2003G004	20/5/2016
2003G004	20/6/2016
2003G004	30/6/2016
2003G004	20/7/2016
2004G009	16/5/2016
2004G009	28/5/2016
2004G009	9/6/2016
2004G009	21/6/2016
2004G009	1/7/2016
2004G009	22/7/2016
2004G009	12/8/2016
2004G035	28/8/2016
2004G053	15/7/2016
2004G053	16/8/2016
2004G056	20/7/2016
2004G056	31/8/2016
2004G057	8/8/2016
2004G057	18/8/2016
2004G057	10/9/2016

2004G059	6/7/2016
2004G059	16/7/2016
2004G059	17/8/2016
2004G059	6/9/2016
2006G025	14/5/2016
2006G025	19/6/2016
2006G025	28/7/2016
2007G005	22/3/2016
2007G005	1/4/2016
2007G005	10/4/2016
2007G005	20/4/2016
2007G005	30/4/2016
2007G005	9/5/2016
2007G005	18/5/2016
2007G015	4/5/2016
2007G015	15/5/2016
2007G015	26/5/2016
2007G015	5/6/2016
2007G015	26/6/2016
2007G015	6/7/2016
2007G015	17/7/2016
2007G020	20/5/2016
2007G020	8/6/2016
2007G020	27/6/2016
2007G020	6/7/2016
2007G030	7/7/2016
2007G030	18/7/2016
2007G030	28/7/2016
2007G030	6/8/2016
2007G030	5/9/2016
2007G036	27/5/2016
2008G036	11/3/2016
2008G036	21/3/2016
2008G036	31/3/2016
2008G036	10/4/2016
2008G036	29/4/2016
2008G049	13/5/2016
2008G067	8/7/2016
2008G067	17/7/2016
2008G067	5/8/2016
2008G067	14/8/2016
2008G067	2/9/2016
2008G068	30/6/2016
2008G068	20/7/2016
2008G068	29/7/2016
2008G068	7/8/2016

2008G069	7/7/2016
2009G001	18/3/2016
2009G001	29/3/2016
2009G001	8/4/2016
2009G001	19/4/2016
2009G001	30/4/2016
2009G001	9/5/2016
2009G001	21/5/2016
2009G006	31/1/2016
2009G006	10/2/2016
2009G006	5/3/2016
2009G006	15/3/2016
2009G006	26/3/2016
2009G006	5/4/2016
2009G006	17/4/2016
2009G010	23/4/2016
2009G010	17/5/2016
2009G037	27/5/2016
2009G037	18/6/2016
2009G037	8/7/2016
2009G037	17/7/2016
2009G037	26/7/2016
2009G037	5/8/2016
2009G056	24/5/2016
2010G005	15/4/2016
2010G005	10/5/2016
2010G005	20/5/2016
2010G005	9/6/2016
2010G005	19/6/2016
2010G014	7/3/2016
2010G014	18/3/2016
2010G014	7/4/2016
2010G014	17/4/2016
2010G014	27/4/2016
2010G014	7/5/2016
2010G021	20/6/2016
2010G022	23/3/2016
2010G022	23/4/2016
2010G022	12/5/2016
2010G022	23/5/2016
2010G022	2/6/2016
2010G032	29/4/2016
2010G032	10/5/2016
2010G032	21/5/2016
2010G032	10/6/2016
2010G032	20/7/2016

2010G040	21/3/2016
2010G040	11/4/2016
2010G040	21/4/2016
2010G040	11/5/2016
2010G040	22/5/2016
2010G040	1/6/2016
2010G057	25/5/2016
2010G057	4/6/2016
2010G057	14/6/2016
2010G057	24/6/2016
2010G057	15/7/2016
2010G058	15/6/2016
2010G058	25/6/2016
2010G058	8/7/2016
2010G058	18/7/2016
2010G058	9/8/2016
2010G058	19/8/2016
2010G082	10/6/2016
2010G082	19/6/2016
2010G082	29/6/2016
2010G082	8/7/2016
2010G082	13/8/2016
2010G082	23/8/2016
2010G082	1/9/2016
2010G089	23/4/2016
2010G089	6/5/2016
2010G089	16/5/2016
2010G089	27/5/2016
2010G089	7/6/2016
2010G089	25/6/2016
2010G099	8/7/2016
2010G099	18/7/2016
2010G099	7/8/2016
2010G099	18/8/2016
2010G107	27/6/2016
2010G107	9/7/2016
2010G107	20/7/2016
2010G107	29/7/2016
2010G107	8/8/2016
2010G107	17/8/2016
2010G107	27/8/2016
2011G025	31/1/2016
2011G025	10/2/2016
2011G025	8/3/2016
2011G034	17/4/2016
2011G034	28/4/2016

2011G034	10/5/2016
2011G034	21/5/2016
2011G034	31/5/2016
2011G034	22/6/2016
2011G041	3/9/2016
2011G041	14/9/2016
2011G045	18/5/2016
2011G045	10/6/2016
2011G045	21/6/2016
2011G045	22/7/2016
2011G045	1/8/2016
2011G045	12/8/2016
2011G049	25/6/2016
2011G049	5/7/2016
2011G049	14/7/2016
2011G049	12/8/2016
2011G052	24/4/2016
2011G052	4/5/2016
2011G052	27/5/2016
2011G052	6/7/2016
2011G052	17/6/2016
2011G052	27/6/2016
2011G052	8/7/2016
2011G058	25/5/2016
2011G058	5/6/2016
2011G058	14/6/2016
2011G058	23/6/2016
2011G058	11/7/2016
2011G058	9/8/2016
2011G059	27/4/2016
2011G059	25/5/2016
2011G059	3/6/2016
2011G059	13/6/2016
2011G059	23/6/2016
2011G059	3/7/2016
2011G066	22/5/2016
2011G084	20/6/2016
2011G084	8/7/2016
2011G084	18/7/2016
2011G084	27/7/2016
2011G084	5/8/2016
2011G084	14/8/2016
2011G086	16/6/2016
2011G086	27/6/2016
2011G086	7/7/2016
2011G086	27/7/2016

2011G086	6/8/2016
2012G005	12/3/2016
2012G005	24/3/2016
2012G005	4/4/2016
2012G005	15/4/2016
2012G005	6/5/2016
2012G033	2/4/2016
2012G033	11/6/2016
2012G038	30/4/2016
2012G062	4/7/2016
2012G062	15/7/2016
2012G062	12/8/2016
2012G063	26/5/2016
2012G063	19/6/2016
2012G063	21/7/2016
2012G063	9/8/2016
2012G063	19/8/2016
2012G079	6/6/2016
2012G079	18/6/2016
2012G079	10/7/2016
2012G079	8/8/2016
2012G079	18/8/2016
2012G084	6/3/2016
2012G084	18/3/2016
2012G084	29/3/2016
2012G085	19/8/2016
2012G085	31/8/2016
2012G093	18/4/2016
2012G093	29/4/2016
2012G093	12/5/2016
2012G093	22/5/2016
2012G093	14/6/2016
2012G093	25/6/2016
2012G093	6/7/2016
2013G003	13/3/2016
2013G003	26/3/2016
2013G008	21/3/2016
2013G008	13/4/2016
2013G008	24/4/2016
2013G008	7/5/2016
2013G008	20/5/2016
2013G008	31/5/2016
2013G012	7/3/2016
2013G012	19/3/2016
2013G012	29/3/2016
2013G012	18/4/2016

2013G012	1/5/2016
2013G012	10/5/2016
2013G012	21/5/2016
2013G024	3/5/2016
2013G024	15/5/2016
2013G024	25/5/2016
2013G024	7/6/2016
2013G024	17/6/2016
2013G024	27/6/2016
2013G024	7/7/2016
2013G026	10/4/2016
2013G026	20/4/2016
2013G026	2/5/2016
2013G026	25/5/2016
2013G026	4/6/2016
2013G027	20/3/2016
2013G027	30/3/2016
2013G027	10/4/2016
2013G027	20/4/2016
2013G027	10/5/2016
2013G027	20/5/2016
2013G027	30/5/2016
2013G029	17/4/2016
2013G029	28/4/2016
2013G029	10/5/2016
2013G029	21/5/2016
2013G029	1/6/2016
2013G029	23/6/2016
2013G029	4/7/2016
2013G029	16/7/2016
2013G030	20/4/2016
2013G030	1/5/2016
2013G030	2/5/2016
2013G030	14/5/2016
2013G030	24/5/2016
2013G030	4/6/2016
2013G030	17/6/2016
2013G030	28/6/2016
2013G034	9/4/2016
2013G034	20/4/2016
2013G034	3/5/2016
2013G034	15/5/2016
2013G034	27/5/2016
2013G034	8/6/2016
2013G034	18/6/2016
2013G035	30/4/2016

2013G035	20/5/2016
2013G035	30/5/2016
2013G035	9/6/2016
2013G035	28/6/2016
2013G035	8/7/2016
2013G036	17/4/2016
2013G036	26/4/2016
2013G036	6/5/2016
2013G036	15/5/2016
2013G036	25/5/2016
2013G036	12/6/2016
2013G036	23/6/2016
2013G038	18/4/2016
2013G038	28/4/2016
2013G038	9/5/2016
2013G038	19/5/2016
2013G038	8/6/2016
2013G038	30/6/2016
2013G042	26/4/2016
2013G042	7/5/2016
2013G042	17/5/2016
2013G042	15/6/2016
2013G042	25/6/2016
2013G042	5/7/2016
2013G042	16/7/2016
2013G056	8/3/2016
2013G056	19/3/2016
2013G056	28/3/2016
2013G056	6/4/2016
2013G056	15/4/2016
2013G056	25/4/2016
2013G056	4/5/2016
2013G056	14/5/2016
2013G056	24/5/2016
2013G063	15/4/2016
2013G063	26/4/2016
2013G063	8/5/2016
2013G063	19/5/2016
2013G063	3/7/2016
2013G069	12/4/2016
2013G069	22/4/2016
2013G069	21/5/2016
2013G074	4/4/2016
2013G074	14/4/2016
2013G074	24/4/2016
2013G074	5/5/2016

2013G074	15/5/2016
2013G074	25/5/2016
2013G074	4/6/2016
2013G087	29/4/2016
2013G087	10/5/2016
2013G087	23/5/2016
2013G087	2/6/2016
2013G087	12/6/2016
2013G087	21/6/2016
2013G087	30/6/2016
2013G104	11/7/2016
2013G104	21/7/2016
2013G110	11/3/2016
2013G110	22/3/2016
2013G110	1/4/2016
2013G110	10/4/2016
2013G110	20/4/2016
2013G110	30/4/2016
2013G110	10/5/2016
2013G110	20/5/2016
2013G121	9/5/2016
2013G121	22/5/2016
2013G126	22/4/2016
2013G126	14/5/2016
2013G126	4/6/2016
2013G126	16/6/2016
2013G132	20/5/2016
2013G148	25/6/2016
2013G148	5/7/2016
2013G148	17/7/2016
2013G149	15/5/2016
2013G149	18/5/2016
2013G149	8/6/2016
2013G149	18/6/2016
2013G149	28/6/2016
2013G149	22/7/2016
2014G049	15/6/2016
2014G045	22/6/2016
2014G045	4/7/2016
2014G045	15/7/2016
2014G045	25/8/2016
2014G045	4/9/2016
2014G048	9/5/2016
2016G001	1/2/2016
2016G001	12/2/2016
2016G001	4/3/2016

2016G001	15/3/2016
2016G002	15/2/2016
2016G002	7/3/2016
2016G002	16/3/2016
2016G002	25/3/2016
2016G002	11/4/2016
2016G003	16/2/2016
2016G003	28/2/2016
2016G003	11/3/2016
2016G003	3/4/2016
2016G003	13/4/2016
2016G003	24/4/2016
2016G004	23/2/2016
2016G004	4/3/2016
2016G004	13/3/2016
2016G004	23/3/2016
2016G004	1/4/2016
2016G005	3/3/2016
2016G005	16/3/2016
2016G005	30/3/2016
2016G005	22/4/2016
2016G006	5/3/2016
2016G006	9/4/2016
2016G006	20/4/2016
2016G007	9/3/2016
2016G007	22/3/2016
2016G008	9/3/2016
2016G008	20/3/2016
2016G008	31/3/2016
2016G008	20/4/2016
2016G008	30/4/2016
2016G008	24/5/2016
2016G009	11/3/2016
2016G009	23/3/2016
2016G009	3/4/2016
2016G010	11/3/2016
2016G010	27/4/2016
2016G010	7/5/2016
2016G010	6/6/2016
2016G011	11/3/2016
2016G011	9/6/2016
2016G011	19/6/2016
2016G011	30/6/2016
2016G011	10/7/2016
2016G011	20/7/2016
2016G012	11/3/2016

2016G013	11/3/2016
2016G013	24/3/2016
2016G013	3/4/2016
2016G013	13/4/2016
2016G013	22/4/2016
2016G013	2/5/2016
2016G013	12/5/2016
2016G014	12/3/2016
2016G014	24/3/2016
2016G014	5/5/2016
2016G014	15/5/2016
2016G015	16/3/2016
2016G015	26/3/2016
2016G015	6/4/2016
2016G015	17/4/2016
2016G015	28/4/2016
2016G015	7/5/2016
2016G015	16/5/2016
2016G016	18/3/2016
2016G016	2/4/2016
2016G016	27/4/2016
2016G016	7/5/2016
2016G017	21/3/2016
2016G017	31/3/2016
2016G017	10/4/2016
2016G017	3/5/2016
2016G017	16/5/2016
2016G017	27/5/2016
2016G018	22/3/2016
2016G018	3/4/2016
2016G019	22/3/2016
2016G019	1/4/2016
2016G019	12/4/2016
2016G020	23/3/2016
2016G020	3/5/2016
2016G020	12/5/2016
2016G021	24/3/2016
2016G021	4/4/2016
2016G021	24/4/2016
2016G021	27/5/2016
2016G022	24/3/2016
2016G023	26/3/2016
2016G023	7/4/2016
2016G023	30/4/2016
2016G023	14/5/2016
2016G023	20/5/2016

2016G024	26/3/2016
2016G024	5/4/2016
2016G024	15/4/2016
2016G024	25/4/2016
2016G025	26/3/2016
2016G025	7/4/2016
2016G025	15/4/2016
2016G025	21/4/2016
2016G025	2/5/2016
2016G025	10/6/2016
2016G026	26/3/2016
2016G026	7/4/2016
2016G026	8/4/2016
2016G026	18/4/2016
2016G026	29/4/2016
2016G026	11/5/2016
2016G026	21/5/2016
2016G026	3/6/2016
2016G027	27/3/2016
2016G027	9/4/2016
2016G027	30/4/2016
2016G027	30/4/2016
2016G027	10/5/2016
2016G027	12/6/2016
2016G028	28/3/2016
2016G028	7/4/2016
2016G028	18/4/2016
2016G028	29/4/2016
2016G028	20/5/2016
2016G028	2/6/2016
2016G029	8/4/2016
2016G030	28/3/2016
2016G030	8/4/2016
2016G031	29/3/2016
2016G031	9/4/2016
2016G031	29/4/2016
2016G031	9/5/2016
2016G031	20/5/2016
2016G032	29/3/2016
2016G032	8/4/2016
2016G032	18/4/2016
2016G032	9/5/2016
2016G032	8/6/2016
2016G032	17/6/2016
2016G032	27/6/2016
2016G033	31/3/2016

2016G033	20/4/2016
2016G033	10/5/2016
2016G033	31/5/2016
2016G034	31/3/2016
2016G034	11/4/2016
2016G034	20/4/2016
2016G034	1/5/2016
2016G034	10/5/2016
2016G034	20/5/2016
2016G034	4/7/2016
2016G035	31/3/2016
2016G035	13/4/2016
2016G035	25/4/2016
2016G035	9/5/2016
2016G035	21/5/2016
2016G035	4/6/2016
2016G036	1/4/2016
2016G036	12/4/2016
2016G036	27/4/2016
2016G036	7/5/2016
2016G036	19/5/2016
2016G036	29/5/2016
2016G036	9/6/2016
2016G037	2/4/2016
2016G037	12/4/2016
2016G037	23/4/2016
2016G037	2/5/2016
2016G037	11/5/2016
2016G037	20/5/2016
2016G038	2/4/2016
2016G038	13/4/2016
2016G038	23/4/2016
2016G038	4/5/2016
2016G038	31/5/2016
2016G039	6/4/2016
2016G039	14/4/2016
2016G039	23/4/2016
2016G039	1/5/2016
2016G039	10/5/2016
2016G039	20/5/2016
2016G040	6/4/2016
2016G040	24/4/2016
2016G040	13/5/2016
2016G040	22/5/2016
2016G041	7/4/2016
2016G041	18/4/2016

2016G041	28/4/2016
2016G041	20/5/2016
2016G041	29/5/2016
2016G041	9/6/2016
2016G041	18/6/2016
2016G042	7/4/2016
2016G042	18/4/2016
2016G042	19/6/2016
2016G043	7/4/2016
2016G043	18/4/2016
2016G043	19/5/2016
2016G043	30/5/2016
2016G044	8/4/2016
2016G044	10/5/2016
2016G044	29/5/2016
2016G045	8/4/2016
2016G045	29/4/2016
2016G045	9/5/2016
2016G045	18/5/2016
2016G045	28/5/2016
2016G045	8/6/2016
2016G046	8/4/2016
2016G046	20/4/2016
2016G046	1/5/2016
2016G046	11/5/2016
2016G046	21/5/2016
2016G046	31/5/2016
2016G046	11/6/2016
2016G047	8/4/2016
2016G047	18/4/2016
2016G047	29/4/2016
2016G047	9/5/2016
2016G048	8/4/2016
2016G048	18/5/2016
2016G048	28/5/2016
2016G048	10/6/2016
2016G049	9/4/2016
2016G049	18/4/2016
2016G049	27/4/2016
2016G050	9/4/2016
2016G050	19/4/2016
2016G050	29/4/2016
2016G050	9/5/2016
2016G050	20/5/2016
2016G051	9/4/2016
2016G051	18/4/2016

2016G051	29/4/2016
2016G051	9/5/2016
2016G051	17/5/2016
2016G051	27/5/2016
2016G052	10/4/2016
2016G052	20/4/2016
2016G052	30/4/2016
2016G052	10/5/2016
2016G052	20/5/2016
2016G052	10/6/2016
2016G052	21/6/2016
2016G053	10/4/2016
2016G053	21/4/2016
2016G053	2/5/2016
2016G053	4/5/2016
2016G053	24/5/2016
2016G053	14/6/2016
2016G053	27/6/2016
2016G054	11/4/2016
2016G054	23/4/2016
2016G054	15/5/2016
2016G054	27/5/2016
2016G055	11/4/2016
2016G055	22/4/2016
2016G055	4/5/2016
2016G055	14/5/2016
2016G055	24/5/2016
2016G056	11/4/2016
2016G057	11/4/2016
2016G058	12/4/2016
2016G059	13/4/2016
2016G059	3/5/2016
2016G059	14/5/2016
2016G060	13/4/2016
2016G060	23/4/2016
2016G060	19/5/2016
2016G061	14/4/2016
2016G061	25/4/2016
2016G061	6/5/2016
2016G062	14/4/2016
2016G063	15/4/2016
2016G063	24/4/2016
2016G063	7/5/2016
2016G064	15/4/2016
2016G064	27/4/2016
2016G064	9/5/2016

2016G064	14/7/2016
2016G065	15/4/2016
2016G066	15/4/2016
2016G067	16/4/2016
2016G067	26/4/2016
2016G067	7/5/2016
2016G067	17/5/2016
2016G067	27/5/2016
2016G067	6/6/2016
2016G067	16/6/2016
2016G068	18/4/2016
2016G069	18/4/2016
2016G069	30/4/2016
2016G069	20/5/2016
2016G069	31/5/2016
2016G069	9/6/2016
2016G070	18/4/2016
2016G070	27/4/2016
2016G070	10/5/2016
2016G070	19/5/2016
2016G070	28/5/2016
2016G070	17/6/2016
2016G070	26/6/2016
2016G070	7/7/2016
2016G071	18/4/2016
2016G071	27/4/2016
2016G071	8/5/2016
2016G072	18/4/2016
2016G072	29/4/2016
2016G073	18/4/2016
2016G073	29/4/2016
2016G073	9/5/2016
2016G073	20/5/2016
2016G073	9/6/2016
2016G074	20/4/2016
2016G074	1/5/2016
2016G074	12/5/2016
2016G074	22/5/2016
2016G074	16/6/2016
2016G074	7/7/2016
2016G075	21/4/2016
2016G075	2/5/2016
2016G076	21/4/2016
2016G076	2/5/2016
2016G076	14/5/2016
2016G076	24/5/2016

2016G076	14/6/2016
2016G076	24/6/2016
2016G077	22/4/2016
2016G077	5/5/2016
2016G078	22/4/2016
2016G078	28/4/2016
2016G079	22/4/2016
2016G080	22/4/2016
2016G080	23/5/2016
2016G080	2/6/2016
2016G081	23/4/2016
2016G082	23/4/2016
2016G082	17/5/2016
2016G082	10/6/2016
2016G082	22/6/2016
2016G082	17/7/2016
2016G082	4/8/2016
2016G083	23/4/2016
2016G084	24/4/2016
2016G084	4/5/2016
2016G084	24/5/2016
2016G084	3/6/2016
2016G084	13/6/2016
2016G084	21/6/2016
2016G084	1/7/2016
2016G085	24/4/2016
2016G085	16/5/2016
2016G085	8/6/2016
2016G085	18/6/2016
2016G085	4/7/2016
2016G086	24/4/2016
2016G086	6/5/2016
2016G086	16/5/2016
2016G086	25/5/2016
2016G087	24/4/2016
2016G087	5/5/2016
2016G087	15/5/2016
2016G087	25/5/2016
2016G087	13/6/2016
2016G088	25/4/2016
2016G089	25/4/2016
2016G089	7/5/2016
2016G089	18/5/2016
2016G089	18/6/2016
2016G089	7/7/2016
2016G089	17/7/2016

2016G090	26/4/2016
2016G091	26/4/2016
2016G091	6/5/2016
2016G091	21/5/2016
2016G091	31/5/2016
2016G091	12/6/2016
2016G091	23/6/2016
2016G091	5/7/2016
2016G092	26/4/2016
2016G092	9/5/2016
2016G092	20/5/2016
2016G093	27/4/2016
2016G093	7/5/2016
2016G093	8/5/2016
2016G093	17/5/2016
2016G093	28/5/2016
2016G093	7/6/2016
2016G094	27/4/2016
2016G094	23/5/2016
2016G094	4/6/2016
2016G094	16/6/2016
2016G095	27/4/2016
2016G095	9/5/2016
2016G095	8/6/2016
2016G095	18/6/2016
2016G096	27/4/2016
2016G096	7/5/2016
2016G096	17/5/2016
2016G097	27/4/2016
2016G098	28/4/2016
2016G098	11/5/2016
2016G098	21/5/2016
2016G098	1/6/2016
2016G098	12/6/2016
2016G098	23/6/2016
2016G099	29/4/2016
2016G099	20/5/2016
2016G100	29/4/2016
2016G101	29/4/2016
2016G102	30/4/2016
2016G102	10/5/2016
2016G102	6/6/2016
2016G102	16/6/2016
2016G102	25/6/2016
2016G102	5/7/2016
2016G103	30/4/2016

2016G103	11/5/2016
2016G103	21/5/2016
2016G103	30/6/2016
2016G104	1/5/2016
2016G105	3/5/2016
2016G105	15/5/2016
2016G105	25/5/2016
2016G105	17/6/2016
2016G105	9/7/2016
2016G106	5/5/2016
2016G106	15/5/2016
2016G106	25/5/2016
2016G107	5/5/2016
2016G107	15/5/2016
2016G108	5/5/2016
2016G108	14/5/2016
2016G108	23/5/2016
2016G108	2/6/2016
2016G109	6/5/2016
2016G109	17/5/2016
2016G110	6/5/2016
2016G110	27/5/2016
2016G110	7/6/2016
2016G110	18/6/2016
2016G111	6/5/2016
2016G112	6/5/2016
2016G112	15/5/2016
2016G112	24/5/2016
2016G112	3/6/2016
2016G112	8/7/2016
2016G113	6/5/2016
2016G113	18/5/2016
2016G113	29/5/2016
2016G113	7/6/2016
2016G113	22/6/2016
2016G113	4/7/2016
2016G114	7/5/2016
2016G114	21/5/2016
2016G114	11/8/2016
2016G115	7/5/2016
2016G115	21/5/2016
2016G115	1/6/2016
2016G115	12/6/2016
2016G115	22/6/2016
2016G115	12/7/2016
2016G115	22/7/2016

2016G115	1/8/2016
2016G116	7/5/2016
2016G117	8/5/2016
2016G118	8/5/2016
2016G118	29/5/2016
2016G118	8/6/2016
2016G118	17/6/2016
2016G118	27/6/2016
2016G118	8/7/2016
2016G119	9/5/2016
2016G119	21/5/2016
2016G119	2/6/2016
2016G119	12/6/2016
2016G119	22/6/2016
2016G119	14/7/2016
2016G120	9/5/2016
2016G120	22/5/2016
2016G120	3/6/2016
2016G120	15/6/2016
2016G121	6/5/2016
2016G121	21/5/2016
2016G121	22/5/2016
2016G121	1/6/2016
2016G122	9/5/2016
2016G123	10/5/2016
2016G123	20/5/2016
2016G123	31/5/2016
2016G124	11/5/2016
2016G124	21/5/2016
2016G124	2/6/2016
2016G124	13/6/2016
2016G124	23/6/2016
2016G125	11/5/2016
2016G125	21/5/2016
2016G125	2/6/2016
2016G125	23/6/2016
2016G126	12/5/2016
2016G126	21/5/2016
2016G126	2/6/2016
2016G126	3/7/2016
2016G126	11/8/2016
2016G127	12/5/2016
2016G128	12/5/2016
2016G128	22/5/2016
2016G128	25/6/2016
2016G128	6/7/2016

2016G128	17/7/2016
2016G129	12/5/2016
2016G129	24/5/2016
2016G129	4/6/2016
2016G129	14/6/2016
2016G129	5/7/2016
2016G130	12/5/2016
2016G130	24/5/2016
2016G130	6/7/2016
2016G130	17/7/2016
2016G131	12/5/2016
2016G131	24/5/2016
2016G131	4/6/2016
2016G132	14/5/2016
2016G132	27/5/2016
2016G132	19/6/2016
2016G132	29/6/2016
2016G132	9/7/2016
2016G132	21/7/2016
2016G133	15/5/2016
2016G133	25/5/2016
2016G133	16/6/2016
2016G133	27/6/2016
2016G133	8/7/2016
2016G134	15/5/2016
2016G135	15/5/2016
2016G135	28/5/2016
2016G136	15/5/2016
2016G136	27/5/2016
2016G136	15/6/2016
2016G136	24/6/2016
2016G136	4/7/2016
2016G136	15/7/2016
2016G136	25/7/2016
2016G136	5/8/2016
2016G137	16/5/2016
2016G138	16/5/2016
2016G138	8/6/2016
2016G138	19/6/2016
2016G139	16/5/2016
2016G139	3/6/2016
2016G140	16/5/2016
2016G140	19/6/2016
2016G140	7/7/2016
2016G141	16/5/2016
2016G142	16/5/2016

2016G142	27/5/2016
2016G142	6/6/2016
2016G143	16/5/2016
2016G144	17/5/2016
2016G144	27/5/2016
2016G144	28/5/2016
2016G144	10/6/2016
2016G145	17/5/2016
2016G146	17/5/2016
2016G147	17/5/2016
2016G147	28/5/2016
2016G147	8/6/2016
2016G148	17/5/2016
2016G149	17/5/2016
2016G149	5/6/2016
2016G150	17/5/2016
2016G150	1/6/2016
2016G151	18/5/2016
2016G151	27/5/2016
2016G151	16/6/2016
2016G152	18/5/2016
2016G152	29/5/2016
2016G152	8/6/2016
2016G152	19/6/2016
2016G153	19/5/2016
2016G153	8/6/2016
2016G153	20/6/2016
2016G153	30/6/2016
2016G153	10/7/2016
2016G154	20/5/2016
2016G154	1/6/2016
2016G154	12/6/2016
2016G154	23/6/2016
2016G154	3/7/2016
2016G154	14/7/2016
2016G155	20/5/2016
2016G155	12/6/2016
2016G155	22/6/2016
2016G156	21/5/2016
2016G156	4/6/2016
2016G156	17/6/2016
2016G157	22/5/2016
2016G157	2/6/2016
2016G157	12/6/2016
2016G157	22/6/2016
2016G157	2/7/2016

2016G157	11/7/2016
2016G158	22/5/2016
2016G158	4/6/2016
2016G158	14/6/2016
2016G158	25/6/2016
2016G158	6/7/2016
2016G158	8/7/2016
2016G158	21/7/2016
2016G158	31/7/2016
2016G158	19/8/2016
2016G158	30/8/2016
2016G159	22/5/2016
2016G159	1/6/2016
2016G159	12/6/2016
2016G160	22/5/2016
2016G161	24/5/2016
2016G161	14/7/2016
2016G161	23/7/2016
2016G162	24/5/2016
2016G162	17/6/2016
2016G162	27/6/2016
2016G162	7/7/2016
2016G162	17/7/2016
2016G162	28/7/2016
2016G163	24/5/2016
2016G163	16/6/2016
2016G164	24/5/2016
2016G164	2/6/2016
2016G165	24/5/2016
2016G165	17/6/2016
2016G166	24/5/2016
2016G166	5/6/2016
2016G166	16/6/2016
2016G166	17/6/2016
2016G166	27/6/2016
2016G166	7/7/2016
2016G166	18/7/2016
2016G167	25/5/2016
2016G167	5/6/2016
2016G167	15/6/2016
2016G167	25/6/2016
2016G167	4/7/2016
2016G167	15/7/2016
2016G168	25/5/2016
2016G168	5/6/2016
2016G168	16/6/2016

2016G168	7/7/2016
2016G168	27/7/2016
2016G169	15/6/2016
2016G169	25/6/2016
2016G169	4/7/2016
2016G169	17/7/2016
2016G170	26/5/2016
2016G170	6/6/2016
2016G170	15/6/2016
2016G170	25/6/2016
2016G170	6/7/2016
2016G170	16/7/2016
2016G171	26/5/2016
2016G171	6/6/2016
2016G171	16/6/2016
2016G171	26/6/2016
2016G172	26/5/2016
2016G173	26/5/2016
2016G174	27/5/2016
2016G174	7/6/2016
2016G174	17/6/2016
2016G174	28/6/2016
2016G174	8/7/2016
2016G175	27/5/2016
2016G176	27/5/2016
2016G176	8/6/2016
2016G176	19/6/2016
2016G176	31/7/2016
2016G176	11/8/2016
2016G177	27/5/2016
2016G177	17/6/2016
2016G178	27/5/2016
2016G179	28/5/2016
2016G179	8/6/2016
2016G179	19/6/2016
2016G179	20/6/2016
2016G180	28/5/2016
2016G180	25/6/2016
2016G181	28/5/2016
2016G181	11/6/2016
2016G181	29/7/2016
2016G182	28/5/2016
2016G183	3/6/2016
2016G184	3/6/2016
2016G185	4/6/2016
2016G185	16/6/2016

2016G185	9/7/2016
2016G185	19/7/2016
2016G186	5/6/2016
2016G186	27/6/2016
2016G186	9/8/2016
2016G186	19/8/2016
2016G186	31/8/2016
2016G187	5/6/2016
2016G187	27/6/2016
2016G188	6/6/2016
2016G189	7/6/2016
2016G189	17/6/2016
2016G190	8/6/2016
2016G190	19/6/2016
2016G191	8/6/2016
2016G191	19/6/2016
2016G192	8/6/2016
2016G192	18/6/2016
2016G193	8/6/2016
2016G193	19/6/2016
2016G193	9/7/2016
2016G194	10/6/2016
2016G194	21/6/2016
2016G195	10/6/2016
2016G195	10/7/2016
2016G195	22/7/2016
2016G195	3/8/2016
2016G195	14/8/2016
2016G196	11/6/2016
2016G196	20/6/2016
2016G196	30/6/2016
2016G196	10/7/2016
2016G196	9/8/2016
2016G197	12/6/2016
2016G197	24/6/2016
2016G197	4/7/2016
2016G197	15/7/2016
2016G197	25/7/2016
2016G197	4/8/2016
2016G197	14/8/2016
2016G197	25/8/2016
2016G198	13/6/2016
2016G198	23/6/2016
2016G198	3/7/2016
2016G198	13/7/2016
2016G198	24/7/2016

2016G199	13/6/2016
2016G199	22/6/2016
2016G199	1/7/2016
2016G199	21/7/2016
2016G200	13/6/2016
2016G200	15/7/2016
2016G200	25/7/2016
2016G200	3/8/2016
2016G200	13/8/2016
2016G200	24/8/2016
2016G201	14/6/2016
2016G201	24/6/2016
2016G201	16/7/2016
2016G201	25/7/2016
2016G202	14/6/2016
2016G202	25/6/2016
2016G202	7/7/2016
2016G202	17/7/2016
2016G202	21/7/2016
2016G202	27/7/2016
2016G203	15/6/2016
2016G203	5/7/2016
2016G203	16/7/2016
2016G204	15/6/2016
2016G204	26/6/2016
2016G204	7/7/2016
2016G204	17/7/2016
2016G204	28/7/2016
2016G205	15/6/2016
2016G205	6/7/2016
2016G205	29/7/2016
2016G205	7/8/2016
2016G205	17/8/2016
2016G205	27/8/2016
2016G206	18/6/2016
2016G206	27/6/2016
2016G206	7/7/2016
2016G206	19/7/2016
2016G207	18/6/2016
2016G207	2/7/2016
2016G208	18/6/2016
2016G208	29/6/2016
2016G208	30/7/2016
2016G208	20/8/2016
2016G208	30/8/2016
2016G209	19/6/2016

2016G209	1/7/2016
2016G209	9/7/2016
2016G209	19/7/2016
2016G209	29/7/2016
2016G209	7/8/2016
2016G209	18/8/2016
2016G210	20/6/2016
2016G211	20/6/2016
2016G212	20/6/2016
2016G213	20/6/2016
2016G213	1/7/2016
2016G213	4/7/2016
2016G213	14/7/2016
2016G213	5/8/2016
2016G213	25/8/2016
2016G214	20/6/2016
2016G215	22/6/2016
2016G215	2/7/2016
2016G215	13/7/2016
2016G215	24/7/2016
2016G215	3/8/2016
2016G216	22/6/2016
2016G216	4/7/2016
2016G216	14/7/2016
2016G216	24/7/2016
2016G216	3/8/2016
2016G216	15/8/2016
2016G217	22/6/2016
2016G217	10/7/2016
2016G217	20/7/2016
2016G217	29/7/2016
2016G218	22/6/2016
2016G218	2/7/2016
2016G220	23/6/2016
2016G220	3/7/2016
2016G220	14/7/2016
2016G220	24/7/2016
2016G221	23/6/2016
2016G221	14/7/2016
2016G221	3/8/2016
2016G221	13/8/2016
2016G222	23/6/2016
2016G222	7/7/2016
2016G222	29/7/2016
2016G222	8/8/2016
2016G222	19/8/2016

2016G223	25/6/2016
2016G223	5/7/2016
2016G223	17/7/2016
2016G223	29/7/2016
2016G223	10/8/2016
2016G223	22/8/2016
2016G224	26/6/2016
2016G224	19/7/2016
2016G224	10/8/2016
2016G224	21/8/2016
2016G224	1/9/2016
2016G225	26/6/2016
2016G225	18/7/2016
2016G225	13/8/2016
2016G226	28/6/2016
2016G227	28/6/2016
2016G227	29/7/2016
2016G228	30/6/2016
2016G228	13/8/2016
2016G229	30/6/2016
2016G229	20/7/2016
2016G229	22/7/2016
2016G229	1/8/2016
2016G229	11/8/2016
2016G229	22/8/2016
2016G230	30/6/2016
2016G230	20/7/2016
2016G230	8/8/2016
2016G230	9/8/2016
2016G230	30/8/2016
2016G231	3/7/2016
2016G231	17/7/2016
2016G232	5/7/2016
2016G233	5/7/2016
2016G233	15/7/2016
2016G234	6/7/2016
2016G235	6/7/2016
2016G236	7/7/2016
2016G236	18/8/2016
2016G236	29/8/2016
2016G236	22/9/2016
2016G237	7/7/2016
2016G237	17/7/2016
2016G238	7/7/2016
2016G238	30/7/2016
2016G239	7/7/2016

2016G239	17/7/2016
2016G239	6/8/2016
2016G239	16/8/2016
2016G240	7/7/2016
2016G241	9/7/2016
2016G241	19/7/2016
2016G241	6/8/2016
2016G241	26/8/2016
2016G241	5/9/2016
2016G242	11/7/2016
2016G242	22/7/2016
2016G243	13/7/2016
2016G243	3/8/2016
2016G244	14/7/2016
2016G244	24/7/2016
2016G244	3/8/2016
2016G244	13/8/2016
2016G244	23/8/2016
2016G245	15/7/2016
2016G245	14/8/2016
2016G245	24/8/2016
2016G246	15/7/2016
2016G246	25/7/2016
2016G246	4/8/2016
2016G246	15/8/2016
2016G246	26/8/2016
2016G246	17/9/2016
2016G247	15/7/2016
2016G247	24/7/2016
2016G247	4/8/2016
2016G247	14/8/2016
2016G247	23/8/2016
2016G247	1/9/2016
2016G247	10/9/2016
2016G248	15/7/2016
2016G248	26/7/2016
2016G248	4/8/2016
2016G248	13/8/2016
2016G248	23/8/2016
2016G249	15/7/2016
2016G250	16/7/2016
2016G251	17/7/2016
2016G251	28/7/2016
2016G251	9/8/2016
2016G252	17/7/2016
2016G252	7/8/2016

2016G252	18/8/2016
2016G252	21/9/2016
2016G253	17/7/2016
2016G253	28/7/2016
2016G253	18/8/2016
2016G253	27/8/2016
2016G253	5/9/2016
2016G254	18/7/2016
2016G255	19/7/2016
2016G255	29/7/2016
2016G255	9/8/2016
2016G255	19/8/2016
2016G255	29/8/2016
2016G255	22/9/2016
2016G256	3/5/2016
2016G257	20/7/2016
2016G258	20/7/2016
2016G258	7/8/2016
2016G258	17/8/2016
2016G259	20/7/2016
2016G259	18/8/2016
2016G260	21/7/2016
2016G260	19/8/2016
2016G260	30/8/2016
2016G261	21/7/2016
2016G261	4/8/2016
2016G261	15/8/2016
2016G261	4/9/2016
2016G261	14/9/2016
2016G262	21/7/2016
2016G263	24/7/2016
2016G263	3/8/2016
2016G263	13/8/2016
2016G264	24/7/2016
2016G264	6/9/2016
2016G265	24/7/2016
2016G265	2/8/2016
2016G265	24/8/2016
2016G266	24/7/2016
2016G266	4/8/2016
2016G267	25/7/2016
2016G267	6/8/2016
2016G267	15/8/2016
2016G267	26/8/2016
2016G268	26/7/2016
2016G269	28/7/2016

2016G269	7/8/2016
2016G269	18/8/2016
2016G269	28/8/2016
2016G269	17/9/2016
2016G269	27/9/2016
2016G270	29/7/2016
2016G270	9/8/2016
2016G270	18/8/2016
2016G270	27/8/2016
2016G270	5/9/2016
2016G271	29/7/2016
2016G272	30/7/2016
2016G272	9/8/2016
2016G272	16/8/2016
2016G273	30/7/2016
2016G274	31/7/2016
2016G275	1/8/2016
2016G276	1/8/2016
2016G277	2/8/2016
2016G278	2/8/2016
2016G278	12/8/2016
2016G278	22/8/2016
2016G278	1/9/2016
2016G278	22/9/2016
2016G279	2/8/2016
2016G279	12/8/2016
2016G279	22/8/2016
2016G280	2/8/2016
2016G280	13/8/2016
2016G281	3/8/2016
2016G282	3/8/2016
2016G282	14/8/2016
2016G282	26/8/2016
2016G282	18/9/2016
2016G282	29/9/2016
2016G283	4/8/2016
2016G283	16/8/2016
2016G283	27/8/2016
2016G284	4/8/2016
2016G285	4/8/2016
2016G286	4/8/2016
2016G287	5/8/2016
2016G287	15/8/2016
2016G288	5/8/2016
2016G288	15/8/2016
2016G288	4/9/2016

2016G289	6/8/2016
2016G290	9/8/2016
2016G291	9/8/2016
2016G291	20/8/2016
2016G292	10/8/2016
2016G292	22/8/2016
2016G293	10/8/2016
2016G294	10/8/2016
2016G295	11/8/2016
2016G296	11/8/2016
2016G297	11/8/2016
2016G297	22/8/2016
2016G297	12/9/2016
2016G297	22/9/2016
2016G297	2/10/2016
2016G297	13/10/2016
2016G298	12/8/2016
2016G299	12/8/2016
2016G299	22/8/2016
2016G300	12/8/2016
2016G300	23/8/2016
2016G300	2/9/2016
2016G300	23/9/2016
2016G301	13/8/2016
2016G302	14/8/2016
2016G303	18/8/2016
2016G304	18/8/2016
2016G304	27/8/2016
2016G304	6/9/2016
2016G305	19/8/2016
2016G305	22/9/2016
2016G306	20/8/2016
2016G307	22/8/2016
2016G308	22/8/2016
2016G308	3/9/2016
2016G309	24/8/2016
2016G309	6/10/2016
2016G309	16/10/2016
2016G310	26/8/2016
2016G310	16/9/2016
2016G311	1/9/2016
2016G311	23/9/2016
2016G312	3/9/2016
2016G312	16/9/2016
2016G313	5/9/2016
2016G313	16/9/2016

2016G314	8/9/2016
2016G315	20/9/2016
2016G316	23/9/2016
2016G316	2/10/2016
2016G316	12/10/2016
2016G317	1/10/2016
2016G318	8/10/2016
2016G319	9/10/2016
2016G320	23/6/2016
1993G071	2/7/2017
1993G077	10/5/2017
1993G077	21/6/2017
1993G077	28/6/2017
1993G077	8/7/2017
1993G077	12/7/2017
1993G077	22/7/2017
1993G077	28/7/2017
1993G077	1/8/2017
1993G077	11/8/2017
1993G122	21/8/2017
1993G122	2/9/2017
1996G009	2/6/2017
1996G009	12/6/2017
1996G009	23/6/2017
1996G009	2/7/2017
1996G009	11/7/2017
1996G009	21/7/2017
1996G009	30/7/2017
1996G009	9/8/2017
1996G009	19/8/2017
1996G016	15/7/2017
1998G033	8/7/2017
1998G033	19/7/2017
1998G033	29/7/2017
1998G033	9/8/2017
1998G033	27/8/2017
1998G033	7/9/2017
1999G003	2/4/2017
1999G016	12/6/2017
1999G016	23/6/2017
1999G016	5/7/2017
1999G016	27/7/2017
1999G024	7/4/2017
1999G024	8/4/2017
1999G024	18/4/2017
1999G024	28/4/2017

1999G024	8/5/2017
1999G024	17/5/2017
1999G024	27/5/2017
1999G024	6/6/2017
1999G077	20/5/2017
1999G077	18/6/2017
2001G035	26/4/2017
2001G035	7/5/2017
2001G035	16/5/2017
2001G035	5/6/2017
2001G035	18/6/2017
2003G008	28/5/2017
2004G052	27/7/2017
2004G052	7/8/2017
2004G052	27/8/2017
2004G052	5/9/2017
2006G012	24/4/2017
2006G012	14/5/2017
2006G012	2/6/2017
2006G012	13/6/2017
2008G002	29/3/2017
2008G002	12/4/2017
2008G002	23/4/2017
2008G002	6/5/2017
2008G002	7/5/2017
2008G002	28/5/2017
2008G002	9/6/2017
2008G006	16/4/2017
2008G006	26/4/2017
2008G006	27/4/2017
2008G006	6/5/2017
2008G006	17/5/2017
2008G006	26/5/2017
2008G006	14/6/2017
2008G021	3/4/2017
2008G021	13/4/2017
2008G021	23/4/2017
2008G021	14/5/2017
2008G021	24/5/2017
2008G021	17/6/2017
2008G063	17/3/2017
2009G002	22/4/2017
2009G024	26/5/2017
2009G024	7/6/2017
2009G024	28/6/2017
2009G024	17/7/2017

2009G027	18/5/2017
2009G027	29/5/2017
2009G027	9/6/2017
2009G027	19/6/2017
2009G027	29/6/2017
2009G027	10/7/2017
2010G015	16/5/2017
2010G015	27/5/2017
2010G015	16/6/2017
2010G015	27/6/2017
2010G015	18/7/2017
2010G015	28/7/2017
2010G015	9/8/2017
2010G015	22/8/2017
2010G018	25/4/2017
2010G018	6/5/2017
2010G018	18/5/2017
2010G018	29/5/2017
2010G018	8/6/2017
2010G018	18/6/2017
2010G030	13/4/2017
2010G030	24/4/2017
2010G030	15/5/2017
2010G030	17/6/2017
2010G060	26/5/2017
2010G060	4/6/2017
2010G060	12/7/2017
2010G060	21/7/2017
2010G060	8/8/2017
2010G060	18/8/2017
2010G100	27/5/2017
2010G100	15/6/2017
2010G100	24/6/2017
2010G100	4/7/2017
2010G100	14/7/2017
2010G100	24/7/2017
2010G100	3/8/2017
2010G100	13/8/2017
2010G103	18/8/2017
2010G103	30/8/2017
2010G103	10/9/2017
2010G103	21/9/2017
2011G038	18/8/2017
2011G038	30/8/2017
2011G060	26/6/2017
2011G060	20/7/2017

2011G060	30/7/2017
2011G060	10/8/2017
2011G060	21/8/2017
2011G060	1/9/2017
2011G074	12/6/2017
2011G100	9/9/2017
2012G024	9/3/2017
2012G024	20/3/2017
2012G024	29/3/2017
2012G024	8/4/2017
2012G024	17/4/2017
2012G025	25/3/2017
2012G025	4/4/2017
2012G025	15/4/2017
2012G025	25/4/2017
2012G025	4/5/2017
2012G025	23/5/2017
2012G025	13/6/2017
2012G025	17/6/2017
2012G032	9/5/2017
2012G032	21/5/2017
2012G032	12/6/2017
2012G032	23/6/2017
2012G032	14/7/2017
2012G032	6/8/2017
2012G035	11/5/2017
2012G035	20/5/2017
2012G035	7/6/2017
2012G035	26/6/2017
2012G035	6/7/2017
2012G035	15/7/2017
2013G006	12/3/2017
2013G006	24/3/2017
2013G006	4/4/2017
2013G006	14/4/2017
2013G006	24/4/2017
2013G006	5/5/2017
2013G006	15/5/2017
2013G019	29/3/2017
2013G019	9/4/2017
2013G019	19/4/2017
2013G019	29/4/2017
2013G019	9/5/2017
2013G019	18/5/2017
2013G019	28/5/2017
2013G019	8/6/2017

2013G028	14/3/2017
2013G028	25/3/2017
2013G028	5/4/2017
2013G028	16/4/2017
2013G031	15/3/2017
2013G031	25/3/2017
2013G031	5/4/2017
2013G031	15/4/2017
2013G031	24/4/2017
2013G031	5/5/2017
2013G051	11/5/2017
2013G051	21/5/2017
2013G051	1/6/2017
2013G051	11/6/2017
2013G051	21/6/2017
2013G051	2/7/2017
2013G051	14/7/2017
2013G066	17/4/2017
2013G066	28/4/2017
2013G066	9/5/2017
2013G066	18/5/2017
2013G066	9/6/2017
2013G072	23/4/2017
2013G072	5/5/2017
2013G075	12/6/2017
2013G075	2/7/2017
2013G075	11/7/2017
2013G075	21/7/2017
2013G075	9/8/2017
2013G075	20/8/2017
2013G079	21/6/2017
2013G079	1/7/2017
2013G079	11/7/2017
2013G079	8/8/2017
2013G079	27/8/2017
2013G093	10/5/2017
2013G093	19/5/2017
2013G093	30/5/2017
2013G093	11/6/2017
2013G093	21/6/2017
2013G093	3/7/2017
2013G109	16/7/2017
2013G109	25/7/2017
2013G109	4/8/2017
2013G109	14/8/2017
2013G109	24/8/2017

2013G109	17/9/2017
2013G139	12/6/2017
2013G162	27/7/2017
2014G009	12/3/2017
2014G009	22/3/2017
2014G009	1/4/2017
2014G009	2/4/2017
2014G009	12/4/2017
2014G009	22/4/2017
2014G012	15/3/2017
2014G012	6/4/2017
2014G015	10/3/2017
2014G015	22/3/2017
2014G015	5/4/2017
2014G015	15/4/2017
2014G015	25/4/2017
2014G015	5/5/2017
2014G015	15/5/2017
2014G015	8/6/2017
2014G020	12/3/2017
2014G020	31/3/2017
2014G020	9/4/2017
2014G020	19/4/2017
2014G040	16/3/2017
2014G040	16/4/2017
2014G040	25/4/2017
2014G040	6/5/2017
2014G041	22/3/2017
2014G041	13/4/2017
2014G041	1/5/2017
2014G041	10/5/2017
2014G041	18/5/2017
2014G041	7/6/2017
2014G041	18/6/2017
2014G069	2/5/2017
2014G069	4/6/2017
2014G069	15/6/2017
2014G069	19/7/2017
2014G101	21/7/2017
2014G101	2/8/2017
2014G101	13/8/2017
2014G101	23/8/2017
2014G101	4/9/2017
2014G101	12/9/2017
2014G101	13/9/2017
2014G101	24/9/2017

2014G101	6/10/2017
2014G102	23/6/2017
2014G102	30/7/2017
2014G102	11/8/2017
2014G127	21/7/2017
2014G127	30/7/2017
2014G132	30/7/2017
2014G132	20/8/2017
2014G132	30/8/2017
2014G132	9/9/2017
2014G132	19/9/2017
2014G132	20/9/2017
2014G132	30/9/2017
2014G133	21/4/2017
2014G133	1/5/2017
2014G133	11/5/2017
2014G133	21/5/2017
2014G133	11/6/2017
2014G133	20/6/2017
2014G133	2/7/2017
2014G133	11/7/2017
2015G120	22/9/2017
2017G001	4/3/2017
2017G001	16/3/2017
2017G002	5/3/2017
2017G002	18/3/2017
2017G002	29/3/2017
2017G002	10/4/2017
2017G002	23/4/2017
2017G002	4/5/2017
2017G003	6/3/2017
2017G003	27/3/2017
2017G003	6/4/2017
2017G003	16/4/2017
2017G003	26/4/2017
2017G003	6/5/2017
2017G004	6/3/2017
2017G004	18/3/2017
2017G004	30/3/2017
2017G004	11/4/2017
2017G004	22/4/2017
2017G004	4/5/2017
2017G005	8/3/2017
2017G005	21/3/2017
2017G005	31/3/2017
2017G006	9/3/2017

2017G006	24/3/2017
2017G006	14/4/2017
2017G006	23/4/2017
2017G006	3/5/2017
2017G007	10/3/2017
2017G007	21/3/2017
2017G007	1/4/2017
2017G007	11/4/2017
2017G007	21/4/2017
2017G007	1/5/2017
2017G008	14/3/2017
2017G008	26/3/2017
2017G008	7/4/2017
2017G008	17/4/2017
2017G008	27/4/2017
2017G008	8/5/2017
2017G008	19/6/2017
2017G009	15/3/2017
2017G009	27/3/2017
2017G009	9/4/2017
2017G010	10/3/2017
2017G010	22/3/2017
2017G010	2/4/2017
2017G010	14/4/2017
2017G010	24/4/2017
2017G010	4/5/2017
2017G010	24/5/2017
2017G011	25/3/2017
2017G011	5/4/2017
2017G011	16/4/2017
2017G011	26/4/2017
2017G011	9/5/2017
2017G012	28/3/2017
2017G012	9/4/2017
2017G012	20/4/2017
2017G012	30/4/2017
2017G012	13/5/2017
2017G012	28/5/2017
2017G013	28/3/2017
2017G013	21/4/2017
2017G013	1/5/2017
2017G013	10/5/2017
2017G013	19/5/2017
2017G013	29/5/2017
2017G013	8/6/2017
2017G013	20/6/2017

2017G014	1/4/2017
2017G014	14/4/2017
2017G014	24/4/2017
2017G014	17/5/2017
2017G014	29/5/2017
2017G014	3/6/2017
2017G014	5/6/2017
2017G015	2/4/2017
2017G015	14/4/2017
2017G015	7/5/2017
2017G015	29/5/2017
2017G015	8/6/2017
2017G015	21/6/2017
2017G016	2/4/2017
2017G016	13/4/2017
2017G016	4/5/2017
2017G016	22/5/2017
2017G016	1/6/2017
2017G016	12/6/2017
2017G017	2/4/2017
2017G017	13/4/2017
2017G017	23/4/2017
2017G017	4/5/2017
2017G017	12/5/2017
2017G017	21/5/2017
2017G017	30/5/2017
2017G017	12/6/2017
2017G018	3/4/2017
2017G018	14/4/2017
2017G018	24/4/2017
2017G018	6/5/2017
2017G018	15/5/2017
2017G018	25/5/2017
2017G019	3/4/2017
2017G019	14/4/2017
2017G019	25/4/2017
2017G019	6/5/2017
2017G019	15/5/2017
2017g019	26/5/2017
2017G019	5/6/2017
2017G019	16/6/2017
2017G020	5/4/2017
2017G020	24/4/2017
2017G020	14/5/2017
2017G020	23/5/2017
2017G021	9/4/2017

2017G022	10/4/2017
2017G022	20/4/2017
2017G022	30/4/2017
2017G022	12/5/2017
2017G023	10/4/2017
2017G023	21/4/2017
2017G023	14/5/2017
2017G023	23/5/2017
2017G023	4/6/2017
2017G024	11/4/2017
2017G024	21/4/2017
2017G024	11/5/2017
2017G024	20/5/2017
2017G024	30/5/2017
2017G024	10/6/2017
2017G025	11/4/2017
2017G025	21/4/2017
2017G025	2/5/2017
2017G025	12/5/2017
2017G025	23/5/2017
2017G026	11/4/2017
2017G026	2/5/2017
2017G026	12/5/2017
2017G026	22/5/2017
2017G027	12/4/2017
2017G027	22/4/2017
2017G027	2/5/2017
2017G027	24/5/2017
2017G027	13/6/2017
2017G028	13/4/2017
2017G028	22/4/2017
2017G028	1/5/2017
2017G029	14/4/2017
2017G029	25/4/2017
2017G030	14/4/2017
2017G030	15/5/2017
2017G030	24/6/2017
2017G030	4/7/2017
2017G031	14/4/2017
2017G031	24/4/2017
2017G031	4/5/2017
2017G031	14/5/2017
2017G031	26/5/2017
2017G032	15/4/2017
2017G032	25/4/2017
2017G032	4/5/2017

2017G032	14/5/2017
2017G032	23/5/2017
2017G032	2/6/2017
2017G033	16/4/2017
2017G034	17/4/2017
2017G034	27/4/2017
2017G034	15/5/2017
2017G034	23/5/2017
2017G034	31/5/2017
2017G034	9/6/2017
2017G034	18/6/2017
2017G035	18/4/2017
2017G035	29/4/2017
2017G036	18/4/2017
2017G037	19/4/2017
2017G037	4/7/2017
2017G037	20/7/2017
2017G038	19/4/2017
2017G038	1/5/2017
2017G039	19/4/2017
2017G039	28/4/2017
2017G039	18/5/2017
2017G040	6/4/2017
2017G040	21/4/2017
2017G040	2/5/2017
2017G040	13/5/2017
2017G040	24/6/2017
2017G040	4/7/2017
2017G040	15/7/2017
2017G041	22/4/2017
2017G041	15/5/2017
2017G041	5/6/2017
2017G041	17/6/2017
2017G041	29/6/2017
2017G042	24/4/2017
2017G042	6/5/2017
2017G042	17/5/2017
2017G042	8/6/2017
2017G042	20/6/2017
2017G042	1/7/2017
2017G043	24/4/2017
2017G043	4/5/2017
2017G043	16/5/2017
2017G043	26/5/2017
2017G043	15/6/2017
2017G043	25/6/2017

2017G044	25/4/2017
2017G044	6/5/2017
2017G044	26/5/2017
2017G044	14/6/2017
2017G044	23/6/2017
2017G045	25/4/2017
2017G045	6/5/2017
2017G045	29/5/2017
2017G045	8/6/2017
2017G045	22/6/2017
2017G045	4/7/2017
2017G046	25/4/2017
2017G046	15/5/2017
2017G046	26/5/2017
2017G046	6/6/2017
2017G046	26/6/2017
2017G047	27/4/2017
2017G047	17/5/2017
2017G047	27/5/2017
2017G047	7/6/2017
2017G047	17/6/2017
2017G047	28/6/2017
2017G048	12/5/2017
2017G048	22/5/2017
2017G049	28/4/2017
2017G049	8/5/2017
2017G049	21/5/2017
2017G049	31/5/2017
2017G050	19/5/2017
2017G050	29/4/2017
2017G050	10/5/2017
2017G050	22/5/2017
2017G050	1/6/2017
2017G050	11/6/2017
2017G050	22/6/2017
2017G050	2/7/2017
2017G050	12/7/2017
2017G050	26/7/2017
2017G051	29/4/2017
2017G051	9/5/2017
2017G051	9/5/2017
2017G052	1/5/2017
2017G053	3/5/2017
2017G053	14/5/2017
2017G053	25/5/2017
2017G053	4/6/2017

2017G053	15/6/2017
2017G054	3/5/2017
2017G054	15/5/2017
2017G054	6/6/2017
2017G054	16/4/2017
2017G054	26/6/2017
2017G055	3/5/2017
2017G055	23/5/2017
2017G055	6/6/2017
2017G056	4/5/2017
2017G056	11/6/2017
2017G057	6/5/2017
2017G057	12/6/2017
2017G057	23/6/2017
2017G058	8/5/2017
2017G058	21/5/2017
2017G058	1/6/2017
2017G058	24/6/2017
2017G059	9/5/2017
2017G059	19/5/2017
2017G059	30/5/2017
2017G059	11/6/2017
2017G059	22/6/2017
2017G059	3/7/2017
2017G059	15/7/2017
2017G060	9/5/2017
2017G060	19/5/2017
2017G060	30/5/2017
2017G060	10/6/2017
2017G060	21/6/2017
2017G061	10/5/2017
2017G062	10/5/2017
2017G062	20/5/2017
2017G062	30/5/2017
2017G062	8/6/2017
2017G062	17/6/2017
2017G062	6/7/2017
2017G062	15/7/2017
2017G063	30/5/2017
2017G063	10/5/2017
2017G063	9/6/2017
2017G063	18/6/2017
2017G063	28/6/2017
2017G063	8/7/2017
2017G064	10/5/2017
2017G064	20/5/2017

2017G064	30/5/2017
2017G064	9/6/2017
2017G064	19/6/2017
2017G064	29/6/2017
2017G064	29/6/2017
2017G064	10/7/2017
2017G064	21/7/2017
2017G064	31/7/2017
2017G065	10/5/2017
2017G065	29/5/2017
2017G065	8/6/2017
2017G065	19/6/2017
2017G065	24/6/2017
2017G065	1/7/2017
2017G066	12/5/2017
2017G067	12/5/2017
2017G068	15/5/2017
2017G068	26/5/2017
2017G068	5/6/2017
2017G068	15/6/2017
2017G068	24/6/2017
2017G068	15/7/2017
2017G069	16/5/2017
2017G070	18/5/2017
2017G070	29/5/2017
2017G070	8/6/2017
2017G070	27/6/2017
2017G070	9/7/2017
2017G071	19/5/2017
2017G071	28/5/2017
2017G071	5/6/2017
2017G071	15/6/2017
2017G071	26/6/2017
2017G072	19/5/2017
2017G072	29/5/2017
2017G072	8/6/2017
2017G072	18/6/2017
2017G072	29/6/2017
2017G072	11/7/2017
2017G073	19/5/2017
2017G073	30/5/2017
2017G073	9/6/2017
2017G074	19/5/2017
2017G074	9/6/2017
2017G074	19/6/2017
2017G074	29/6/2017

2017G074	9/7/2017
2017G075	20/5/2017
2017G076	20/5/2017
2017G076	30/5/2017
2017G076	18/6/2017
2017G076	29/6/2017
2017G077	23/5/2017
2017G077	14/6/2017
2017G078	23/5/2017
2017G078	2/6/2017
2017G078	16/6/2017
2017G078	26/6/2017
2017G078	7/7/2017
2017G079	15/6/2017
2017G079	6/7/2017
2017G079	16/7/2017
2017G079	27/7/2017
2017G080	24/5/2017
2017G080	8/6/2017
2017G080	21/6/2017
2017G080	2/7/2017
2017G080	14/7/2017
2017G080	24/7/2017
2017G080	8/8/2017
2017G081	2/6/2017
2017G082	2/6/2017
2017G082	12/7/2017
2017G083	5/6/2017
2017G083	17/6/2017
2017G083	29/6/2017
2017G083	7/7/2017
2017G083	10/7/2017
2017G083	22/7/2017
2017G084	6/6/2017
2017G084	16/6/2017
2017G084	6/7/2017
2017G084	15/7/2017
2017G084	25/7/2017
2017G084	3/8/2017
2017G084	14/8/2017
2017G085	7/6/2017
2017G085	19/6/2017
2017G085	30/6/2017
2017G085	14/7/2017
2017G086	9/6/2017
2017G087	9/6/2017

2017G087	18/6/2017
2017G087	29/6/2017
2017G087	16/7/2017
2017G087	26/7/2017
2017G087	4/8/2017
2017G087	13/8/2017
2017G088	30/6/2017
2017G088	10/6/2017
2017G088	19/6/2017
2017G088	30/6/2017
2017G088	10/7/2017
2017G088	19/7/2017
2017G089	10/6/2017
2017G089	20/6/2017
2017G089	25/7/2017
2017G089	14/8/2017
2017G090	13/6/2017
2017G090	2/7/2017
2017G090	12/7/2017
2017G090	21/7/2017
2017G090	11/8/2017
2017G091	13/6/2017
2017G091	23/6/2017
2017G091	4/7/2017
2017G091	25/7/2017
2017G092	14/6/2017
2017G092	25/6/2017
2017G093	14/6/2017
2017G093	28/6/2017
2017G093	9/8/2017
2017G093	20/8/2017
2017G093	13/9/2017
2017G094	14/6/2017
2017G094	23/6/2017
2017G094	14/7/2017
2017G094	2/8/2017
2017G094	12/8/2017
2017G094	22/8/2017
2017G094	3/9/2017
2017G095	15/6/2017
2017G095	15/6/2017
2017G095	15/7/2017
2017G095	25/7/2017
2017G095	14/8/2017
2017G096	16/6/2017
2017G096	26/6/2017

2017G096	17/7/2017
2017G096	28/7/2017
2017G097	18/6/2017
2017G098	18/6/2017
2017G098	28/6/2017
2017G098	8/7/2017
2017G099	18/6/2017
2017G100	19/6/2017
2017G100	28/6/2017
2017G100	8/7/2017
2017G100	17/7/2017
2017G100	26/7/2017
2017G100	5/8/2017
2017G100	14/8/2017
2017G100	23/8/2017
2017G101	22/6/2017
2017G101	3/7/2017
2017G101	14/7/2017
2017G101	3/8/2017
2017G101	14/8/2017
2017G102	23/6/2017
2017G102	14/7/2017
2017G103	25/6/2017
2017G104	26/6/2017
2017G104	15/7/2017
2017G104	25/7/2017
2017G104	6/8/2017
2017G104	17/8/2017
2017G105	7/7/2017
2017G106	27/6/2017
2017G106	8/7/2017
2017G106	31/7/2017
2017G106	12/8/2017
2017G107	29/6/2017
2017G107	10/7/2017
2017G107	20/7/2017
2017G108	30/6/2017
2017G109	1/7/2017
2017G109	11/7/2017
2017G109	21/7/2017
2017G109	30/7/2017
2017G109	23/8/2017
2017G110	1/7/2017
2017G110	11/7/2017
2017G110	20/7/2017
2017G110	30/7/2017

2017G111	2/7/2017
2017G111	12/7/2017
2017G111	22/7/2017
2017G112	6/7/2017
2017G112	11/8/2017
2017G112	11/8/2017
2017G112	24/8/2017
2017G112	5/9/2017
2017G113	6/7/2017
2017G113	16/7/2017
2017G113	4/8/2017
2017G113	23/8/2017
2017G114	6/7/2017
2017G114	15/7/2017
2017G114	25/7/2017
2017G114	4/8/2017
2017G114	14/8/2017
2017G114	26/8/2017
2017G114	6/9/2017
2017G115	10/7/2017
2017G115	18/7/2017
2017G115	28/7/2017
2017G115	29/7/2017
2017G115	9/8/2017
2017G115	18/8/2017
2017G115	28/8/2017
2017G115	7/9/2017
2017G115	17/9/2017
2017G116	10/7/2017
2017G116	3/8/2017
2017G116	14/8/2017
2017G117	11/7/2017
2017G118	11/7/2017
2017G119	12/7/2017
2017G119	22/7/2017
2017G120	14/7/2017
2017G120	24/7/2017
2017G120	2/8/2017
2017G120	12/8/2017
2017G120	21/8/2017
2017G120	30/8/2017
2017G120	9/9/2017
2017G121	15/7/2017
2017G121	26/7/2017
2017G121	6/8/2017
2017G122	15/7/2017

2017G122	25/7/2017
2017G122	5/8/2017
2017G122	14/8/2017
2017G122	24/8/2017
2017G123	15/7/2017
2017G123	27/7/2017
2017G123	20/8/2017
2017G123	31/8/2017
2017G124	15/7/2017
2017G124	11/8/2017
2017G124	5/9/2017
2017G125	16/7/2017
2017G126	16/7/2017
2017G126	26/7/2017
2017G127	17/7/2017
2017G127	21/8/2017
2017G127	2/9/2017
2017G128	7/8/2017
2017G128	17/8/2017
2017G128	17/8/2017
2017G128	27/8/2017
2017G128	6/9/2017
2017G128	16/9/2017
2017G129	20/7/2017
2017G129	9/8/2017
2017G129	19/8/2017
2017G130	22/7/2017
2017G130	1/8/2017
2017G130	13/8/2017
2017G130	23/8/2017
2017G130	4/9/2017
2017G131	25/7/2017
2017G132	26/7/2017
2017G133	26/7/2017
2017G134	27/7/2017
2017G134	7/8/2017
2017G134	17/8/2017
2017G134	27/8/2017
2017G134	8/9/2017
2017G135	28/7/2017
2017G136	29/7/2017
2017G136	10/8/2017
2017G136	22/8/2017
2017G136	3/9/2017
2017G136	13/9/2017
2017G137	30/7/2017

2017G137	10/8/2017
2017G137	20/8/2017
2017G137	30/8/2017
2017G138	30/7/2017
2017G138	30/8/2017
2017G139	30/7/2017
2017G139	30/8/2017
2017G139	10/9/2017
2017G140	31/7/2017
2017G141	31/7/2017
2017G141	9/8/2017
2017G141	18/8/2017
2017G141	27/8/2017
2017G141	7/9/2017
2017G142	3/8/2017
2017G142	15/8/2017
2017G143	5/8/2017
2017G143	16/8/2017
2017G143	17/8/2017
2017G143	28/8/2017
2017G143	17/9/2017
2017G144	7/8/2017
2017G144	28/8/2017
2017G144	7/9/2017
2017G144	17/9/2017
2017G145	8/8/2017
2017G145	18/8/2017
2017G145	28/8/2017
2017G145	7/9/2017
2017G145	27/9/2017
2017G146	8/8/2017
2017G146	17/8/2017
2017G146	19/8/2017
2017G147	6/8/2017
2017G147	21/8/2017
2017G147	1/9/2017
2017G147	12/9/2017
2017G147	24/9/2017
2017G148	9/8/2017
2017G148	23/8/2017
2017G148	17/9/2017
2017G149	9/8/2017
2017G149	20/8/2017
2017G149	30/8/2017
2017G150	12/8/2017
2017G150	13/10/2017

2017G152	23/8/2017
2017G152	1/9/2017
2017G152	29/9/2017
2017G152	12/10/2017
2017G152	18/10/2017
2017G153	29/8/2017
2017G153	21/9/2017
2017G154	31/8/2017
2017G155	1/9/2017
2017G155	20/10/2017
2017G156	3/9/2017
2017G156	13/9/2017
2017G156	3/10/2017
2017G157	14/9/2017
2017G157	24/9/2017
2017G157	5/10/2017
2017G157	15/10/2017
2017G158	15/9/2017
2017G158	9/10/2017
2017G158	19/10/2017
2017G160	4/10/2017
2017G161	5/10/2017
2017G162	10/10/2017
2017G163	19/10/2017
1994G022	20/6/2018
1994G022	11/7/2018
1994G022	21/7/2018
1994G022	1/8/2018
1994G022	10/8/2018
1995G024	21/4/2018
1995G024	24/5/2018
1995G024	2/6/2018
1995G024	12/6/2018
1995G024	22/6/2018
1995G024	2/7/2018
1995G024	12/7/2018
1995G095	28/8/2018
1995G095	16/9/2018
1995G095	26/9/2018
2000G004	20/2/2018
2000G004	15/3/2018
2000G004	28/3/2018
2000G004	10/5/2018
2000G015	28/7/2018
2000G015	8/8/2018
2000G019	20/6/2018

2000G019	3/7/2018
2000G019	16/7/2018
2000G026	6/8/2018
2000G026	17/8/2018
2000G026	28/8/2018
2000G026	8/9/2018
2000G026	18/9/2018
2000G026	30/9/2018
2000G026	10/10/2018
2000G026	25/10/2018
2001G009	16/7/2018
2003G014	11/7/2018
2003G014	24/7/2018
2003G014	4/8/2018
2006G037	5/7/2018
2006G037	18/7/2018
2006G037	29/7/2018
2006G037	8/8/2018
2006G037	19/8/2018
2006G037	29/8/2018
2006G037	28/9/2018
2006G037	8/10/2018
2006G037	27/10/2018
2007G009	2/5/2018
2007G009	23/5/2018
2007G009	2/6/2018
2007G009	12/6/2018
2007G009	24/6/2018
2007G009	4/7/2018
2008G001	9/8/2018
2008G001	29/8/2018
2008G003	23/6/2018
2008G003	3/7/2018
2008G003	12/7/2018
2008G003	21/7/2018
2009G014	4/4/2018
2009G035	23/4/2018
2009G035	4/5/2018
2009G035	27/5/2018
2009G035	6/6/2018
2009G035	16/6/2018
2009G035	7/7/2018
2009G035	27/7/2018
2009G049	23/8/2018
2009G049	11/9/2018
2009G049	20/9/2018

2009G049	10/10/2018
2009G049	31/10/2018
2009G035	27/6/2018
2010G014	11/4/2018
2010G014	21/4/2018
2010G014	1/5/2018
2010G014	11/5/2018
2010G014	21/5/2018
2010G014	31/5/2018
2010G014	8/6/2018
2010G062	23/4/2018
2010G062	3/5/2018
2010G062	14/5/2018
2010G062	23/5/2018
2010G062	2/6/2018
2010G062	12/6/2018
2010G062	2/7/2018
2011G029	23/5/2018
2011G029	2/6/2018
2011G029	12/6/2018
2011G029	23/6/2018
2011G029	13/7/2018
2011G069	12/9/2018
2011G069	10/10/2018
2011G082	30/5/2018
2011G082	9/6/2018
2011G082	19/6/2018
2011G082	21/7/2018
2011G082	31/7/2018
2012G028	1/4/2018
2012G028	12/4/2018
2012G028	13/5/2018
2012G028	23/5/2018
2012G029	23/2/2018
2012G029	4/5/2018
2012G029	16/4/2018
2012G036	21/4/2018
2012G036	3/5/2018
2012G036	25/5/2018
2012G036	5/6/2018
2012G036	17/6/2018
2012G036	11/7/2018
2012G036	21/7/2018
2012G036	2/8/2018
2012G041	27/4/2018
2012G041	7/5/2018

2012G041	19/5/2018
2012G041	29/5/2018
2012G041	8/6/2018
2013G017	3/4/2018
2013G017	14/4/2018
2013G017	3/5/2018
2013G017	22/5/2018
2013G017	1/6/2018
2013G019	24/4/2018
2013G101	29/5/2018
2013G101	9/6/2018
2013G101	1/7/2018
2013G101	11/7/2018
2013G101	22/7/2018
2013G101	1/8/2018
2013G101	12/8/2018
2013G101	23/8/2018
2013G125	4/5/2018
2013G125	13/5/2018
2013G125	23/5/2018
2013G125	11/6/2018
2013G125	21/6/2018
2014G038	27/5/2018
2014G038	30/6/2018
2014G038	10/7/2018
2014G038	21/7/2018
2014G099	16/4/2018
2014G099	6/5/2018
2014G099	15/5/2018
2014G099	24/5/2018
2014G099	3/6/2018
2014G109	14/6/2018
2014G109	27/6/2018
2014G109	6/7/2018
2014G109	27/7/2018
2014G109	5/8/2018
2014G109	14/8/2018
2014G109	1/9/2018
2015G003	10/3/2018
2015G003	23/3/2018
2015G003	5/5/2018
2015G003	15/5/2018
2015G040	12/7/2018
2015G040	22/7/2018
2015G045	11/6/2018
2015G045	21/6/2018

2015G045	13/8/2018
2015G049	24/5/2018
2015G049	3/6/2018
2015G049	13/6/2018
2015G049	3/7/2018
2015G049	13/7/2018
2015G125	27/6/2018
2015G125	30/8/2018
2015G125	9/9/2018
2015G125	22/9/2018
2015G131	3/5/2018
2015G131	12/5/2018
2015G131	21/5/2018
2015G131	30/5/2018
2015G131	27/6/2018
2015G131	7/7/2018
2016G011	18/6/2018
2016G011	7/8/2018
2016G163	28/4/2018
2017G133	14/7/2018
2017G133	4/8/2018
2017G133	14/8/2018
2017G133	24/8/2018
2017G133	3/9/2018
2018G001	14/1/2018
2018G001	27/2/2018
2018G002	15/1/2018
2018G002	9/2/2018
2018G002	18/3/2018
2018G002	31/3/2018
2018G002	13/4/2018
2018G003	18/1/2018
2018G004	21/1/2018
2018G004	10/6/2018
2018G006	24/2/2018
2018G007	10/3/2018
2018G007	21/3/2018
2018G007	31/3/2018
2018G007	19/4/2018
2018G007	9/5/2018
2018G007	20/5/2018
2018G007	30/5/2018
2018G008	11/3/2018
2018G009	14/3/2018
2018G009	28/4/2018
2018G009	9/5/2018

2018G010	16/3/2018
2018G011	16/3/2018
2018G011	27/3/2018
2018G011	8/4/2018
2018G011	19/4/2018
2018G011	30/4/2018
2018G012	21/3/2018
2018G012	2/4/2018
2018G012	6/5/2018
2018G012	17/5/2018
2018G013	26/3/2018
2018G013	8/4/2018
2018G013	21/4/2018
2018G014	28/3/2018
2018G014	9/4/2018
2018G014	21/4/2018
2018G014	3/5/2018
2018G014	17/5/2018
2018G015	30/3/2018
2018G015	9/4/2018
2018G016	30/3/2018
2018G017	4/4/2018
2018G017	25/4/2018
2018G017	5/5/2018
2018G017	9/5/2018
2018G018	4/4/2018
2018G018	18/4/2018
2018G019	5/4/2018
2018G019	16/4/2018
2018G019	28/4/2018
2018G020	8/4/2018
2018G020	19/4/2018
2018G020	30/4/2018
2018G020	10/5/2018
2018G020	19/5/2018
2018G020	28/5/2018
2018G020	6/6/2018
2018G021	8/4/2018
2018G021	18/4/2018
2018G021	28/4/2018
2018G021	8/5/2018
2018G021	18/5/2018
2018G021	29/5/2018
2018G021	7/6/2018
2018G021	17/6/2018
2018G021	29/6/2018

2018G022	9/4/2018
2018G022	20/4/2018
2018G022	1/5/2018
2018G022	11/5/2018
2018G022	21/5/2018
2018G022	31/5/2018
2018G022	9/6/2018
2018G022	18/6/2018
2018G022	28/6/2018
2018G023	10/4/2018
2018G023	2/5/2018
2018G023	21/5/2018
2018G023	1/6/2018
2018G023	13/6/2018
2018G024	11/4/2018
2018G024	4/5/2018
2018G024	13/5/2018
2018G024	1/6/2018
2018G025	11/4/2018
2018G025	1/5/2018
2018G025	11/5/2018
2018G025	22/5/2018
2018G025	31/5/2018
2018G025	9/6/2018
2018G026	15/4/2018
2018G026	26/4/2018
2018G026	23/6/2018
2018G027	18/4/2018
2018G027	23/5/2018
2018G028	21/4/2018
2018G028	23/5/2018
2018G028	14/6/2018
2018G029	23/4/2018
2018G029	5/5/2018
2018G029	17/5/2018
2018G029	28/5/2018
2018G029	7/6/2018
2018G029	18/6/2018
2018G029	11/7/2018
2018G030	23/4/2018
2018G030	14/5/2018
2018G030	23/5/2018
2018G030	1/6/2018
2018G030	10/6/2018
2018G030	20/6/2018
2018G031	25/4/2018

2018G032	26/4/2018
2018G032	5/6/2018
2018G033	26/4/2018
2018G033	7/5/2018
2018G033	18/5/2018
2018G033	29/5/2018
2018G033	8/6/2018
2018G033	19/6/2018
2018G034	27/4/2018
2018G034	7/5/2018
2018G034	17/5/2018
2018G034	3/6/2018
2018G034	11/6/2018
2018G034	21/6/2018
2018G034	2/7/2018
2018G035	27/4/2018
2018G035	6/5/2018
2018G035	17/5/2018
2018G035	28/5/2018
2018G035	15/6/2018
2018G035	25/6/2018
2018G036	28/4/2018
2018G036	8/5/2018
2018G036	18/5/2018
2018G036	27/5/2018
2018G036	7/6/2018
2018G037	30/4/2018
2018G037	11/5/2018
2018G037	21/5/2018
2018G037	31/5/2018
2018G038	30/4/2018
2018G038	10/5/2018
2018G038	21/5/2018
2018G038	31/5/2018
2018G038	10/6/2018
2018G038	22/6/2018
2018G038	4/7/2018
2018G039	2/5/2018
2018G039	24/5/2018
2018G039	3/6/2018
2018G039	14/6/2018
2018G040	3/5/2018
2018G040	23/5/2018
2018G040	2/7/2018
2018G041	6/5/2018
2018G041	16/5/2018

2018G041	4/6/2018
2018G041	13/6/2018
2018G041	2/7/2018
2018G042	6/5/2018
2018G042	17/5/2018
2018G042	27/5/2018
2018G042	6/6/2018
2018G042	17/6/2018
2018G042	29/6/2018
2018G042	10/7/2018
2018G043	7/5/2018
2018G043	26/5/2018
2018G043	4/6/2018
2018G043	12/6/2018
2018G043	21/6/2018
2018G043	30/6/2018
2018G043	10/7/2018
2018G044	7/5/2018
2018G044	18/5/2018
2018G044	28/5/2018
2018G044	7/6/2018
2018G044	27/6/2018
2018G045	8/5/2018
2018G045	3/7/2018
2018G045	4/6/2018
2018G046	11/5/2018
2018G046	23/5/2018
2018G046	2/6/2018
2018G046	12/6/2018
2018G046	23/6/2018
2018G046	4/7/2018
2018G047	12/5/2018
2018G047	23/5/2018
2018G047	3/6/2018
2018G048	15/5/2018
2018G048	2/6/2018
2018G048	12/6/2018
2018G048	23/6/2018
2018G048	2/7/2018
2018G048	15/7/2018
2018G049	15/5/2018
2018G049	24/5/2018
2018G049	12/6/2018
2018G050	17/5/2018
2018G050	18/5/2018
2018G050	29/5/2018

2018G050	8/6/2018
2018G050	18/6/2018
2018G050	1/7/2018
2018G051	18/5/2018
2018G052	19/5/2018
2018G052	2/6/2018
2018G052	6/7/2018
2018G052	7/7/2018
2018G053	21/5/2018
2018G053	30/5/2018
2018G053	8/6/2018
2018G053	18/6/2018
2018G053	28/6/2018
2018G053	7/7/2018
2018G053	27/7/2018
2018G053	6/8/2018
2018G054	22/5/2018
2018G054	2/6/2018
2018G054	12/6/2018
2018G054	3/7/2018
2018G054	14/7/2018
2018G055	23/5/2018
2018G055	1/6/2018
2018G055	10/6/2018
2018G055	19/6/2018
2018G055	28/6/2018
2018G056	23/5/2018
2018G056	2/6/2018
2018G056	12/6/2018
2018G056	23/6/2018
2018G056	4/7/2018
2018G056	15/7/2018
2018G057	23/5/2018
2018G057	2/6/2018
2018G057	12/6/2018
2018G057	21/6/2018
2018G057	2/7/2018
2018G057	12/7/2018
2018G057	22/7/2018
2018G058	24/5/2018
2018G058	5/6/2018
2018G058	15/6/2018
2018G058	26/6/2018
2018G058	6/7/2018
2018G058	16/7/2018
2018G058	27/7/2018

2018G059	27/5/2018
2018G059	5/6/2018
2018G059	15/6/2018
2018G059	25/6/2018
2018G059	7/7/2018
2018G060	28/5/2018
2018G060	5/6/2018
2018G060	14/6/2018
2018G060	2/7/2018
2018G060	12/7/2018
2018G061	28/5/2018
2018G061	8/6/2018
2018G062	29/5/2018
2018G063	1/6/2018
2018G063	27/6/2018
2018G063	10/7/2018
2018G063	21/7/2018
2018G063	2/8/2018
2018G063	14/8/2018
2018G064	1/6/2018
2018G065	3/6/2018
2018G065	15/7/2018
2018G065	26/7/2018
2018G065	7/8/2018
2018G066	3/6/2018
2018G066	13/6/2018
2018G066	23/6/2018
2018G066	3/7/2018
2018G066	13/7/2018
2018G066	22/7/2018
2018G067	4/6/2018
2018G067	14/6/2018
2018G068	4/6/2018
2018G068	15/6/2018
2018G068	27/6/2018
2018G068	22/7/2018
2018G069	4/6/2018
2018G069	12/6/2018
2018G069	20/6/2018
2018G069	1/7/2018
2018G069	11/7/2018
2018G070	5/6/2018
2018G070	16/6/2018
2018G070	27/6/2018
2018G070	27/6/2018
2018G070	7/7/2018

2018G070	9/7/2018
2018G070	18/7/2018
2018G071	5/6/2018
2018G071	14/6/2018
2018G072	5/6/2018
2018G072	16/6/2018
2018G072	27/6/2018
2018G072	6/7/2018
2018G072	30/7/2018
2018G073	5/6/2018
2018G073	16/6/2018
2018G073	27/6/2018
2018G073	10/7/2018
2018G074	5/6/2018
2018G074	27/6/2018
2018G074	18/7/2018
2018G075	5/6/2018
2018G075	14/6/2018
2018G076	5/6/2018
2018G076	16/6/2018
2018G076	28/6/2018
2018G077	5/6/2018
2018G078	6/6/2018
2018G078	16/6/2018
2018G078	27/6/2018
2018G078	7/7/2018
2018G078	17/7/2018
2018G078	6/8/2018
2018G078	18/8/2018
2018G079	6/6/2018
2018G080	6/6/2018
2018G080	16/6/2018
2018G080	27/6/2018
2018G080	9/7/2018
2018G081	6/6/2018
2018G081	18/6/2018
2018G081	29/6/2018
2018G081	12/7/2018
2018G082	8/6/2018
2018G082	18/6/2018
2018G082	6/7/2018
2018G083	8/6/2018
2018G083	18/6/2018
2018G083	29/6/2018
2018G083	9/7/2018
2018G083	20/7/2018

2018G083	31/7/2018
2018G084	9/6/2018
2018G084	2/7/2018
2018G084	13/7/2018
2018G084	27/7/2018
2018G084	26/7/2018
2018G084	6/8/2018
2018G085	10/6/2018
2018G085	20/6/2018
2018G085	1/7/2018
2018G085	11/7/2018
2018G086	10/6/2018
2018G086	23/6/2018
2018G086	17/7/2018
2018G087	12/6/2018
2018G087	24/6/2018
2018G088	12/6/2018
2018G088	22/6/2018
2018G088	3/7/2018
2018G089	13/6/2018
2018G090	13/6/2018
2018G090	23/6/2018
2018G090	3/7/2018
2018G090	13/7/2018
2018G090	24/7/2018
2018G091	14/6/2018
2018G092	15/6/2018
2018G092	4/7/2018
2018G092	13/7/2018
2018G092	22/7/2018
2018G092	31/7/2018
2018G093	15/6/2018
2018G093	26/7/2018
2018G094	17/6/2018
2018G094	30/6/2018
2018G095	17/5/2018
2018G095	18/6/2018
2018G095	29/6/2018
2018G095	8/7/2018
2018G095	17/7/2018
2018G095	26/7/2018
2018G095	4/8/2018
2018G096	20/6/2018
2018G097	20/6/2018
2018G097	11/7/2018
2018G097	21/7/2018

2018G098	22/6/2018
2018G098	3/7/2018
2018G099	23/6/2018
2018G099	16/7/2018
2018G099	27/7/2018
2018G099	7/8/2018
2018G100	23/6/2018
2018G100	3/7/2018
2018G100	12/7/2018
2018G100	21/7/2018
2018G100	31/7/2018
2018G100	9/8/2018
2018G100	20/8/2018
2018G101	23/6/2018
2018G101	19/7/2018
2018G102	24/6/2018
2018G103	25/6/2018
2018G103	17/7/2018
2018G103	29/7/2018
2018G103	9/8/2018
2018G104	26/6/2018
2018G104	15/7/2018
2018G104	27/7/2018
2018G104	4/8/2018
2018G104	14/8/2018
2018G104	24/8/2018
2018G104	2/9/2018
2018G104	11/9/2018
2018G105	27/6/2018
2018G105	7/7/2018
2018G106	27/6/2018
2018G106	28/7/2018
2018G107	29/6/2018
2018G107	1/7/2018
2018G107	12/7/2018
2018G107	21/7/2018
2018G107	31/7/2018
2018G107	10/8/2018
2018G108	19/6/2018
2018G109	30/6/2018
2018G109	10/7/2018
2018G109	21/7/2018
2018G109	14/8/2018
2018G110	30/6/2018
2018G110	11/7/2018
2018G110	22/7/2018

2018G111	1/7/2018
2018G112	3/7/2018
2018G112	13/7/2018
2018G112	24/7/2018
2018G112	3/8/2018
2018G113	4/7/2018
2018G113	17/7/2018
2018G113	31/7/2018
2018G114	4/7/2018
2018G114	18/7/2018
2018G114	30/7/2018
2018G114	9/8/2018
2018G114	29/8/2018
2018G114	21/9/2018
2018G115	6/7/2018
2018G116	9/7/2018
2018G118	12/7/2018
2018G119	12/7/2018
2018G120	25/8/2018
2018G120	13/7/2018
2018G120	24/7/2018
2018G120	14/8/2018
2018G121	13/7/2018
2018G121	7/8/2018
2018G122	20/7/2018
2018G122	31/7/2018
2018G122	9/8/2018
2018G122	30/8/2018
2018G122	9/9/2018
2018G122	29/9/2018
2018G123	20/7/2018
2018G125	13/7/2018
2018G125	23/7/2018
2018G125	10/8/2018
2018G125	20/8/2018
2018G125	31/8/2018
2018G126	24/7/2018
2018G127	24/7/2018
2018G127	2/8/2018
2018G127	12/8/2018
2018G127	21/8/2018
2018G127	19/9/2018
2018G128	27/7/2018
2018G128	5/8/2018
2018G128	18/5/2018
2018G128	25/8/2018

2018G128	15/9/2018
2018G128	28/9/2018
2018G130	29/7/2018
2018G130	9/8/2018
2018G131	31/7/2018
2018G131	13/8/2018
2018G131	24/8/2018
2018G131	26/9/2018
2018G131	7/10/2018
2018G131	19/10/2018
2018G132	1/8/2018
2018G132	14/8/2018
2018G132	26/8/2018
2018G133	2/8/2018
2018G133	12/8/2018
2018G134	2/8/2018
2018G134	13/8/2018
2018G134	24/8/2018
2018G135	6/8/2018
2018G135	17/8/2018
2018G136	6/8/2018
2018G136	16/8/2018
2018G137	6/8/2018
2018G138	9/8/2018
2018G139	11/8/2018
2018G139	22/8/2018
2018G139	2/9/2018
2018G139	13/9/2018
2018G140	23/8/2018
2018G142	28/8/2018
2018G142	9/9/2018
2018G142	19/9/2018
2018G143	1/9/2018
2018G143	22/9/2018
2018G143	2/10/2018
2018G143	12/10/2018
2018G144	31/8/2018
2018G144	27/9/2018
2018G145	7/9/2018
2018G146	12/9/2018
2018G146	23/9/2018
2018G147	12/9/2018
2018G148	17/9/2018
2018G148	28/9/2018
2018G148	17/10/2018
2018G149	19/9/2018

2018G149	1/10/2018
2018G150	20/9/2018
2018G151	15/9/2018
2018G151	28/9/2018
2018G152	25/9/2018
2018G152	15/10/2018
2018G153	28/9/2018
2018G154	7/2/2018
2018G155	20/6/2018
2018G156	15/7/2018
2018G157	20/7/2018
2018G158	28/7/2018
2018G158	7/8/2018
2018G159	25/8/2018
2018G160	28/9/2018
2018G151	22/10/2018
1993G019	2/8/2019
1993G019	14/8/2019
1993G019	24/8/2019
1993G019	4/9/2019
1993G019	14/9/2019
1993G019	25/9/2019
1993G019	7/10/2019
1993G109	18/10/2019
1994G060	31/8/2019
1995G005	3/3/2019
1995G006	27/9/2019
1995G006	7/10/2019
1995G006	25/10/2019
1995G006	4/11/2019
1995G006	13/11/2019
1995G023	24/5/2019
1995G023	3/6/2019
1995G023	13/6/2019
1995G023	23/6/2019
1995G023	2/7/2019
1995G023	11/7/2019
1999G005	23/3/2019
1999G005	1/4/2019
1999G005	21/4/2019
1999G005	30/4/2019
1999G005	9/5/2019
2001G036	22/6/2019
2001G036	29/8/2019
2001G037	25/6/2019
2001G037	8/7/2019

2001G037	21/7/2019
2004G036	28/6/2019
2004G036	8/7/2019
2004G036	17/7/2019
2004G036	26/7/2019
2008G004	12/5/2019
2008G004	23/5/2019
2008G004	2/6/2019
2008G004	13/6/2019
2008G004	23/6/2019
2008G004	24/7/2019
2008G012	7/4/2019
2008G012	9/5/2019
2008G012	12/6/2019
2008G036	6/4/2019
2008G036	16/4/2019
2008G049	19/5/2019
2008G063	15/4/2019
2008G063	28/4/2019
2008G063	9/5/2019
2008G063	19/5/2019
2008G063	31/5/2019
2008G063	11/6/2019
2009G059	23/6/2019
2009G059	3/7/2019
2009G059	14/7/2019
2009G059	24/7/2019
2009G059	3/8/2019
2009G059	13/8/2019
2009G059	23/8/2019
2010G032	19/4/2019
2010G032	30/4/2019
2010G032	9/5/2019
2010G032	19/5/2019
2010G032	29/5/2019
2010G032	7/6/2019
2010G032	17/6/2019
2010G032	27/6/2019
2010G032	7/7/2019
2010G032	18/7/2019
2010G075	2/5/2019
2010G075	13/5/2019
2010G075	22/5/2019
2010G075	1/6/2019
2010G075	20/6/2019
2010G075	30/6/2019

2010G075	10/7/2019
2010G075	19/7/2019
2010G083	17/6/2019
2010G089	15/4/2019
2010G089	6/5/2019
2010G089	17/5/2019
2010G089	27/5/2019
2010G089	7/6/2019
2010G089	16/6/2019
2010G089	26/6/2019
2010G095	18/5/2019
2010G095	28/5/2019
2010G097	25/7/2019
2010G240	18/9/2019
2011G036	27/7/2019
2011G036	6/8/2019
2011G044	12/4/2019
2011G044	24/4/2019
2011G044	4/5/2019
2011G044	15/5/2019
2011G044	26/5/2019
2011G044	15/6/2019
2011G044	27/6/2019
2011G044	10/7/2019
2011G081	19/6/2019
2011G081	2/7/2019
2011G081	24/7/2019
2011G081	3/8/2019
2011G095	21/7/2019
2011G178	7/8/2019
2012G011	16/5/2019
2012G016	13/4/2019
2012G022	12/5/2019
2012G022	22/5/2019
2012G022	1/6/2019
2012G022	14/6/2019
2012G047	9/5/2019
2012G047	19/5/2019
2012G047	29/5/2019
2012G047	18/6/2019
2012G047	28/6/2019
2012G047	8/7/2019
2012G050	11/5/2019
2012G050	22/5/2019
2012G050	2/6/2019
2012G050	12/6/2019

2012G050	23/6/2019
2012G050	3/7/2019
2012G050	15/7/2019
2013G012	16/3/2019
2013G012	26/3/2019
2013G012	6/4/2019
2013G012	16/4/2019
2013G012	26/4/2019
2013G012	25/5/2019
2013G020	26/3/2019
2013G020	6/4/2019
2013G020	18/4/2019
2013G020	28/4/2019
2013G020	9/5/2019
2013G020	19/5/2019
2013G020	29/5/2019
2013G020	10/6/2019
2013G026	31/5/2019
2013G027	13/3/2019
2013G027	23/3/2019
2013G027	3/4/2019
2013G027	12/4/2019
2013G027	23/4/2019
2013G027	2/5/2019
2013G027	21/5/2019
2013G027	4/6/2019
2013G034	16/4/2019
2013G034	28/4/2019
2013G034	10/5/2019
2013G034	20/5/2019
2013G034	30/5/2019
2013G034	10/6/2019
2013G034	21/6/2019
2013G034	2/7/2019
2013G056	19/3/2019
2013G074	4/5/2019
2013G074	13/5/2019
2013G074	23/5/2019
2013G074	1/6/2019
2013G074	30/6/2019
2013G074	1/7/2019
2013G074	13/7/2019
2013G094	1/5/2019
2013G094	10/5/2019
2013G094	20/5/2019
2013G094	29/5/2019

2013G094	7/6/2019
2013G094	16/6/2019
2013G094	4/7/2019
2013G110	18/3/2019
2013G110	27/3/2019
2013G110	6/4/2019
2013G110	16/4/2019
2013G110	25/4/2019
2013G110	4/5/2019
2013G110	14/5/2019
2013G115	21/5/2019
2013G115	3/6/2019
2013G115	17/6/2019
2013G115	11/7/2019
2013G115	22/7/2019
2013G145	18/8/2019
2013G148	23/5/2019
2013G148	25/6/2019
2013G148	4/7/2019
2013G148	16/7/2019
2013G152	3/8/2019
2013G152	14/8/2019
2013G152	3/9/2019
2013G160	24/7/2019
2013G160	12/8/2019
2013G160	21/8/2019
2013G169	14/8/2019
2013G169	2/9/2019
2013G169	12/9/2019
2013G169, 2	23/8/2019
2013G171	21/9/2019
2013G171	3/10/2019
2013G171	13/10/2019
2014G003	1/4/2019
2014G003	10/4/2019
2014G003	20/4/2019
2014G003	1/5/2019
2014G003	10/5/2019
2014G006	3/4/2019
2014G006	15/4/2019
2014G027	5/8/2019
2014G028	19/3/2019
2014G028	30/3/2019
2014G028	8/4/2019
2014G028	17/4/2019
2014G028	28/4/2019

2014G028	7/5/2019
2014G028	16/5/2019
2014G028	25/5/2019
2014G045	9/5/2019
2014G045	19/5/2019
2014G045	28/5/2019
2014G045	18/6/2019
2014G045	29/6/2019
2014G045	18/4/2019
2014G048	3/4/2019
2014G058	7/6/2019
2014G058	18/6/2019
2014G058	29/6/2019
2014G058	10/7/2019
2014G058	20/7/2019
2014G058	30/7/2019
2014G058	9/8/2019
2014G059	22/4/2019
2014G059	12/5/2019
2014G059	21/5/2019
2014G073	2/6/2019
2014G073	12/6/2019
2014G073	14/7/2019
2014G073	24/7/2019
2014G079	21/5/2019
2014G079	1/6/2019
2014G079	12/6/2019
2014G079	25/6/2019
2014G079	26/7/2019
2014G098	13/5/2019
2014G098	5/6/2019
2014G098	15/6/2019
2014G098	5/7/2019
2014G098	16/7/2019
2014G100	18/5/2019
2014G100	28/5/2019
2014G100	16/6/2019
2014G100	4/7/2019
2014G100	14/7/2019
2014G121	8/7/2019
2014G121	30/7/2019
2014G121	9/8/2019
2014G121	21/8/2019
2014G121	1/9/2019
2014G121	12/9/2019
2014G122	19/6/2019

2014G122	10/7/2019
2014G122	20/7/2019
2014G124	19/6/2019
2014G124	30/6/2019
2015G010	30/1/2019
2015G010	19/3/2019
2015G010	2/4/2019
2015G014	6/4/2019
2015G014	18/4/2019
2015G014	1/5/2019
2015G014	10/5/2019
2015G014	20/5/2019
2015G014	31/5/2019
2015G014	10/6/2019
2015G014	21/6/2019
2015G014	2/7/2019
2015G016	8/3/2019
2015G016	19/3/2019
2015G016	8/4/2019
2015G036	3/4/2019
2015G036	13/4/2019
2015G036	23/4/2019
2015G036	12/6/2019
2015G050	9/5/2019
2015G050	19/5/2019
2015G050	6/6/2019
2015G050	15/6/2019
2015G050	24/6/2019
2015G050	3/7/2019
2015G050	12/7/2019
2015G056	9/3/2019
2015G056	29/3/2019
2015G056	7/4/2019
2015G056	28/4/2019
2015G056	7/5/2019
2015G056	17/5/2019
2015G056	22/5/2019
2015G056	27/5/2019
2015G056	1/6/2019
2015G056	23/6/2019
2015G056	4/7/2019
2015G062	15/4/2019
2015G062	24/4/2019
2015G062	23/5/2019
2015G111	19/7/2019
2015G111	30/7/2019

2015G111	10/8/2019
2015G111	23/8/2019
2015G111	15/9/2019
2015G111	26/9/2019
2015G111	6/10/2019
2015G116	17/7/2019
2015G128	27/6/2019
2015G128	29/7/2019
2015G128	19/8/2019
2015G128	30/8/2019
2016G002	6/4/2019
2016G002	15/4/2019
2016G002	24/4/2019
2016G002	11/5/2019
2016G002	23/5/2019
2016G007	19/3/2019
2016G008	14/4/2019
2016G014	4/3/2019
2016G014	14/3/2019
2016G014	24/4/2019
2016G026	23/3/2019
2016G026	13/4/2019
2016G026	25/4/2019
2016G026	5/5/2019
2016G026	16/5/2019
2016G026	27/5/2019
2016G034	7/4/2019
2016G034	26/4/2019
2016G034	6/5/2019
2016G034	16/5/2019
2016G034	26/5/2019
2016G038	29/3/2019
2016G038	8/4/2019
2016G038	18/4/2019
2016G038	9/5/2019
2016G094	4/5/2019
2016G097	7/3/2019
2016G097	19/3/2019
2016G097	28/4/2019
2016G108	11/3/2019
2016G108	31/3/2019
2016G108	9/4/2019
2016G108	18/4/2019
2016G108	27/4/2019
2016G108	6/5/2019
2016G108	17/5/2019

2016G137	14/10/2019
2016G137	24/10/2019
2016G153	11/5/2019
2016G199	26/5/2019
2016G199	7/6/2019
2016G199	7/7/2019
2016G199	15/7/2019
2016G199	26/7/2019
2016G205	17/8/2019
2016G205, 2(	4/7/2019
2016G236	14/6/2019
2016G236	24/6/2019
2016G236	4/7/2019
2016G236	15/7/2019
2016G236	25/7/2019
2016G236	5/8/2019
2016G236	16/8/2019
2016G236	26/8/2019
2016G236	9/9/2019
2016G283	26/5/2019
2016G283	17/6/2019
2016G283	29/6/2019
2016G283	10/7/2019
2016G301	23/5/2019
2016G301	4/6/2019
2016G301	29/6/2019
2016G301	11/7/2019
2016G301	13/7/2019
2016G317	7/8/2019
2016G317	18/8/2019
2016G317	27/8/2019
2016G317	6/9/2019
2016G317	15/9/2019
2016G317	26/9/2019
2017G002	29/3/2019
2017G002	17/5/2019
2017G074	2/8/2019
2017G093	21/6/2019
2017G093	30/6/2019
2017G093	11/7/2019
2017G093	22/7/2019
2017G093	2/8/2019
2017G093	23/8/2019
2017G093	5/9/2019
2019G001	14/1/2019
2019G001	25/1/2019

2019G001	6/2/2019
2019G001	16/2/2019
2019G002	22/1/2019
2019G003	14/1/2019
2019G004	22/2/2019
2019G004	11/4/2019
2019G004	22/4/2019
2019G004	10/2/2019
2019G005	17/2/2019
2019G006	17/2/2019
2019G006	12/3/2019
2019G006	23/3/2019
2019G006	23/3/2019
2019G006	13/4/2019
2019G006	23/4/2019
2019G006	2/5/2019
2019G007	18/2/2019
2019G007	1/3/2019
2019G008	28/2/2019
2019G008	10/3/2019
2019G008	22/3/2019
2019G009	1/3/2019
2019G009	18/4/2019
2019G010	12/3/2019
2019G010	22/3/2019
2019G010	1/4/2019
2019G010	11/4/2019
2019G010	21/4/2019
2019G011	13/3/2019
2019G011	3/4/2019
2019G011	14/4/2019
2019G011	25/4/2019
2019G011	7/5/2019
2019G011	17/5/2019
2019G011	28/5/2019
2019G012	14/3/2019
2019G012	24/3/2019
2019G012	3/4/2019
2019G013	20/3/2019
2019G013	9/4/2019
2019G013	19/4/2019
2019G013	29/4/2019
2019G013	19/5/2019
2019G013	21/5/2019
2019G013	28/5/2019
2019G014	20/3/2019

2019G014	30/3/2019
2019G014	25/5/2019
2019G015	20/3/2019
2019G015	12/4/2019
2019G015	23/4/2019
2019G015	3/5/2019
2019G015	13/5/2019
2019G015	23/5/2019
2019G015	4/6/2019
2019G016	21/3/2019
2019G017	22/3/2019
2019G017	1/4/2019
2019G017	10/4/2019
2019G017	20/4/2019
2019G017	9/5/2019
2019G018	22/3/2019
2019G018	3/4/2019
2019G019	22/3/2019
2019G019	20/5/2019
2019G020	23/3/2019
2019G020	12/4/2019
2019G020	22/4/2019
2019G020	1/5/2019
2019G020	20/5/2019
2019G020	30/5/2019
2019G020	8/6/2019
2019G021	23/3/2019
2019G021	4/4/2019
2019G021	16/4/2019
2019G021	29/4/2019
2019G021	11/5/2019
2019G021	26/5/2019
2019G022	23/3/2019
2019G022	15/4/2019
2019G022	25/4/2019
2019G022	5/5/2019
2019G022	16/5/2019
2019G023	25/3/2019
2019G024	25/3/2019
2019G024	6/4/2019
2019G024	17/4/2019
2019G024	5/5/2019
2019G024	17/5/2019
2019G025	27/3/2019
2019G025	8/4/2019
2019G025	19/4/2019

2019G025	18/5/2019
2019G025	28/5/2019
2019G026	28/3/2019
2019G026	7/4/2019
2019G026	18/4/2019
2019G026	28/4/2019
2019G026	7/5/2019
2019G026	19/5/2019
2019G026	29/5/2019
2019G026	21/6/2019
2019G027	29/3/2019
2019G027	8/4/2019
2019G027	10/5/2019
2019G027	20/5/2019
2019G028	30/3/2019
2019G029	31/3/2019
2019G029	11/4/2019
2019G030	31/3/2019
2019G030	13/4/2019
2019G030	22/4/2019
2019G030	2/5/2019
2019G030	12/5/2019
2019G030	22/5/2019
2019G030	31/5/2019
2019G030	12/6/2019
2019G031	1/4/2019
2019G031	23/4/2019
2019G031	4/5/2019
2019G032	1/4/2019
2019G032	12/4/2019
2019G032	13/5/2019
2019G032	23/5/2019
2019G033	3/4/2019
2019G033	24/4/2019
2019G033	6/5/2019
2019G033	16/5/2019
2019G034	6/3/2019
2019G034	4/4/2019
2019G034	24/4/2019
2019G035	4/4/2019
2019G036	5/4/2019
2019G036	16/4/2019
2019G036	5/5/2019
2019G037	7/4/2019
2019G037	2/5/2019
2019G037	12/5/2019

2019G037	31/5/2019
2019G037	20/6/2019
2019G037	17/6/2019
2019G038	7/4/2019
2019G038	18/4/2019
2019G038	28/4/2019
2019G038	30/5/2019
2019G039	8/4/2019
2019G039	8/5/2019
2019G039	18/5/2019
2019G039	7/6/2019
2019G040	8/4/2019
2019G041	8/4/2019
2019G041	18/4/2019
2019G041	27/4/2019
2019G041	17/5/2019
2019G041	27/5/2019
2019G042	9/4/2019
2019G042	4/5/2019
2019G042	26/5/2019
2019G042	6/6/2019
2019G042	18/6/2019
2019G043	11/4/2019
2019G043	25/4/2019
2019G043	8/6/2019
2019G044	11/4/2019
2019G044	20/4/2019
2019G044	28/4/2019
2019G045	13/4/2019
2019G045	24/4/2019
2019G045	5/5/2019
2019G045	14/5/2019
2019G045	25/5/2019
2019G045	1/6/2019
2019G045	4/6/2019
2019G045	14/6/2019
2019G045	24/6/2019
2019G045	4/7/2019
2019G046	13/4/2019
2019G047	14/4/2019
2019G047	26/4/2019
2019G047	19/5/2019
2019G047	30/5/2019
2019G047	10/6/2019
2019G048	15/4/2019
2019G048	6/5/2019

2019G048	27/5/2019
2019G049	16/4/2019
2019G049	25/4/2019
2019G049	25/5/2019
2019G049	5/6/2019
2019G049	5/6/2019
2019G049	26/6/2019
2019G050	17/4/2019
2019G050	27/4/2019
2019G050	8/5/2019
2019G050	18/5/2019
2019G050	28/5/2019
2019G050	28/5/2019
2019G050	8/6/2019
2019G050	18/6/2019
2019G050	27/6/2019
2019G050	4/8/2019
2019G051	17/4/2019
2019G051	30/4/2019
2019G051	22/5/2019
2019G051	1/6/2019
2019G051	10/6/2019
2019G051	19/6/2019
2019G052	18/4/2019
2019G052	9/5/2019
2019G053	18/4/2019
2019G054	18/4/2019
2019G054	28/4/2019
2019G054	24/6/2019
2019G055	18/4/2019
2019G055	30/4/2019
2019G055	10/5/2019
2019G055	21/5/2019
2019G055	31/5/2019
2019G055	10/6/2019
2019G056	19/4/2019
2019G056	21/5/2019
2019G056	31/5/2019
2019G056	21/6/2019
2019G057	19/4/2019
2019G057	10/5/2019
2019G057	22/5/2019
2019G057	1/6/2019
2019G057	12/6/2019
2019G057	23/6/2019
2019G057	4/7/2019

2019G058	19/4/2019
2019G058	13/5/2019
2019G058	25/5/2019
2019G058	6/6/2019
2019G058	17/6/2019
2019G058	30/6/2019
2019G059	20/4/2019
2019G060	20/4/2019
2019G061	21/4/2019
2019G061	1/5/2019
2019G061	1/6/2019
2019G062	21/4/2019
2019G062	12/5/2019
2019G062	22/5/2019
2019G062	4/6/2019
2019G062	16/6/2019
2019G063	22/4/2019
2019G063	3/5/2019
2019G063	13/5/2019
2019G063	23/5/2019
2019G063	2/6/2019
2019G064	22/4/2019
2019G065	23/4/2019
2019G065	3/5/2019
2019G065	13/5/2019
2019G065	23/5/2019
2019G065	2/6/2019
2019G066	22/4/2019
2019G066	23/4/2019
2019G067	23/4/2019
2019G067	3/5/2019
2019G067	15/5/2019
2019G067	25/5/2019
2019G067	4/6/2019
2019G067	26/6/2019
2019G068	23/4/2019
2019G068	13/5/2019
2019G068	24/5/2019
2019G069	23/4/2019
2019G069	3/5/2019
2019G069	13/5/2019
2019G069	23/5/2019
2019G070	24/4/2019
2019G070	5/5/2019
2019G070	25/5/2019
2019G070	4/6/2019

2019G070	15/6/2019
2019G070	24/6/2019
2019G070	26/6/2019
2019G070	7/7/2019
2019G070	12/7/2019
2019G070	18/7/2019
2019G071	28/4/2019
2019G071	11/5/2019
2019G071	29/5/2019
2019G071	10/6/2019
2019G071	25/7/2019
2019G071	10/8/2019
2019G072	29/4/2019
2019G072	27/5/2019
2019G073	30/4/2019
2019G073	11/5/2019
2019G073	20/5/2019
2019G073	29/5/2019
2019G073	7/6/2019
2019G073	17/6/2019
2019G073	26/6/2019
2019G074	30/4/2019
2019G075	30/4/2019
2019G075	10/5/2019
2019G075	20/5/2019
2019G075	13/6/2019
2019G075	24/6/2019
2019G075	5/7/2019
2019G076	10/5/2019
2019G076	20/5/2019
2019G076	30/5/2019
2019G077	1/5/2019
2019G077	10/5/2019
2019G077	20/5/2019
2019G077	1/6/2019
2019G077	11/6/2019
2019G078	1/5/2019
2019G078	12/5/2019
2019G078	23/5/2019
2019G078	3/6/2019
2019G078	13/6/2019
2019G078	25/6/2019
2019G078	6/7/2019
2019G078	18/7/2019
2019G079	3/5/2019
2019G079	28/5/2019

2019G079	5/7/2019
2019G080	5/5/2019
2019G080	26/5/2019
2019G080	5/6/2019
2019G080	15/6/2019
2019G080	25/6/2019
2019G081	6/5/2019
2019G081	16/5/2019
2019G081	27/5/2019
2019G082	6/5/2019
2019G082	16/5/2019
2019G082	18/5/2019
2019G082	8/6/2019
2019G082	18/6/2019
2019G082	29/6/2019
2019G082	11/7/2019
2019G083	6/5/2019
2019G083	25/5/2019
2019G083	6/6/2019
2019G083	16/6/2019
2019G083	26/6/2019
2019G083	6/7/2019
2019G084	6/5/2019
2019G084	16/5/2019
2019G084	27/5/2019
2019G084	5/6/2019
2019G084	14/6/2019
2019G084	23/6/2019
2019G085	6/5/2019
2019G085	17/5/2019
2019G085	5/6/2019
2019G086	7/5/2019
2019G086	29/5/2019
2019G086	8/6/2019
2019G086	19/6/2019
2019G086	11/7/2019
2019G087	7/5/2019
2019G087	19/5/2019
2019G087	7/6/2019
2019G087	26/6/2019
2019G087	9/7/2019
2019G088	7/5/2019
2019G088	18/5/2019
2019G088	21/6/2019
2019G088	1/7/2019
2019G089	9/5/2019

2019G089	11/6/2019
2019G090	9/5/2019
2019G090	22/5/2019
2019G090	2/6/2019
2019G090	13/6/2019
2019G090	24/6/2019
2019G090	4/7/2019
2019G090	14/7/2019
2019G090	27/7/2019
2019G090	3/8/2019
2019G090	16/8/2019
2019G091	9/5/2019
2019G092	9/5/2019
2019G092	20/5/2019
2019G092	30/5/2019
2019G092	8/6/2019
2019G092	19/6/2019
2019G093	9/5/2019
2019G093	20/5/2019
2019G093	30/5/2019
2019G093	8/6/2019
2019G093	18/6/2019
2019G094	10/5/2019
2019G094	21/5/2019
2019G094	31/5/2019
2019G094	10/6/2019
2019G094	21/6/2019
2019G095	10/5/2019
2019G095	30/5/2019
2019G095	9/6/2019
2019G095	19/6/2019
2019G095	11/7/2019
2019G096	10/5/2019
2019G098	10/5/2019
2019G098	22/5/2019
2019G098	3/6/2019
2019G098	14/6/2019
2019G098	26/6/2019
2019G098	6/7/2019
2019G098	16/7/2019
2019G098	26/7/2019
2019G098	6/8/2019
2019G099	10/5/2019
2019G099	19/5/2019
2019G099	7/6/2019
2019G099	17/6/2019

2019G099	27/6/2019
2019G100	10/5/2019
2019G100	4/6/2019
2019G100	17/6/2019
2019G100	30/6/2019
2019G101	11/5/2019
2019G101	23/5/2019
2019G101	2/6/2019
2019G101	12/6/2019
2019G101	22/6/2019
2019G101	4/7/2019
2019G101	17/7/2019
2019G102	11/5/2019
2019G102	23/5/2019
2019G102	14/6/2019
2019G103	11/5/2019
2019G103	2/6/2019
2019G104	11/5/2019
2019G104	2/6/2019
2019G104	13/6/2019
2019G105	12/5/2019
2019G105	23/5/2019
2019G105	2/6/2019
2019G105	12/6/2019
2019G105	22/6/2019
2019G105	2/7/2019
2019G106	12/5/2019
2019G107	12/5/2019
2019G108	13/5/2019
2019G108	24/5/2019
2019G108	3/6/2019
2019G108	14/6/2019
2019G108	26/6/2019
2019G108	8/7/2019
2019G108	16/7/2019
2019G109	14/5/2019
2019G110	16/5/2019
2019G111	17/5/2019
2019G111	27/5/2019
2019G111	6/6/2019
2019G112	17/5/2019
2019G112	27/5/2019
2019G112	14/6/2019
2019G112	23/6/2019
2019G112	4/7/2019
2019G113	17/5/2019

2019G113	28/5/2019
2019G113	7/6/2019
2019G113	16/6/2019
2019G113	25/6/2019
2019G113	5/7/2019
2019G114	17/5/2019
2019G114	28/5/2019
2019G114	7/6/2019
2019G114	10/6/2019
2019G114	17/6/2019
2019G114	26/6/2019
2019G114	27/6/2019
2019G114	5/7/2019
2019G114	8/7/2019
2019G114	24/7/2019
2019G114	2/8/2019
2019G114	12/8/2019
2019G115	17/5/2019
2019G115	28/5/2019
2019G115	2/6/2019
2019G115	12/6/2019
2019G115	24/6/2019
2019G115	7/7/2019
2019G115	16/7/2019
2019G115	29/7/2019
2019G117	18/5/2019
2019G117	28/5/2019
2019G117	8/6/2019
2019G117	19/6/2019
2019G118	18/5/2019
2019G118	29/5/2019
2019G118	21/6/2019
2019G118	30/6/2019
2019G118	1/7/2019
2019G118	23/7/2019
2019G119	19/5/2019
2019G119	28/5/2019
2019G119	6/6/2019
2019G119	15/6/2019
2019G119	17/7/2019
2019G120	19/5/2019
2019G120	11/6/2019
2019G121	21/5/2019
2019G121	4/6/2019
2019G121	14/6/2019
2019G121	24/6/2019

2019G121	4/7/2019
2019G121	15/7/2019
2019G121	26/7/2019
2019G121	5/8/2019
2019G122	22/5/2019
2019G122	28/5/2019
2019G122	1/6/2019
2019G122	23/6/2019
2019G123	22/5/2019
2019G123	2/6/2019
2019G123	15/6/2019
2019G123	31/7/2019
2019G123	13/8/2019
2019G124	22/5/2019
2019G125	23/5/2019
2019G125	2/6/2019
2019G125	13/6/2019
2019G125	16/7/2019
2019G126	24/5/2019
2019G127	24/5/2019
2019G128	24/5/2019
2019G128	13/6/2019
2019G128	23/6/2019
2019G128	8/7/2019
2019G128	13/7/2019
2019G128	23/7/2019
2019G128	3/8/2019
2019G129	24/5/2019
2019G129	4/6/2019
2019G129	15/6/2019
2019G129	26/6/2019
2019G129	18/7/2019
2019G129	29/7/2019
2019G129	19/8/2019
2019G129	30/8/2019
2019G130	25/5/2019
2019G130	7/6/2019
2019G130	17/6/2019
2019G130	18/7/2019
2019G131	25/5/2019
2019G131	4/6/2019
2019G131	23/6/2019
2019G131	27/6/2019
2019G131	2/7/2019
2019G131	12/7/2019
2019G131	22/7/2019

2019G131	14/8/2019
2019G132	25/5/2019
2019G133	26/5/2019
2019G133	7/6/2019
2019G134	26/5/2019
2019G134	7/7/2019
2019G135	26/5/2019
2019G136	26/5/2019
2019G136	18/6/2019
2019G136	28/6/2019
2019G136	7/7/2019
2019G136	25/7/2019
2019G136	4/8/2019
2019G137	28/5/2019
2019G138	28/5/2019
2019G138	20/6/2019
2019G138	30/6/2019
2019G138	10/7/2019
2019G138	19/7/2019
2019G138	30/7/2019
2019G139	29/5/2019
2019G139	7/6/2019
2019G139	17/6/2019
2019G139	17/6/2019
2019G139	27/6/2019
2019G140	29/5/2019
2019G140	10/6/2019
2019G140	21/6/2019
2019G141	30/5/2019
2019G141	24/6/2019
2019G141	4/7/2019
2019G141	14/7/2019
2019G141	6/8/2019
2019G142	31/5/2019
2019G142	25/6/2019
2019G142	8/7/2019
2019G143	31/5/2019
2019G143	9/6/2019
2019G143	18/6/2019
2019G143	28/6/2019
2019G143	9/7/2019
2019G144	31/5/2019
2019G144	11/6/2019
2019G144	23/6/2019
2019G144	4/7/2019
2019G144	17/7/2019

2019G145	31/5/2019
2019G145	11/6/2019
2019G145	21/6/2019
2019G145	1/7/2019
2019G145	10/7/2019
2019G145	19/7/2019
2019G146	1/6/2019
2019G147	1/6/2019
2019G147	21/6/2019
2019G147	4/7/2019
2019G148	1/6/2019
2019G148	11/6/2019
2019G148	21/6/2019
2019G148	2/7/2019
2019G148	6/8/2019
2019G149	2/6/2019
2019G149	12/6/2019
2019G149	21/6/2019
2019G149	30/6/2019
2019G149	9/7/2019
2019G149	18/7/2019
2019G149	29/7/2019
2019G149	16/8/2019
2019G150	2/6/2019
2019G150	21/6/2019
2019G150	9/7/2019
2019G150	31/7/2019
2019G150	12/8/2019
2019G151	2/6/2019
2019G151	12/6/2019
2019G151	12/6/2019
2019G151	1/7/2019
2019G151	11/7/2019
2019G153	3/6/2019
2019G154	3/6/2019
2019G154	15/6/2019
2019G154	27/6/2019
2019G154	8/7/2019
2019G154	18/7/2019
2019G154	28/7/2019
2019G155	3/6/2019
2019G155	22/6/2019
2019G156	4/6/2019
2019G156	6/6/2019
2019G156	14/6/2019
2019G156	5/7/2019

2019G156	14/7/2019
2019G156	24/7/2019
2019G156	3/8/2019
2019G156	15/8/2019
2019G157	5/6/2019
2019G157	5/6/2019
2019G157	14/6/2019
2019G158	19/6/2019
2019G159	17/6/2019
2019G159	27/6/2019
2019G159	7/7/2019
2019G159	17/7/2019
2019G160	7/6/2019
2019G161	7/6/2019
2019G161	17/6/2019
2019G161	6/7/2019
2019G161	16/7/2019
2019G161	28/7/2019
2019G162	8/6/2019
2019G162	18/6/2019
2019G162	10/7/2019
2019G162	20/7/2019
2019G163	8/6/2019
2019G164	8/6/2019
2019G164	19/6/2019
2019G164	28/6/2019
2019G164	7/7/2019
2019G164	16/7/2019
2019G164	26/7/2019
2019G165	9/6/2019
2019G165	21/6/2019
2019G166	9/6/2019
2019G166	2/7/2019
2019G167	9/6/2019
2019G167	19/6/2019
2019G167	28/6/2019
2019G167	7/7/2019
2019G167	16/7/2019
2019G167	25/7/2019
2019G167	3/8/2019
2019G167	13/8/2019
2019G168	10/6/2019
2019G169	10/6/2019
2019G169	20/6/2019
2019G169	2/7/2019
2019G170	10/6/2019

2019G170	20/6/2019
2019G170	30/6/2019
2019G170	28/7/2019
2019G170	7/8/2019
2019G171	11/6/2019
2019G171	22/6/2019
2019G171	2/7/2019
2019G172	11/6/2019
2019G173	11/6/2019
2019G173	21/6/2019
2019G173	1/7/2019
2019G173	12/7/2019
2019G173	22/7/2019
2019G173	20/8/2019
2019G174	11/6/2019
2019G174	21/6/2019
2019G174	1/7/2019
2019G174	11/7/2019
2019G175	12/6/2019
2019G176	14/6/2019
2019G176	25/6/2019
2019G176	14/7/2019
2019G176	24/7/2019
2019G176	3/8/2019
2019G177	15/6/2019
2019G177	24/6/2019
2019G177	4/7/2019
2019G177	13/7/2019
2019G177	22/7/2019
2019G178	15/6/2019
2019G178	26/6/2019
2019G178	6/7/2019
2019G178	28/7/2019
2019G179	15/6/2019
2019G179	5/7/2019
2019G179	16/7/2019
2019G180	15/6/2019
2019G180	25/6/2019
2019G180	15/7/2019
2019G180	26/7/2019
2019G181	15/6/2019
2019G181	25/6/2019
2019G181	6/7/2019
2019G181	17/7/2019
2019G181	7/8/2019
2019G182	17/6/2019

2019G182	27/6/2019
2019G182	7/7/2019
2019G182	16/7/2019
2019G183	17/6/2019
2019G183	27/6/2019
2019G184	17/6/2019
2019G185	17/6/2019
2019G186	17/6/2019
2019G186	27/6/2019
2019G186	7/7/2019
2019G186	17/7/2019
2019G186	26/7/2019
2019G186	4/8/2019
2019G186	16/8/2019
2019G187	17/6/2019
2019G187	27/6/2019
2019G188	18/6/2019
2019G188	3/7/2019
2019G189	18/6/2019
2019G189	28/6/2019
2019G190	18/6/2019
2019G190	28/6/2019
2019G190	8/7/2019
2019G190	19/7/2019
2019G190	29/7/2019
2019G191	18/6/2019
2019G191	1/7/2019
2019G191	12/7/2019
2019G191	26/7/2019
2019G191	1/8/2019
2019G192	18/6/2019
2019G192	10/7/2019
2019G192	20/7/2019
2019G193	18/6/2019
2019G193	28/6/2019
2019G193	10/7/2019
2019G193	22/7/2019
2019G193	1/8/2019
2019G193	12/8/2019
2019G194	18/6/2019
2019G194	9/7/2019
2019G195	18/6/2019
2019G195	27/6/2019
2019G196	19/6/2019
2019G197	19/6/2019
2019G198	19/6/2019

2019G198	10/7/2019
2019G198	10/8/2019
2019G198	21/8/2019
2019G199	20/6/2019
2019G199	2/7/2019
2019G199	24/7/2019
2019G199	16/8/2019
2019G200	21/6/2019
2019G200	1/7/2019
2019G200	11/7/2019
2019G200	31/7/2019
2019G201	21/6/2019
2019G201	1/7/2019
2019G201	12/7/2019
2019G201	22/7/2019
2019G201	2/8/2019
2019G201	13/8/2019
2019G202	21/6/2019
2019G202	4/7/2019
2019G202	16/7/2019
2019G202	26/7/2019
2019G202	6/8/2019
2019G203	22/6/2019
2019G204	22/6/2019
2019G204	4/7/2019
2019G204	25/7/2019
2019G204	4/8/2019
2019G204	15/8/2019
2019G204	26/8/2019
2019G205	22/6/2019
2019G205	4/7/2019
2019G205	24/7/2019
2019G205	3/8/2019
2019G205	14/8/2019
2019G205	23/8/2019
2019G206	23/6/2019
2019G207	23/6/2019
2019G207	5/7/2019
2019G208	23/6/2019
2019G209	23/6/2019
2019G209	4/7/2019
2019G209	17/7/2019
2019G209	31/7/2019
2019G210	24/6/2019
2019G210	5/7/2019
2019G210	16/7/2019

2019G210	26/7/2019
2019G210	5/8/2019
2019G211	24/6/2019
2019G211	5/7/2019
2019G211	15/7/2019
2019G211	25/7/2019
2019G211	4/8/2019
2019G211	16/8/2019
2019G212	25/6/2019
2019G212	4/7/2019
2019G212	14/7/2019
2019G212	3/8/2019
2019G212	13/8/2019
2019G212	23/8/2019
2019G212	5/9/2019
2019G213	25/6/2019
2019G213	7/7/2019
2019G213	18/7/2019
2019G213	29/7/2019
2019G213	9/8/2019
2019G214	26/6/2019
2019G214	7/7/2019
2019G214	19/7/2019
2019G215	27/6/2019
2019G215	6/7/2019
2019G215	15/7/2019
2019G215	2/8/2019
2019G216	27/6/2019
2019G217	30/6/2019
2019G217	20/7/2019
2019G217	29/7/2019
2019G217	8/8/2019
2019G217	18/8/2019
2019G218	1/7/2019
2019G218	11/7/2019
2019G218	22/7/2019
2019G219	1/7/2019
2019G219	10/7/2019
2019G219	20/7/2019
2019G221	3/7/2019
2019G221	14/7/2019
2019G221	23/7/2019
2019G221	3/8/2019
2019G221	16/8/2019
2019G222	3/7/2019
2019G223	3/7/2019

2019G224	4/7/2019
2019G225	4/7/2019
2019G225	27/8/2019
2019G225	8/9/2019
2019G226	5/7/2019
2019G227	6/7/2019
2019G230	2/7/2019
2019G230	26/7/2019
2019G231	8/5/2019
2019G233	8/7/2019
2019G233	19/7/2019
2019G233	22/8/2019
2019G233	15/9/2019
2019G233	28/9/2019
2019G2337	14/8/2019
2019G234	8/7/2019
2019G234	19/7/2019
2019G234	31/7/2019
2019G234	10/8/2019
2019G235	8/7/2019
2019G235	19/7/2019
2019G235	29/7/2019
2019G236	11/7/2019
2019G237	9/7/2019
2019G237	18/7/2019
2019G237	27/7/2019
2019G237	5/8/2019
2019G237	23/8/2019
2019G238	9/7/2019
2019G239	10/7/2019
2019G239	22/8/2019
2019G240	10/7/2019
2019G240	22/7/2019
2019G240	2/8/2019
2019G240	14/8/2019
2019G240	6/9/2019
2019G240	1/10/2019
2019G241	10/7/2019
2019G241	22/7/2019
2019G241	14/8/2019
2019G242	12/7/2019
2019G242	3/8/2019
2019G243	12/7/2019
2019G244	12/7/2019
2019G244	22/7/2019
2019G244	3/8/2019

2019G244	14/8/2019
2019G245	15/7/2019
2019G245	26/7/2019
2019G245	7/8/2019
2019G245	19/8/2019
2019G246	15/7/2019
2019G247	16/7/2019
2019G247	30/7/2019
2019G247	12/8/2019
2019G247	24/8/2019
2019G247	5/9/2019
2019G247	17/9/2019
2019G247	29/9/2019
2019G248	16/7/2019
2019G248	8/8/2019
2019G248	21/8/2019
2019G249	16/7/2019
2019G250	17/7/2019
2019G250	29/7/2019
2019G250	8/8/2019
2019G250	18/8/2019
2019G250	28/8/2019
2019G250	7/9/2019
2019G250	17/9/2019
2019G251	18/7/2019
2019G251	1/8/2019
2019G251	22/8/2019
2019G253	23/7/2019
2019G253	16/8/2019
2019G253	27/8/2019
2019G254	27/7/2019
2019G254	9/8/2019
2019G254	16/8/2019
2019G254	26/8/2019
2019G254	17/9/2019
2019G255	29/7/2019
2019G256	29/7/2019
2019G257	19/7/2019
2019G257	29/7/2019
2019G257	3/8/2019
2019G257	12/8/2019
2019G257	14/8/2019
2019G258	4/8/2019
2019G258	19/8/2019
2019G258	30/8/2019
2019G259	4/8/2019

2019G259	14/8/2019
2019G260	5/8/2019
2019G260	16/8/2019
2019G261	9/8/2019
2019G261	30/8/2019
2019G261	10/9/2019
2019G262	12/8/2019
2019G263	13/8/2019
2019G263	23/8/2019
2019G263	3/9/2019
2019G263	14/9/2019
2019G263	25/9/2019
2019G264	13/8/2019
2019G264	23/8/2019
2019G264	3/9/2019
2019G264	14/9/2019
2019G265	15/8/2019
2019G265	26/8/2019
2019G266	21/8/2019
2019G266	31/8/2019
2019G266	21/9/2019
2019G267	26/8/2019
2019G267	3/9/2019
2019G267	6/9/2019
2019G268	31/7/2019
2019G268	3/8/2019
2019G269	2/8/2019
2019G270	9/8/2019
2019G270	21/8/2019
2019G271	22/8/2019
2019G271	1/9/2019
2019G272	24/8/2019
2019G272	6/10/2019
2019G272	4/9/2019
2019G272	18/10/2019
2019G272	17/11/2019
2019G273	5/9/2019
2019G274	18/9/2019
2019G275	12/9/2019
2019G276	15/9/2019
2019G277	6/9/2019
2019G277	20/9/2019
2019G278	20/9/2019
2019G279	4/10/2019
2019G280	17/8/2019
2019G280	8/9/2019

2019G280	20/9/2019
2019G282	14/8/2019
2019G282	23/8/2019
2019G282	2/9/2019
2019G282	12/9/2019
2019G283	24/8/2019
2019G283	13/9/2019
2019G283	24/9/2019
2019G284	13/9/2019
2019G285	24/8/2019
2019G287	17/5/2019
2019G288	2/6/2019
2019G289	30/6/2019
2019G290	2/7/2019
2019G291	2/7/2019
2019G292	5/7/2019
2019G293	19/7/2019
2019G293	29/7/2019
2019G293	3/9/2019
2019G295	1/8/2019
2019G296	4/8/2019
2019G296	4/9/2019
2019G297	7/8/2019
2019G297	19/8/2019
2019G298	9/8/2019
2019G299	12/8/2019
2019G300	13/8/2019
2019G301	13/8/2019
2019G301	23/8/2019
2019G302	26/8/2019
2019G302	6/9/2019
2019G303	31/8/2019
2019G304	8/9/2019
2019G305	11/9/2019
2019G305	23/9/2019
2019G305	3/10/2019
2019G305	24/10/2019
2019G306	11/9/2019
2019G307	16/9/2019
2019G308	18/9/2019
2019G309	18/9/2019
2019G309	30/9/2019
2019G309	11/10/2019
2019G309	21/10/2019
2019G310	25/9/2019
2019G310	19/10/2019

2019G311	27/9/2019
2019G311	7/10/2019
2019G312	8/10/2019
2019G312	31/10/2019
2019G313	19/10/2019
2019G314	19/10/2019
2019G315	1/9/2019
2020G014, 2[illegible]	10/5/2019
2021G100, 2[illegible]	30/6/2019
1996G009	6/5/2020
1996G009	15/5/2020
1996G009	26/5/2020
1996G009	4/6/2020
1996G009	13/6/2020
1996G009	23/6/2020
1996G009	3/7/2020
1996G009	21/7/2020
1997G018	13/5/2020
1998G030	9/6/2020
1998G030	20/7/2020
1998G030	31/7/2020
1998G030	21/8/2020
1999G024	15/3/2020
1999G024	27/3/2020
1999G024	16/4/2020
1999G024	26/4/2020
1999G024	16/5/2020
1999G024	25/5/2020
2002G052	18/5/2020
2006G012	26/3/2020
2006G012	7/4/2020
2006G012	17/4/2020
2006G012	29/4/2020
2006G012	10/5/2020
2006G012	21/5/2020
2008G002	23/3/2020
2008G002	25/4/2020
2008G002	17/5/2020
2008G006	5/5/2020
2008G006	15/5/2020
2008G006	25/5/2020
2008G006	23/6/2020
2008G006	13/7/2020
2008G021	13/3/2020
2008G021	25/3/2020
2008G021	19/4/2020

2008G021	2/5/2020
2008G050	26/8/2020
2008G050	5/9/2020
2008G050	17/9/2020
2008G050	27/9/2020
2008G050	9/10/2020
2009G001	24/4/2020
2009G001	6/5/2020
2009G001	16/5/2020
2009G001	8/6/2020
2009G001	7/7/2020
2009G006	3/3/2020
2009G006	14/3/2020
2009G006	25/3/2020
2009G006	4/4/2020
2009G006	13/4/2020
2009G006	22/4/2020
2009G006	2/5/2020
2009G006	11/5/2020
2009G037	7/6/2020
2009G037	18/6/2020
2009G037	8/7/2020
2009G037	18/7/2020
2009G037	28/7/2020
2009G037	7/8/2020
2009G037	17/8/2020
2009G037	8/9/2020
2010G015	24/3/2020
2010G015	6/4/2020
2010G015	16/4/2020
2010G015	27/4/2020
2010G015	8/5/2020
2010G015	8/6/2020
2010G033	23/6/2020
2010G040	15/2/2020
2010G040	27/2/2020
2010G040	19/3/2020
2010G040	29/3/2020
2010G040	8/4/2020
2010G040	18/4/2020
2010G060	4/5/2020
2010G060	14/5/2020
2010G060	26/5/2020
2010G060	13/6/2020
2010G060	21/6/2020
2010G060	29/6/2020

2010G060	17/7/2020
2010G100	12/6/2020
2010G100	21/6/2020
2010G100	11/7/2020
2010G100	21/7/2020
2010G100	30/7/2020
2010G100	9/8/2020
2010G100	30/8/2020
2010G107	9/9/2020
2010G107	11/10/2020
2010G107	30/10/2020
2011G025	11/4/2020
2011G035	3/3/2020
2011G035	13/3/2020
2011G035	21/4/2020
2011G100	10/8/2020
2012G003	14/2/2020
2012G003	10/3/2020
2012G003	22/3/2020
2012G029	6/3/2020
2012G029	16/3/2020
2012G029	26/3/2020
2012G029	4/4/2020
2012G029	22/4/2020
2012G029	2/5/2020
2012G086	5/8/2020
2012G088	16/7/2020
2012G088	27/7/2020
2012G088	5/8/2020
2012G088	16/8/2020
2012G088	25/8/2020
2012G088	3/9/2020
2012G088	11/9/2020
2012G088	21/9/2020
2012G088	11/10/2020
2012G091	27/5/2020
2012G091	6/6/2020
2012G091	7/7/2020
2012G091	19/7/2020
2012G093	7/5/2020
2012G093	29/5/2020
2012G093	18/6/2020
2012G093	30/6/2020
2012G093	11/7/2020
2012G093	23/7/2020
2013G024	12/6/2020

2013G024	24/6/2020
2013G024	5/7/2020
2013G024	27/7/2020
2013G024	21/8/2020
2013G028	13/2/2020
2013G031	24/3/2020
2013G031	4/4/2020
2013G031	15/4/2020
2013G031	24/4/2020
2013G031	4/5/2020
2013G031	13/5/2020
2013G036	17/4/2020
2013G036	9/5/2020
2013G036	18/5/2020
2013G036	27/5/2020
2013G036	14/6/2020
2013G038	19/4/2020
2013G038	11/5/2020
2013G038	21/5/2020
2013G038	1/6/2020
2013G038	28/6/2020
2013G038	8/7/2020
2013G038	21/7/2020
2013G054	1/5/2020
2013G054	11/5/2020
2013G054	21/5/2020
2013G054	28/6/2020
2013G054	8/7/2020
2013G054	17/7/2020
2013G063	4/5/2020
2013G063	27/5/2020
2013G063	8/6/2020
2013G063	30/6/2020
2013G063	23/7/2020
2013G071	20/5/2020
2014G015	9/4/2020
2014G015	30/4/2020
2014G015	11/5/2020
2014G015	2/6/2020
2014G040	26/4/2020
2014G040	7/5/2020
2014G041	18/3/2020
2014G041	28/3/2020
2014G041	8/4/2020
2014G041	18/4/2020
2014G041	16/5/2020

2014G041	17/5/2020
2014G041	26/5/2020
2014G057	25/9/2020
2014G057	27/9/2020
2014G063	13/4/2020
2014G063	23/4/2020
2014G063	5/5/2020
2014G069	28/5/2020
2014G069	8/6/2020
2014G069	29/6/2020
2014G069	10/7/2020
2014G069	2/8/2020
2014G069	16/8/2020
2014G089	24/9/2020
2014G096	2/5/2020
2014G096	13/5/2020
2014G101	22/7/2020
2014G101	1/8/2020
2014G107	20/7/2020
2014G107	31/7/2020
2014G107	11/8/2020
2014G107	22/8/2020
2014G107	2/9/2020
2015G002	9/6/2020
2015G013	6/5/2020
2015G019	16/4/2020
2015G019	26/4/2020
2015G025	13/6/2020
2015G025	6/7/2020
2015G025	18/8/2020
2015G034	28/5/2020
2015G034	8/6/2020
2015G034	18/6/2020
2015G034	29/6/2020
2015G034	11/7/2020
2015G049	23/8/2020
2015G066	22/7/2020
2015G066	2/8/2020
2015G066	13/8/2020
2015G066	23/8/2020
2015G066	12/9/2020
2015G066	22/9/2020
2015G073	28/6/2020
2015G073	19/7/2020
2015G073	30/7/2020
2015G092	14/7/2020

2015G092	25/7/2020
2015G092	4/8/2020
2015G092	15/8/2020
2015G092	25/8/2020
2015G092	4/9/2020
2015G092	14/9/2020
2015G092	26/9/2020
2015G108	24/6/2020
2015G118	27/5/2020
2015G118	8/6/2020
2015G118	6/7/2020
2015G118	16/7/2020
2015G118	26/7/2020
2015G118	8/8/2020
2015G228	21/6/2020
2016G003	25/5/2020
2016G003	6/7/2020
2016G012	5/4/2020
2016G023	11/4/2020
2016G023	21/4/2020
2016G023	2/5/2020
2016G023	12/5/2020
2016G023	21/5/2020
2016G023	8/6/2020
2016G023	18/6/2020
2016G023	28/6/2020
2016G025	6/4/2020
2016G028	23/3/2020
2016G028	26/4/2020
2016G028	12/5/2020
2016G028	26/5/2020
2016G043	20/3/2020
2016G043	9/4/2020
2016G043	19/4/2020
2016G046	27/3/2020
2016G046	8/4/2020
2016G046	19/4/2020
2016G046	11/5/2020
2016G070	5/3/2020
2016G070	10/6/2020
2016G076	13/4/2020
2016G076	23/4/2020
2016G076	23/5/2020
2016G076	3/6/2020
2016G084	5/6/2020
2016G084	25/6/2020

2016G084	4/7/2020
2016G084	14/7/2020
2016G084	24/7/2020
2016G084	2/8/2020
2016G084	13/8/2020
2016G101	1/5/2020
2016G101	12/5/2020
2016G109	27/4/2020
2016G109	8/5/2020
2016G109	20/5/2020
2016G110	12/5/2020
2016G123	17/4/2020
2016G123	7/5/2020
2016G123	16/5/2020
2016G123	26/5/2020
2016G123	22/6/2020
2016G129	24/4/2020
2016G129	4/5/2020
2016G129	14/5/2020
2016G129	2/6/2020
2016G138	5/5/2020
2016G138	18/6/2020
2016G177	31/5/2020
2016G228	3/7/2020
2016G228	13/7/2020
2016G228	27/7/2020
2016G254	27/3/2020
2016G254	25/4/2020
2016G254	4/5/2020
2016G254	24/5/2020
2016G256	14/5/2020
2016G256	27/5/2020
2016G256	11/6/2020
2016G261	30/9/2020
2016G293	29/4/2020
2016G293	10/5/2020
2016G293	21/5/2020
2016G297	16/7/2020
2016G297	26/7/2020
2016G297	5/8/2020
2016G297	16/8/2020
2016G297	26/8/2020
2016G297	6/9/2020
2016G305	7/7/2020
2016G305	11/8/2020
2016G305	22/8/2020

2016G305	1/9/2020
2016G305	11/9/2020
2016G305	4/10/2020
2016G308	13/5/2020
2016G308	12/6/2020
2016G308	4/7/2020
2016G311	18/9/2020
2016G311	28/9/2020
2016G318	10/9/2020
2016G318	21/9/2020
2016G318	1/10/2020
2016G318	11/10/2020
2017G009	22/9/2020
2017G028	8/3/2020
2017G028	19/3/2020
2017G028	9/4/2020
2017G028	17/4/2020
2017G028	6/5/2020
2017G028	25/5/2020
2017G030	18/3/2020
2017G030	30/3/2020
2017G031	24/3/2020
2017G031	14/4/2020
2017G040	16/2/2020
2017G040	28/2/2020
2017G040	8/3/2020
2017G040	28/3/2020
2017G040	1/5/2020
2017G040	11/5/2020
2017G040	22/5/2020
2017G040	11/6/2020
2017G040	22/6/2020
2017G040	5/7/2020
2017G040	15/7/2020
2017G069	9/5/2020
2017G069	1/6/2020
2017G070	7/6/2020
2017G070	17/6/2020
2017G070	27/6/2020
2017G070	6/7/2020
2017G070	17/7/2020
2017G071	8/4/2020
2017G071	16/4/2020
2017G071	25/4/2020
2017G071	14/5/2020
2017G071	1/6/2020

2017G077	24/3/2020
2017G077	3/4/2020
2017G077	3/5/2020
2017G087	18/4/2020
2017G087	27/4/2020
2017G087	7/5/2020
2017G087	16/5/2020
2017G087	25/5/2020
2017G087	8/7/2020
2017G115	18/6/2020
2017G115	27/6/2020
2017G115	16/7/2020
2017G115	25/7/2020
2017G115	12/8/2020
2017G115	22/8/2020
2017G115	1/9/2020
2017G155	26/4/2020
2017G155	10/5/2020
2017G155	3/6/2020
2017G155	24/6/2020
2017G155	6/7/2020
2017G155	17/7/2020
2017G155	28/7/2020
2017G155	8/8/2020
2018G015	28/7/2020
2018G149	13/4/2020
2019G019	6/5/2020
2019G029	22/6/2020
2019G029	24/7/2020
2019G037	7/9/2020
2020G001	11/1/2020
2020G002	12/1/2020
2020G002	23/1/2020
2020G002	4/2/2020
2020G003	24/1/2020
2020G004	24/1/2020
2020G004	7/3/2020
2020G004	17/3/2020
2020G005	26/1/2020
2020G005	21/3/2020
2020G005	10/4/2020
2020G005	30/4/2020
2020G005	17/5/2020
2020G006	3/2/2020
2020G007	4/2/2020
2020G008	16/2/2020

2020G008	27/2/2020
2020G009	2/3/2020
2020G010	6/3/2020
2020G011	9/3/2020
2020G012	9/3/2020
2020G013	9/3/2020
2020G014	11/3/2020
2020G015	12/3/2020
2020G015	24/3/2020
2020G015	13/4/2020
2020G015	22/4/2020
2020G015	2/5/2020
2020G015	11/5/2020
2020G016	13/3/2020
2020G016	24/3/2020
2020G016	23/4/2020
2020G016	4/5/2020
2020G017	13/3/2020
2020G018	15/3/2020
2020G018	26/3/2020
2020G018	16/4/2020
2020G018	26/4/2020
2020G018	6/5/2020
2020G019	16/3/2020
2020G019	25/3/2020
2020G019	4/4/2020
2020G019	13/4/2020
2020G019	2/5/2020
2020G019	11/5/2020
2020G019	21/5/2020
2020G020	18/3/2020
2020G021	21/3/2020
2020G021	14/5/2020
2020G021	4/6/2020
2020G022	21/3/2020
2020G022	12/4/2020
2020G022	22/4/2020
2020G022	1/5/2020
2020G022	12/5/2020
2020G023	22/3/2020
2020G024	22/3/2020
2020G024	1/4/2020
2020G024	11/4/2020
2020G024	21/4/2020
2020G024	12/5/2020
2020G025	22/3/2020

2020G025	3/4/2020
2020G025	14/4/2020
2020G025	24/4/2020
2020G025	4/5/2020
2020G026	23/3/2020
2020G026	4/4/2020
2020G026	14/4/2020
2020G026	24/4/2020
2020G027	24/3/2020
2020G027	13/4/2020
2020G028	24/3/2020
2020G028	4/4/2020
2020G029	24/3/2020
2020G029	3/4/2020
2020G029	12/4/2020
2020G029	13/4/2020
2020G029	22/4/2020
2020G029	12/5/2020
2020G029	21/5/2020
2020G029	31/5/2020
2020G030	24/3/2020
2020G030	14/4/2020
2020G030	23/4/2020
2020G030	12/5/2020
2020G030	10/6/2020
2020G031	25/3/2020
2020G031	5/4/2020
2020G031	16/4/2020
2020G032	26/3/2020
2020G032	6/4/2020
2020G033	26/3/2020
2020G033	6/4/2020
2020G033	25/4/2020
2020G033	6/5/2020
2020G033	15/5/2020
2020G034	27/3/2020
2020G034	6/4/2020
2020G034	16/4/2020
2020G035	28/3/2020
2020G035	18/4/2020
2020G035	29/4/2020
2020G035	9/5/2020
2020G036	28/3/2020
2020G036	12/5/2020
2020G036	23/5/2020
2020G037	28/3/2020

2020G037	1/5/2020
2020G038	29/3/2020
2020G038	8/4/2020
2020G038	29/4/2020
2020G038	10/5/2020
2020G038	9/6/2020
2020G039	30/3/2020
2020G039	10/4/2020
2020G039	20/4/2020
2020G039	24/4/2020
2020G039	2/5/2020
2020G039	5/5/2020
2020G039	11/5/2020
2020G039	21/5/2020
2020G039	10/6/2020
2020G039	21/6/2020
2020G040	1/4/2020
2020G041	1/4/2020
2020G041	23/4/2020
2020G041	25/5/2020
2020G041	4/6/2020
2020G042	1/4/2020
2020G042	11/4/2020
2020G042	1/5/2020
2020G042	11/5/2020
2020G042	1/6/2020
2020G043	2/4/2020
2020G043	24/4/2020
2020G043	3/5/2020
2020G043	2/6/2020
2020G044	3/4/2020
2020G045	4/4/2020
2020G045	16/4/2020
2020G045	14/5/2020
2020G046	6/4/2020
2020G046	16/4/2020
2020G046	16/5/2020
2020G047	6/4/2020
2020G047	8/5/2020
2020G048	7/4/2020
2020G048	16/4/2020
2020G048	26/4/2020
2020G048	2/5/2020
2020G049	8/4/2020
2020G049	28/4/2020
2020G049	9/5/2020

2020G049	9/6/2020
2020G050	8/4/2020
2020G050	1/5/2020
2020G050	24/5/2020
2020G051	8/4/2020
2020G051	9/5/2020
2020G051	2/6/2020
2020G052	9/3/2020
2020G052	9/4/2020
2020G052	8/5/2020
2020G053	17/3/2020
2020G053	1/5/2020
2020G054	28/3/2020
2020g054	9/4/2020
2020G054	20/4/2020
2020G055	29/3/2020
2020G056	29/3/2020
2020G057	2/4/2020
2020G057	13/4/2020
2020G057	24/4/2020
2020G057	5/5/2020
2020G058	4/4/2020
2020G059	6/4/2020
2020G059	9/4/2020
2020G059	14/5/2020
2020g060	9/4/2020
2020G060	19/4/2020
2020G060	29/4/2020
2020G060	21/5/2020
2020g061	9/4/2020
2020G061	18/4/2020
2020G061	8/5/2020
2020g062	9/4/2020
2020G062	3/5/2020
2020G063	9/4/2020
2020G063	23/4/2020
2020G063	29/4/2020
2020G063	9/5/2020
2020G063	13/5/2020
2020G063	20/5/2020
2020G063	31/5/2020
2020G063	12/6/2020
2020G064	9/4/2020
2020G064	20/4/2020
2020G064	1/5/2020
2020G064	11/5/2020

2020G064	21/5/2020
2020G065	9/4/2020
2020G065	24/4/2020
2020G065	22/5/2020
2020G066	10/4/2020
2020G066	1/7/2020
2020G067	11/4/2020
2020G068	13/4/2020
2020G068	22/5/2020
2020G068	25/6/2020
2020G069	13/4/2020
2020G069	23/4/2020
2020G069	12/5/2020
2020G070	14/4/2020
2020G070	24/4/2020
2020G070	25/5/2020
2020G070	14/6/2020
2020G071	12/4/2020
2020G071	19/4/2020
2020G071	3/5/2020
2020G071	13/5/2020
2020G071	24/5/2020
2020G072	19/4/2020
2020G073	19/4/2020
2020G074	19/4/2020
2020G075	17/4/2020
2020G076	17/4/2020
2020G076	11/5/2020
2020G077	17/4/2020
2020G077	27/4/2020
2020G077	8/5/2020
2020G077	18/5/2020
2020G077	28/5/2020
2020G078	17/4/2020
2020G078	5/5/2020
2020G079	18/4/2020
2020G079	27/4/2020
2020G079	8/5/2020
2020G079	17/5/2020
2020G079	16/6/2020
2020G080	18/4/2020
2020G080	2/5/2020
2020G080	12/5/2020
2020G080	12/6/2020
2020G081	20/4/2020
2020G081	10/5/2020

2020G082	20/4/2020
2020G082	3/5/2020
2020G082	14/5/2020
2020G082	20/6/2020
2020G082	15/7/2020
2020G083	22/4/2020
2020G083	3/5/2020
2020G083	13/5/2020
2020G084	23/4/2020
2020G084	4/6/2020
2020G084	24/6/2020
2020G085	23/4/2020
2020G085	5/5/2020
2020G085	15/5/2020
2020G085	24/5/2020
2020G085	12/6/2020
2020G085	23/6/2020
2020G086	23/4/2020
2020G087	24/4/2020
2020G087	5/5/2020
2020G087	5/7/2020
2020G088	24/4/2020
2020G088	31/5/2020
2020G089	24/4/2020
2020G089	16/5/2020
2020G089	26/5/2020
2020G089	15/6/2020
2020G089	25/6/2020
2020G090	25/4/2020
2020G090	7/5/2020
2020G090	19/5/2020
2020G090	5/7/2020
2020G091	25/4/2020
2020G092	27/4/2020
2020G092	11/5/2020
2020G093	28/4/2020
2020G094	29/4/2020
2020G095	30/4/2020
2020G096	3/5/2020
2020G096	13/5/2020
2020G096	22/5/2020
2020G096	1/6/2020
2020G097	1/5/2020
2020G098	5/5/2020
2020G098	26/5/2020
2020G098	18/6/2020

2020G099	6/5/2020
2020G099	8/6/2020
2020G099	19/6/2020
2020G099	30/6/2020
2020G100	9/5/2020
2020G101	10/5/2020
2020G102	10/5/2020
2020G103	5/5/2020
2020G103	15/5/2020
2020G104	15/5/2020
2020G105	15/5/2020
2020G105	26/5/2020
2020G105	7/6/2020
2020G106	21/5/2020
2020G106	28/6/2020
2020G106	17/7/2020
2020G106	7/8/2020
2020G107	21/5/2020
2020G108	21/5/2020
2020G108	1/6/2020
2020G108	12/6/2020
2020G108	25/6/2020
2020G109	22/5/2020
2020G110	22/5/2020
2020G111	23/5/2020
2020G111	2/6/2020
2020G111	12/6/2020
2020G111	24/6/2020
2020G111	5/7/2020
2020G111	14/7/2020
2020G111	26/7/2020
2020G112	26/5/2020
2020G112	6/6/2020
2020G112	14/6/2020
2020G112	24/6/2020
2020G112	4/7/2020
2020G113	27/5/2020
2020G114	27/5/2020
2020G115	30/5/2020
2020G116	12/5/2020
2020G116	1/6/2020
2020G116	22/6/2020
2020G117	2/6/2020
2020G117	12/6/2020
2020G117	3/7/2020
2020G117	13/7/2020

2020G118	4/6/2020
2020G118	14/6/2020
2020G119	7/6/2020
2020G119	11/6/2020
2020G119	7/7/2020
2020G119	18/7/2020
2020G120	8/6/2020
2020G120	7/7/2020
2020G121	8/6/2020
2020G122	8/6/2020
2020G123	9/6/2020
2020G124	12/6/2020
2020G124	16/7/2020
2020G125	14/6/2020
2020G126	16/6/2020
2020G127	21/6/2020
2020G127	2/7/2020
2020G127	13/7/2020
2020G128	21/6/2020
2020G128	8/7/2020
2020G128	30/7/2020
2020G129	21/6/2020
2020G129	5/7/2020
2020G129	11/7/2020
2020G129	22/7/2020
2020G129	1/8/2020
2020G129	11/8/2020
2020G129	1/9/2020
2020G130	21/6/2020
2020G130	2/7/2020
2020G130	13/7/2020
2020G130	23/7/2020
2020G130	2/8/2020
2020G130	21/8/2020
2020G130	1/9/2020
2020G131	21/6/2020
2020G132	22/6/2020
2020G132	4/7/2020
2020G132	16/7/2020
2020G132	26/7/2020
2020G132	8/8/2020
2020G133	22/6/2020
2020G134	22/6/2020
2020G134	5/7/2020
2020G135	22/6/2020
2020G135	22/6/2020

2020G135	3/7/2020
2020G135	5/7/2020
2020G136	22/6/2020
2020G136	5/7/2020
2020G136	13/7/2020
2020G137	22/6/2020
2020G137	3/7/2020
2020G137	14/7/2020
2020G137	25/7/2020
2020G138	23/6/2020
2020G138	7/7/2020
2020G139	23/6/2020
2020G140	23/6/2020
2020G140	13/7/2020
2020G140	24/7/2020
2020G140	3/8/2020
2020G140	14/8/2020
2020G141	23/6/2020
2020G141	25/6/2020
2020G142	23/6/2020
2020G142	4/7/2020
2020G143	23/6/2020
2020G144	23/6/2020
2020G144	4/7/2020
2020G144	5/7/2020
2020G144	25/7/2020
2020G144	4/8/2020
2020G144	14/8/2020
2020G144	24/8/2020
2020G145	23/6/2020
2020G145	4/7/2020
2020G145	5/7/2020
2020G145	14/7/2020
2020G145	24/7/2020
2020G145	4/8/2020
2020G146	23/6/2020
2020G146	5/7/2020
2020G147	23/6/2020
2020G147	4/7/2020
2020G147	15/7/2020
2020G147	28/7/2020
2020G148	24/6/2020
2020G148	4/7/2020
2020G148	14/7/2020
2020G148	24/7/2020
2020G148	4/8/2020

2020G149	24/6/2020
2020G150	24/6/2020
2020G150	4/7/2020
2020G151	24/6/2020
2020G152	24/6/2020
2020G152	5/7/2020
2020G153	24/6/2020
2020G154	24/6/2020
2020G154	6/7/2020
2020G154	16/7/2020
2020G155	25/6/2020
2020G155	25/6/2020
2020G155	6/8/2020
2020G155	16/8/2020
2020G155	27/8/2020
2020G156	25/6/2020
2020G156	6/7/2020
2020G156	16/7/2020
2020G156	27/7/2020
2020G156	7/8/2020
2020G156	18/8/2020
2020G156	29/8/2020
2020G157	25/6/2020
2020G157	6/7/2020
2020G158	6/7/2020
2020G158	17/7/2020
2020G158	30/7/2020
2020G159	25/6/2020
2020G159	14/7/2020
2020G160	26/5/2020
2020G160	6/7/2020
2020G160	19/7/2020
2020G161	24/6/2020
2020G162	26/6/2020
2020G163	27/6/2020
2020G163	7/7/2020
2020G163	26/7/2020
2020G163	6/8/2020
2020G164	27/6/2020
2020G164	8/7/2020
2020G164	18/7/2020
2020G164	27/7/2020
2020G165	27/6/2020
2020G166	27/6/2020
2020G166	9/7/2020
2020G166	20/7/2020

2020G166	31/7/2020
2020G167	27/6/2020
2020G167	7/7/2020
2020G167	17/7/2020
2020G167	26/7/2020
2020G167	5/8/2020
2020G168	27/6/2020
2020G168	19/7/2020
2020G168	11/8/2020
2020G169	27/6/2020
2020G170	27/6/2020
2020G170	8/7/2020
2020G172	27/6/2020
2020G172	8/7/2020
2020G172	1/8/2020
2020G173	27/6/2020
2020G173	7/7/2020
2020G174	27/6/2020
2020G174	8/7/2020
2020G174	18/7/2020
2020G174	30/7/2020
2020G175	28/6/2020
2020G175	19/7/2020
2020G175	30/7/2020
2020G176	28/6/2020
2020G176	7/7/2020
2020G176	26/7/2020
2020G176	24/8/2020
2020G177	28/6/2020
2020G177	8/7/2020
2020G178	28/6/2020
2020G178	8/7/2020
2020G178	18/7/2020
2020G178	28/7/2020
2020G179	28/6/2020
2020G179	10/8/2020
2020G179	23/8/2020
2020G180	28/6/2020
2020G181	28/6/2020
2020G182	28/6/2020
2020G182	8/7/2020
2020G182	18/7/2020
2020G182	28/7/2020
2020G182	21/8/2020
2020G183	28/6/2020
2020G184	28/6/2020

2020G185	28/6/2020
2020G186	28/6/2020
2020G186	20/7/2020
2020G186	30/7/2020
2020G187	29/6/2020
2020G187	10/7/2020
2020G187	22/7/2020
2020G187	3/8/2020
2020G188	29/6/2020
2020G188	13/7/2020
2020G188	27/7/2020
2020G189	29/6/2020
2020G190	29/6/2020
2020G190	9/7/2020
2020G191	29/6/2020
2020G191	24/7/2020
2020G192	29/6/2020
2020G193	29/6/2020
2020G193	20/7/2020
2020G193	30/7/2020
2020G193	10/8/2020
2020G194	29/6/2020
2020G195	29/6/2020
2020G195	22/7/2020
2020G195	1/8/2020
2020G196	29/6/2020
2020G196	11/7/2020
2020G197	30/6/2020
2020G198	30/6/2020
2020G198	11/7/2020
2020G198	21/7/2020
2020G198	30/7/2020
2020G198	9/8/2020
2020G198	31/8/2020
2020G199	30/6/2020
2020G199	10/7/2020
2020G199	1/9/2020
2020G199	10/9/2020
2020G200	30/6/2020
2020G200	10/7/2020
2020G200	20/7/2020
2020G201	1/7/2020
2020G202	1/7/2020
2020G202	11/7/2020
2020G202	21/7/2020
2020G202	30/7/2020

2020G202	8/8/2020
2020G202	19/8/2020
2020G202	28/8/2020
2020G203	2/7/2020
2020G203	12/7/2020
2020G204	3/7/2020
2020G204	13/7/2020
2020G204	22/7/2020
2020G205	3/7/2020
2020G206	3/7/2020
2020G207	3/7/2020
2020G208	3/7/2020
2020G209	4/7/2020
2020G209	14/7/2020
2020G209	24/7/2020
2020G209	3/8/2020
2020G209	14/8/2020
2020G209	25/8/2020
2020G210	4/7/2020
2020G210	16/7/2020
2020G211	5/7/2020
2020G211	17/7/2020
2020G211	28/7/2020
2020G211	7/8/2020
2020G211	17/8/2020
2020G211	27/8/2020
2020G212	5/7/2020
2020G213	5/7/2020
2020G214	5/7/2020
2020G215	5/7/2020
2020G215	19/7/2020
2020G216	5/7/2020
2020G216	14/7/2020
2020G216	24/7/2020
2020G216	3/8/2020
2020G217	5/7/2020
2020G217	14/7/2020
2020G218	5/7/2020
2020G218	6/7/2020
2020G218	15/7/2020
2020G219	15/7/2020
2020G219	25/7/2020
2020G220	6/7/2020
2020G220	18/7/2020
2020G221	6/7/2020
2020G221	26/7/2020

2020G221	5/8/2020
2020G222	6/7/2020
2020G222	17/7/2020
2020G223	6/7/2020
2020G223	9/8/2020
2020G224	7/7/2020
2020G225	10/7/2020
2020G225	21/7/2020
2020G225	31/7/2020
2020G226	11/7/2020
2020G226	21/7/2020
2020G226	10/8/2020
2020G227	14/7/2020
2020G228	15/7/2020
2020G228	25/7/2020
2020G229	16/7/2020
2020G229	26/7/2020
2020G230	17/7/2020
2020G231	20/7/2020
2020G231	31/7/2020
2020G231	13/8/2020
2020G232	20/7/2020
2020G232	1/8/2020
2020G232	13/8/2020
2020G232	23/8/2020
2020G232	4/9/2020
2020G233	21/7/2020
2020G234	23/7/2020
2020G235	13/7/2020
2020G235	24/7/2020
2020G235	3/8/2020
2020G235	15/8/2020
2020G235	25/8/2020
2020G235	5/9/2020
2020G236	28/7/2020
2020G237	28/7/2020
2020G237	8/8/2020
2020G237	29/8/2020
2020G238	1/9/2020
2020G239	5/8/2020
2020G239	17/8/2020
2020G240	5/8/2020
2020G242	8/8/2020
2020G243	19/8/2020
2020G244	6/8/2020
2020G244	22/8/2020

2020G245	29/8/2020
2020G245	9/9/2020
2020G245	18/9/2020
2020G246	29/8/2020
2020G246	8/9/2020
2020G246	18/9/2020
2020G246	28/9/2020
2020G247	29/8/2020
2020G248	29/8/2020
2020G249	30/8/2020
2020G249	9/9/2020
2020G249	18/9/2020
2020G250	1/9/2020
2020G250	22/9/2020
2020G250	2/10/2020
2020G251	2/9/2020
2020G251	12/9/2020
2020G252	2/9/2020
2020G253	2/9/2020
2020G253	11/9/2020
2020G253	5/10/2020
2020G254	2/9/2020
2020G255	2/9/2020
2020G256	2/9/2020
2020G257	3/9/2020
2020G258	3/9/2020
2020G258	11/9/2020
2020G258	20/9/2020
2020G259	3/9/2020
2020G259	14/9/2020
2020G260	3/9/2020
2020G260	13/9/2020
2020G261	3/9/2020
2020G262	4/9/2020
2020G262	14/9/2020
2020G263	4/9/2020
2020G263	13/9/2020
2020G263	4/10/2020
2020G263	14/10/2020
2020G263	4/11/2020
2020G263	14/11/2020
2020G264	4/9/2020
2020G264	23/9/2020
2020G265	5/9/2020
2020G266	6/9/2020
2020G266	27/9/2020

2020G266	17/10/2020
2020G266	29/10/2020
2020G267	7/9/2020
2020G267	26/9/2020
2020G268	7/9/2020
2020G268	17/9/2020
2020G269	7/9/2020
2020G269	17/9/2020
2020G270	8/9/2020
2020G271	29/9/2020
2020G272	30/9/2020
2020G273	9/9/2020
2020G273	18/9/2020
2020G273	19/9/2020
2020G273	30/9/2020
2020G274	11/9/2020
2020G274	23/9/2020
2020G274	2/10/2020
2020G275	11/9/2020
2020G276	11/9/2020
2020G277	12/9/2020
2020G278	14/9/2020
2021G051	29/9/2020
2021G096	20/9/2020
2021G096	30/9/2020
2021G096	8/10/2020
2077G081	22/3/2020
3030G175	8/7/2020
2021G001	20/1/2021
2021G002	22/1/2021
2021G003	24/1/2021
2021G004	8/2/2021
2021G005	9/2/2021
2021G006	14/2/2021
2021G007	15/2/2021
2021G003	17/2/2021
2006G003	22/2/2021
2021G008	24/2/2021
2021G009	25/2/2021
2021G003	28/2/2021
2021G010	28/2/2021
2021G011	2/3/2021
2021G012	3/3/2021
2021G013	3/3/2021
2017G111	6/3/2021
2021G014	7/3/2021

2010G014	8/3/2021
2021G009	9/3/2021
2021G015	9/3/2021
2021G003	11/3/2021
2015G131	16/3/2021
2021G016	16/3/2021
2017G024	16/3/2021
2017G111	17/3/2021
2021G014	18/3/2021
2021G028	19/3/2021
2021G017	19/3/2021
2010G074	19/3/2021
2014G093	20/3/2021
2021G018	20/3/2021
2017G101	20/3/2021
2021G019	21/3/2021
2021G020	21/3/2021
2021G021	21/3/2021
2021G023	22/3/2021
2021G003	22/3/2021
2021G010	22/3/2021
2009G014	25/3/2021
2016G224	26/3/2021
2017G024	28/3/2021
2017G108	28/3/2021
2021G024	29/3/2021
2021G025	29/3/2021
2021G017	29/3/2021
2021G014	30/3/2021
2021G026	30/3/2021
2013G030	30/3/2021
2010G014	31/3/2021
2021G027	31/3/2021
2021G009	31/3/2021
2021G028	31/3/2021
2021G029	31/3/2021
2021G030	1/4/2021
2021G019	1/4/2021
2012G068	1/4/2021
2021G023	2/4/2021
2016G067	2/4/2021
2016G131	2/4/2021
2021G073	2/4/2021
2021G032	2/4/2021
2021G033	3/4/2021
2021G034	3/4/2021

2021G035	3/4/2021
2021G010	3/4/2021
2021G003	3/4/2021
2021G036	3/4/2021
2021G042	4/4/2021
2018G028	4/4/2021
2021G044	4/4/2021
2021G037	5/4/2021
2016G095	5/4/2021
2018G021	5/4/2021
2021G040	5/4/2021
2016G224	5/4/2021
2021G016	6/4/2021
2011G082	6/4/2021
2017G111	6/4/2021
2015G021	7/4/2021
2017G024	7/4/2021
2021G045	7/4/2021
2019G119	7/4/2021
2021G039	7/4/2021
2021G041	8/4/2021
2021G043	8/4/2021
2021G046	8/4/2021
2021G047	8/4/2021
2013G056	8/4/2021
2021G048	8/4/2021
2021G049	9/4/2021
2021G050	9/4/2021
2021G051	9/4/2021
2018G069	9/4/2021
2021G007	9/4/2021
2013G030	9/4/2021
2021G052	9/4/2021
2021G024	9/4/2021
2021G027	9/4/2021
2021G045	9/4/2021
2021G009	10/4/2021
2021G053	11/4/2021
2021G054	11/4/2021
2021G055	11/4/2021
2021G056	11/4/2021
2021G019	11/4/2021
2020G200	12/4/2021
2021G057	12/4/2021
2016G059	12/4/2021
2016G039	12/4/2021

2016G067	12/4/2021
2016G131	12/4/2021
2021G058	13/4/2021
2021G035	13/4/2021
2021G032	13/4/2021
2021G033	13/4/2021
2021G059	13/4/2021
2021G003	13/4/2021
2021G044	14/4/2021
2021G060	14/4/2021
2021G036	14/4/2021
2021G042	14/4/2021
2016G095	15/4/2021
2018G028	15/4/2021
2021G034	15/4/2021
2021G061	15/4/2021
2018G021	15/4/2021
2016G224	15/4/2021
2021G062	15/4/2021
2021G016	15/4/2021
2021G067	16/4/2021
2017G111	16/4/2021
2021G040	16/4/2021
2021G022	16/4/2021
2021G063	16/4/2021
2016G180	17/4/2021
2021G064	17/4/2021
2021G065	17/4/2021
2011G082	17/4/2021
2021G039	17/4/2021
2018G017	17/4/2021
2017G024	17/4/2021
2021G046	18/4/2021
2021G007	18/4/2021
2021G048	18/4/2021
2021G017	18/4/2021
2021G027	19/4/2021
2021G047	19/4/2021
2010G014	19/4/2021
2018G026	19/4/2021
2021G024	19/4/2021
2021G041	19/4/2021
2021G050	20/4/2021
2013G030	20/4/2021
2018G069	20/4/2021
2021G049	20/4/2021

2016G130	20/4/2021
2017G023	20/4/2021
2021G052	20/4/2021
2021G066	21/4/2021
2017G019	21/4/2021
2012G068	21/4/2021
2021G053	21/4/2021
2014G079	21/4/2021
2021G054	21/4/2021
2018G035	21/4/2021
2021G019	22/4/2021
2021G057	22/4/2021
2016G059	22/4/2021
2016G131	22/4/2021
2021G023	24/4/2021
2021G058	24/4/2021
2012G038	24/4/2021
2021G042	24/4/2021
2016G204	24/4/2021
2021G044	24/4/2021
2021G016	24/4/2021
2016G224	25/4/2021
2018G021	25/4/2021
2021G059	25/4/2021
2018G017	25/4/2021
2016G095	25/4/2021
2018G028	25/4/2021
2015G131	26/4/2021
2017G111	26/4/2021
2021G067	26/4/2021
2021G062	26/4/2021
2021G036	26/4/2021
2011G082	26/4/2021
2016G071	27/4/2021
2016G180	27/4/2021
2021G061	27/4/2021
2015G075	27/4/2021
2006G003	27/4/2021
2021G065	27/4/2021
2017G024	27/4/2021
2021G040	28/4/2021
2021G070	28/4/2021
2021G022	28/4/2021
2021G069	28/4/2021
2014G079	28/4/2021
2021G068	28/4/2021

2015G021	29/4/2021
2021G063	29/4/2021
2018G069	29/4/2021
2021G024	29/4/2021
2014G099	29/4/2021
2015G018	29/4/2021
2021G071	30/4/2021
2016G025	30/4/2021
2018G026	30/4/2021
2017G078	30/4/2021
2021G049	30/4/2021
2016G130	30/4/2021
2012G036	30/4/2021
2017G023	30/4/2021
2021G050	1/5/2021
2013G030	1/5/2021
2012G132	1/5/2021
2016G039	1/5/2021
2016G059	1/5/2021
2012G068	1/5/2021
2021G054	1/5/2021
2021G072	1/5/2021
2015G042	2/5/2021
2017G019	2/5/2021
2018G103	2/5/2021
2018G035	2/5/2021
2016G131	2/5/2021
2021G016	3/5/2021
2021G073	3/5/2021
2021G035	3/5/2021
2021G023	3/5/2021
2021G074	3/5/2021
2021G057	3/5/2021
2021G033	3/5/2021
2021G042	3/5/2021
2021G034	4/5/2021
2021G075	4/5/2021
2018G017	4/5/2021
2021G044	4/5/2021
2018G021	4/5/2021
2021G076	5/5/2021
2018G028	5/5/2021
2016G204	6/5/2021
2009G035	6/5/2021
2021G077	6/5/2021
2016G071	6/5/2021

2014G120	6/5/2021
2021G059	6/5/2021
2021G048	6/5/2021
2021G065	6/5/2021
2017G035	6/5/2021
2016G095	6/5/2021
2014G034	7/5/2021
2014G079	7/5/2021
2021G022	7/5/2021
2021G024	8/5/2021
2021G078	8/5/2021
2021G051	8/5/2021
2021G040	8/5/2021
2018G069	8/5/2021
2021G079	8/5/2021
2015G075	9/5/2021
2021G017	9/5/2021
2021G041	9/5/2021
2015G021	9/5/2021
2016G130	9/5/2021
2016G025	10/5/2021
2018G025	10/5/2021
2013G030	10/5/2021
2017G021	10/5/2021
2021G054	11/5/2021
2018G035	11/5/2021
2021G050	11/5/2021
2016G059	11/5/2021
2021G049	11/5/2021
2016G105	11/5/2021
2012G036	11/5/2021
2016G131	12/5/2021
2015G042	12/5/2021
2015G045	12/5/2021
2018G046	12/5/2021
2021G074	13/5/2021
2017G019	13/5/2021
2021G016	13/5/2021
2021G080	13/5/2021
2018G017	13/5/2021
2021G034	13/5/2021
2017G098	13/5/2021
2014G065	13/5/2021
2021G019	14/5/2021
2014G081	14/5/2021
2018G021	14/5/2021

2021G042	15/5/2021
2021G081	15/5/2021
2021G058	15/5/2021
2016G071	15/5/2021
2014G076	15/5/2021
2021G075	16/5/2021
2018G028	16/5/2021
2016G224	16/5/2021
2010G014	16/5/2021
2009G035	16/5/2021
2011G082	16/5/2021
2021G077	17/5/2021
2021G048	17/5/2021
2016G095	17/5/2021
2021G061	17/5/2021
2021G059	17/5/2021
2021G024	17/5/2021
2021G022	17/5/2021
2016G204	18/5/2021
2016G130	18/5/2021
2021G078	18/5/2021
2021G087	18/5/2021
2014G101	18/5/2021
2021G041	19/5/2021
2015G075	19/5/2021
2017G088	20/5/2021
2021G031	20/5/2021
2021G068	20/5/2021
2018G035	20/5/2021
2013G030	20/5/2021
2016G025	20/5/2021
2021G054	21/5/2021
2021G082	21/5/2021
2016G025	21/5/2021
2015G045	21/5/2021
2021G067	23/5/2021
2014G065	23/5/2021
2015G042	23/5/2021
2015G131	23/5/2021
2021G023	23/5/2021
2017G019	24/5/2021
2017G078	24/5/2021
2017G111	25/5/2021
2021G085	25/5/2021
2021G046	26/5/2021
2021G086	26/5/2021

2021G044	27/5/2021
2021G051	27/5/2021
2021G087	27/5/2021
2021G024	27/5/2021
2008G069	27/5/2021
2021G061	27/5/2021
2018G028	27/5/2021
2016G204	28/5/2021
2021G088	28/5/2021
2011G082	28/5/2021
2016G130	28/5/2021
2021G022	28/5/2021
2021G059	28/5/2021
2021G089	29/5/2021
2018G026	29/5/2021
2015G075	29/5/2021
2009G033	29/5/2021
2015G021	30/5/2021
2015G007	30/5/2021
2018G035	30/5/2021
2016G243	30/5/2021
2017G088	31/5/2021
2018G017	31/5/2021
2010G082	31/5/2021
2016G157	31/5/2021
2015G131	1/6/2021
2021G063	1/6/2021
2021G054	1/6/2021
2013G030	1/6/2021
2021G034	1/6/2021
2016G059	1/6/2021
2015G045	1/6/2021
2021G031	1/6/2021
2016G025	1/6/2021
2016G190	1/6/2021
2015G042	1/6/2021
2021G062	1/6/2021
2018G103	1/6/2021
2021G083	2/6/2021
2015G049	2/6/2021
2021G084	2/6/2021
2018G132	2/6/2021
2021G041	2/6/2021
2018G021	2/6/2021
2021G082	3/6/2021
1994G060	3/6/2021

2018G098	4/6/2021
2016G131	4/6/2021
2017G019	4/6/2021
2021G046	4/6/2021
2021G087	5/6/2021
2021G051	5/6/2021
2012G061	5/6/2021
2009G035	5/6/2021
2018G069	6/6/2021
MYT3122	6/6/2021
2021G061	6/6/2021
2018G028	6/6/2021
2016G204	7/6/2021
2016G130	7/6/2021
2021G090	7/6/2021
2013G162	7/6/2021
2011G082	7/6/2021
2021G059	7/6/2021
2021G089	7/6/2021
2016G095	7/6/2021
2021G091	8/6/2021
2018G035	8/6/2021
2018G026	8/6/2021
2021G034	10/6/2021
2016G157	10/6/2021
2016G059	10/6/2021
2015G045	10/6/2021
2015G001	10/6/2021
2017G023	11/6/2021
2018G103	11/6/2021
2021G092	11/6/2021
2021G093	11/6/2021
2013G030	12/6/2021
2021G063	12/6/2021
2021G031	12/6/2021
2021G046	12/6/2021
2021G057	12/6/2021
2021G054	12/6/2021
2021G132	12/6/2021
2021G081	14/6/2021
2021G042	14/6/2021
2010G099	14/6/2021
2016G039	14/6/2021
2014G068	14/6/2021
2017G019	15/6/2021
2021G058	16/6/2021

2016G004	16/6/2021
2021G051	16/6/2021
2009G035	16/6/2021
2021G094	16/6/2021
2021G085	16/6/2021
2017G078	17/6/2021
2018G074	17/6/2021
2016G095	17/6/2021
2021G061	17/6/2021
2016G204	18/6/2021
2021G089	18/6/2021
2021G059	18/6/2021
2021G095	19/6/2021
2014G109	19/6/2021
1999G015	19/6/2021
2016G211	19/6/2021
2016G130	19/6/2021
2018G035	19/6/2021
2011G058	19/6/2021
2011G086	19/6/2021
2015G075	19/6/2021
2021G097	19/6/2021
2012G061	20/6/2021
2016G157	21/6/2021
2015G042	21/6/2021
2017G023	21/6/2021
221G038	21/6/2021
2021G084	21/6/2021
2021G098	21/6/2021
2021G057	22/6/2021
2021G034	23/6/2021
2013G132	23/6/2021
2021G064	24/6/2021
2016G190	24/6/2021
2016G039	24/6/2021
2016G004	25/6/2021
2012G036	25/6/2021
2016G105	25/6/2021
2021V031	25/6/2021
2008G069	26/6/2021
2010G099	26/6/2021
2021G094	26/6/2021
2009G035	26/6/2021
2021G031	26/6/2021
2021G081	27/6/2021
1994G060	27/6/2021

2021G085	27/6/2021
2014G068	27/6/2021
1999G015	28/6/2021
2018G074	28/6/2021
2014G109	28/6/2021
2021G087	28/6/2021
2021G099	28/6/2021
2016G095	29/6/2021
2016G204	29/6/2021
2021G091	29/6/2021
2021G097	29/6/2021
2017G088	29/6/2021
2021G059	29/6/2021
2021G089	29/6/2021
2018G058	29/6/2021
2016G223	29/6/2021
2016G130	30/6/2021
2021G057	30/6/2021
2010G082	30/6/2021
2021G095	30/6/2021
2018G131	30/6/2021
2015G045	30/6/2021
2016G157	1/7/2021
2016G166	1/7/2021
2021G057	2/7/2021
2021G100	4/7/2021
2016G105	4/7/2021
2016G190	4/7/2021
2009G035	6/7/2021
2010G099	6/7/2021
2017G019	6/7/2021
2021G101	7/7/2021
2021G102	8/7/2021
2018G074	8/7/2021
2016G243	8/7/2021
1994G060	8/7/2021
2021G097	8/7/2021
2017G088	8/7/2021
2017G112	9/7/2021
2016G204	9/7/2021
2011G058	9/7/2021
2021G095	10/7/2021
2016G157	10/7/2021
2015G045	11/7/2021
2018G131	11/7/2021
2011G058	11/7/2021

2016G223	12/7/2021
2021G103	12/7/2021
2021G104	12/7/2021
2015G049	12/7/2021
2011G086	12/7/2021
2021G098	12/7/2021
2021G100	14/7/2021
2016G105	14/7/2021
2016G025	15/7/2021
2016G009	15/7/2021
2017G084	15/7/2021
2009G035	16/7/2021
2010G099	16/7/2021
2013G162	16/7/2021
2021G105	17/7/2021
2021G101	17/7/2021
2021G087	17/7/2021
2018G074	18/7/2021
2017G088	18/7/2021
2012G036	18/7/2021
2021G106	18/7/2021
2021G097	18/7/2021
2017G019	18/7/2021
2012G061	18/7/2021
2007G015	19/7/2021
2021G107	19/7/2021
2021G089	19/7/2021
1994G060	19/7/2021
2004G052	20/7/2021
2021G099	20/7/2021
2011G058	20/7/2021
2017G078	21/7/2021
2016G157	21/7/2021
2021G103	22/7/2021
2021G104	24/7/2021
2016G190	24/7/2021
2008G069	24/7/2021
2016G004	25/7/2021
2009G035	25/7/2021
2014G109	25/7/2021
2017G085	25/7/2021
2021G108	25/7/2021
2014G132	26/7/2021
2021G109	26/7/2021
2021G105	26/7/2021
2009G046	26/7/2021

2021G094	26/7/2021
2008G001	26/7/2021
2018G074	27/7/2021
2010G099	27/7/2021
2006G037	27/7/2021
2021G102	27/7/2021
2010G082	27/7/2021
2008G001	27/7/2021
2017G088	28/7/2021
2012G036	28/7/2021
2021G097	28/7/2021
2016G243	29/7/2021
2004G052	29/7/2021
2021G089	29/7/2021
2021G099	30/7/2021
2016G157	30/7/2021
2021G110	1/8/2021
2011G086	1/8/2021
2011G058	2/8/2021
2016G004	3/8/2021
2017G112	3/8/2021
2016G255	3/8/2021
2018G131	3/8/2021
2014G109	3/8/2021
1999G015	3/8/2021
2021G109	5/8/2021
2021G111	6/8/2021
2021G115	6/8/2021
2004G052	7/8/2021
2021G112	8/8/2021
2012G036	9/8/2021
2021G099	9/8/2021
2021G113	9/8/2021
1994G060	10/8/2021
2021G103	11/8/2021
2017G084	12/8/2021
2016G004	12/8/2021
1999G015	12/8/2021
2016G166	12/8/2021
2018G131	14/8/2021
2021G114	14/8/2021
2018G068	14/8/2021
2011G086	14/8/2021
2008G001	14/8/2021
2010G082	15/8/2021
2016G255	15/8/2021

2016G223	15/8/2021
2021G114	16/8/2021
2017G112	16/8/2021
2006G037	16/8/2021
2009G046	16/8/2021
2007G015	16/8/2021
2021G109	17/8/2021
2004G052	17/8/2021
2011G044	17/8/2021
2021G094	18/8/2021
2021G113	20/8/2021
2021G110	21/8/2021
2017G084	21/8/2021
1994G060	22/8/2021
2016G004	22/8/2021
2014G109	22/8/2021
2021G103	23/8/2021
2016G166	23/8/2021
2018G112	23/8/2021
2008G003	24/8/2021
2010G082	25/8/2021
2018G131	25/8/2021
2018G068	25/8/2021
2007G015	26/8/2021
2006G037	26/8/2021
2014G132	26/8/2021
2016G223	26/8/2021
2021G102	26/8/2021
2016G255	27/8/2021
2009G046	27/8/2021
2017G112	28/8/2021
1994G022	31/8/2021
2017G084	31/8/2021
2014G109	31/8/2021
2008G003	2/9/2021
2021G117	2/9/2021
2016G166	2/9/2021
2010G082	3/9/2021
2018G068	4/9/2021
2007G015	4/9/2021
2014G132	5/9/2021
2006G037	5/9/2021
1995G095	6/9/2021
2016G255	8/9/2021
2017G112	8/9/2021
2009G046	8/9/2021

2016G223	8/9/2021
2017G084	9/9/2021
2013G165	10/9/2021
1994G022	11/9/2021
2021G109	11/9/2021
2008G001	11/9/2021
2006G037	16/9/2021
2017G112	20/9/2021
2008G003	20/9/2021
2021G118	22/9/2021
2013G165	22/9/2021
2021G119	22/9/2021
2021G117	24/9/2021
2014G132	26/9/2021
2017G161	27/9/2021
2016G255	29/9/2021
2017G112	2/10/2021
2018G018	3/10/2021
2021G117	5/10/2021
2014G132	6/10/2021
2016G255	10/10/2021
2021G118	13/10/2021
2021G120	24/10/2021
2017G163	30/10/2021
2017G163	21/1/2022
2015G120	14/2/2022
2016G108	19/2/2022
2016G014	7/3/2022
2008G036	9/3/2022
2018G007	14/3/2022
2009G040	16/3/2022
1999G005	17/3/2022
2022G001	17/3/2022
2015G120	19/3/2022
2016G014	19/3/2022
2013G006	20/3/2022
2008G036	20/3/2022
2013G006	20/3/2022
2013G056	21/3/2022
2018G007	24/3/2022
2015G003	24/3/2022
2019G049	27/3/2022
2016G108	27/3/2022
2016G050	28/3/2022
2015G120	29/3/2022
2016G014	29/3/2022

2013G006	30/3/2022
2013G056	30/3/2022
2014G012	1/4/2022
2013G110	2/4/2022
2016G010	2/4/2022
2018G007	2/4/2022
2014G048	3/4/2022
2019G045	4/4/2022
2022G001	5/4/2022
2018G024	6/4/2022
1999G005	6/4/2022
2016G002	6/4/2022
2012G084	6/4/2022
2016G050	8/4/2022
2015G120	8/4/2022
2013G006	9/4/2022
2016G029	10/4/2022
2018G063	10/4/2022
2022G002	10/4/2022
2018G007	11/4/2022
2013G110	12/4/2022
2015G003	13/4/2022
2014G048	14/4/2022
1999G005	15/4/2022
2022G001	15/4/2022
2019G098	15/4/2022
2016G002	16/4/2022
2018G024	16/4/2022
2013G056	17/4/2022
2022G003	18/4/2022
2013G034	18/4/2022
2016G014	18/4/2022
2016G050	18/4/2022
2022G004	20/4/2022
2022G005	20/4/2022
2017G028	20/4/2022
2022G002	20/4/2022
2018G034	20/4/2022
2018G029	21/4/2022
2015G014	21/4/2022
2013G074	21/4/2022
2014G012	21/4/2022
2017G050	21/4/2022
2010G032	22/4/2022
2019G140	22/4/2022
2008G063	22/4/2022

2018GO20	22/4/2022
2022G006	22/4/2022
2013G110	22/4/2022
2014G045	23/4/2022
2022G007	23/4/2022
2016G159	23/4/2022
2022G001	24/4/2022
1999G005	24/4/2022
2019G045	25/4/2022
2019G094	25/4/2022
2009G049	25/4/2022
2019G098	26/4/2022
2022G008	26/4/2022
2013G056	26/4/2022
2018G024	26/4/2022
2010G021	26/4/2022
2016G021	28/4/2022
2016G014	28/4/2022
2022G003	28/4/2022
2012G028	28/4/2022
2012G028	29/4/2022
2016G093	29/4/2022
2022G002	29/4/2022
2017G060	29/4/2022
2018G007	29/4/2022
2010G015	30/4/2022
2022G009	30/4/2022
2013G034	30/4/2022
2017G028	30/4/2022
2019G085	30/4/2022
2019G121	1/5/2022
2019G140	1/5/2022
2013G051	2/5/2022
2022G007	2/5/2022
2013G110	2/5/2022
2016G002	2/5/2022
2013G125	2/5/2022
2013G074	2/5/2022
2019G061	2/5/2022
2014G012	2/5/2022
1999G005	3/5/2022
2015G014	3/5/2022
2018G078	3/5/2022
2014G045	3/5/2022
2018G029	3/5/2022
2010G032	3/5/2022

2015G038	3/5/2022
2022G005	3/5/2022
2015G003	4/5/2022
2022G001	4/5/2022
2009G049	4/5/2022
2008G063	4/5/2022
2018G050	5/5/2022
2013G056	5/5/2022
2013G056	5/5/2022
2002G044	5/5/2022
2014G048	5/5/2022
2019G045	5/5/2022
2022G008	5/5/2022
2016G159	5/5/2022
2012G084	5/5/2022
2022G010	6/5/2022
2018G024	6/5/2022
2010G021	7/5/2022
2019G098	7/5/2022
2022G011	7/5/2022
2016G108	7/5/2022
2022G012	7/5/2022
2016G050	8/5/2022
2018G007	8/5/2022
2013G006	9/5/2022
2017G051	9/5/2022
2017G012	9/5/2022
2017G028	9/5/2022
2022G013	10/5/2022
2012G025	10/5/2022
2018G103	10/5/2022
2019G085	10/5/2022
2010G015	11/5/2022
2013G034	11/5/2022
2017G050	11/5/2022
2015G038	13/5/2022
2018G029	13/5/2022
2014G012	13/5/2022
2010G032	13/5/2022
2015G014	13/5/2022
2010G089	13/5/2022
2014G045	13/5/2022
2018G095	13/5/2022
2015G003	13/5/2022
2013G074	13/5/2022
2018G078	13/5/2022

2018G020	13/5/2022
2017G037	14/5/2022
2012G041	14/5/2022
2017G002	14/5/2022
2013G056	14/5/2022
2014G038	14/5/2022
2008G063	15/5/2022
2009G049	15/5/2022
2019G045	15/5/2022
2022G014	15/5/2022
2018G024	15/5/2022
2022G015	16/5/2022
2022G016	16/5/2022
2019G094	16/5/2022
2022G017	16/5/2022
2022G018	17/5/2022
2016G108	17/5/2022
2022G010	17/5/2022
2019G002	17/5/2022
2018G007	17/5/2022
2012G028	17/5/2022
2016G0093	17/5/2022
2018G096	17/5/2022
2018G150	18/5/2022
2022G012	18/5/2022
2016G050	18/5/2022
2017G046	18/5/2022
2022G003	18/5/2022
2018G111	19/5/2022
2017G060	19/5/2022
2022G004	19/5/2022
2018G057	20/5/2022
2022G019	20/5/2022
2018G034	20/5/2022
2017G012	20/5/2022
2012G025	20/5/2022
2017G050	20/5/2022
2022G020	20/5/2022
2022G022	21/5/2022
2010G015	21/5/2022
2016G196	21/5/2022
2022G027	21/5/2022
2022G021	21/5/2022
2013G006	21/5/2022
2022G023	21/5/2022
2013G034	22/5/2022

2010G032	22/5/2022
2016G002	22/5/2022
2014G045	23/5/2022
2019G121	23/5/2022
2018G059	23/5/2022
2015G014	23/5/2022
2010G089	23/5/2022
2018G029	23/5/2022
2018G078	23/5/2022
2013G051	24/5/2022
2011G045	24/5/2022
2014G038	24/5/2022
2018G024	24/5/2022
2013G125	24/5/2022
2018G020	24/5/2022
2013G056	25/5/2022
2019G045	25/5/2022
2022G024	25/5/2022
2022G025	25/5/2022
2022G015	25/5/2022
2022G026	25/5/2022
2022G008	25/5/2022
2012G052	25/5/2022
2010G015	25/5/2022
2008G063	26/5/2022
2018G044	26/5/2022
2018G104	26/5/2022
2012G028	26/5/2022
2018G072	26/5/2022
2022G010	27/5/2022
2019G098	27/5/2022
2017G060	28/5/2022
2016G050	28/5/2022
2017G046	28/5/2022
2018G076	29/5/2022
2018G150	29/5/2022
2022G020	30/5/2022
2010G021	30/5/2022
2019G140	30/5/2022
1995G001	30/5/2022
2019G179	30/5/2022
2016G196	30/5/2022
2017G012	31/5/2022
2019G085	31/5/2022
2022G027	31/5/2022
2010G032	1/6/2022

2022G028	1/6/2022
2013G090	1/6/2022
2015G020	1/6/2022
2013G117	2/6/2022
2015G003	2/6/2022
2015G014	2/6/2022
2013G034	2/6/2022
2013G074	2/6/2022
2018G024	2/6/2022
2018G070	2/6/2022
2015G038	2/6/2022
2013G125	2/6/2022
2013G051	3/6/2022
2018G078	3/6/2022
2022G006	3/6/2022
2018G029	3/6/2022
2018G020	3/6/2022
2014G045	3/6/2022
2015G072	4/6/2022
2022G025	4/6/2022
2012G041	4/6/2022
2019G104	4/6/2022
2011G045	5/6/2022
2014G048	5/6/2022
2019G094	5/6/2022
2022G005	5/6/2022
2022G008	5/6/2022
2016G236	5/6/2022
2017G037	5/6/2022
2014G061	6/6/2022
2019G098	6/6/2022
2012G048	6/6/2022
2017G037	6/6/2022
2017G028	6/6/2022
2012G028	6/6/2022
2018G063	6/6/2022
2019G061	7/6/2022
2018G072	7/6/2022
2019G002	7/6/2022
2017G060	7/6/2022
2018G048	7/6/2022
2018G034	7/6/2022
2022G029	7/6/2022
2018G096	7/6/2022
2022G012	8/6/2022
2022G010	8/6/2022

2010G021	9/6/2022
2022G024	9/6/2022
2016G196	9/6/2022
2019G153	9/6/2022
2019G166	9/6/2022
2022G030	10/6/2022
2017G042	10/6/2022
2022G027	10/6/2022
2022G031	11/6/2022
2010G015	11/6/2022
2019G140	11/6/2022
2013G138	12/6/2022
2018G027	12/6/2022
2015G014	12/6/2022
2010G032	12/6/2022
2018G077	12/6/2022
2019G121	12/6/2022
2013G034	12/6/2022
2022G032	12/6/2022
2022G006	13/6/2022
2018G078	13/6/2022
2018G024	13/6/2022
2018G020	13/6/2022
2014G045	14/6/2022
2013G051	14/6/2022
2019G045	14/6/2022
2022G025	14/6/2022
2022G015	14/6/2022
2022G033	15/6/2022
2013G017	15/6/2022
2013G130	15/6/2022
2022G018	15/6/2022
2016G029	15/6/2022
2022G018	15/6/2022
2019G104	15/6/2022
2018G148	16/6/2022
2018G034	16/6/2022
2018G114	16/6/2022
2014G097	16/6/2022
2016G236	16/6/2022
2012G014	14/6/2022
2017G028	15/6/2022
2014G061	17/6/2022
2013G101	18/6/2022
2019G098	18/6/2022
2017G060	18/6/2022

2017G110	18/6/2022
2018G096	18/6/2022
2018G057	18/6/2022
2022G035	18/6/2022
2017G046	18/6/2022
2018G057	18/6/2022
2018G072	18/6/2022
2022G005	18/6/2022
2018G111	18/6/2022
2022G020	19/6/2022
2022G036	19/6/2022
2022G012	19/6/2022
2017G050	19/6/2022
2019G061	19/6/2022
2016G196	20/6/2022
2015G058	20/6/2022
2015G020	20/6/2022
2022G037	20/6/2022
2022G038	21/6/2022
2019G179	21/6/2022
2013G125	21/6/2022
2010G015	21/6/2022
2019G121	21/6/2022
2015G038	21/6/2022
2022G027	21/6/2022
2017G079	21/6/2022
2017G127	21/6/2022
2013G117	22/6/2022
2010G032	22/6/2022
2018G059	22/6/2022
2013G090	22/6/2022
2014G038	22/6/2022
2010G089	22/6/2022
2018G078	23/6/2022
2022G040	23/6/2022
2019G045	24/6/2022
2017G089	24/6/2022
2012G052	24/6/2022
2017G028	24/6/2022
2022G041	24/6/2022
2022G042	24/6/2022
2012G041	25/6/2022
2018G067	25/6/2022
2018G027	25/6/2022
2018G035	25/6/2022
2016G093	25/6/2022

2022G043	25/6/2022
2003G008	25/6/2022
2018G114	25/6/2022
2019G002	26/6/2022
2022G044	26/6/2022
2018G147	26/6/2022
2014G097	27/6/2022
2015G072	27/6/2022
2022G045	27/6/2022
2011G045	27/6/2022
2022G046	27/6/2022
2013G101	28/6/2022
2016G029	28/6/2022
2017G046	28/6/2022
2012G048	28/6/2022
2022G047	28/6/2022
2012G025	28/6/2022
2022G048	29/6/2022
2022G049	29/6/2022
2022G012	29/6/2022
2022G050	29/6/2022
2022G051	29/6/2022
2022G052	30/6/2022
2022G053	1/7/2022
2022G054	1/7/2022
2013G072	1/7/2022
2013G125	1/7/2022
2013G135	1/7/2022
2010G032	1/7/2022
2022G055	1/7/2022
2017G012	2/7/2022
2022G056	2/7/2022
2022G057	2/7/2022
2022G058	2/7/2022
2010G015	2/7/2022
2018G059	2/7/2022
2015G058	2/7/2022
2018G078	2/7/2022
2022G059	3/7/2022
6056: 2013G	3/7/2022
2022G040	3/7/2022
2013G051	4/7/2022
2017G045	4/7/2022
2019G045	4/7/2022
MYT2190:201	5/7/2022
2018G050	5/7/2022

2007G032	5/7/2022
2018G035	5/7/2022
2022G060	5/7/2022
2022G043	5/7/2022
2022G061	6/7/2022
2019G002	6/7/2022
2022G046	6/7/2022
2018G147	6/7/2022
2014G061	6/7/2022
2018G136	7/7/2022
2011G045	7/7/2022
2013G101	7/7/2022
2022G044	8/7/2022
2022G047	8/7/2022
2022G020	8/7/2022
2022G062	8/7/2022
2017G046	8/7/2022
2012G048	8/7/2022
2012G025	8/7/2022
2018G072	8/7/2022
2022G063	8/7/2022
2014G097	9/7/2022
2018G111	9/7/2022
2018G150	9/7/2022
2022G012	9/7/2022
2022G064	9/7/2022
2016G196	9/7/2022
2018G048	10/7/2022
2022G055	10/7/2022
2022G052	10/7/2022
2016G093	10/7/2022
2019G179	10/7/2022
2013G117	10/7/2022
2015G038	10/7/2022
2022G038	11/7/2022
2012G052	11/7/2022
2017G127	11/7/2022
2019G061	11/7/2022
2022G065	12/7/2022
2017G037	12/7/2022
2022G066	12/7/2022
2022G067	13/7/2022
2018G125	13/7/2022
2022G057	13/7/2022
2014G038	13/7/2022
2019G166	13/7/2022

2022G056	13/7/2022
2018G078	13/7/2022
2017G050	13/7/2022
2022G058	14/7/2022
2022G068	14/7/2022
2013G074	14/7/2022
2010G015	14/7/2022
2022G040	14/7/2022
2017G089	14/7/2022
2022G053	14/7/2022
2022G055	13/7/2022
2015G107	15/7/2022
2022G040	15/7/2022
2013G051	16/7/2022
2019G121	16/7/2022
2019G204	16/7/2022
2016G236	16/7/2022
2019G002	16/7/2022
2013G101	17/7/2022
2018G114	17/7/2022
2014G061	17/7/2022
2012G048	18/7/2022
2017G047	18/7/2022
2022G020	19/7/2022
2015G020	19/7/2022
2015G072	19/7/2022
2012G052	20/7/2022
2022G038	20/7/2022
2018G072	20/7/2022
2022G048	21/7/2022
2018G025	21/7/2022
2022G047	21/7/2022
2022G044	21/7/2022
2015G056	21/7/2022
2022G044	22/7/2022
2022G065	22/7/2022
2022G029	22/7/2022
2008G004	22/7/2022
2022G053	22/7/2022
2018G125	23/7/2022
2019G179	23/7/2022
2022G069	24/7/2022
2017G037	24/7/2022
2022G060	24/7/2022
2010G015	24/7/2022
2015G058	25/7/2022

2015G107	26/7/2022
2014G038	26/7/2022
2017G012	26/7/2022
2019G104	27/7/2022
2018G114	27/7/2022
2019G204	27/7/2022
2018G147	28/7/2022
2022G015	29/7/2022
2022G050	29/7/2022
2022G063	30/7/2022
2022G070	30/7/2022
2014G097	30/7/2022
2018G048	31/7/2022
2022G038	1/8/2022
2022G047	1/8/2022
2008G004	1/8/2022
2017G158	1/8/2022
2015G056	1/8/2022
2022G029	1/8/2022
2018G078	1/8/2022
2017G127	1/8/2022
2003G008	2/8/2022
2022G053	2/8/2022
2022G069	3/8/2022
2017G089	3/8/2022
2015G107	5/8/2022
2014G061	5/8/2022
2018G104/2(	6/8/2022
2014G115	5/8/2022
2017G037	6/8/2022
2022G043	6/8/2022
2018G114	6/8/2022
2019G204	6/8/2022
2014G040	6/8/2022
2011G045	6/8/2022
2017G012	7/8/2022
2022G050	8/8/2022
2018G147	8/8/2022
2017G134	9/8/2022
2019G108/2(	11/8/2022
2016G137	11/8/2022
2016G196	11/8/2022
2018G048	11/8/2022
2022G051	12/8/2022
2018G078	13/8/2022
2017G089	13/8/2022

2022G053	13/8/2022
2022G069	13/8/2022
2022G072	15/8/2022
2015G107	15/8/2022
2017G134	19/8/2022
2016G137	21/8/2022
2016G196	21/8/2022
2003G004	21/8/2022
2022G065	21/8/2022
2018G125	23/8/2022
2018G007	24/8/2022
20142120	25/8/2022
2022G073	26/8/2022
2020G007	26/8/2022
2022G072	26/8/2022
2019G204	27/8/2022
2022G074	28/8/2022
2016G137	30/8/2022
2022G065	31/8/2022
2013G101	4/9/2022
2022G072	6/9/2022
2022G074	7/9/2022
2019G268	9/9/2022
2017G158	11/9/2022
2022G072	16/9/2022
2022G075	16/9/2022
2016G137	17/9/2022
2019G204	17/9/2022
2022G074	19/9/2022
2018G175	20/9/2022
2014G040	26/9/2022
2016G137	27/9/2022
2022G075	27/9/2022
2022G072	29/9/2022
2022G076	1/10/2022
2022G075	8/10/2022
2022G072	11/10/2022
2023MS001	11/1/2023
2023MS002	12/1/2023
2023MS003	14/1/2023
2023MS004	15/1/2023
2023MS005	19/2/2023
2023MS006	20/2/2023
2023MS007	20/2/2023
2023MS008	21/2/2023
2023MS009	21/2/2023

2023MS010	22/2/2023
2023MS011	23/2/2023
2023MS012	23/2/2023
2023MS013	23/2/2023
2023MS014	26/2/2023
2023MS015	26/2/2023
2023MS016	28/2/2023
2023MS017	28/2/2023
2023MS018	28/2/2023
2023MS019	1/3/2023
2023MS020	1/3/2023
2023MS021	1/3/2023
2023MS022	4/3/2023
2020G017	6/3/2023
2023MS023	6/3/2023
2023MS024	6/3/2023
2023MS025	7/3/2023
2023MS026	7/3/2023
2023MS027	7/3/2023
2023MS028	8/3/2023
2023MS029	8/3/2023
2023MS030	10/3/2023
2023MS031	11/3/2023
2023MS032	11/3/2023
2023MS033	12/3/2023
2023MS034	13/3/2023
2023MS035	14/3/2023
2015G010	17/3/2023
2023MS036	17/3/2023
2023MS037	17/3/2023
2023MS038	18/3/2023
2023MS039	18/3/2023
2023MS040	19/3/2023
2023MS041	19/3/2023
2014G041	19/3/2023
2023MS042	19/3/2023
2023MS043	19/3/2023
2023MS044	19/3/2023
2023MS045	19/3/2023
2023MS046	19/3/2023
2023MS047	20/3/2023
2023MS048	21/3/2023
2023MS049	21/3/2023
2023MS050	21/3/2023
2023MS051	22/3/2023
2023MS052	22/3/2023

2023MS053	22/3/2023
2020G095	23/3/2023
2023MS054	23/3/2023
2004G007	24/3/2023
2023MS055	24/3/2023
2023MS056	24/3/2023
2009G006	24/3/2023
2023MS057	24/3/2023
2023MS058	24/3/2023
2023MS059	25/3/2023
2023MS060	25/3/2023
2023MS061	25/3/2023
2023MS062	26/3/2023
2023MS063	26/3/2023
2023MS064	26/3/2023
2023MS065	26/3/2023
2023MS066	26/3/2023
2023MS067	26/3/2023
2023MS068	27/3/2023
2019G026	27/3/2023
2023MS069	27/3/2023
2023MS070	28/3/2023
2015G010	28/3/2023
2023MS071	28/3/2023
2023MS072	29/3/2023
2020G083	29/3/2023
2023MS073	29/3/2023
2011G044	29/3/2023
2023MS074	29/3/2023
2023MS075	30/3/2023
2023MS076	30/3/2023
2023MS077	31/3/2023
2010G040	31/3/2023
2017G039	31/3/2023
2014G041	31/3/2023
2023MS079	31/3/2023
2023MS080	31/3/2023
2023MS081	31/3/2023
2023MS082	1/4/2023
2023MS083	1/4/2023
2023MS084	2/4/2023
2023MS085	3/4/2023
2023MS086	3/4/2023
2023MS087	3/4/2023
2023MS088	3/4/2023
2020G095	3/4/2023

1997G018	3/4/2023
2023MS089	3/4/2023
2023MS090	4/4/2023
2023MS091	4/4/2023
2009G006	4/4/2023
2023MS092	4/4/2023
2015G009	4/4/2023
2023MS093	4/4/2023
2023MS094	4/4/2023
2023MS095	5/4/2023
2019G018	5/4/2023
2023MS096	5/4/2023
2023G001	5/4/2023
2023MS097	5/4/2023
2023MS098	6/4/2023
2023G001	6/4/2023
2023MS099	6/4/2023
2023MS100	6/4/2023
2013G017	6/4/2023
2023MS101	6/4/2023
2023MS102	7/4/2023
2019G026	7/4/2023
2023MS103	7/4/2023
2023MS104	7/4/2023
2023MS105	7/4/2023
2023MS106	7/4/2023
2015G010	7/4/2023
2023MS107	7/4/2023
2023MS108	7/4/2023
2023MS109	7/4/2023
2023MS110	8/4/2023
2023MS111	8/4/2023
2013G012	8/4/2023
2023MS112	8/4/2023
2023MS113	8/4/2023
2023MS114	9/4/2023
2016G101	9/4/2023
2023MS115	9/4/2023
2020G078	10/4/2023
2020G083	10/4/2023
2023MS116	10/4/2023
2016G129	10/4/2023
2018G085	10/4/2023
2023MS117	10/4/2023
2023MS118	10/4/2023
2023MS119	10/4/2023

2023MS120	10/4/2023
2023MS121	10/4/2023
2023MS122	10/4/2023
2015G016	11/4/2023
2014G015	11/4/2023
2023MS123	11/4/2023
2010G040	11/4/2023
2014G041	11/4/2023
2023MS124	12/4/2023
2023MS125	12/4/2023
2013G038	12/4/2023
1999G024	12/4/2023
2023MS126	12/4/2023
1999G024	13/4/2023
2023MS127	13/4/2023
2023MS128	13/4/2023
2016G003	13/4/2023
2023MS129	13/4/2023
2023MS130	13/4/2023
2023MS131	13/4/2023
2023MS133	13/4/2023
2018G009	13/4/2023
2020G095	13/4/2023
2023MS134	14/4/2023
2023MS135	14/4/2023
2023MS136	15/4/2023
2023G001	15/4/2023
2023MS137	15/4/2023
2009G006	15/4/2023
2023MS138	15/4/2023
2015G010	16/4/2023
2023MS139	16/4/2023
2023MS140	16/4/2023
2023G002	16/4/2023
2023MS141	16/4/2023
2013G017	16/4/2023
2019G026	17/4/2023
2015G019	17/4/2023
2023MS142	17/4/2023
2023MS143	17/4/2023
2007G009	17/4/2023
2023MS144	17/4/2023
2023MS145	17/4/2023
2019G018	18/4/2023
2016G028	18/4/2023
2023MS146	18/4/2023

2023MS147	19/4/2023
2023MS148	19/4/2023
2023MS149	19/4/2023
2016G192	19/4/2023
2013G012	19/4/2023
2023MS150	19/4/2023
2023MS151	20/4/2023
2023MS152	20/4/2023
2023MS153	20/4/2023
2023G003	20/4/2023
2019G192	20/4/2023
2023MS154	20/4/2023
2023MS155	20/4/2023
2023MS156	20/4/2023
2015G016	20/4/2023
2023MS157	21/4/2023
2023MS158	21/4/2023
2013G038	21/4/2023
2023MS159	21/4/2023
2014G015	21/4/2023
2023MS160	21/4/2023
2017G039	22/4/2023
2014G041	22/4/2023
1999G024	22/4/2023
2011G044	23/4/2023
2018G085	23/4/2023
2023MS161	23/4/2023
2010G040	23/4/2023
2023MS162	23/4/2023
2023MS163	23/4/2023
2023MS164	23/4/2023
2020G095	24/4/2023
2016G003	24/4/2023
2023MS165	24/4/2023
2023MS166	24/4/2023
2023MS167	24/4/2023
2023MS168	24/4/2023
2023MS169	24/4/2023
2016G044	25/4/2023
2023MS170	25/4/2023
2009G006	25/4/2023
2023MS171	25/4/2023
2023MS173	25/4/2023
2023MS174	26/4/2023
2015G010	26/4/2023
2019G177	26/4/2023

2023MS175	26/4/2023
2023MS176	26/4/2023
2023MS177	26/4/2023
2023MS178	26/4/2023
2023MS179	26/4/2023
2023MS180	27/4/2023
2023G002	27/4/2023
2019G134	27/4/2023
2019G026	27/4/2023
2023MS181	27/4/2023
2023MS182	27/4/2023
2023MS183	27/4/2023
2023MS184	28/4/2023
2023MS185	28/4/2023
2023MS186	28/4/2023
2023MS187	28/4/2023
2016G101	28/4/2023
2015G019	28/4/2023
2023MS188	29/4/2023
2020G078	29/4/2023
2019G018	29/4/2023
2019G192	29/4/2023
2016G140	29/4/2023
2013G012	29/4/2023
2023MS189	29/4/2023
2023MS190	29/4/2023
2016G129	29/4/2023
2023MS191	29/4/2023
2016G192	29/4/2023
2023MS192	29/4/2023
2020G083	29/4/2023
2023MS193	29/4/2023
2023MS194	29/4/2023
2023MS195	29/4/2023
2023MS196	29/4/2023
2023MS197	30/4/2023
2013G038	30/4/2023
2023MS198	30/4/2023
2013G036	30/4/2023
2023MS199	30/4/2023
2023MS200	30/4/2023
2023MS201	30/4/2023
2023MS202	30/4/2023
2023MS203	1/5/2023
2023MS204	1/5/2023
2023MS205	1/5/2023

2023MS206	1/5/2023
2023MS207	1/5/2023
2023MS208	1/5/2023
2023G001	2/5/2023
2023MS209	2/5/2023
2023MS210	2/5/2023
2016G126	2/5/2023
2023MS211	2/5/2023
1999G024	2/5/2023
2023G001	2/5/2023
2011G044	3/5/2023
2019G092	3/5/2023
2017G039	3/5/2023
2023MS212	3/5/2023
2023MS213	3/5/2023
2014G015	3/5/2023
2010G040	3/5/2023
2019G293	3/5/2023
2023MS214	3/5/2023
2020G095	4/5/2023
2020G015	4/5/2023
2015G043	4/5/2023
2023MS215	4/5/2023
2023MS216	4/5/2023
2019G168	4/5/2023
2010G075	4/5/2023
2023MS217	4/5/2023
2023MS218	5/5/2023
2023MS219	5/5/2023
2023MS220	5/5/2023
2018G009	5/5/2023
2023G004	5/5/2023
2023MS221	5/5/2023
2023MS222	5/5/2023
2023G005	5/5/2023
2016G003	5/5/2023
2023MS223	5/5/2023
2023MS224	5/5/2023
2023MS225	5/5/2023
2023MS226	5/5/2023
2013G017	6/5/2023
2023G006	6/5/2023
2023MS227	6/5/2023
2023MS228	6/5/2023
2019G177	6/5/2023
2023MS229	6/5/2023

2023MS230	6/5/2023
2023MS231	7/5/2023
2019G073	7/5/2023
2023MS232	7/5/2023
2007G009	7/5/2023
2010G030	7/5/2023
2018G086	7/5/2023
2017G056	7/5/2023
2016G044	7/5/2023
2023MS233	7/5/2023
2023MS234	7/5/2023
2023MS235	7/5/2023
2023MS236	8/5/2023
2015G010	8/5/2023
2023MS237	8/5/2023
2015G019	8/5/2023
2023MS238	8/5/2023
2023MS239	8/5/2023
2023G007	9/5/2023
2019G192	9/5/2023
2023MS240	9/5/2023
2023G008	9/5/2023
2020G083	9/5/2023
2023G002	9/5/2023
2023MS241	9/5/2023
2023G009	9/5/2023
2023G010	9/5/2023
2023MS242	9/5/2023
2023MS243	9/5/2023
2023MS244	10/5/2023
2023MS245	10/5/2023
2020G078	10/5/2023
2023MS246	10/5/2023
2016G192	10/5/2023
2023MS247	10/5/2023
2023MS248	10/5/2023
2023MS249	10/5/2023
2023MS250	10/5/2023
2013G036	10/5/2023
2023MS251	10/5/2023
2023MS252	10/5/2023
2014G079	10/5/2023
2014G041	10/5/2023
2023MS253	10/5/2023
2023MS254	10/5/2023
2023MS255	10/5/2023

2019G018	11/5/2023
2023MS256	11/5/2023
2023MS257	11/5/2023
2023MS258	11/5/2023
2023MS259	11/5/2023
2016G150	11/5/2023
2023MS260	11/5/2023
2023MS261	11/5/2023
2023MS262	11/5/2023
2023MS263	12/5/2023
2023MS264	12/5/2023
2023MS265	12/5/2023
2017G039	12/5/2023
2018G085	12/5/2023
2013G094	12/5/2023
2023MS266	12/5/2023
2023MS267	12/5/2023
2016G126	12/5/2023
2023MS268	12/5/2023
2023MS269	12/5/2023
2023MS270	12/5/2023
2023MS271	12/5/2023
2023MS272	13/5/2023
2014G015	13/5/2023
2023MS273	13/5/2023
2021G003	13/5/2023
2013G066	13/5/2023
2023MS274	13/5/2023
2023G011	13/5/2023
2011G044	13/5/2023
2019G092	13/5/2023
2019G270	13/5/2023
2023MS275	13/5/2023
2023MS276	13/5/2023
2020G015	14/5/2023
2023MS277	14/5/2023
2019G293	14/5/2023
2023MS278	14/5/2023
2019G168	14/5/2023
2014G100	14/5/2023
2023MS279	14/5/2023
2023MS280	14/5/2023
2023MS281	15/5/2023
2023MS282	15/5/2023
2010G075	15/5/2023
2023MS283	15/5/2023

2023MS284	15/5/2023
2023MS285	15/5/2023
2023MS286	16/5/2023
2023G004	16/5/2023
2023MS288	16/5/2023
2023MS289	16/5/2023
2023MS290	16/5/2023
2023MS291	16/5/2023
2019G134	17/5/2023
2019G026	17/5/2023
2023MS292	17/5/2023
2023G006	17/5/2023
2023MS293	17/5/2023
2023MS294	17/5/2023
2023MS295	17/5/2023
2023MS296	17/5/2023
2023MS297	17/5/2023
2023MS298	17/5/2023
2023MS299	17/5/2023
2023G012	17/5/2023
2023G013	17/5/2023
2023MS300	17/5/2023
2017G041	18/5/2023
2013G012	18/5/2023
2013G038	18/5/2023
2023MS301	18/5/2023
2023MS302	18/5/2023
2023MS303	18/5/2023
2023MS304	18/5/2023
2016G140	18/5/2023
2019G192	18/5/2023
2020G133	18/5/2023
2023MS305	18/5/2023
2023MS306	18/5/2023
2014G041	18/5/2023
2023MS307	18/5/2023
2023MS308	19/5/2023
2015G019	19/5/2023
2020G078	19/5/2023
2023MS309	19/5/2023
2023MS310	19/5/2023
2023MS311	19/5/2023
2020G083	19/5/2023
2013G036	19/5/2023
2016G129	19/5/2023
2023MS312	19/5/2023

2023MS313	19/5/2023
2023MS314	20/5/2023
2023MS315	20/5/2023
2023MS316	20/5/2023
2023MS317	20/5/2023
2023MS318	20/5/2023
2023G014	20/5/2023
2018G139	20/5/2023
2023MS319	20/5/2023
2023MS320	20/5/2023
2018G085	21/5/2023
2023MS321	21/5/2023
2023MS322	21/5/2023
2023MS323	21/5/2023
2023MS324	21/5/2023
2023MS325	21/5/2023
2023MS326	21/5/2023
2023MS327	21/5/2023
2014G079	21/5/2023
2023MS328	22/5/2023
2023MS329	22/5/2023
2023MS330	22/5/2023
2023MS331	22/5/2023
2023MS332	22/5/2023
2023MS333	22/5/2023
2023MS334	22/5/2023
2023MS335	22/5/2023
2023MS336	22/5/2023
2023MS337	22/5/2023
2011G044	22/5/2023
2023G015	22/5/2023
2023MS338	23/5/2023
2023G016	23/5/2023
2013G094	23/5/2023
2020G095	23/5/2023
2016G033	23/5/2023
2023MS339	23/5/2023
2023MS340	23/5/2023
2023MS341	23/5/2023
2023MS342	23/5/2023
2023MS343	23/5/2023
2023MS344	23/5/2023
2019G018	23/5/2023
2023MS345	23/5/2023
2023MS346	23/5/2023
2023MS347	23/5/2023

2023MS348	23/5/2023
2023MS349	23/5/2023
2019G092	23/5/2023
2023MS350	23/5/2023
2023G011	23/5/2023
2023MS351	23/5/2023
2023MS352	23/5/2023
2023MS353	24/5/2023
2023MS354	24/5/2023
2020G151	24/5/2023
2023MS355	24/5/2023
2023MS356	24/5/2023
2023MS357	24/5/2023
2017G110	24/5/2023
2021G003	24/5/2023
2023MS358	24/5/2023
2023MS359	24/5/2023
2023MS360	24/5/2023
2023MS361	24/5/2023
2019G086	25/5/2023
2023MS362	25/5/2023
2023MS363	25/5/2023
2013G017	25/5/2023
2018G095	25/5/2023
2013G066	25/5/2023
2023MS364	25/5/2023
2023MS365	25/5/2023
2023MS366	25/5/2023
2023MS367	25/5/2023
2023MS368	25/5/2023
2023MS369	26/5/2023
2023MS370	26/5/2023
2023MS371	26/5/2023
2010G075	26/5/2023
2019G177	26/5/2023
2023MS372	26/5/2023
2020G186	26/5/2023
2023MS373	26/5/2023
2023MS374	26/5/2023
2023MS375	26/5/2023
2007G009	26/5/2023
2023G017	26/5/2023
2023G013	26/5/2023
2023MS376	26/5/2023
2023MS377	26/5/2023
2023MS378	26/5/2023

2023MS379	27/5/2023
2023MS380	27/5/2023
2023G006	27/5/2023
2019G192	27/5/2023
2023MS381	27/5/2023
2020G133	27/5/2023
2023MS382	27/5/2023
2023MS383	27/5/2023
2019G134	27/5/2023
2018G073	27/5/2023
2023MS384	27/5/2023
2023MS385	27/5/2023
2023G018	27/5/2023
2023MS386	27/5/2023
2023G004	27/5/2023
2023MS387	27/5/2023
2023MS388	27/5/2023
2023MS389	27/5/2023
2023MS390	27/5/2023
2023G019	28/5/2023
2023MS391	28/5/2023
2023MS392	28/5/2023
2023MS393	28/5/2023
2023MS394	28/5/2023
2014G041	28/5/2023
2023MS395	28/5/2023
2010G030	28/5/2023
2023G002	28/5/2023
2013G036	28/5/2023
2023MS396	28/5/2023
2023MS397	28/5/2023
2023MS398	28/5/2023
2023MS399	28/5/2023
2023MS400	28/5/2023
2023MS401	28/5/2023
2016G140	28/5/2023
2023MS402	28/5/2023
2023MS403	29/5/2023
2016G192	29/5/2023
2016G028	29/5/2023
2018G086	29/5/2023
2023MS405	29/5/2023
2020G078	29/5/2023
2023MS406	29/5/2023
2020G083	29/5/2023
2023MS407	29/5/2023

2020G130	29/5/2023
2017G041	29/5/2023
2014G105	29/5/2023
2023MS408	29/5/2023
2023MS409	30/5/2023
2023MS410	30/5/2023
2023MS411	30/5/2023
2023MS412	30/5/2023
2023MS413	30/5/2023
2023MS414	30/5/2023
2023MS415	30/5/2023
2023MS416	30/5/2023
2023MS417	30/5/2023
2023MS418	30/5/2023
2018G139	30/5/2023
2023MS419	30/5/2023
2023MS420	31/5/2023
2023MS421	31/5/2023
2018G085	31/5/2023
2023MS422	31/5/2023
2023MS423	31/5/2023
2023MS424	31/5/2023
2023MS425	31/5/2023
2023MS426	1/6/2023
2011G044	1/6/2023
2023MS427	1/6/2023
2023MS428	1/6/2023
2023MS429	1/6/2023
2013G094	1/6/2023
2023MS430	1/6/2023
2023MS431	1/6/2023
2023MS432	1/6/2023
2023G014	1/6/2023
2020G015	1/6/2023
2023MS433	1/6/2023
2023MS434	1/6/2023
2023MS435	1/6/2023
2023MS436	2/6/2023
2023MS437	2/6/2023
2023MS438	2/6/2023
2023MS439	2/6/2023
2019G092	2/6/2023
2023MS440	2/6/2023
2023MS441	2/6/2023
2023MS442	2/6/2023
2023MS443	2/6/2023

2023MS444	3/6/2023
2017G039	3/6/2023
2018G084	3/6/2023
2023MS445	3/6/2023
2023MS446	3/6/2023
2023MS447	3/6/2023
2023MS448	3/6/2023
2021G003	4/6/2023
2023MS449	4/6/2023
2023MS450	4/6/2023
2023MS451	4/6/2023
2023MS452	4/6/2023
2023MS453	4/6/2023
2016G003	4/6/2023
2023MS454	4/6/2023
2023MS455	4/6/2023
2018G095	5/6/2023
2023MS456	5/6/2023
2023MS457	5/6/2023
2023MS458	5/6/2023
2023MS459	5/6/2023
2017G106	5/6/2023
2020G151	5/6/2023
2023MS460	5/6/2023
2023MS461	5/6/2023
2023MS462	5/6/2023
2023MS463	5/6/2023
2010G075	5/6/2023
2019G177	5/6/2023
2016G150	5/6/2023
2017G110	5/6/2023
2023MS464	5/6/2023
2023MS465	5/6/2023
2023G017	5/6/2023
2023MS466	5/6/2023
2016G044	5/6/2023
2023MS467	6/6/2023
2023MS468	6/6/2023
2023G020	6/6/2023
2013G036	6/6/2023
2023MS469	6/6/2023
2020G133	6/6/2023
2023MS470	6/6/2023
2023MS471	6/6/2023
2013G066	6/6/2023
2023MS472	6/6/2023

2019G086	6/6/2023
2023MS473	6/6/2023
2019G293	6/6/2023
2019G192	6/6/2023
2023MS474	6/6/2023
2016G305	6/6/2023
2023G021	6/6/2023
2023MS475	6/6/2023
2023MS476	6/6/2023
2023MS477	7/6/2023
2023MS478	7/6/2023
2019G134	7/6/2023
2023MS479	7/6/2023
2023MS480	7/6/2023
2023MS481	7/6/2023
2023MS482	7/6/2023
2023MS483	7/6/2023
2023MS484	7/6/2023
2023MS485	7/6/2023
2013G038	7/6/2023
2018G073	7/6/2023
2023MS486	7/6/2023
2023G004	7/6/2023
2016G140	7/6/2023
2023MS487	7/6/2023
2023MS488	7/6/2023
2023MS489	7/6/2023
2023G019	7/6/2023
2023G006	7/6/2023
2023MS490	7/6/2023
2023MS491	7/6/2023
2023MS492	7/6/2023
2023MS493	7/6/2023
2023MS494	8/6/2023
2023MS495	8/6/2023
2023MS496	8/6/2023
2020G186	8/6/2023
1993G122	8/6/2023
2014G105	8/6/2023
2023MS497	8/6/2023
2023MS498	8/6/2023
2023MS499	8/6/2023
2023MS500	8/6/2023
2010G018	8/6/2023
2023G022	8/6/2023
2023MS501	8/6/2023

2023MS502	8/6/2023
2020G195	8/6/2023
2023MS503	8/6/2023
2023MS504	8/6/2023
2023MS505	8/6/2023
2023MS506	8/6/2023
2023G023	8/6/2023
2023MS507	8/6/2023
2023MS508	9/6/2023
2023MS509	9/6/2023
2018G086	9/6/2023
2023MS510	9/6/2023
2023MS511	9/6/2023
2023G024	9/6/2023
2023MS512	9/6/2023
2023MS513	9/6/2023
2023MS514	9/6/2023
2020G130	9/6/2023
2023MS515	9/6/2023
2023MS516	9/6/2023
2023MS517	9/6/2023
2023MS518	10/6/2023
2020G083	10/6/2023
2023MS519	10/6/2023
2023MS520	10/6/2023
2023MS521	10/6/2023
2023MS522	10/6/2023
2023MS523	10/6/2023
2023MS524	10/6/2023
2023MS525	10/6/2023
2023MS526	10/6/2023
2018G085	11/6/2023
2023MS527	11/6/2023
2023MS528	11/6/2023
2023MS529	11/6/2023
2023MS530	11/6/2023
2013G094	11/6/2023
2023MS531	11/6/2023
2023MS532	11/6/2023
2023MS533	11/6/2023
2023MS534	11/6/2023
2023G025	11/6/2023
2023MS535	11/6/2023
2023MS536	11/6/2023
2016G126	11/6/2023
2023MS537	11/6/2023

2023MS538	11/6/2023
1995G038	11/6/2023
2023MS539	11/6/2023
1996G009	12/6/2023
2023MS540	12/6/2023
2023MS541	12/6/2023
2023MS542	12/6/2023
2023G011	12/6/2023
2014G079	12/6/2023
2014G056	12/6/2023
2023MS543	12/6/2023
2018G065	12/6/2023
2023MS544	13/6/2023
2014G100	13/6/2023
2011G044	13/6/2023
2023MS545	13/6/2023
2023MS546	13/6/2023
2023MS547	13/6/2023
2023MS548	13/6/2023
2023MS549	13/6/2023
2023MS550	14/6/2023
2023MS551	14/6/2023
2023MS552	14/6/2023
2018G084	14/6/2023
2023MS553	14/6/2023
2019G092	14/6/2023
2023MS554	14/6/2023
2023MS555	14/6/2023
2023MS556	14/6/2023
2023MS557	14/6/2023
2023MS558	14/6/2023
2023MS559	14/6/2023
2023MS560	15/6/2023
2016G003	15/6/2023
2023MS561	15/6/2023
2023G014	15/6/2023
2023MS562	15/6/2023
2019G177	15/6/2023
2023MS563	15/6/2023
2023MS564	15/6/2023
2023MS565	16/6/2023
2010G075	16/6/2023
2023G009	16/6/2023
2023MS566	16/6/2023
2023MS567	16/6/2023
2023MS568	16/6/2023

2022G065	16/6/2023
2013G036	16/6/2023
2023MS569	16/6/2023
2023MS570	17/6/2023
2007G009	17/6/2023
2023MS571	17/6/2023
2023MS572	17/6/2023
2023MS573	17/6/2023
2023G020	17/6/2023
2023MS574	17/6/2023
2023MS575	17/6/2023
2017G110	17/6/2023
2023MS576	17/6/2023
2019G086	17/6/2023
2023MS577	17/6/2023
2023MS578	17/6/2023
2015G043	17/6/2023
2023MS579	17/6/2023
2023MS580	17/6/2023
2023MS581	17/6/2023
2016G044	18/6/2023
2023MS582	18/6/2023
2016G305	18/6/2023
2016G140	18/6/2023
2023MS583	18/6/2023
2023MS584	18/6/2023
2023G019	19/6/2023
2015G042	19/6/2023
2023G006	19/6/2023
2023MS585	19/6/2023
2020G186	19/6/2023
2023G026	19/6/2023
2023MS586	19/6/2023
2020G195	19/6/2023
2023G027	19/6/2023
2023MS587	19/6/2023
2023MS588	19/6/2023
2014G105	19/6/2023
2023MS589	19/6/2023
2016G192	19/6/2023
2023MS590	19/6/2023
2023MS591	19/6/2023
2023MS592	19/6/2023
2023MS593	19/6/2023
2023MS594	19/6/2023
2023G028	19/6/2023

2023MS595	20/6/2023
1993G122	20/6/2023
2014G015	20/6/2023
2023MS596	20/6/2023
2023G004	20/6/2023
2023MS597	20/6/2023
2013G012	20/6/2023
2023MS598	20/6/2023
2023G022	20/6/2023
2020G130	20/6/2023
2023MS599	20/6/2023
2023MS600	20/6/2023
2023MS601	20/6/2023
2023MS602	21/6/2023
2013G094	21/6/2023
2023MS603	21/6/2023
2023MS604	21/6/2023
2018G086	21/6/2023
2023MS605	21/6/2023
2023MS606	21/6/2023
2020G015	21/6/2023
2023MS607	21/6/2023
2023MS608	21/6/2023
2023G025	21/6/2023
2023MS609	21/6/2023
2023MS610	21/6/2023
2023MS611	21/6/2023
2023MS612	21/6/2023
2017G041	22/6/2023
2023MS613	22/6/2023
2023MS614	22/6/2023
2017G106	22/6/2023
2023MS615	22/6/2023
1995G038	22/6/2023
2023MS616	22/6/2023
2023MS617	22/6/2023
2023MS618	22/6/2023
2023MS619	22/6/2023
2023G029	22/6/2023
2023MS620	22/6/2023
2023MS621	22/6/2023
2023MS622	22/6/2023
2023MS623	22/6/2023
2018G139	22/6/2023
2023MS624	22/6/2023
2023MS625	22/6/2023

2023MS626	22/6/2023
2014G100	22/6/2023
2023MS627	22/6/2023
2023G024	22/6/2023
2023MS628	22/6/2023
2023MS629	23/6/2023
2023MS630	23/6/2023
2018G095	23/6/2023
2023MS631	23/6/2023
2023MS632	23/6/2023
2023G030	23/6/2023
2023MS633	23/6/2023
2023MS634	23/6/2023
2023MS635	23/6/2023
1996G009	23/6/2023
2023MS636	23/6/2023
2014G079	23/6/2023
2023MS637	23/6/2023
2023MS638	23/6/2023
2023MS639	23/6/2023
2023MS640	23/6/2023
2023MS641	23/6/2023
2023MS642	24/6/2023
2023MS643	24/6/2023
2023G011	24/6/2023
2023MS644	24/6/2023
2023MS645	24/6/2023
2023MS646	24/6/2023
2018G085	24/6/2023
2023MS647	24/6/2023
2023MS648	24/6/2023
2023MS649	24/6/2023
2023MS650	25/6/2023
2023MS651	25/6/2023
2023MS652	25/6/2023
2023MS653	25/6/2023
2023MS654	25/6/2023
2019G108	25/6/2023
2023MS655	25/6/2023
2023MS656	25/6/2023
2023MS657	25/6/2023
2010G075	25/6/2023
2023G034	25/6/2023
2018G065	25/6/2023
2023MS658	25/6/2023
2019G160	26/6/2023

2023MS659	26/6/2023
2016G044	26/6/2023
2023MS660	26/6/2023
2019G192	26/6/2023
2023G014	26/6/2023
2023MS661	26/6/2023
2023MS662	26/6/2023
2013G160	26/6/2023
2016G003	26/6/2023
2023MS663	26/6/2023
2023MS664	26/6/2023
2023MS665	26/6/2023
2023MS666	26/6/2023
2023MS667	26/6/2023
2023MS668	26/6/2023
2015G014	26/6/2023
2023MS669	27/6/2023
2023MS670	27/6/2023
2023MS671	27/6/2023
2023MS672	27/6/2023
2023MS673	27/6/2023
2023MS674	27/6/2023
2023G012	27/6/2023
2023MS675	27/6/2023
2023MS676	27/6/2023
2023MS677	27/6/2023
2023MS678	27/6/2023
2023MS679	27/6/2023
2023MS680	27/6/2023
2023MS681	27/6/2023
2023MS682	27/6/2023
2023MS683	27/6/2023
2023MS684	27/6/2023
2023MS685	27/6/2023
2023MS686	27/6/2023
2018G084	28/6/2023
2023MS687	28/6/2023
2015G042	28/6/2023
2023MS688	28/6/2023
2023MS689	28/6/2023
2023MS690	28/6/2023
2023G021	28/6/2023
2023MS691	28/6/2023
2023G031	28/6/2023
2023MS692	28/6/2023
2023MS693	28/6/2023

2023G026	28/6/2023
2023MS694	29/6/2023
2023MS695	29/6/2023
2020G195	29/6/2023
2019G293	29/6/2023
2023MS696	29/6/2023
2023MS697	29/6/2023
2023MS698	29/6/2023
2014G105	30/6/2023
2023MS699	30/6/2023
2023MS700	30/6/2023
2016G192	30/6/2023
2023MS701	30/6/2023
2023MS702	30/6/2023
2023MS703	30/6/2023
2023MS704	30/6/2023
2023MS705	30/6/2023
2023MS706	30/6/2023
2023MS707	30/6/2023
2023MS708	30/6/2023
2023MS709	1/7/2023
2023MS710	1/7/2023
2023MS711	1/7/2023
2013G094	1/7/2023
2023MS712	1/7/2023
2023MS713	1/7/2023
2023MS714	1/7/2023
2023MS715	1/7/2023
2023MS716	1/7/2023
2023MS717	1/7/2023
2023MS718	1/7/2023
2023MS719	1/7/2023
2023MS720	1/7/2023
2023MS721	1/7/2023
2023MS722	1/7/2023
2023MS723	1/7/2023
2023MS724	1/7/2023
2023MS725	1/7/2023
2023MS726	2/7/2023
2023MS727	2/7/2023
2020G130	2/7/2023
2023MS728	2/7/2023
2023G029	2/7/2023
2023MS729	3/7/2023
2023MS730	3/7/2023
2018G086	3/7/2023

2023MS731	3/7/2023
2023MS732	3/7/2023
2023MS733	3/7/2023
2023MS734	3/7/2023
2023MS735	3/7/2023
2023MS736	3/7/2023
2023MS737	3/7/2023
2023MS738	3/7/2023
2023MS739	3/7/2023
2023MS740	3/7/2023
2023MS741	3/7/2023
2023MS742	3/7/2023
2023MS743	3/7/2023
2018G065	3/7/2023
2023MS744	3/7/2023
2017G041	4/7/2023
2023MS745	4/7/2023
2023MS746	4/7/2023
2023MS747	4/7/2023
2023MS748	4/7/2023
2023G006	4/7/2023
2023MS749	4/7/2023
2023MS750	4/7/2023
2023MS751	4/7/2023
1996G009	4/7/2023
2023MS752	4/7/2023
2023MS753	4/7/2023
2023MS754	4/7/2023
2023MS755	4/7/2023
2023MS756	4/7/2023
2017G106	4/7/2023
2023MS757	4/7/2023
2016G003	4/7/2023
2023MS758	4/7/2023
2023MS759	4/7/2023
2023G024	4/7/2023
2023MS760	4/7/2023
2023MS761	5/7/2023
2023MS762	5/7/2023
2023MS763	5/7/2023
2023MS764	5/7/2023
2023MS765	5/7/2023
2014G056	5/7/2023
2023MS766	5/7/2023
2023MS767	5/7/2023
2023MS768	5/7/2023

2023MS769	5/7/2023
2023MS770	5/7/2023
2023MS771	5/7/2023
2023MS772	5/7/2023
2019G167	5/7/2023
2019G177	5/7/2023
2023MS773	5/7/2023
2023MS774	5/7/2023
2023MS775	6/7/2023
2023MS776	6/7/2023
2023MS777	6/7/2023
2023MS778	6/7/2023
2023MS779	6/7/2023
2023MS780	6/7/2023
2023MS781	6/7/2023
2013G160	6/7/2023
2023MS782	6/7/2023
2023MS783	6/7/2023
2015G043	6/7/2023
2023MS784	6/7/2023
2023MS785	6/7/2023
2023MS786	6/7/2023
2023MS787	6/7/2023
2023MS788	6/7/2023
2023MS789	6/7/2023
2023MS790	6/7/2023
2016G044	6/7/2023
2023MS791	6/7/2023
2023MS792	7/7/2023
2023MS793	7/7/2023
2023MS794	7/7/2023
2023MS795	7/7/2023
2023MS796	7/7/2023
2023MS797	7/7/2023
2023G032	7/7/2023
2023MS798	7/7/2023
2023MS799	7/7/2023
2016G125	7/7/2023
2023MS800	7/7/2023
2023MS801	8/7/2023
2023MS802	8/7/2023
2023MS803	8/7/2023
2023MS804	8/7/2023
2016G140	8/7/2023
2023MS805	8/7/2023
2023MS806	8/7/2023

2023G026	8/7/2023
2023MS807	8/7/2023
2013G066	8/7/2023
2023G033	8/7/2023
2023MS808	8/7/2023
2023MS809	8/7/2023
2023MS810	8/7/2023
2023MS811	8/7/2023
2023MS812	8/7/2023
2023MS813	8/7/2023
2023MS814	8/7/2023
2016G305	8/7/2023
2017G110	8/7/2023
2023MS815	9/7/2023
2023MS816	9/7/2023
2023MS817	9/7/2023
2023MS818	9/7/2023
2013G094	9/7/2023
2018G084	9/7/2023
2023G020	9/7/2023
2023G025	9/7/2023
2023MS819	9/7/2023
2023MS820	9/7/2023
2023MS821	9/7/2023
2023MS822	9/7/2023
2023MS823	9/7/2023
2023MS824	9/7/2023
2023MS825	9/7/2023
2023MS826	9/7/2023
2023MS827	9/7/2023
2023MS828	9/7/2023
2023MS829	9/7/2023
2023MS830	10/7/2023
2023MS831	10/7/2023
2023MS832	10/7/2023
2023MS833	10/7/2023
2018G139	10/7/2023
2023MS834	10/7/2023
2023MS835	10/7/2023
2023MS836	11/7/2023
2023MS837	11/7/2023
2023MS838	11/7/2023
2023MS839	11/7/2023
2014G100	11/7/2023
2016G126	11/7/2023
2014G105	11/7/2023

1995G038	11/7/2023
2023MS840	11/7/2023
2023MS841	11/7/2023
2023MS842	11/7/2023
2023MS843	11/7/2023
2023MS844	11/7/2023
2023MS845	11/7/2023
2023MS846	11/7/2023
2023MS847	11/7/2023
2023MS848	11/7/2023
2023MS849	11/7/2023
2023MS850	11/7/2023
2023MS851	11/7/2023
2023MS852	11/7/2023
2007G009	11/7/2023
2023MS853	11/7/2023
2023MS854	11/7/2023
2023MS855	11/7/2023
2023MS856	11/7/2023
2023MS857	12/7/2023
2023MS858	12/7/2023
2023MS859	12/7/2023
2023MS860	12/7/2023
2016G150	12/7/2023
2018G056	12/7/2023
2023MS861	12/7/2023
2023MS862	12/7/2023
2023MS863	12/7/2023
2023MS864	12/7/2023
2023MS865	12/7/2023
2023MS866	12/7/2023
2023MS867	12/7/2023
2023MS868	12/7/2023
2023MS869	12/7/2023
2023MS870	12/7/2023
2023MS871	13/7/2023
2023MS872	13/7/2023
2023MS873	13/7/2023
2023MS874	13/7/2023
2023MS875	13/7/2023
2023MS876	13/7/2023
2023MS877	14/7/2023
2023MS878	14/7/2023
2023MS879	14/7/2023
2023MS880	14/7/2023
2023MS881	14/7/2023

2023MS882	14/7/2023
2023MS883	14/7/2023
2023MS884	14/7/2023
2023MS885	14/7/2023
2023MS886	14/7/2023
2023MS887	14/7/2023
2023MS888	14/7/2023
2023MS889	14/7/2023
2023MS890	14/7/2023
2023MS891	14/7/2023
2023MS892	14/7/2023
2023MS893	14/7/2023
2023MS894	14/7/2023
2023MS895	14/7/2023
2023MS896	14/7/2023
2015G108	15/7/2023
2023MS897	15/7/2023
2023MS898	15/7/2023
2023MS899	15/7/2023
2023MS900	15/7/2023
2023MS901	15/7/2023
2023G027	15/7/2023
2023MS902	15/7/2023
2023MS903	16/7/2023
2004G009	16/7/2023
2023MS904	16/7/2023
2023MS905	16/7/2023
2023MS906	16/7/2023
2023MS907	16/7/2023
2023MS908	16/7/2023
2023MS909	16/7/2023
2023MS910	16/7/2023
2023G034	16/7/2023
2023MS911	16/7/2023
2023MS912	17/7/2023
2023MS913	17/7/2023
2019G313	17/7/2023
2023MS914	17/7/2023
2023MS915	17/7/2023
2023MS916	17/7/2023
2017G096	17/7/2023
2023MS917	18/7/2023
2023MS918	18/7/2023
2023MS919	18/7/2023
2023MS920	18/7/2023
2023G026	18/7/2023

2023MS921	18/7/2023
2023MS922	18/7/2023
2023MS923	18/7/2023
2019G167	18/7/2023
2023MS924	18/7/2023
2023MS925	18/7/2023
2023MS926	18/7/2023
2023MS927	18/7/2023
2023MS928	18/7/2023
2013G066	18/7/2023
2023MS929	18/7/2023
2023MS930	18/7/2023
2016G140	18/7/2023
2023MS931	18/7/2023
2023MS932	18/7/2023
2023MS933	18/7/2023
2023MS934	18/7/2023
2016G125	18/7/2023
2023G033	18/7/2023
2023MS935	18/7/2023
2023MS936	18/7/2023
2019G086	18/7/2023
2015G043	18/7/2023
2023MS937	18/7/2023
2023MS938	18/7/2023
2023MS939	18/7/2023
2023MS940	18/7/2023
2023MS941	18/7/2023
2023MS942	19/7/2023
2016G030	19/7/2023
2023MS943	19/7/2023
2023MS944	19/7/2023
2023MS945	19/7/2023
2016G232	19/7/2023
2015G038	19/7/2023
2023MS946	19/7/2023
2023MS947	19/7/2023
2023MS948	19/7/2023
2023MS949	19/7/2023
2023MS950	19/7/2023
2023MS951	19/7/2023
2023MS952	19/7/2023
2023MS953	19/7/2023
2023MS954	19/7/2023
2023MS955	19/7/2023
2023MS956	19/7/2023

2023MS957	20/7/2023
2023MS958	20/7/2023
2015G111	20/7/2023
2023G020	20/7/2023
2023MS959	20/7/2023
2023MS960	20/7/2023
2017G110	20/7/2023
2023MS961	20/7/2023
2019G293	20/7/2023
2023MS962	20/7/2023
2016G126	20/7/2023
2023MS963	20/7/2023
2023MS964	20/7/2023
2023MS965	21/7/2023
2023MS966	21/7/2023
2023MS967	21/7/2023
2023MS968	21/7/2023
2023MS969	21/7/2023
2023G036	21/7/2023
2014G105	21/7/2023
2023G028	21/7/2023
2023MS970	21/7/2023
2023MS971	21/7/2023
2023MS972	21/7/2023
2023MS973	21/7/2023
2023MS974	21/7/2023
2020G130	21/7/2023
2023MS975	21/7/2023
1995G038	21/7/2023
2023MS976	21/7/2023
2018G065	21/7/2023
2023MS977	21/7/2023
2023MS978	21/7/2023
2023MS979	21/7/2023
2023MS980	21/7/2023
2023MS981	21/7/2023
2023MS982	21/7/2023
2023MS983	21/7/2023
2023G030	22/7/2023
2020G151	22/7/2023
2023MS984	22/7/2023
2023MS985	22/7/2023
2023MS986	22/7/2023
2017G062	22/7/2023
2023MS987	22/7/2023
2023MS988	23/7/2023

2023MS989	23/7/2023
2023MS990	23/7/2023
2018G056	23/7/2023
2023MS991	23/7/2023
2023MS992	23/7/2023
2018G095	23/7/2023
2023MS993	23/7/2023
2023MS994	23/7/2023
2023MS995	23/7/2023
2023G029	23/7/2023
2023MS996	23/7/2023
2023MS997	23/7/2023
2023MS998	23/7/2023
2023MS999	23/7/2023
2023MS1000	23/7/2023
2023MS1001	23/7/2023
2023MS1002	23/7/2023
2023G037	23/7/2023
2023MS1003	23/7/2023
2023MS1004	23/7/2023
2017G106	23/7/2023
2023MS1005	24/7/2023
2023MS1006	24/7/2023
2023MS1007	24/7/2023
2023MS1008	24/7/2023
2023MS1009	24/7/2023
2023MS1010	24/7/2023
2023MS1011	24/7/2023
2023MS1012	24/7/2023
2023MS1013	24/7/2023
2023MS1014	24/7/2023
2023MS1015	24/7/2023
2023MS1016	25/7/2023
2023MS1017	25/7/2023
2023MS1018	25/7/2023
2023MS1019	25/7/2023
2023MS1020	25/7/2023
2023MS1021	25/7/2023
2023MS1022	25/7/2023
2023MS1023	25/7/2023
2023MS1024	25/7/2023
2023MS1025	25/7/2023
2023MS1026	25/7/2023
2023MS1027	25/7/2023
2016G232	25/7/2023
2023MS1028	26/7/2023

2023MS1029	26/7/2023
2023MS1030	26/7/2023
2023MS1031	26/7/2023
2023MS1032	26/7/2023
2023MS1033	26/7/2023
2023MS1034	26/7/2023
2023MS1035	26/7/2023
2023MS1036	26/7/2023
2023MS1037	26/7/2023
2023MS1038	26/7/2023
2023MS1039	26/7/2023
2023MS1040	27/7/2023
2023MS1041	27/7/2023
2013G160	27/7/2023
2023MS1042	27/7/2023
2023MS1043	27/7/2023
2004G009	27/7/2023
2023MS1044	27/7/2023
2023MS1045	27/7/2023
2023MS1046	27/7/2023
2023G032	27/7/2023
2023MS1047	28/7/2023
2023MS1048	28/7/2023
2023MS1049	28/7/2023
2023MS1050	28/7/2023
2023G033	28/7/2023
2016G030	28/7/2023
2023MS1051	28/7/2023
2023MS1052	28/7/2023
2023MS1053	28/7/2023
2023MS1054	28/7/2023
2023MS1055	28/7/2023
2023MS1056	28/7/2023
2023MS1057	29/7/2023
2023MS1058	29/7/2023
2023MS1059	29/7/2023
2019G313	29/7/2023
2023MS1060	29/7/2023
2023G021	29/7/2023
2023MS1061	29/7/2023
2009G059	29/7/2023
2023MS1062	29/7/2023
2023MS1063	29/7/2023
2023MS1064	29/7/2023
2023MS1065	30/7/2023
2023MS1066	30/7/2023

2023MS1067	30/7/2023
2023MS1068	30/7/2023
2023MS1069	30/7/2023
2023MS1070	30/7/2023
2023MS1071	30/7/2023
2017G062	31/7/2023
2023MS1072	31/7/2023
2023MS1073	31/7/2023
2020G130	31/7/2023
2023MS1074	31/7/2023
2023MS1075	31/7/2023
2023MS1076	31/7/2023
2023G038	31/7/2023
2023G030	31/7/2023
2023MS1077	31/7/2023
2023MS1078	31/7/2023
2023MS1079	31/7/2023
2023MS1080	31/7/2023
2016G232	31/7/2023
2023MS1081	31/7/2023
2023MS1082	31/7/2023
2023MS1083	31/7/2023
2023MS1084	1/8/2023
2023MS1085	1/8/2023
2023MS1086	1/8/2023
2023MS1087	1/8/2023
2023MS1088	1/8/2023
2023MS1089	1/8/2023
2023MS1090	1/8/2023
2023G036	1/8/2023
2023MS1091	1/8/2023
2023MS1092	1/8/2023
2023MS1093	1/8/2023
2001G037	1/8/2023
2023MS1094	1/8/2023
2023MS1095	1/8/2023
2023MS1096	1/8/2023
2023MS1097	1/8/2023
2023MS1098	1/8/2023
2023MS1099	1/8/2023
2023MS1100	1/8/2023
2018G095	1/8/2023
2023MS1101	2/8/2023
2023MS1102	2/8/2023
2023MS1103	2/8/2023
2023MS1104	2/8/2023

2023MS1105	2/8/2023
2023MS1106	2/8/2023
2013G066	2/8/2023
2023MS1107	2/8/2023
2023MS1108	2/8/2023
2023G029	2/8/2023
2018G056	2/8/2023
2023MS1109	2/8/2023
2023MS1110	2/8/2023
2023MS1111	2/8/2023
2023MS1112	2/8/2023
2023MS1113	2/8/2023
2023MS1114	2/8/2023
2023MS1115	2/8/2023
2023MS1116	3/8/2023
2023MS1117	3/8/2023
2023MS1118	3/8/2023
2023MS1119	3/8/2023
2023MS1120	3/8/2023
2017G106	3/8/2023
2023MS1121	4/8/2023
2023MS1122	4/8/2023
2023MS1123	4/8/2023
2023MS1124	4/8/2023
2023MS1125	4/8/2023
2023MS1126	4/8/2023
2023MS1127	4/8/2023
2023MS1128	4/8/2023
2023MS1129	4/8/2023
2023MS1130	4/8/2023
2023MS1131	4/8/2023
2023G039	4/8/2023
2023MS1132	5/8/2023
2023MS1133	5/8/2023
2023MS1134	5/8/2023
2023MS1135	5/8/2023
2023MS1136	5/8/2023
2023MS1137	5/8/2023
2023MS1138	5/8/2023
2023MS1139	5/8/2023
2023MS1140	5/8/2023
2023MS1141	5/8/2023
2023MS1142	5/8/2023
2023MS1143	5/8/2023
2023MS1144	5/8/2023
2023MS1145	5/8/2023

2023MS1146	5/8/2023
2023MS1147	5/8/2023
2013G160	6/8/2023
2010G075	6/8/2023
2023MS1148	6/8/2023
2023MS1149	6/8/2023
2023MS1150	6/8/2023
2023MS1151	6/8/2023
2023MS1152	6/8/2023
2023MS1153	7/8/2023
2023MS1154	7/8/2023
2023MS1155	7/8/2023
2023MS1156	7/8/2023
2023MS1157	7/8/2023
2017G096	7/8/2023
2023MS1158	7/8/2023
2023MS1159	7/8/2023
2023MS1160	7/8/2023
2023MS1161	7/8/2023
2023MS1162	7/8/2023
2023MS1163	7/8/2023
2023G033	8/8/2023
2023MS1164	8/8/2023
2009G059	8/8/2023
2023MS1165	8/8/2023
2023MS1166	8/8/2023
2023MS1167	8/8/2023
2023MS1168	8/8/2023
2023MS1169	8/8/2023
2023MS1170	8/8/2023
2023MS1171	8/8/2023
2023G034	8/8/2023
2023MS1172	8/8/2023
2023MS1173	9/8/2023
2023MS1174	9/8/2023
2023MS1175	9/8/2023
2023MS1176	9/8/2023
2023MS1177	9/8/2023
2016G031	9/8/2023
2019G313	9/8/2023
2018G139	10/8/2023
2023MS1178	10/8/2023
2023MS1179	10/8/2023
2023MS1180	10/8/2023
2023G030	10/8/2023
2023MS1181	10/8/2023

2023MS1182	10/8/2023
2023MS1183	10/8/2023
2023MS1184	10/8/2023
2023MS1185	10/8/2023
2023MS1186	10/8/2023
2023G021	10/8/2023
2023MS1187	10/8/2023
2023MS1188	10/8/2023
2023MS1189	11/8/2023
2023MS1190	11/8/2023
2023MS1191	11/8/2023
2023MS1192	11/8/2023
2023MS1193	11/8/2023
2017G110	11/8/2023
2019G293	11/8/2023
2023MS1194	11/8/2023
2023MS1195	11/8/2023
2023MS1196	11/8/2023
2023MS1197	11/8/2023
2023MS1198	11/8/2023
2020G195	11/8/2023
2023MS1199	11/8/2023
2023MS1200	11/8/2023
2023MS1201	11/8/2023
2023MS1202	11/8/2023
2023MS1203	12/8/2023
1993G122	12/8/2023
2023MS1204	12/8/2023
2017G106	12/8/2023
2023MS1205	12/8/2023
2023MS1206	13/8/2023
2023MS1207	13/8/2023
2023MS1208	13/8/2023
2023MS1209	13/8/2023
2014G101	13/8/2023
2023MS1210	14/8/2023
2023MS1211	14/8/2023
2023MS1212	14/8/2023
2023MS1213	14/8/2023
2023MS1214	14/8/2023
2023MS1215	14/8/2023
2023MS1216	14/8/2023
2023MS1217	14/8/2023
2023MS1218	14/8/2023
2023MS1219	15/8/2023
2023MS1220	15/8/2023

2019G167	15/8/2023
2023MS1221	15/8/2023
2023MS1222	15/8/2023
2023MS1223	15/8/2023
2023MS1224	15/8/2023
2023MS1225	15/8/2023
2023MS1226	15/8/2023
2013G160	15/8/2023
2023MS1227	15/8/2023
2023MS1228	15/8/2023
2023MS1229	15/8/2023
2023MS1230	15/8/2023
2023MS1231	15/8/2023
2023MS1232	16/8/2023
2023MS1233	16/8/2023
2023MS1234	16/8/2023
2023MS1235	16/8/2023
2023MS1236	16/8/2023
2023MS1237	16/8/2023
2023MS1238	17/8/2023
2023MS1239	17/8/2023
2023MS1240	17/8/2023
2023MS1241	17/8/2023
2023MS1242	17/8/2023
2023MS1243	17/8/2023
2004G009	17/8/2023
2017G096	17/8/2023
2023MS1244	17/8/2023
2023MS1245	17/8/2023
2023MS1246	17/8/2023
2023MS1247	18/8/2023
2023MS1248	18/8/2023
2023MS1249	18/8/2023
2023MS1250	18/8/2023
2023MS1251	18/8/2023
2023MS1252	18/8/2023
2017G062	18/8/2023
2023MS1253	18/8/2023
2023MS1254	18/8/2023
2023MS1255	18/8/2023
2023MS1256	18/8/2023
2023MS1257	18/8/2023
2023MS1258	18/8/2023
2023G030	19/8/2023
2023G033	19/8/2023
2023MS1259	19/8/2023

2023MS1260	19/8/2023
2023MS1261	19/8/2023
2023MS1262	19/8/2023
2023MS1263	19/8/2023
2023MS1264	19/8/2023
2023MS1265	19/8/2023
2023MS1266	19/8/2023
2023MS1267	20/8/2023
2019G313	20/8/2023
2023MS1268	20/8/2023
2016G031	20/8/2023
2023MS1269	20/8/2023
2023MS1270	20/8/2023
2023MS1271	20/8/2023
2023MS1272	20/8/2023
2023MS1273	20/8/2023
2018G139	20/8/2023
2023MS1274	20/8/2023
2023MS1275	21/8/2023
2023MS1276	21/8/2023
2023MS1277	21/8/2023
2023MS1278	21/8/2023
2023MS1279	21/8/2023
2023MS1280	21/8/2023
2023MS1281	21/8/2023
2023MS1282	22/8/2023
2015G111	22/8/2023
2023MS1283	22/8/2023
2023MS1284	22/8/2023
1993G122	23/8/2023
2023MS1285	23/8/2023
2023MS1286	23/8/2023
2023MS1287	23/8/2023
2023MS1288	23/8/2023
2023MS1289	24/8/2023
2023MS1290	24/8/2023
2023MS1291	24/8/2023
2023MS1292	24/8/2023
2023MS1293	24/8/2023
2023MS1294	25/8/2023
2023MS1295	25/8/2023
2023MS1296	25/8/2023
2023MS1297	25/8/2023
2023MS1298	25/8/2023
2013G160	25/8/2023
2023MS1299	25/8/2023

2023MS1300	26/8/2023
2023MS1301	26/8/2023
2023MS1302	26/8/2023
2023MS1303	26/8/2023
2015G064	26/8/2023
2018G146	26/8/2023
2023MS1304	26/8/2023
2023MS1305	26/8/2023
2023MS1306	26/8/2023
2023MS1307	26/8/2023
2023MS1308	26/8/2023
2023MS1309	27/8/2023
2023MS1310	27/8/2023
2016G305	27/8/2023
2023MS1311	27/8/2023
2023MS1312	27/8/2023
2023G040	27/8/2023
2023MS1313	27/8/2023
2023MS1314	27/8/2023
2017G062	28/8/2023
2023MS1315	28/8/2023
2023MS1316	28/8/2023
2023MS1317	28/8/2023
2023MS1318	28/8/2023
2023MS1319	28/8/2023
2023MS1320	28/8/2023
2023MS1321	28/8/2023
2023MS1322	28/8/2023
2023MS1323	28/8/2023
2023MS1324	28/8/2023
2023MS1325	29/8/2023
2023MS1326	29/8/2023
2023MS1327	29/8/2023
2023MS1328	29/8/2023
2023MS1329	29/8/2023
2023MS1330	29/8/2023
2023MS1331	29/8/2023
2017G096	29/8/2023
2023MS1332	29/8/2023
2023MS1333	29/8/2023
2023MS1334	29/8/2023
2023MS1335	29/8/2023
2023MS1336	30/8/2023
2023MS1337	30/8/2023
2023MS1338	30/8/2023
2019G313	30/8/2023

2023MS1339	30/8/2023
2023MS1340	31/8/2023
2023MS1341	31/8/2023
2023MS1342	31/8/2023
2023MS1343	31/8/2023
2023MS1344	1/9/2023
2023MS1345	1/9/2023
2023MS1346	1/9/2023
2023MS1347	1/9/2023
2023MS1348	1/9/2023
2023MS1349	1/9/2023
2023MS1350	2/9/2023
2023MS1351	2/9/2023
2023MS1352	2/9/2023
2023MS1353	2/9/2023
2016G321	3/9/2023
2023MS1354	3/9/2023
2015G111	3/9/2023
2023MS1355	3/9/2023
2023MS1356	3/9/2023
2013G160	3/9/2023
2023MS1357	3/9/2023
2023MS1358	4/9/2023
2023MS1359	5/9/2023
2023MS1360	5/9/2023
2023MS1361	5/9/2023
2023MS1362	5/9/2023
2018G146	5/9/2023
2023MS1363	5/9/2023
2017G062	6/9/2023
2023MS1364	6/9/2023
2023MS1365	6/9/2023
2023MS1366	6/9/2023
2023MS1367	6/9/2023
2023MS1368	6/9/2023
2023G041	6/9/2023
2023MS1369	7/9/2023
2023MS1370	7/9/2023
2020G130	7/9/2023
2023MS1371	7/9/2023
2023MS1372	7/9/2023
2009G059	7/9/2023
2023MS1373	7/9/2023
2023MS1374	7/9/2023
2004G009	8/9/2023
2023MS1375	8/9/2023

2023MS1376	8/9/2023
2023MS1377	8/9/2023
2023MS1378	8/9/2023
2023MS1379	9/9/2023
2023MS1380	9/9/2023
2023MS1381	9/9/2023
2023MS1382	9/9/2023
2023MS1383	9/9/2023
2023MS1384	10/9/2023
2023MS1385	10/9/2023
2023MS1386	10/9/2023
2023MS1387	10/9/2023
2023MS1388	11/9/2023
2019G313	11/9/2023
2023G042	12/9/2023
2015G111	12/9/2023
2023MS1389	12/9/2023
2023MS1390	14/9/2023
2023MS1391	14/9/2023
2023MS1392	14/9/2023
2023MS1393	15/9/2023
2023MS1394	15/9/2023
2023MS1395	15/9/2023
2023MS1396	15/9/2023
2023MS1397	15/9/2023
2023MS1398	15/9/2023
2023MS1399	17/9/2023
2023MS1400	17/9/2023
2023MS1401	17/9/2023
2023MS1402	17/9/2023
2023G041	18/9/2023
2023MS1403	20/9/2023
2023G042	21/9/2023
2023MS1404	21/9/2023
2023MS1405	21/9/2023
2023MS1406	22/9/2023
2023MS1407	22/9/2023
2015G111	23/9/2023
2023MS1408	23/9/2023
2023MS1409	24/9/2023
2023G043	24/9/2023
2023MS1410	24/9/2023
2023MS1411	24/9/2023
2023MS1412	25/9/2023
2023MS1413	25/9/2023
2023MS1414	26/9/2023

2023MS1415	26/9/2023
2023MS1416	26/9/2023
2023MS1417	28/9/2023
2009G059	28/9/2023
2023MS1418	1/10/2023
2015G064	1/10/2023
2023MS1419	2/10/2023
2023G042	2/10/2023
2023MS1420	2/10/2023
2023MS1421	3/10/2023
2023MS1422	3/10/2023
2015G111	3/10/2023
2017G138	4/10/2023
2023G043	4/10/2023
2023MS1423	5/10/2023
2023MS1424	5/10/2023
2023MS1425	6/10/2023
2023MS1426	7/10/2023
2023MS1427	9/10/2023
2014G123	10/10/2023
2023MS1428	12/10/2023
2018G138	12/10/2023
2023MS1429	13/10/2023
2023MS1430	13/10/2023
2023MS1431	13/10/2023
2015G111	14/10/2023
2017G138	14/10/2023
2023MS1432	18/10/2023
2023MS1433	19/10/2023
2023MS1434	22/10/2023
2023MS1435	23/10/2023
2023MS1436	23/10/2023
2023MS1437	25/10/2023
2023MS1438	26/10/2023
2023MS1439	27/10/2023
2023MS1440	2/11/2023
2023MS1441	3/11/2023
2023MS1442	4/11/2023
2023MS1443	8/11/2023
2023MS1444	10/11/2023
2023MS1445	12/11/2023
2023MS1446	13/11/2023
2023MS1447	17/11/2023
2023MS1448	19/11/2023
2008G006	25/2/2024
2021G023	10/3/2024

2014G091	17/3/2024
2021G023	21/3/2024
2012G003	25/3/2024
2014G091	27/3/2024
2018G069	27/3/2024
2019G005	31/3/2024
2021G023	1/4/2024
2014G091	6/4/2024
2016G046	6/4/2024
2021G088	7/4/2024
2018G069	15/4/2024
2014G091	16/4/2024
2012G003	17/4/2024
2016G046	17/4/2024
2012G003	18/4/2024
2018G028	20/4/2024
2021G023	22/4/2024
2021G022	23/4/2024
2019G005	24/4/2024
2018G069	24/4/2024
2014G091	26/4/2024
2016G046	27/4/2024
2012G003	28/4/2024
2019G101	30/4/2024
2016G165	30/4/2024
2011G082	30/4/2024
2021G023	2/5/2024
2018G028	2/5/2024
2019G005	6/5/2024
2009G003	7/5/2024
2016G046	7/5/2024
2012G003	8/5/2024
2012G036	8/5/2024
2016G120	9/5/2024
2015G001	9/5/2024
2011G082	10/5/2024
2019G101	12/5/2024
2018G028	12/5/2024
2014G068	12/5/2024
2018G069	12/5/2024
2018G021	14/5/2024
2018G103	14/5/2024
2016G126	16/5/2024
2016G046	16/5/2024
2016G112	16/5/2024
2017G024	18/5/2024

2016G120	19/5/2024
2012G036	19/5/2024
2011G082	21/5/2024
2016G087	21/5/2024
2021G023	22/5/2024
2021G022	22/5/2024
2018G028	22/5/2024
2010G100	22/5/2024
2019G101	22/5/2024
2018G021	23/5/2024
2016G126	24/5/2024
2018G103	25/5/2024
2016G046	25/5/2024
2019G156	26/5/2024
2014G073	28/5/2024
2016G120	30/5/2024
2012G036	30/5/2024
2016G087	31/5/2024
2011G082	31/5/2024
2018G028	31/5/2024
2006G025	31/5/2024
2014G068	1/6/2024
2016G126	1/6/2024
2019G224	2/6/2024
2018G021	2/6/2024
2018G040	3/6/2024
2019G156	6/6/2024
2014G073	7/6/2024
2021G024	8/6/2024
2012G036	9/6/2024
2018G142	9/6/2024
2010G100	9/6/2024
2016G225	9/6/2024
2019G093	10/6/2024
2016G126	10/6/2024
2018G028	10/6/2024
2011G082	9/6/2024
2016G120	11/6/2024
2019G101	11/6/2024
2014G068	11/6/2024
2016G224	5/7/2024
2019G156	5/7/2024
2016G176	6/7/2024
2010G100	6/7/2024
